# Development and validation of a customised PRO-CTCAE scale for adult-type diffuse gliomas (VERONICA): a multicentre, prospective, observational cohort study in China

**DOI:** 10.1016/j.eclinm.2026.103879

**Published:** 2026-04-10

**Authors:** Sida Song, Xiefeng Wang, Xiangyi Cao, Chen Luo, Kunpeng Na, Chengcheng Guo, Jiankun Xu, Menglin Nie, Chaoxi Li, Qingting Huang, Sanzhong Li, Hucheng Wang, Hongxiang Wang, Min Liu, Yongjie Wang, Chongran Sun, Zhang Xiong, Zhenyu Wu, Yajie Sun, Chunxia Ni, Wei Zhang, Zanyi Wu, Song Chen, Yourui Zou, Xiaochuan Sun, Hui Ma, Jianmin Zhang, Chunlong Zhong, Juxiang Chen, Zhou Fei, Lin Kong, Yongping You, Jinsong Shen, Ying Cao, Lu Zhang, Shuai Wu, Ye Yao, Kai Shu, Xiaoguang Qiu, Jie Tang, Yonggao Mou, Zhiwei Tang, Linbo Cai, Junxia Zhang, Jinsong Wu

**Affiliations:** a Department of Neurosurgery, Huashan Hospital, Shanghai Medical College, Fudan University, Shanghai, China; b Department of Neurosurgery, The First Affiliated Hospital of Nanjing Medical University, Nanjing, Jiangsu, China; c Department of Oncology, Guangdong Sanjiu Brain Hospital, Guangzhou, Guangdong, China; d Department of Neurosurgery, The First Affiliated Hospital of Kunming Medical University, Kunming, Yunnan, China; e Department of Neurosurgery/Neuro-Oncology, State Key Laboratory of Oncology in South China, Guangdong Provincial Clinical Research Center for Cancer, Sun Yat-Sen University Cancer Center, Guangzhou, Guangdong, China; f Department of Neurosurgery, Xuanwu Hospital, Capital Medical University, Beijing, China; g Department of Radiation Oncology, Beijing Tiantan Hospital, Capital Medical University, Beijing, China; h Department of Neurosurgery, Tongji Hospital, Tongji Medical College, Huazhong University of Science and Technology, Wuhan, Hubei, China; i Department of Radiation Oncology, Shanghai Proton and Heavy Ion Center, Fudan University Cancer Hospital, Shanghai, China; j Department of Neurosurgery, Xijing Hospital, Fourth Military Medical University, Xi'an, Shaanxi, China; k Department of Neurosurgery, Changhai Hospital, Naval Medical University (Second Military Medical University), Shanghai, China; l Department of Neurosurgery, Shanghai East Hospital, Tongji University School of Medicine, Shanghai, China; m Department of Neurosurgery, The Second Affiliated Hospital, School of Medicine, Zhejiang University, Hangzhou, Zhejiang, China; n Department of Biostatistics, School of Public Health, Fudan University, Shanghai, China; o Department of Neurosurgery, The Affiliated Yijishan Hospital of Wannan Medical College, Wuhu, Anhui, China; p Department of Radiation Oncology, Shanghai Gamma Hospital, Shanghai, China; q Department of Neurosurgery, Tsinghua University Beijing Tsinghua Changgung Hospital, Beijing, China; r Department of Neurosurgery, Neurosurgery Research Institute, The First Affiliated Hospital, Fujian Medical University, Fuzhou, Fujian, China; s Department of Neurosurgery, General Hospital of Ningxia Medical University, Yinchuan, Ningxia, China; t Department of Neurosurgery, The First Affiliated Hospital of Chongqing Medical University, Chongqing, China; u Nursing Department, Huashan Hospital, Shanghai Medical College, Fudan University, Shanghai, China

**Keywords:** Patient-reported outcomes, PRO-CTCAE, Adult-type diffuse glioma, Symptomatic adverse events, Psychometric validation, Observational cohort study

## Abstract

**Background:**

Patient-reported outcomes (PROs) are essential for assessing symptomatic adverse events (AEs) from a patient perspective, which significantly impact the quality of life and clinical outcomes in patients with glioma. However, no validated patient-reported outcome measures (PROMs) exist to quantify symptomatic AEs in adult-type diffuse gliomas.

**Methods:**

The study was conducted in two parts. First, we developed a customised Patient-Reported Outcomes version of the Common Terminology Criteria for Adverse Events (PRO-CTCAE) scale for adult-type diffuse gliomas using the Simplified Chinese PRO-CTCAE® item library, informed by initial item screening, patient pilot testing, and a two-round Delphi survey. Delphi experts were recruited through the National Glioma Multidisciplinary Team (MDT) Alliance (NGMA) and invited by email in June 2022 (1st round) and August 2022 (2nd round). We subsequently conducted a multicentre, prospective, observational cohort study (VERONICA) at 13 glioma treatment centres in China between September 2022 and March 2025. Eligible participants were adults aged 18 years or older with a diagnosis of adult-type diffuse glioma, who were able to understand and complete the questionnaires; patients with severe cognitive impairment, severe language dysfunction, or other conditions precluding questionnaire completion were excluded. The primary outcome was the psychometric performance of the customised PRO-CTCAE scale, including test-retest reliability, convergent validity, known-groups validity, and responsiveness, evaluated longitudinally across repeated study visits. VERONICA is registered with ClinicalTrials.gov, NCT05486923.

**Findings:**

For the Delphi survey, all seven invited experts from six centres participated in 1st round (response rate 100·0%), with moderate agreement in symptom rankings (Kendall's W = 0·415; p < 0·001). In 2nd round, 16 of 20 invited experts from 14 centres participated (response rate 80·0%), with consistent agreement in expert ratings (Kendall's W = 0·351; p < 0·001). The final version of the customised PRO-CTCAE scale comprised 53 items covering 31 symptoms, together with one open-ended free-text item. For VERONICA, 450 participants were enrolled across 13 glioma treatment centres. Mean age was 49·1 years (SD 12·8), and the mean Karnofsky Performance Status (KPS) at baseline (Visit 2) was 72·2 (SD 17·1). 424 provided data eligible for at least one prespecified psychometric analysis. Test-retest reliability was acceptable (intraclass correlation coefficient [ICC] ≥0·70 for 47 of 53 items). Convergent validity was supported by correlations in the expected direction with matched European Organisation for Research and Treatment of Cancer Quality of Life Questionnaire-Core 30 (EORTC QLQ-C30) domains, with predominantly moderate-to-strong associations (25 items with r ≥ 0·50). Known-groups validity was supported by discrimination between KPS <70 and ≥70 (Cohen's d ≥ 0·20 for 49 of 53 items; p < 0·05 for 43 of 49 items). In Global Impression of Change (GIC)-anchored responsiveness analyses, 37 items showed standardised response means (SRMs) ≥0·20 among participants reporting worsened overall status.

**Interpretation:**

The customised PRO-CTCAE scale showed robust psychometric performance for adult-type diffuse gliomas. Remote, longitudinal administration supports low-burden quantification of patient-reported symptomatic AEs in clinical trials and routine neuro-oncology practice. Future work should assess implementation in routine care and clinical trials, and extend translation, cultural adaptation, and validation across different languages.

**Funding:**

Beijing Medical Award Foundation; Shanghai Municipal Health Commission; Department of Science and Technology of Ningxia Hui Autonomous Region; Huashan Hospital, Fudan University (Clinical Research Project).

**Research in context**

**Evidence before this study**

We searched *ClinicalTrials.gov* for completed interventional phase II/III oncology clinical trials using glioma-related condition terms and patient-reported outcome (PRO)-related terms from Jan 1, 2010, to May 15, 2022, and identified 100 registered studies. We also searched PubMed, ScienceDirect, Google Scholar, and China National Knowledge Infrastructure (CNKI) from Jan 1, 2010, to May 15, 2022, using the terms “PRO”, “EORTC QLQ-C30”, “PRO-CTCAE”, “glioma”, “symptomatic adverse events”, “reliability”, “validity”, and “responsiveness”, included records published in English and Chinese, and identified 4983 records. We also hand-searched relevant guidelines and position papers, including The U.S. Food and Drug Administration (FDA) PRO guidance, Response Assessment in Neuro-Oncology Patient-Reported Outcome (RANO-PRO) recommendations, and Setting International Standards in Analysing Patient-Reported Outcomes and Quality of Life Endpoints in Cancer Clinical Trials—Innovative Medicines Initiative (SISAQoL-IMI) materials. The final search was conducted on May 15, 2022. PRO use in glioma research and oncology trials has predominantly focused on health-related quality of life and broader functioning, with limited and inconsistent patient-reported quantification of symptomatic adverse events (AEs). The National Cancer Institute Patient-Reported Outcomes version of the Common Terminology Criteria for Adverse Events (NCI PRO-CTCAE®) is a standardised, pan-tumour PRO instrument aligned with CTCAE and specifically designed to quantify symptomatic AEs directly from patients. However, we identified no glioma-specific psychometric validation studies of PRO-CTCAE. Existing glioma PRO research has largely relied on generic instruments such as the European Organisation for Research and Treatment of Cancer Quality of Life Questionnaire-Core 30 (EORTC QLQ-C30), which are not designed to systematically assess symptomatic AEs in a population characterised by distinct neurological symptoms, treatment-related toxicities, and prolonged disease trajectories.

**Added value of this study**

Using a structured, evidence-based approach incorporating expert consensus and patient input, we developed a customised PRO-CTCAE scale for adult-type diffuse gliomas. We then conducted a large, multicentre, prospective observational study to rigorously evaluate its reliability, validity, and responsiveness. The customised scale demonstrated robust psychometric performance across tumour subtypes and treatment regimens and was feasible for remote, longitudinal electronic PRO (ePRO) collection. It also enables more harmonised assessment of symptomatic AE burden and improves comparability across centres and studies. To our knowledge, this study provides the first glioma-specific validation of the PRO-CTCAE for the systematic quantification of symptomatic AEs and generates high-quality real-world data on postoperative and treatment-related symptom burden in glioma patients. Moreover, the customised scale focuses on meaningful, clinically relevant items, thereby minimising respondent burden and improving feasibility for patients.

**Implications of all the available evidence**

Our findings support the use of a psychometrically validated, glioma-specific customised PRO-CTCAE scale for adult-type diffuse gliomas to complement clinician-reported toxicity assessments in both clinical trials and routine practice. The development and validation framework presented here offers a replicable model for adapting PRO-CTCAE to other tumour types. Broader implementation of validated symptomatic AE patient-reported outcome measures (PROMs) may improve safety evaluation, enhance patient-centred care, and strengthen the interpretability of treatment tolerability across oncology studies. Future research should evaluate implementation in routine care and clinical trials, and extend translation, cross-cultural adaptation, and validation of this approach across different languages and healthcare settings.

## Introduction

As treatment options expand, systematic collection of treatment-related adverse events (AEs) has become increasingly important.^1^ Although AEs are not typically primary endpoints, symptomatic AEs can substantially impair patients’ quality of life (QoL) and may even affect key outcomes, including survival.^2^, ^3^, ^4^ However, comprehensive, systematic, standardised, and quantifiable assessment of symptomatic AEs remains challenging in clinical research. Symptom burden may be incompletely captured by clinician assessment,^5^^,^^6^ and clinician-reported grading systems (e.g., CTCAE) can underestimate the frequency and severity of symptomatic AEs compared with patient report.^7^ Moreover, intermittent or individualised symptoms may be missed, resulting in incomplete characterisation of treatment tolerability.^7^

Adult-type diffuse gliomas are the most common malignant tumours of the central nervous system.^8^ They are characterised by limited therapeutic options and generally poor prognosis.^8^ In glioma, a substantial proportion of the symptom burden is subjective, fluctuating, and functionally consequential, including fatigue, sleep disturbance, anxiety, seizure-related impacts, sexual dysfunction, and so on.^9^^,^^10^ With advances in research, an increasing number of innovative therapeutic approaches are being validated in glioma clinical trials, further amplifying the need for rigorous, patient-centred assessment of treatment-related symptomatic AEs. International initiatives, including the Response Assessment in Neuro-Oncology Patient-Reported Outcome (RANO-PRO) initiative, have highlighted that traditional endpoints can fail to capture patients’ real-world experiences and have emphasised standardisation of symptom and function assessment in glioma studies.^9^ Nevertheless, prior patient-reported outcome (PRO) assessment in glioma research has largely focused on health-related quality of life (HRQoL) and broader functional domains, whereas systematic, standardised quantification of symptomatic AEs has been less consistent.^10^ Consequently, there is a clear need for a structured and patient-centred approach to quantifying symptomatic AEs in adult-type diffuse glioma trials and routine care.

The U.S. Food and Drug Administration (FDA) defined patient-reported outcomes (PROs) as a measurement based on a report that comes directly from the patient (i.e., the study subject) about the status of a patient's health condition without interpretation of the patient's response by a clinician or anyone else.^11^ PROs provide a direct and efficient means of capturing patients' subjective perceptions of symptoms, treatment effects, and adverse reactions, and therefore complement traditional clinical endpoints such as survival, tumour response, and clinician-reported AEs.^12^, ^13^, ^14^ Although PRO endpoints in glioma studies have most commonly been implemented as secondary or exploratory measures, they are increasingly advocated in neuro-oncology outcome assessment frameworks.^9^^,^^15^

Among the most widely used patient-reported outcome measures (PROMs) in oncology research are the European Organisation for Research and Treatment of Cancer Quality of Life Questionnaire-Core 30 (EORTC QLQ-C30) and the National Cancer Institute Patient-Reported Outcomes version of the Common Terminology Criteria for Adverse Events (NCI PRO-CTCAE®), both characterised by standardised, modular design.^16^^,^^17^ The QLQ-C30 primarily assesses HRQoL, functional status, and key symptom domains, whereas the PRO-CTCAE® is specifically designed to quantify symptomatic AEs directly from patients and is increasingly regarded as a preferred PROM for AE assessment in clinical trials.^16^^,^^17^ The PRO-CTCAE® includes 78 symptomatic AEs suitable for patient self-reporting, represented by one to three items evaluating frequency (F), severity (S), interference with daily activities (I), presence/absence (P), and amount (A), resulting in 124 structured items (Supplementary Fig. S1).^18^ The PRO-CTCAE® has been linguistically validated across multiple cancer types and translated into various languages, including Simplified Chinese.^19^^,^^20^

However, symptom relevance and toxicity profiles vary substantially across tumour entities and care pathways.^21^ Routine administration of the full PRO-CTCAE® item library may therefore be burdensome and may not be optimal for a specific tumour type, intervention, or follow-up context. Careful selection of the most relevant symptomatic AEs is essential to maximise informational value while minimising response burden.^17^ Accordingly, tumour-specific PRO-CTCAE scales have been developed in several cancer populations using structured consensus methods, but a comparable, systematically developed and validated customised PRO-CTCAE scale remains largely lacking for adult-type diffuse gliomas.^22^, ^23^, ^24^, ^25^, ^26^, ^27^, ^28^, ^29^ Given the distinct neurological symptomatology, treatment trajectories, and the known limitations of clinician-reported toxicity grading in capturing patient experience, a glioma-tailored PRO-CTCAE scale could enable standardised, low-burden quantification of symptomatic AEs in both trials and routine neuro-oncology practice.

In this manuscript, we report the development of a customised PRO-CTCAE scale for adult-type diffuse gliomas using patient pilot testing and a two-round Delphi survey, and we present the results of a prospective, multicentre, observational cohort study designed to evaluate its reliability, validity, and responsiveness.

## Methods

### Study design

**Fig. 1:**
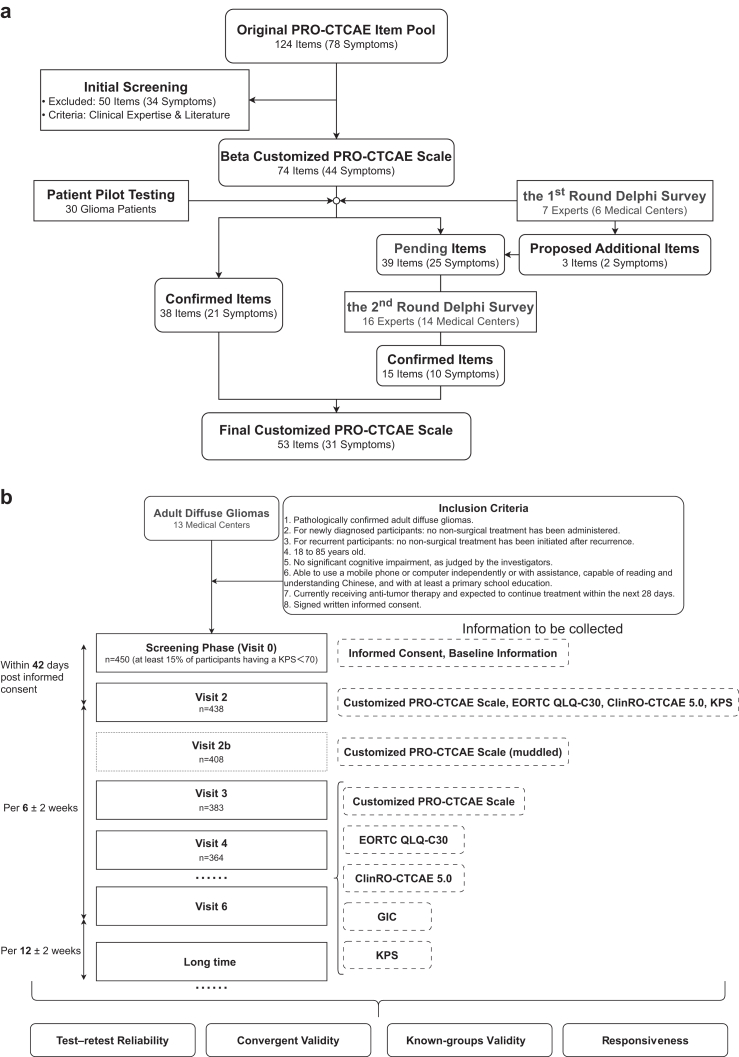
**Clinical Study Workflow. a. Scale development.** Starting from the original PRO-CTCAE® item library (124 items; 78 symptoms), an initial screening based on clinical expertise and literature excluded 50 items (34 symptoms) to form a beta customised scale (74 items; 44 symptoms). The beta scale was refined using patient pilot testing (n = 30) and a two-round Delphi process (1st round: 7 experts from 6 centres; 2nd round: 16 experts from 14 centres), including consideration of proposed additional items (3 items; 2 symptoms). The final customised scale comprised 53 items covering 31 symptoms. **b. VERONICA study procedures and assessment schedule.** Adults with diffuse gliomas were enrolled across 13 centres (screening phase, Visit 0; n = 450). Baseline assessments were completed at Visit 2 (n = 438) within 42 days after informed consent and included the customised PRO-CTCAE scale, EORTC QLQ-C30, clinician-reported symptomatic adverse events from CTCAE v5·0 (ClinRO-CTCAE 5·0), and Karnofsky Performance Status (KPS). Test-retest reliability was assessed using the re-administered customised PRO-CTCAE at Visit 2b (n = 408; item order randomised). Remote follow-up visits were conducted every 6 ± 2 weeks (e.g., Visit 3; n = 383; Visit 4; n = 364) with the customised PRO-CTCAE, and convergent/known-groups validity and responsiveness were evaluated using prespecified anchors (QLQ-C30, KPS, and Global Impression of Change [GIC]). **Abbreviations:** PRO-CTCAE, Patient-Reported Outcomes Version of the Common Terminology Criteria for Adverse Events; EORTC QLQ-C30, European Organisation for Research and Treatment of Cancer Quality of Life Questionnaire-Core 30; ClinRO-CTCAE 5·0, Clinician-Reported Symptomatic Adverse Events from *Clinician-Reported Common Terminology Criteria for Adverse Events (CTCAE) v5·0*, Corresponding to the Customised PRO-CTCAE Scale; KPS, Karnofsky Performance Status; GIC, Global Impression of Change.

This study was conducted in two stages. In the first stage (Fig. 1a), we developed a customised PRO-CTCAE scale for adult-type diffuse gliomas using the PRO-CTCAE® item library as the item pool. A beta version was generated through initial screening informed by clinical experience and a targeted literature review,^3^^,^^5^^,^^30^ and was subsequently refined through patient pilot testing and a two-round Delphi survey (Supplementary Figs. S2–S3). As the leading institution of the National Glioma Multidisciplinary Team (MDT) Alliance (NGMA) at the National Medical Centre for Neurological Diseases, we mobilised experts and patients from more than ten major glioma treatment centres in China to co-develop and validate the scale, thereby supporting its broader standardised adoption.

In the second stage (Fig. 1b), we conducted a multicentre, prospective, observational cohort study (VERONICA; *ClinicalTrials.gov*: NCT05486923) at 13 glioma treatment centres in China (September 2022 to March 2025) to evaluate the reliability, validity, and responsiveness of the customised scale.^18^^,^^31^ The study was reported in accordance with the STROBE guidelines.^32^

### Ethics

The study protocol was approved by the Huashan Institutional Review Board (HIRB), Huashan Hospital, Fudan University (approval number KY2022-681), and by the institutional review boards of all other participating centres (13 centres in total). The full names of the approving authorities and their corresponding approval numbers are provided in the Supplementary Materials (Supplementary Table S1). The full study protocol is also available in the Supplementary Materials. In the first stage of the study, clinical experts participated voluntarily; the invitation letter described the purpose of the survey, confidentiality safeguards, and intended use of the data, and questionnaire completion was taken as implied consent. In the second stage, written informed consent was obtained from all patient participants before any study procedures were undertaken.

### Participants

#### Delphi experts

Experts were eligible if they were practising in a tertiary/referral centre with an established glioma programme, had at least 10 years of clinical and/or research experience in glioma management, had experience in clinical trials and/or routine clinical outcome assessment in neuro-oncology, and were willing to participate within the specified timeline.

#### Patient participants

Participants in the pilot testing and VERONICA study who completed the customised PRO-CTCAE scale were adults aged 18–85 years who met all prespecified inclusion criteria and no exclusion criteria. Key eligibility criteria included an integrated diagnosis of adult-type diffuse glioma according to the 2021 WHO CNS5 classification, including IDH-mutant astrocytoma, IDH-mutant and 1p/19q-codeleted oligodendroglioma, and IDH-wildtype glioblastoma. Newly diagnosed patients had not received any non-surgical treatment, while recurrent patients were required to be within 42 days of recurrence confirmation based on RANO criteria. All participants were planned to initiate or continue anti-tumour therapy within 28 days and provided written informed consent. Full eligibility criteria, including exclusion and withdrawal criteria, are provided in the Supplementary Method S3.

### Part 1: scale development via Delphi survey

All candidate items were selected from the official Simplified Chinese version of the NCI PRO-CTCAE® item library and were administered without any modification to item wording, response options, or scoring, in accordance with the PRO-CTCAE® Measurement System.^17^ In initial screening, items considered less relevant to adult-type diffuse gliomas and neurosurgical/neuro-oncology care pathways were removed to generate a beta customised scale (Supplementary Fig. S2).^5^^,^^6^^,^^10^

Patient pilot testing was conducted at the lead centre (Huashan Hospital) to assess feasibility and to inform expert consensus (Supplementary Method S1). Eligible participants completed the beta scale once.^33^ Spearman rank correlations (r_s) between each symptom score and the total score and patient-reported positivity rates (proportion reporting at least mild symptoms) for each symptom were summarised.^34^^,^^35^

A two-round Delphi survey was then performed to determine the final version of the glioma-specific customised PRO-CTCAE scale (Supplementary Method S2). Experts were recruited by email through the NGMA in June 2022 (1st round) and August 2022 (2nd round). The Delphi survey was administered electronically using a secure web-based platform. Experts anonymously rated each symptom using a five-point Likert scale to minimise dominance bias and facilitate independent judgement, and agreement was assessed after each round using Kendall's coefficient of concordance (Kendall's W).^36^, ^37^, ^38^

In the 1st round Delphi survey, experts independently rated the clinical importance of each symptom in the beta scale using a five-point Likert scale, blinded to pilot testing results.^36^^,^^37^ Using prespecified thresholds, symptoms with mean importance rating >3 and symptom-total r_s ≥ 0·30 were provisionally classified as confirmed; all others were classified as pending. Summary statistics and the full PRO-CTCAE® items were then shared with experts for comments and suggestions. In the 2nd round Delphi survey, experts re-rated all pending symptoms. We calculated the mean importance score and coefficient of variation (CV) for each symptom and the item-level content validity index (I-CVI). The I-CVI was defined as the proportion of experts assigning 3–5 to a given symptom. Symptoms were prioritised using a prespecified hierarchy (mean importance rating, I-CVI, then CV),^39^ and the final glioma-specific customised PRO-CTCAE scale was assembled by combining confirmed symptoms from both rounds (Supplementary Fig. S3).

### Part 2: VERONICA cohort study

During the screening, demographic and clinical data were collected, including age, sex, pathological subtype, recurrence status, and treatment regimen. Eligible participants were scheduled to complete baseline assessment (Visit 2, V2) within 42 days after signing the informed consent. The V2 was conducted onsite, during which investigators assessed Karnofsky Performance Status (KPS), recorded clinician-reported outcome (ClinRO)-CTCAE v5·0 and guided participants, including the customised PRO-CTCAE scale and the EORTC QLQ-C30.^16^^,^^40^ For participants unable to operate electronic devices independently, assistance from family members was permitted under the investigator supervision.

On the day following V2, a remote visit (V2b) was conducted, during which participants completed the customised PRO-CTCAE scale with randomised item order to assess test-retest reliability and participants’ proficiency with the electronic PRO (ePRO) tool. After V2 and V2b, participants completed remote follow-up visits every six weeks (V3 to V6). Investigators remotely guided completion of the customised PRO-CTCAE scale, QLQ-C30, and Global Impression of Change (GIC) scales.^16^^,^^17^^,^^41^ Data on KPS and adverse events were collected via telephone calls, text messages, or video consultations.^40^ After V6, participants entered long-term follow-up every 12 ± 2 weeks, with procedures consistent with V3–V6.

Data were captured using an ePRO system and recorded in a central electronic data capture (EDC) system. Detailed data-management procedures, definitions of endpoint-specific analysis eligibility, and participant flow are provided in the Supplementary Method S4–S5 and Supplementary Fig. S4.

#### Sample size calculation

The sample size was based on the precision of prevalence estimates for patient-reported AEs. Assuming the most conservative scenario p = 0·50, a two-sided 95% confidence interval half-width of ±0·05 requires $n=Z_{0\cdot 975}^{2}\frac{p\left(1-p\right)}{d^{2}}={1\cdot 96}^{2}\times \frac{0\cdot 25}{{0\cdot 05}^{2}}\approx 384$ analysable questionnaires. Allowing for an anticipated 15·0% non-evaluable rate (i.e., ∼85·0% valid questionnaires), the target enrolment was 384/0·85 ≈ 452, rounded to 450 participants.^42^ An enrichment strategy aimed to ensure a sufficient anchor subgroup for known-groups validity (KPS <70 versus ≥70) by targeting approximately 60–70 participants with impaired performance status (KPS <70; corresponding to ∼15·0% of the cohort), consistent with recommendations for anchor-based interpretation of PRO measures.^41^^,^^43^

### Statistical analysis

Unless otherwise specified, statistical tests were two-sided and p < 0·05 was considered statistically significant.^44^ All statistical analyses were performed using R version 4.4.2.

For part 1 (scale development via Delphi survey), patient pilot testing results were summarised descriptively. Patient-reported positivity rates were summarised to characterise symptom frequency and clinical relevance. Spearman rank correlations (r_s) between each symptom score and the total score were calculated because symptom responses were ordinal and monotonic associations were of primary interest. In the two-round Delphi survey, Kendall's W was used after each round to assess inter-expert agreement.^38^ Mean importance scores were used to summarise overall perceived clinical importance, CVs to quantify dispersion in expert ratings, and I-CVIs to evaluate item-level content validity. Symptoms were prioritised using the prespecified hierarchy of mean importance score, I-CVI, and CV.^39^

For part 2 (VERONICA cohort study), all psychometric analyses were prespecified and conducted on an available-case basis without imputation; participants were included in each analysis if they had the required data for that endpoint (Supplementary Method S5; Supplementary Fig. S4). Items with responses such as “Not applicable”, “Not sexually active”, or “Prefer not to answer” were excluded from validity and responsiveness analyses.

Test-retest reliability was assessed by comparing item-level responses on the customised PRO-CTCAE scale between V2 and V2b. The intraclass correlation coefficient (ICC) was calculated using a two-way mixed-effects model with average measures (ICC [C, k]).^45^ An ICC ≥0·70 was considered acceptable.^18^

Convergent validity was assessed at V4 by examining correlations between customised PRO-CTCAE items and prespecified corresponding EORTC QLQ-C30 domains (Supplementary Table S2). Pearson correlation coefficients were calculated for each matched pair. To ensure consistent directionality (higher scores indicating greater symptom burden), QLQ-C30 global health status and functional domains were reverse-scored where appropriate, whereas symptom domains were retained as scored. Correlation strength was interpreted as follows: r < 0·10: negligible, 0·10 ≤ r < 0·30: low, 0·30 ≤ r < 0·50: moderate, r ≥ 0·50: strong.^35^^,^^46^

Known-groups validity was assessed by comparing scores between participants with different functional statuses based on KPS, to evaluate the scale's ability to distinguish between participants with different levels of performance status. Participants were stratified into KPS <70 and KPS ≥70 groups. Two-sample t-tests were used for PRO-CTCAE items with ordinal (5-level) response options, and chi-square tests were applied to dichotomous items. Cohen's d was calculated as the mean difference divided by the pooled standard deviation. Effect size interpretation (Cohen's d) of 0·20, 0·50, and 0·80 were interpreted as small, medium, and large, respectively.^46^

Responsiveness was evaluated by assessing sensitivity of item scores to changes in health status between V3 and V4, anchored to GIC score at V4. Participants were grouped into three groups: improved (slightly, moderately, or markedly improved), unchanged, and worsened (slightly, moderately, or markedly worsened). Change scores (V4–V3) were calculated, and standardised response means (SRMs) were computed. Responsiveness was classified based on SRM magnitude: SRM >0·8: high responsiveness, 0·50 <SRM ≤0·80: moderate, 0·20 < SRM ≤ 0·50: low, SRM ≤0·20: minimal responsiveness.^46^^,^^47^ Ordered differences across GIC groups were assessed using the one-sided Jonckheere–Terpstra test.^45^

We conducted subgroup analyses. Participants were categorised into six prespecified subgroups by integrated diagnosis (astrocytoma, oligodendroglioma, glioblastoma) and treatment (Stupp vs non-Stupp; standard Stupp vs Stupp plus TTFields). Reliability, validity, and responsiveness were independently assessed within each subgroup. Subsequently, four key psychometric metrics, including test-retest reliability (ICC), convergent validity (Pearson's r, transformed via Fisher's Z), known-group validity (Cohen's d, adjusted to Hedges' g), and responsiveness (SRM) were synthesised across subgroups using random-effects meta-analysis with inverse-variance weighting. Corresponding sampling variances were estimated.^48^ Based on the significance of the Q statistic (p < 0·05) and the magnitude of the I^2^ index, the level of cross-subgroup consistency was categorised as follows: I^2^ < 0·25: low heterogeneity, 0·25 ≤ I^2^ < 0·50: moderate heterogeneity, 0·50 ≤ I^2^ < 0·75: substantial heterogeneity, and I^2^ > 0.75: considerable heterogeneity.^47^^,^^49^

### Role of the funding source

The funders of the study had no role in study design, data collection, data analysis, data interpretation, or writing of the report.

## Results

### Part 1: scale development via Delphi survey

Development of the customised PRO-CTCAE scale for adult-type diffuse glioma was undertaken collaboratively by clinical experts and patients through a multi-step process, comprising initial screening, patient pilot testing, and two-round Delphi survey (Fig. 1a). From the PRO-CTCAE® item library, initial screening excluded 50 items covering 34 symptoms, including nail changes, epistaxis, muscle and joint aches, menstrual abnormalities, and so on (Supplementary Fig. S2).

In the 1st round Delphi survey, seven experts from six glioma treatment centres participated (response rate 100·0%; Table 1), with moderate agreement in symptom rankings (Kendall's W = 0·415; p < 0·001). In parallel, 30 participants completed the beta scale once in pilot testing at the lead centre (Supplementary Tables S3–S4). Integrating the mean importance rating, the patient-reported positivity rate from pilot testing, and additional expert suggestions, 21 symptoms were retained as confirmed symptoms; 23 symptoms were categorised as pending; and two newly suggested symptoms (“Bloating (FS)” and “Decreased libido (S)”) were also categorised as pending (Supplementary Fig. S2).

**Table 1:** Baseline characteristics of experts participating in the two-round Delphi survey.

NO.	Initials	City	Country	Glioma centre	Expertise	Years of experience	1st Round invited	1st Round participated	2nd Round invited	2nd Round participated
01	JW	Shanghai	China	Huashan Hospital, Fudan University	Neurosurgery: Glioma Speciality	>20 years	Y	Y	Y	Y
02	SW	Shanghai	China	Huashan Hospital, Fudan University	Neurosurgery: Glioma Speciality	>10 years	N	N	Y	Y
03	JZ	Nanjing	China	The First Affiliated Hospital of Nanjing Medical University	Neurosurgery: Glioma Speciality	>15 years	N	N	Y	Y
04	YY	Nanjing	China	The First Affiliated Hospital of Nanjing Medical University	Neurosurgery: Glioma Speciality	>30 years	N	N	Y	Y
05	LC	Guangzhou	China	Guangdong Sanjiu Brain Hospital	Oncology: Glioma Speciality	>20 years	N	N	Y	Y
06	YL	Beijing	China	Beijing Tiantan Hospital, Capital Medical University	Oncology: Glioma Speciality	>10 years	Y	Y	Y	N
07	XQ	Beijing	China	Beijing Tiantan Hospital, Capital Medical University	Radiation Oncology: Glioma Speciality	>30 years	N	N	Y	Y
08	ZT	Kunming	China	The First Affiliated Hospital of Kunming Medical University	Neurosurgery: Glioma Speciality	>15 years	N	N	Y	Y
09	CG	Guangzhou	China	Sun Yat-Sen University Cancer Center	Oncology: Glioma Speciality	>15 years	Y	Y	Y	Y
10	JX	Beijing	China	Xuanwu Hospital, Capital Medical University	Oncology: Glioma Speciality	>20 years	Y	Y	Y	Y
11	YC	Beijing	China	Xuanwu Hospital, Capital Medical University	Neurosurgery: Glioma Speciality	>10 years	Y	Y	Y	N
12	KS	Wuhan	China	Tongji Hospital, Tongji Medical College, Huazhong University of Science and Technology	Neurosurgery: Glioma Speciality	>20 years	N	N	Y	Y
13	LK	Shanghai	China	Shanghai Proton and Heavy Ion Centre, Fudan University Cancer Hospital	Radiation Oncology: Glioma Speciality	>20 years	N	N	Y	Y
14	ZF	Xi'an	China	Xijing Hospital, Fourth Military Medical University	Neurosurgery: Glioma Speciality	>30 years	N	N	Y	Y
15	SL	Xi'an	China	Xijing Hospital, Fourth Military Medical University	Neurosurgery: Glioma Speciality	>15 years	Y	Y	Y	N
16	CZ	Shanghai	China	Shanghai East Hospital, Tongji University School of Medicine	Neurosurgery: Glioma Speciality	>20 years	N	N	Y	Y
17	ZW	Fujian	China	The First Affiliated Hospital of Fujian Medical University	Neurosurgery: Glioma Speciality	>15 years	N	N	Y	Y
18	YZ	Ningxia	China	General Hospital of Ningxia Medical University	Neurosurgery: Glioma Speciality	>15 years	N	N	Y	Y
19	XS	Chongqing	China	The First Affiliated Hospital of Chongqing Medical University	Neurosurgery: Glioma Speciality	>30 years	N	N	Y	Y
20	SC	Chongqing	China	The First Affiliated Hospital of Chongqing Medical University	Neurosurgery: Glioma Speciality	>10 years	Y	Y	Y	N

This table summarises the characteristics of experts invited to either round of the Delphi survey. “1st round invited” and “2nd round invited” indicate whether an expert was invited to participate in the respective round; “1st round participated” and “2nd round participated” indicate whether the expert completed the questionnaire in that round. Years of experience refer to clinical and research experience in glioma-related practice.

**Abbreviations:** Y, Yes; N, No.

In the 2nd round Delphi survey, 16 of 20 invited experts from 14 glioma treatment centres participated (response rate 80·0%). Kendall's W again supported internal consistency of expert ratings (Kendall's W = 0·351, p < 0·001). Three of the seven experts who participated in 1st round also participated in 2nd round (Table 1). Based on prespecified decision rules and prioritisation criteria, 10 symptoms were added to the final version of the scale, all of which achieved I-CVI ≥0·88 (Supplementary Table S5).

The final version of the customised PRO-CTCAE scale comprises 53 items covering 31 symptoms, together with one open-ended free-text item (Supplementary Fig. S3, Supplementary Table S2, and Scale). The scale spans multiple symptom domains and physiological systems, enabling standardised and low-burden assessment of patient-reported symptomatic adverse events in adults with diffuse gliomas.

### Part 2: VERONICA cohort study

Between September 2022 and March 2025, 450 participants were enrolled across 13 glioma treatment centres and followed up for at least six months (Table 2, Supplementary Table S6). Overall, 57·6% were male and 42·4% were female. Mean age was 49·1 years (SD 12·8), and the average KPS at baseline (Visit 2) was 72·2 (SD 17·1). A total of 424 participants (94·2%) met the prespecified requirements for at least one psychometric analysis (Supplementary Fig. S4). Integrated diagnoses were astrocytoma (20·9%), oligodendroglioma (19·8%), and glioblastoma (58·2%), with a small remainder classified as diffuse glioma, not otherwise specified (NOS). Among participants with complete postoperative treatment data (n = 288), 250 received radiotherapy, and 249 received chemotherapy. 163 participants underwent concurrent chemoradiotherapy, 106 received the standard Stupp protocol, and 52 received Tumour-Treating Fields (TTFields) (Supplementary Fig. S5).

**Table 2:** Baseline characteristics of participants in the VERONICA study.

Variables	Total (n = 450)
Age at first visit, mean ± SD	49·1 ± 12·8
V2-KPS, mean ± SD	72·2 ± 17·1
Sex, n (%)	
Male	259 (57·6)
Female	191 (42·4)
Disease status, n (%)	
Recurrence	54 (12·0)
Primary	396 (88·0)
Extent of resection, n (%)	
GTR	307 (68·2)
Non-GTR	143 (31·8)
Integrated diagnosis (WHO CNS5), n (%)	
GBM	262 (58·2)
A	94 (20·9)
O	89 (19·8)
NOS	5 (1·1)
WHO CNS5 grade, n (%)	
2	93 (20·7)
3	58 (12·9)
4	299 (66·4)
Treatments, n (%)	Total (n = 288)
RT	250 (86·8)
CT	249 (86·5)
TTFields	52 (18·1)
Stupp	106 (36·8)
Standard stupp	71 (24·7)
TTFields	52 (18·1)
Stupp + TTFields	35 (12·2)
Effective supply analysis, n (%)	
Effective	424 (94·2)
Ineffective	26 (5·8)

Data are presented as n (%) for categorical variables and mean (SD) for continuous variables, as appropriate. Baseline was defined as Visit 2 (V2). Postoperative treatment regimens are summarised among participants with complete postoperative treatment data (n = 288).

**Abbreviations:** V2-KPS, Karnofsky Performance Status at Baseline (Visit 2); GTR, Gross Total Resection; GBM, Glioblastoma; A, Astrocytoma; O, Oligodendroglioma; NOS, Not Otherwise Specified; RT, Radiotherapy; CT, Chemotherapy; TTFields, Tumour Treating Fields.

#### Test-retest reliability

**Fig. 2:**
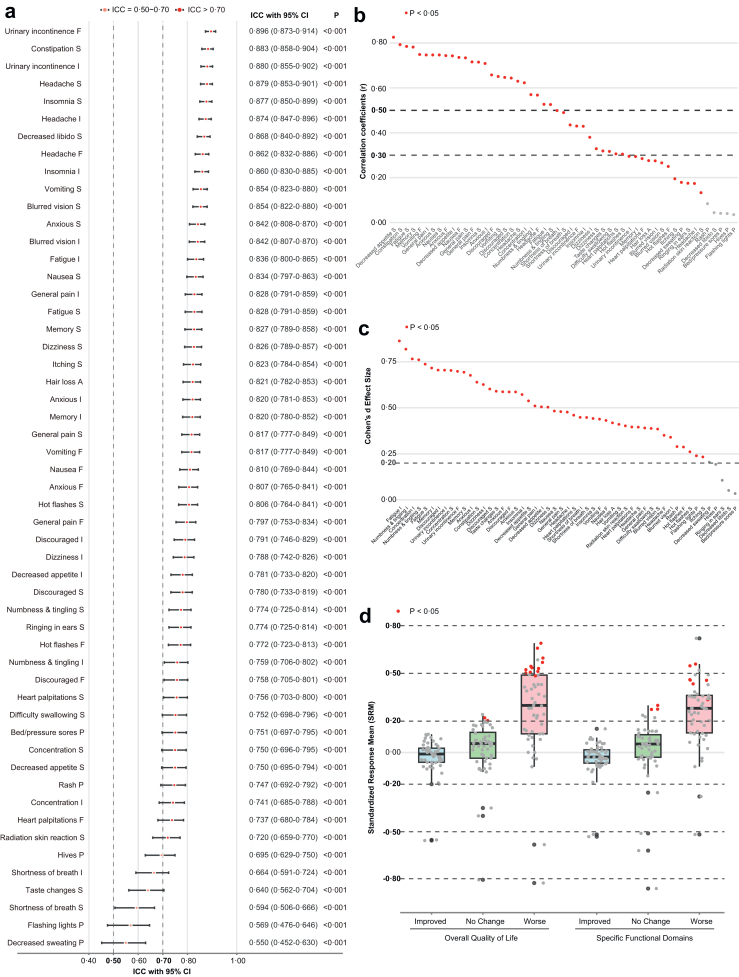
**Psychometric Validation Results. a. Test-retest reliability.** Item-level intraclass correlation coefficients (ICC) with 95% confidence intervals comparing Visit 2 and Visit 2b responses. Dashed vertical lines indicate ICC = 0·50 and ICC = 0·70; items with ICC ≥0·70 indicate acceptable reproducibility (47 of 53 items), whereas items with 0·50–0·70 indicate moderate reproducibility (6 items: Hives [P], Shortness of breath [I], Taste changes [S], Shortness of breath [S], Flashing lights [P], and Decreased sweating [P]). **b. Convergent validity.** Pearson correlation coefficients (r) between prespecified PRO-CTCAE items and corresponding EORTC QLQ-C30 domains at Visit 4. Dashed horizontal lines indicate r = 0·30 and r = 0·50 correlations, correlation strength was interpreted as follows: r < 0·10: negligible, 0·10 ≤ r < 0·30: low, 0·30 ≤ r < 0·50: moderate, r ≥ 0·50: strong; red points denote p < 0·05. **c. Known-groups validity.** Cohen's d effect sizes comparing PRO-CTCAE item scores between participants with KPS <70 versus ≥70 at Visit 4. The dashed horizontal line indicates d = 0·20 (small effect); red points denote p < 0·05. **d. Responsiveness.** Standardised response means (SRM) for change from Visit 3 to Visit 4, stratified by GIC categories (improved, no change, worse) for overall quality of life and domain-specific functional anchors. Dashed horizontal lines indicate SRM = 0·20, 0·50, and 0·80, SRM >0·8: high responsiveness, 0·50 < SRM ≤ 0·80: moderate, 0·20 < SRM ≤ 0·50: low, SRM ≤0·20: minimal responsiveness; red points denote p < 0·05 for ordered differences across GIC categories. **Abbreviations:** ICC, intraclass correlation coefficient; CI, Confidence Interval; r, Pearson correlation coefficient; SRM, standardised response mean; KPS, Karnofsky Performance Status; GIC, Global Impression of Change; F, frequency; S, severity; I, interference with daily activities; P, presence/absence; A, amount.

The customised PRO-CTCAE scale demonstrated good test-retest reliability (Fig. 2a, Supplementary Fig. S2a and Supplementary Table S7). Of 53 items, 47 items demonstrated ICC ≥0·70. 6 items showed ICC values between 0·50 and 0·70, namely “Hives (P)”, “Shortness of breath (I)”, “Taste changes (S)”, “Shortness of breath (S)”, “Flashing lights (P)”, and “Decreased sweating (P)”, indicating moderate reproducibility for these items.

#### Convergent validity

Convergent validity was evaluated at V4 by correlating PRO-CTCAE items with prespecified corresponding EORTC QLQ-C30 domains (Fig. 2b, Supplementary Fig. S2b, Supplementary Fig. S6, Supplementary Fig. S7, and Supplementary Table S8). For symptom/function domain pairings, 25 items demonstrated strong correlations (r ≥ 0·50), 11 showed moderate correlations (0·30 ≤ r < 0·50), and 12 had low correlations (0·10 ≤ r < 0·30). Only 5 items exhibited weak correlations (r < 0·10), including “Flashing lights (P) versus physical functioning”, “Rash/measles (P) versus physical functioning”, “Pressure sores (P) versus physical functioning”, “Decreased libido (S) versus emotional functioning”, and “Skin rash (P) versus physical functioning”. For correlations with QLQ-C30 Global Health Status/QoL domain, 30 items showed moderate correlations (0·30 ≤ r < 0·50), 17 had low correlations (0·10 ≤ r < 0·30), and 6 showed weak associations (r < 0·10). All correlations were generally statistically significant (p < 0·05).

#### Known-groups validity

Known-groups validity was assessed by comparing participants with KPS <70 and ≥70 at V4 (Fig. 2c, Supplementary Fig. S2c and Supplementary Table S9). Among the 53 items, 49 exhibited Cohen's d ≥ 0·20, indicating meaningful discriminatory ability. Of these 49 items, 43 demonstrated statistically significant between-group differences (p < 0·05), supporting the reliability of their known-groups validity. 4 items (“Bed/pressure sores (P)”, “Decreased libido (S)”, “Ringing in ears (S)”, and “Hives (P)”) showed Cohen's d < 0·20 and non-significant differences, suggesting limited sensitivity and discriminatory ability to KPS-based group differences for these items.

#### Responsiveness

Responsiveness was evaluated from V3 to V4, anchored to GIC at V4 (Fig. 2d, Supplementary Fig. S2d; Supplementary Table S10). In the global responsiveness analysis based on the GIC item for “overall quality of life”, 37 items demonstrated small to moderate responsiveness (SRM ≥0·20) in the “worsened” group. 12 of these had SRMs ranging from 0·49 to 0·69 and reached statistical significance (p < 0·05). In contrast, no items in the “improved” group reached both SRM >0·20 and statistical significance, and only a few items in the “unchanged” group showed low SRM values (Supplementary Fig. S2d and Supplementary Table S10). Domain-specific responsiveness analyses based on corresponding GIC dimensions yielded consistent findings: 37 items in the “worsened” group showed moderate or higher responsiveness (SRM ≥0·30), with many reaching statistical significance.

### Subgroup analysis

Across six prespecified subgroups defined by integrated diagnosis and treatment regimen, reliability, validity, and responsiveness were broadly consistent, with generally low heterogeneity for key psychometric metrics and no statistically significant differences (Supplementary Fig. S8; Supplementary Table S11). These findings confirm the scale's strong cross-group stability and comparability among patients with different adult-type diffuse glioma subtypes and treatment modalities.

## Discussion

Based on the linguistically validated Simplified Chinese PRO-CTCAE® item library as the item pool, we developed and psychometrically validated a customised PRO-CTCAE scale for adult-type diffuse gliomas through a structured, stakeholder-informed pipeline. To our knowledge, this is among the first efforts to generate a glioma-tailored PRO-CTCAE scale and to evaluate its reliability, validity, and responsiveness within a prospective, multicentre, observational cohort.^18^^,^^50^ Overall, the scale demonstrated satisfactory performance across prespecified psychometric domains and was feasible for longitudinal administration within a remote ePRO workflow.

**Fig. 3:**
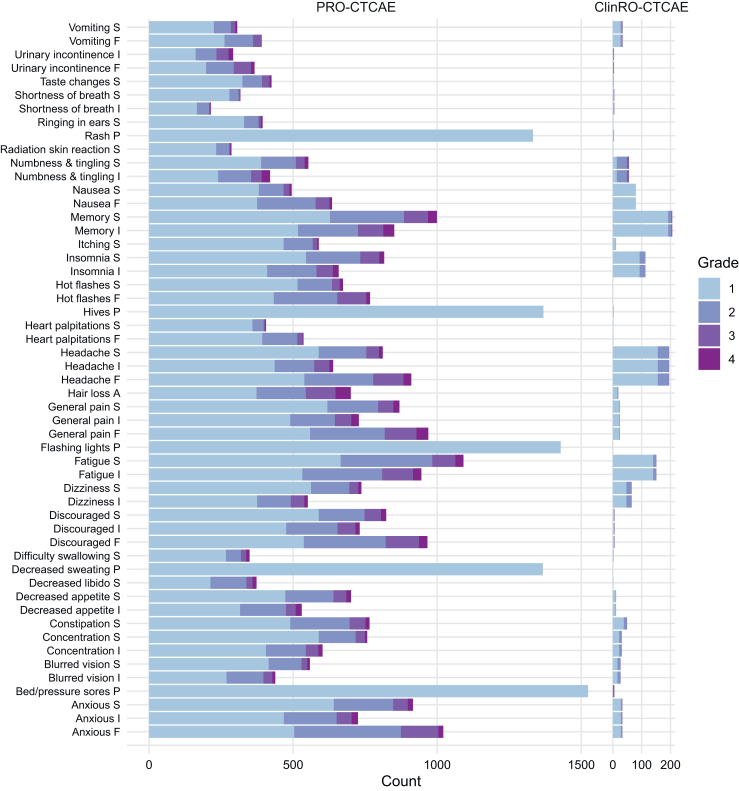
**Comparison of PRO-CTCAE and ClinRO-CTCAE in Adverse Event Reporting.** Stacked bar charts comparing the distribution of non-zero grades (1–4) for symptomatic adverse events captured by the customised PRO-CTCAE (left) and the corresponding clinician-reported CTCAE v5·0 symptomatic adverse events (ClinRO-CTCAE; right). Each row represents a PRO-CTCAE item and colours indicate grade (1–4); the x-axis shows the number of reports. The figure illustrates the greater capture of symptomatic AE burden by patient report relative to clinician grading across the customised scale. **Abbreviations:** PRO-CTCAE, Patient-Reported Outcomes Version of the Common Terminology Criteria for Adverse Events; ClinRO-CTCAE, Clinician-Reported Common Terminology Criteria for Adverse Events; F, Frequency; I, Interference; S, Severity; P, Presence/Absence; A, Amount.

A key contribution of this work is the demonstration that a symptom-targeted customised PRO-CTCAE scale can retain strong measurement properties while reducing burden relative to the full PRO-CTCAE® library. Test-retest reliability was robust overall (median ICC 0·81), supporting stability of item responses under short-interval reassessment with randomised item order.^18^ Convergent validity, anchored to the EORTC QLQ-C30, showed stronger correlations with symptom- and function-specific domains than with global health status, consistent with the conceptual distinction between AE-focused symptom reporting and broader HRQoL constructs.^16^ Known-groups validity further supported clinical discriminative ability by distinguishing participants with impaired versus preserved performance status (KPS <70 versus ≥70). Responsiveness analyses indicated sensitivity to deterioration anchored by GIC, with patterns that were clinically plausible for neurologically and systemically mediated symptoms (e.g., fatigue, sleep disturbance, anxiety, and cognitive difficulties). These results, together with the observed higher sensitivity of PRO relative to clinician-reported grading in capturing symptomatic AEs (Fig. 3), reinforce the added value of systematic patient-report for tolerability assessment and symptom surveillance in neuro-oncology.^4^^,^^51^ Importantly, retention of the PRO-CTCAE free-text item provides a complementary channel for capturing individualised or intermittent toxicities not fully represented by structured items, and offers a practical mechanism for iterative refinement of the scale as treatment paradigms evolve.

Our findings extend the broader evidence supporting PRO-CTCAE as a psychometrically robust framework across diverse disease settings and languages.^17^, ^18^, ^19^, ^20^ At the same time, they address a practical limitation increasingly recognised in PRO implementation: routine administration of the full 124-item PRO-CTCAE® library can be burdensome and may compromise feasibility, especially in populations with neurologic symptoms, fluctuating function, and prolonged treatment trajectories.^17^^,^^21^ Tumour-specific PRO-CTCAE scales have therefore been developed in several oncology settings, primarily to optimise feasibility while maintaining sensitivity to clinically meaningful symptomatic toxicities, most commonly via structured consensus methods such as modified Delphi procedures and, in some cases, multi-method symptom selection or cross-sectional profiling.^17^^,^^22^, ^23^, ^24^, ^25^, ^26^, ^27^, ^28^, ^29^ In that context, our study adds two methodological elements that strengthen tumour-tailoring for adult-type diffuse gliomas: (1) incorporation of patient pilot testing to inform prioritisation alongside expert judgement, and (2) prespecified quantitative decision rules (mean importance rating, I-CVI, then CV) applied to a defined “pending” pool, improving transparency and reproducibility of item selection.^39^ Together, these design choices reduce the risk that item selection reflects only clinician preferences or pan-tumour symptom prevalence patterns, which may not capture glioma-specific symptomatology and treatment-related toxicities.

A notable feature of our development process is that additional experts were recruited for the second Delphi round. This approach broadens centre coverage and stakeholder representation, which is advantageous for generalisability of a multicentre scale, but it can introduce between-round variation in ratings because the voting panel is not identical across rounds. We therefore considered the potential for discontinuity as a methodological limitation and implemented safeguards to mitigate its impact. Specifically, experts received anonymised, group-level summaries of first-round findings and qualitative comments to inform re-rating, without disclosure of individual-level voting; we applied prespecified prioritisation criteria to standardise selection decisions; and we quantified agreement using Kendall's W in each round.^37^^,^^38^ While some between-round variation is possible, these measures support the robustness of the final scale.

Beyond scale development, our study provides evidence supporting feasibility of longitudinal ePRO administration in a geographically distributed neuro-oncology cohort. The high proportion of participants contributing data eligible for at least one prespecified psychometric analysis (94·2%) suggests that remote follow-up with centralised EDC can yield analytically useful data at scale. This is particularly relevant in adult-type diffuse gliomas, where symptom burden is dynamic and where frequent onsite assessments may be impractical. The structured mapping of items to external anchors (QLQ-C30, KPS, GIC, and ClinRO-CTCAE) further enhances interpretability and facilitates triangulation across patient-reported, clinician-reported, and functional measures, which is aligned with emerging expectations for PRO integration in oncology trials and routine care.^9^^,^^15^

This study has limitations. First, although multicentre design enhances generalisability, all participating centres were located in China, which may limit cross-cultural applicability. Second, reliance on electronic devices for PRO data collection required a baseline level of digital literacy and cognitive ability, thereby excluding certain patients with neurologic deficits such as aphasia, agnosia, or hemiplegia. While participants received brief training and clarification at enrolment, some remained unable to complete self-report assessments. Third, as an observational study, causal inferences regarding symptom trajectories or treatment effects cannot be established. Fourth, proxy reporting was strictly prohibited in patient-reported outcomes,^15^ which may have further reduced inclusivity for patients with advanced cognitive or functional impairment. Finally, although the customised PRO-CTCAE scale is effective for capturing symptomatic AEs, it does not fully capture all FDA-recommended PRO domains,^11^^,^^52^ and therefore may be complemented by additional PROMs when broader assessment is required. Future work should evaluate responsiveness to improvement, establish thresholds for meaningful within-person change, and explore scoring approaches for summarising symptom burden while preserving item-level interpretability.

In summary, we developed and validated a customised PRO-CTCAE scale for adult-type diffuse gliomas, with rigorous evaluation against multiple anchors (KPS, EORTC QLQ-C30, GIC, and ClinRO-CTCAE) and within a longitudinal multicentre ePRO workflow, demonstrating strong reliability, validity, and responsiveness. Given the distinct neurological symptomatology, treatment trajectories, and the known limitations of clinician-reported toxicity grading in capturing patient experience, this glioma-specific instrument enables standardised, low-burden quantification of symptomatic AEs and supports broader implementation of patient-centred AE monitoring in both clinical trials and routine neuro-oncology practice.

## Contributors

JW, YY, SW, and SS had full access to all the data in the study and take responsibility for the integrity of the data and the accuracy of the data analysis. SS, XW, XC, CL, KN, CG, JX, MN, CXL, QH, SL, HCW, HXW, ML, YW, CS, and ZX contributed equally to this work. JW, JZ, LBC, ZT, YM, JT, XQ, KS, YY, and SW contributed equally in a senior capacity and are co-senior authors. Study concept and design: JW, JZ, LBC, ZT, YM, JT, XQ, KS, YY, SW, ZW, SS, XW, XC, CL, KN, CG, JX, MN, CXL, QH, SL, HCW, HXW, ML, CS, ZX. Acquisition of data: SS, XW, XC, CL, KN, CG, JX, MN, CXL, QH, SL, HCW, HXW, ML, YW, CS, ZX, CN, WZ, ZW, YS, SC, YZ, XS, HM, JZ, CZ, JC, ZF, LK, JS, YC, LZ. Analysis and interpretation of data: JW, YPY, ZW, SW, SS, JZ, XW, XC, CL, KN, CG, JX, MN, CXL, QH, SL, HCW, HXW, ML, YW, CS, ZX, CN, WZ, ZW, SC, YS, YZ, XS, HM, JZ, CZ, JC, ZF, LK. Drafting of the manuscript: SW, JW, YY, SS. Critical revision of the manuscript for important intellectual content: JW, YPY, JZ, LBC, ZT, YM, JT, XQ, KS, YY, SW, SS, XW, XC, CL, KN, CG, JX, MN, CXL, QH, SL, HCW, HXW, ML, YW, CS, ZX, CN, WZ, ZW, SC, YS, YZ, XC, HM, JZ, CZ, JC, ZF, LK, JS, YC, LZ. Statistical analysis: YY, SW, SS, ZW. Obtained funding: JW. Administrative, technical, or material support: JW, YPY, JZ, LBC, ZT, YM, JT, XQ, KS, YY, SW. Study supervision: JW, YPY, JZ, LBC, ZT, YM, JT, XQ, KS, YY, SW. Correspondence during peer review was primarily coordinated through Prof. Jinsong Wu, MD, PhD, as the primary corresponding author. This administrative arrangement does not detract from the important scientific contributions and senior oversight provided by the participating centre leads within the NGMA, all of whom contributed as described above and approved the final manuscript.

## Data sharing statement

De-identified participant data and a data dictionary will be shared with qualified academic researchers whose proposed use has been reviewed and approved, contingent upon submission of a formal proposal and signing of a data access agreement. Data sharing will be conducted in accordance with institutional ethics approval and applicable regulations. Requests should be directed to Prof. Jinsong Wu (wjsongc@126.com).

## Declaration of interests

The authors declare no competing interests.

## Appendix A Supplementary data

Supplementary Materials

Translated Abstract

Veronica Protocol

STROBE reporting
