# Supplementary material for: Development and validation of a customised PRO-CTCAE scale for adult-type diffuse gliomas (VERONICA): a multicentre, prospective, observational cohort study in China
Source: eClinicalMedicine. 2026 Apr 10;94:103879. doi: 10.1016/j.eclinm.2026.103879 (PMC13091834; doi:10.1016/j.eclinm.2026.103879)
Supplement: Supplementary Materials [file mmc1.pdf]

Method S1. Patient Pilot Testing.

Patient pilot testing was conducted at Huashan Hospital (lead centre) to assess feasibility and inform expert consensus. Eligible adults with diffuse glioma attending outpatient clinics were consecutively screened and completed the beta scale once, consistent with recommendations for establishing content relevance in PRO instrument development.[1] We calculated the spearman rank correlations (r_s) between each symptom score and the total score and patient-reported positivity rate for each symptom , defined as the proportion reporting at least mild symptom burden (i.e., any response above “none/not at all”, where applicable).[2,3]

For correlation-based screening only, PRO-CTCAE responses were prespecified to be analytically recoded into standardised numeric scores to enable symptom-total association analyses across different PRO-CTCAE item structures, without any modification to item wording, response options, scoring format, or administration.[4] Symptoms were grouped by PRO-CTCAE item structure as follows: S5, single item with five response options; T2, two-item symptoms; T3, three-item symptoms; and SD, single dichotomous (presence/absence) item. To allow comparability across structures, the maximum possible symptom score was standardised to 6. The analytical recoding schemes were: S5 (0, 1·5, 3, 4·5, 6); T2 (each item: 0, 0·75, 1·5, 2·25, 3; summed across two items); T3 (each item: 0, 0·5, 1, 1·5, 2; summed across three items); and SD (No=0, Yes=6). This analytical recoding was used solely for correlation/ranking purposes and did not modify the original PRO-CTCAE item wording, response options, scoring format, or administration.

We then computed Spearman rank correlations (r_s) between each symptom score and the total score (sum of all symptom scores). An r_s ≥0·30 was prespecified as indicating an acceptable association for screening purposes.[2,3]

Method S2. Delphi Survey Procedures and Item Selection Rules.

Experts were recruited via the National Glioma Multidisciplinary Team (MDT) Alliance (NGMA) and invited by email. Eligibility criteria required 1) were currently practising in a tertiary/referral centre with an established glioma programme; 2) had ≥10 years of clinical and/or research experience in glioma management; 3) had experience with clinical trials and/or routine clinical outcome assessment in neuro-oncology; and 4) were willing to participate within the specified timeline.

Participation was voluntary, and ratings were anonymised. After each round, we calculated summary statistics and Kendall's coefficient of concordance (Kendall's W) to quantify agreement in symptom ranking across experts, where higher values indicate greater consistency of rankings.[5–7] To support independence and reduce social desirability bias, ratings were only group-level summaries were circulated between rounds.

In the 1st round Delphi survey, experts were not informed of the pilot testing results when providing their initial ratings, consistent with recommendations to preserve independent judgement.[5,6] Using a five-point Likert scale, experts anonymously rated each symptom in the beta scale for clinical importance in adults with diffuse gliomas. We calculated the mean importance rating for each symptom.[2] Using prespecified thresholds, symptoms with mean importance rating >3 and symptom–total r_s ≥0·30 were provisionally classified as confirmed; all others were classified as pending. The 1st round Delphi results, pilot-testing metrics, and the full PRO-CTCAE® item library (including items excluded during pre-screening) were shared with experts for comments and proposed refinements. Symptoms deemed pending were carried forward to 2nd round.

In the 2nd round Delphi survey, experts re-rated all pending symptoms using the same five-point Likert scale. We calculated the mean importance score and coefficient of variation (CV) for each symptom and the item-level content validity index (I-CVI), defined a priori as the proportion of experts rating a symptom at or above the prespecified threshold. Symptoms were prioritised using a prespecified hierarchy (mean importance rating, I-CVI, then CV).[8] Confirmed symptoms were selected sequentially based on this ranking, and the final customised PRO-CTCAE scale was assembled by combining confirmed symptoms from both rounds (Supplementary Fig. S3).

Method S3. Eligibility, Exclusion, and Withdrawal Criteria.

Participants were eligible if they met all inclusion criteria and no exclusion criteria.

Inclusion Criteria: 1) age 18-85 years; 2) integrated diagnosis of adult-type diffuse glioma as defined by the 2021 WHO CNS5 classification (the fifth edition of the WHO Classification of Tumours of the Central Nervous System, published in 2021), included patients with astrocytoma (*IDH*-mutant), oligodendroglioma (*IDH*-mutant with 1p/19q codeletion), and glioblastoma (*IDH*-wildtype); 3) for newly diagnosed disease, no non-surgical anticancer therapy initiated; 4) for recurrent disease, recurrence confirmed within 42 days according to Response Assessment in Neuro-Oncology (RANO) criteria; 5) currently receiving anti-tumour therapy with planned continuation within the next 28 days; 6) written informed consent provided; and 7) additional requirements: no significant cognitive impairment as judged by investigators; ability to use a mobile phone or computer independently or with assistance; literacy in Chinese; and minimum education level of primary school.

Exclusion Criteria: 1) deemed unsuitable for participation by investigators; 2) receipt of non-surgical anticancer treatment since diagnosis; or 3) failure to complete the questionnaire within 42 days after signing informed consent.

Withdrawal Criteria: 1) investigator-initiated withdrawal for major protocol violations, critical deviations, or termination required by regulatory authorities or ethics committees, or if the study was deemed scientifically invalid or infeasible by investigators; or 2) participant-initiated withdrawal at any time, investigator judgement that continued participation was not in the participant's best interest, or death during the study.

Method S4. Data Collection and Management.

To ensure standardised, secure, and traceable data capture across centres, VERONICA adopted an integrated electronic workflow comprising an electronic patient-reported outcome (ePRO) system for questionnaire administration and a central electronic data capture (EDC) system for clinical data management.[9,10]

To standardise administration, investigators guided participants to complete onsite assessments at V2 and remote assessments thereafter, using telephone calls, text messages, or video consultations when needed. For participants unable to operate electronic devices independently, assistance from family members was permitted under investigator supervision, without proxy answering. Investigators provided brief training at enrolment regarding ePRO navigation and item interpretation, and participants were encouraged to complete questionnaires based on their own experience during the prespecified recall period.

At database lock, the final analytic dataset was extracted from the EDC system. Identifiers were removed or replaced with study-specific codes before analysis, and only authorised study personnel had access to the final datasets.

Method S5. Data Completeness and Analysis Eligibility.

To support analytic rigour and transparent reporting of sample denominators across psychometric endpoints, we prespecified a hierarchical framework distinguishing data eligibility (participant-level validity of records) from analysis eligibility (endpoint-specific availability of required assessments). This framework was used to define analysis sets, generate participant flow summaries (Supplementary Fig. S4), and determine inclusion for each psychometric analysis without post hoc modification.

To ensure data quality and analytic rigour, we predefined criteria for data eligibility and analysis eligibility. Data were considered eligible if participants met all inclusion criteria and no exclusion criteria, and if EDC entries were complete without missing values. Participants who were later found to violate eligibility criteria, withdrew before any usable assessment, or had critical protocol deviations affecting interpretability could be deemed ineligible for endpoint analyses, consistent with prespecified withdrawal criteria.

Analysis eligibility was defined by the availability of the minimum dataset required for each psychometric endpoint, assessed at the visit level. Participants were included in an endpoint analysis if—and only if—they provided the required measurements for that endpoint within the prespecified visit windows. Specifically, test–retest reliability required completion of the customised PRO-CTCAE scale at V2, the randomised-order customised PRO-CTCAE scale at V2b, and KPS at V2; convergent validity required the customised PRO-CTCAE scale and QLQ-C30 at V4; known-groups validity required the customised PRO-CTCAE scale and KPS at V4; responsiveness required the customised PRO-CTCAE scale at both V3 and V4, along with GIC at V4. Participants not meeting the data requirements for a given endpoint were not included in that specific analysis.

Completeness checks embedded in the PRO measurement system minimised item-level missingness within individual scale submissions; however, missing data could still occur at the visit level (e.g., missed assessments). No imputation was performed, and analyses were conducted on an available-case basis. Overall participant flow is summarised in Supplementary Fig. S4.

Supplementary Methods References

1. Patrick DL, Burke LB, Gwaltney CJ, et al. Content validity--establishing and reporting the evidence in newly developed patient-reported outcomes (PRO) instruments for medical product evaluation: ISPOR PRO good research practices task force report: part 1--eliciting concepts for a new PRO instrument. Value Health. 2011;14(8):967-77.

2. Ferketich S. Focus on psychometrics. Aspects of item analysis. Res Nurs Health. 1991;14(2):165-8.

3. Akoglu H. User's guide to correlation coefficients. Turk J Emerg Med. 2018;18(3):91-3.

4. Basch E, Reeve BB, Mitchell SA, et al. Development of the National Cancer Institute's patient-reported outcomes version of the common terminology criteria for adverse events (PRO-CTCAE). J Natl Cancer Inst. 2014;106(9):dju244.

5. Hasson F, Keeney S, McKenna H. Research guidelines for the Delphi survey technique. J Adv Nurs. 2000;32(4):1008-15.

6. Jones J, Hunter D. Consensus methods for medical and health services research. BMJ. 1995;311(7001):376-80.

7. Legendre P. Species associations: the Kendall coefficient of concordance revisited. J Agric Biol Environ Stat. 2005;10(2):226-45.

8. Lynn MR. Determination and quantification of content validity. Nurs Res. 1986;35(6):382-5.

9. Coons SJ, Gwaltney CJ, Hays RD, et al. Recommendations on Evidence Needed to Support Measurement Equivalence between Electronic and Paper-Based Patient-Reported Outcome (PRO) Measures: ISPOR ePRO Good Research Practices Task Force Report. Value Health. 2009;12(4):419-29.

10. Bennett AV, Dueck AC, Mitchell SA, et al. Mode equivalence and acceptability of tablet computer-, interactive voice response system-, and paper-based administration of the U.S. National Cancer Institute's Patient-Reported Outcomes version of the Common Terminology Criteria for Adverse Events (PRO-CTCAE). Health Qual Life Outcomes. 2016;14:24.

Patient-Reported Outcomes version Of The Common Terminology Criteria For Adverse Events (PRO-CTCAE®)

QUICK GUIDE TO THE ITEM LIBRARY [a]

National Cancer Institute

Oral	
Dry mouth	S
Difficulty swallowing	S
Mouth/throat sores	SI
Cracking at the corners of the mouth (cheilosis/cheilitis)	S
Voice quality changes	P
Hoarseness	S

Gastrointestinal	
Taste changes	S
Decreased appetite	SI
Nausea	FS
Vomiting	FS
Heartburn	FS
Gas	P
Bloating	FS
Hiccups	FS
Constipation	S
Diarrhea	F
Abdominal pain	FSI
Fecal incontinence	FI

Respiratory	
Shortness of breath	SI
Cough	SI
Wheezing	S

Cardio/Circulatory	
Swelling	FSI
Heart palpitations	FS

Cutaneous	
Rash	P
Skin dryness	S
Acne	S
Hair loss	A
Itching	S
Hives	P
Hand-foot syndrome	S
Nail loss	P
Nail ridging	P
Nail discoloration	P
Sensitivity to sunlight	P
Bed/pressure sores	P
Radiation skin reaction	S
Skin darkening	P
Stretch marks	P

Neurological	
Numbness & tingling	SI
Dizziness	SI

Visual/Perceptual	
Blurred vision	SI
Flashing lights	P
Visual floaters	P
Watery eyes	SI
Ringing in ears	S

Attention/Memory	
Concentration	SI
Memory	SI

Pain	
General pain	FSI
Headache	FSI
Muscle pain	FSI
Joint pain	FSI

Sleep/Wake	
Insomnia	SI
Fatigue	SI

Mood	
Anxious	FSI
Discouraged	FSI
Sad	FSI

Genitourinary	
Irregular periods/vaginal bleeding	P
Missed expected menstrual period	P
Vaginal discharge	A
Vaginal dryness	S
Painful urination	S
Urinary urgency	FI
Urinary frequency	FI
Change in usual urine color	P
Urinary incontinence	FI

Sexual	
Achieve and maintain erection	S
Ejaculation	F
Decreased libido	S
Delayed orgasm	P
Unable to have orgasm	P
Pain w/sexual intercourse	S

Miscellaneous	
Breast swelling and tenderness	S
Bruising	P
Chills	FS
Increased sweating	FS
Decreased sweating	P
Hot flashes	FS
Nosebleed	FS
Pain and swelling at injection site	P
Body odor	S

Attributes	
F: Frequency	I: Interference
S: Severity	P: Presence/Absence
A: Amount	

Figure S1. Original PRO-CTCAE Scale Map.

[a] Complete library of items available at: https://healthcaredelivery.cancer.gov/pro-ctcae

Beta Customized PRO-CTCAE Scale

for Adult-type Diffuse Glioma Patients

Oral	
Dry mouth	S
Difficulty swallowing	S
Mouth/throat sores	SI
Cracking at the corners of the mouth (cheilosis/cheilitis)	S
Voice quality changes	P
Hoarseness	S

Gastrointestinal	
Taste changes	S
Decreased appetite	SI
Nausea	FS
Vomiting	FS
Heartburn	FS
Gas	P
Bloating [a]	FS
Hiccups	FS
Constipation	S
Diarrhea	F
Abdominal pain	FSI
Fecal incontinence	FI

Respiratory	
Shortness of breath	SI
Cough	SI
Wheezing	S

Cardio/Circulatory	
Swelling	FSI
Heart palpitations	FS

Cutaneous	
Rash	P
Skin dryness	S
Acne	S
Hair loss	A
Itching	S
Hives	P
Hand-foot syndrome	S
Nail loss	P
Nail ridging	P
Nail discoloration	P
Sensitivity to sunlight	P
Bed/pressure sores	P
Radiation skin reaction	S
Skin darkening	P
Stretch marks	P

Neurological	
Numbness & tingling	SI
Dizziness	SI

Visual/Perceptual	
Blurred vision	SI
Flashing lights	P
Visual floaters	P
Watery eyes	SI
Ringing in ears	S

Attention/Memory	
Concentration	SI
Memory	SI

Pain	
General pain	FSI
Headache	FSI
Muscle pain	FSI
Joint pain	FSI

Sleep/Wake	
Insomnia	SI
Fatigue	SI

Mood	
Anxious	FSI
Discouraged	FSI
Sad	FSI

Genitourinary	
Irregular periods/vaginal bleeding	P
Missed expected menstrual period	P
Vaginal discharge	A
Vaginal dryness	S
Painful urination	S
Urinary urgency	FI
Urinary frequency	FI
Change in usual urine color	P
Urinary incontinence	FI

Sexual	
Achieve and maintain erection	S
Ejaculation	F
Decreased libido [a]	S
Delayed orgasm	P
Unable to have orgasm	P
Pain w/sexual intercourse	S

Miscellaneous	
Breast swelling and tenderness	S
Bruising	P
Chills	FS
Increased sweating	FS
Decreased sweating	P
Hot flashes	FS
Nosebleed	FS
Pain and swelling at injection site	P
Body odor	S

Confirmed Items

Pending Items

Attributes	
F: Frequency	I: Interference
S: Severity	P: Presence/Absence
A: Amount	

Complete library of items available at: https://healthcaredelivery.cancer.gov/pro-ctcae

Figure S2. Beta Customized PRO-CTCAE Scale Map.

[a] These 3 items (2 symptoms) were not included in the Beta scale, but were proposed by experts during the 1st Round Delphi survey to be included as pending items for evaluation in the 2nd Round Delphi survey.

Final Customized PRO-CTCAE Scale

for Adult-type Diffuse Glioma Patients

Oral

Item	Attribute	Confirmed
Dry mouth	S	
Difficulty swallowing	S	✓
Mouth/throat sores	SI	
Cracking at the corners of the mouth (cheilosis/cheilitis)	S	
Voice quality changes	P	
Hoarseness	S	

Gastrointestinal

Item	Attribute	Confirmed
Taste changes	S	✓
Decreased appetite	SI	✓
Nausea	FS	✓
Vomiting	FS	✓
Heartburn	FS	
Gas	P	
Bloating	FS	
Hiccups	FS	
Constipation	S	✓
Diarrhea	F	
Abdominal pain	FSI	
Fecal incontinence	FI	

Respiratory

Item	Attribute	Confirmed
Shortness of breath	SI	✓
Cough	SI	
Wheezing	S	

Cardio/Circulatory

Item	Attribute	Confirmed
Swelling	FSI	
Heart palpitations	FS	✓

Cutaneous

Item	Attribute	Confirmed
Rash	P	✓
Skin dryness	S	
Acne	S	
Hair loss	A	✓
Itching	S	✓
Hives	P	✓
Hand-foot syndrome	S	
Nail loss	P	
Nail ridging	P	
Nail discoloration	P	
Sensitivity to sunlight	P	
Bed/pressure sores	P	✓
Radiation skin reaction	S	✓
Skin darkening	P	
Stretch marks	P	

Neurological

Item	Attribute	Confirmed
Numbness & tingling	SI	✓
Dizziness	SI	✓

Visual/Perceptual

Item	Attribute	Confirmed
Blurred vision	SI	✓
Flashing lights	P	✓
Visual floaters	P	
Watery eyes	SI	
Ringing in ears	S	✓

Attention/Memory

Item	Attribute	Confirmed
Concentration	SI	✓
Memory	SI	✓

Pain

Item	Attribute	Confirmed
General pain	FSI	✓
Headache	FSI	✓
Muscle pain	FSI	
Joint pain	FSI	

Sleep/Wake

Item	Attribute	Confirmed
Insomnia	SI	✓
Fatigue	SI	✓

Mood

Item	Attribute	Confirmed
Anxious	FSI	✓
Discouraged	FSI	✓
Sad	FSI	

Genitourinary

Item	Attribute	Confirmed
Irregular periods/vaginal bleeding	P	
Missed expected menstrual period	P	
Vaginal discharge	A	
Vaginal dryness	S	
Painful urination	S	
Urinary urgency	FI	
Urinary frequency	FI	
Change in usual urine color	P	
Urinary incontinence	FI	✓

Sexual

Item	Attribute	Confirmed
Achieve and maintain erection	S	
Ejaculation	F	
Decreased libido	S	✓
Delayed orgasm	P	
Unable to have orgasm	P	
Pain w/sexual intercourse	S	

Miscellaneous

Item	Attribute	Confirmed
Breast swelling and tenderness	S	
Bruising	P	
Chills	FS	
Increased sweating	FS	
Decreased sweating	P	✓
Hot flashes	FS	✓
Nosebleed	FS	
Pain and swelling at injection site	P	
Body odor	S	

Confirmed Items

Attributes

F: Frequency	I: Interference
S: Severity	P: Presence/Absence
A: Amount	

Complete library of items available at: https://healthcaredelivery.cancer.gov/pro-ctcae

Figure S3. Final Customized PRO-CTCAE Scale Map.

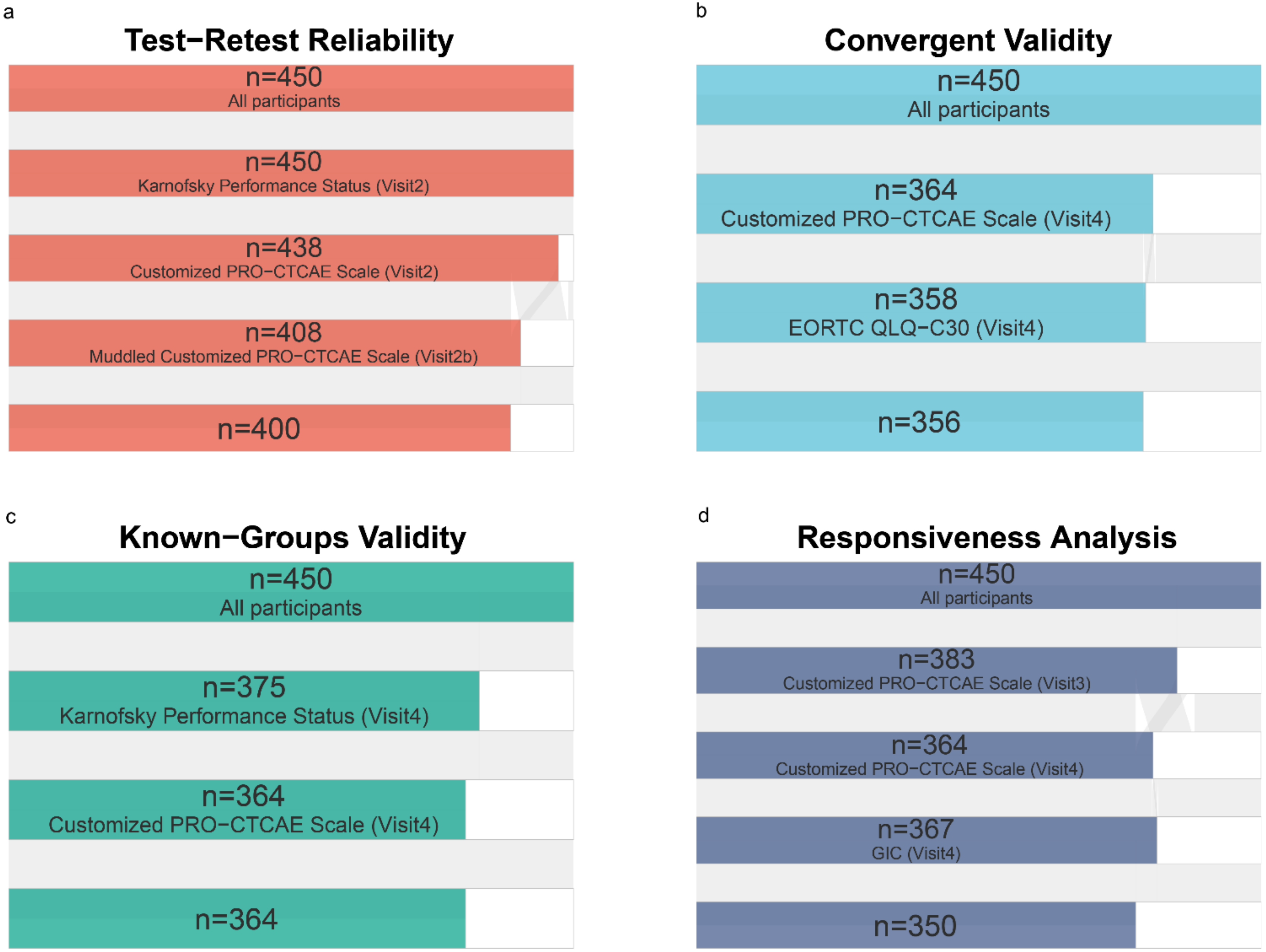

Figure S4. Eligibility of Participants for Reliability, Validity, and Responsiveness Analyses.

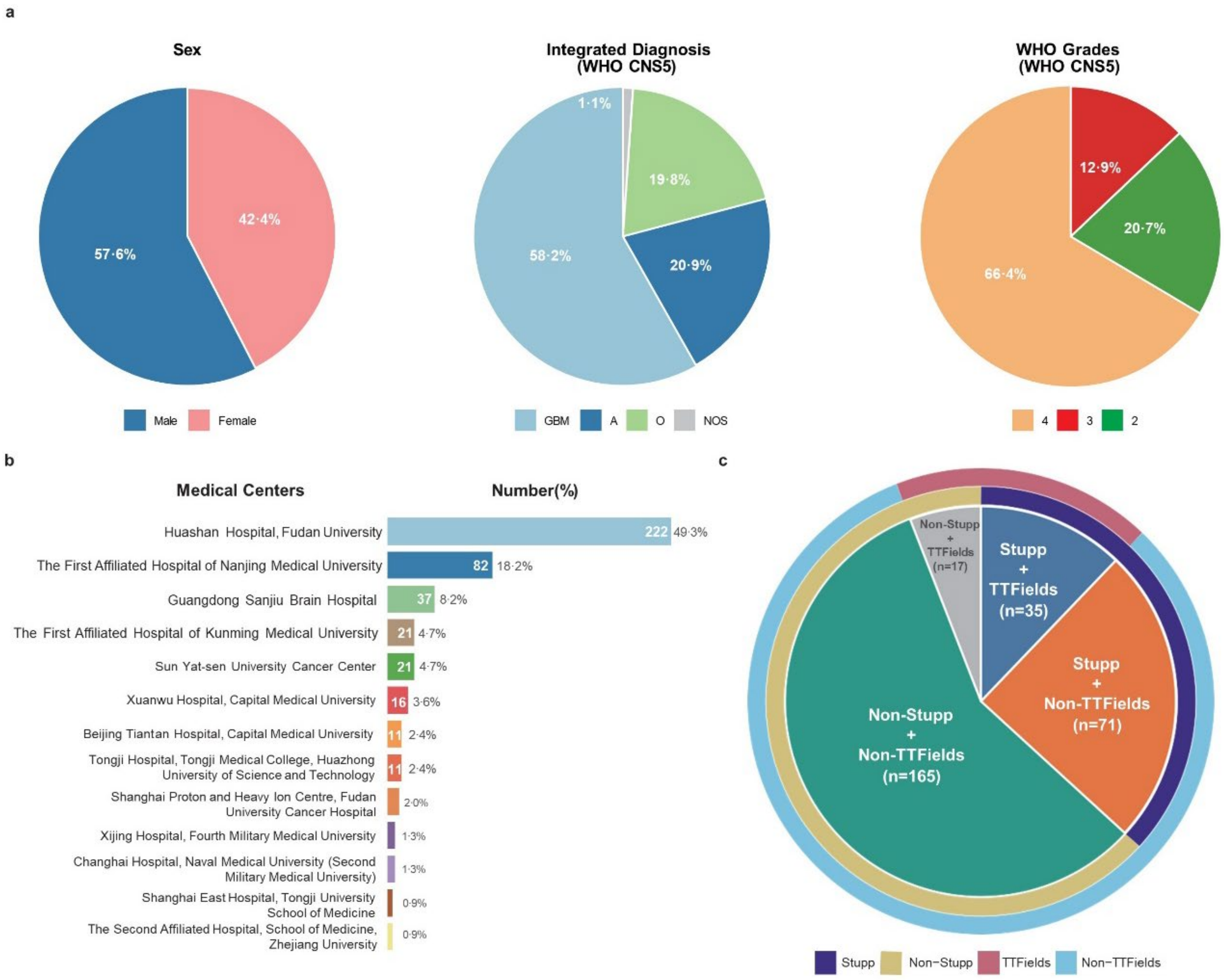

Figure S5. Participant Profile in the VERONICA Study.

a. Baseline Characteristics. b. Participant Recruitment Sources. c. Treatment Regimens.

Abbreviations: GBM, Glioblastoma; A, Astrocytoma; O, Oligodendroglioma; NOS, Not Otherwise Specified; TTFields, Tumor Treating Fields.

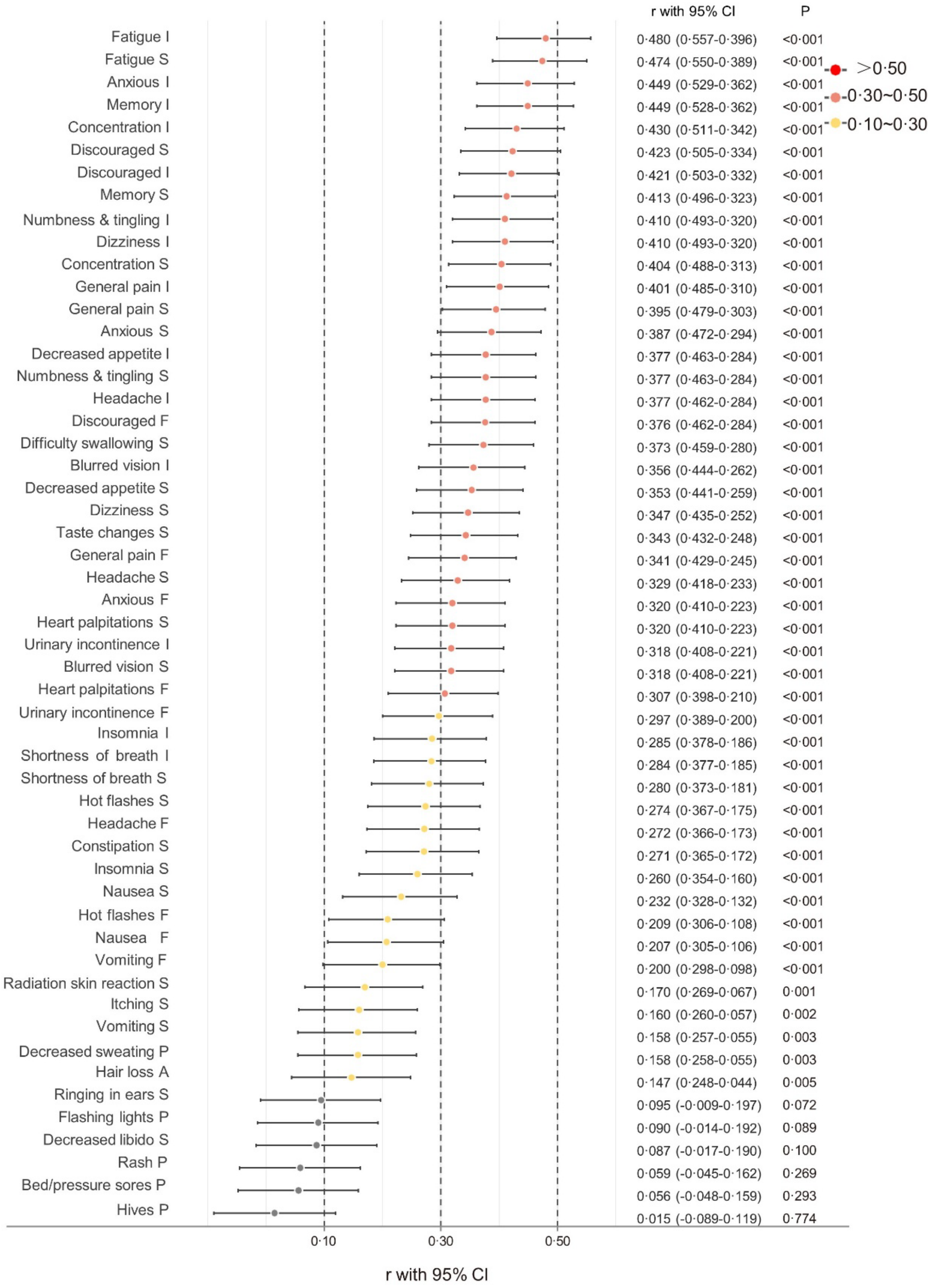

Figure S6. Convergent Validity Analysis Results by Global Health Status/Quality of Life (QoL) Scale Score.

Abbreviations: F, Frequency; I, Interference; S, Severity; P, Presence/Absence; A, Amount.

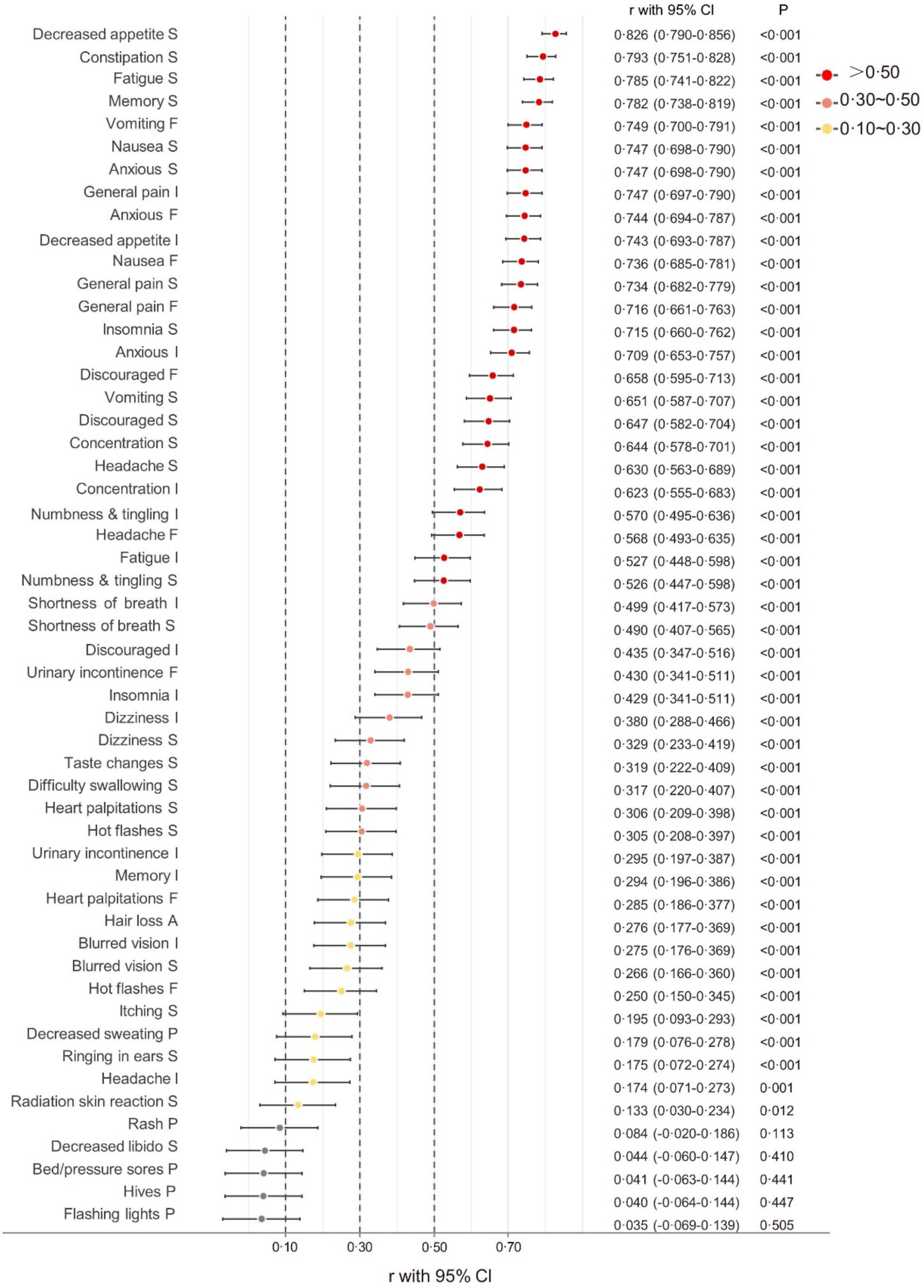

Figure S7. Convergent Validity Analysis Results by Functional and Symptom Domains.

Abbreviations: F, Frequency; I, Interference; S, Severity; P, Presence/Absence; A, Amount.

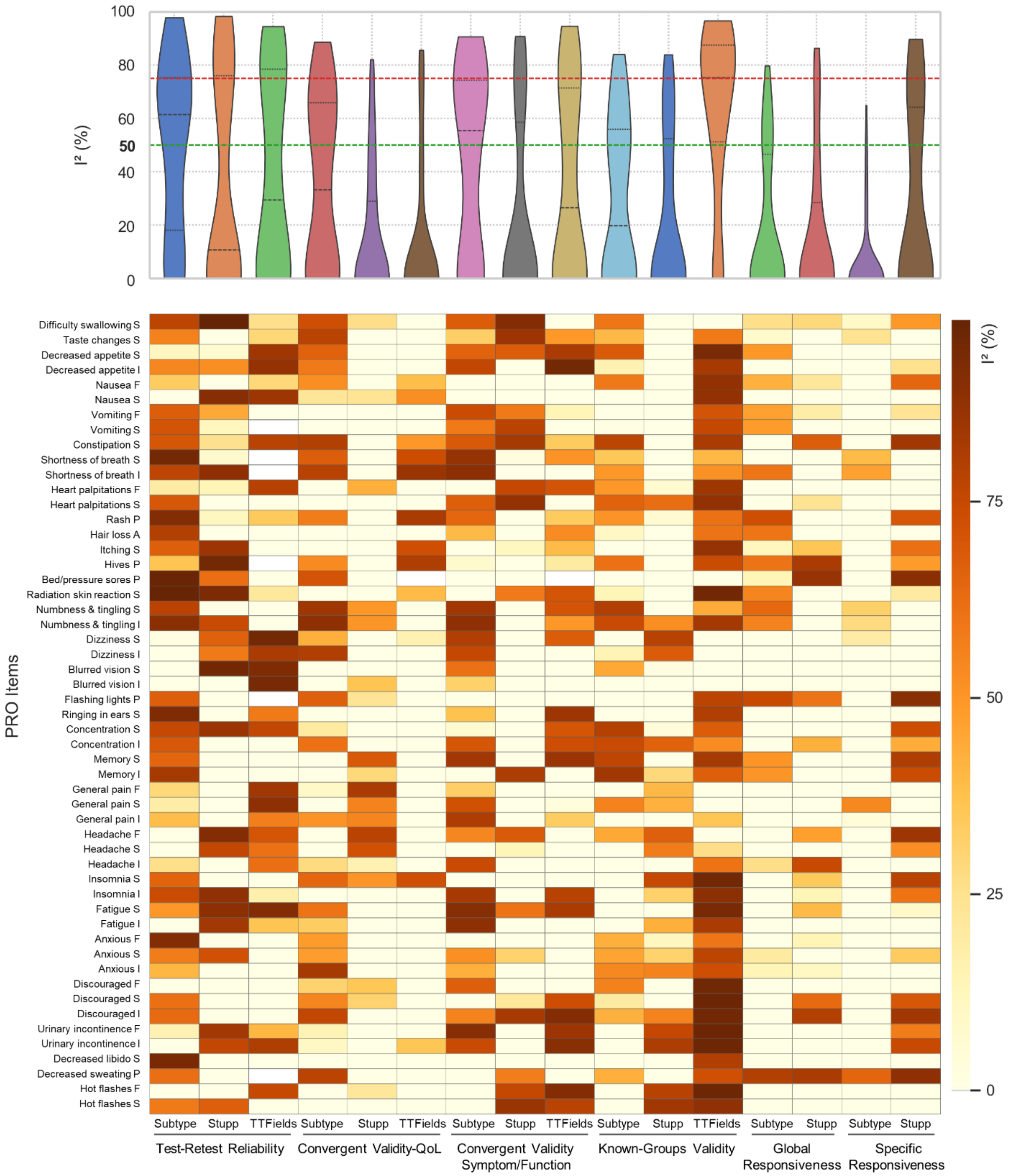

Figure S8. Subgroup Analysis Results.

Abbreviations: F, Frequency; I, Interference; S, Severity; P, Presence/Absence; A, Amount; TTFields, Tumor Treating Fields.

Table S1. Ethical Approval Information of Participating Centres.

Centre ID	Participating Centre	Ethical Approving Committee	Approval Number
01	Huashan Hospital, Fudan University	Huashan Institutional Review Board (HIRB), Huashan Hospital, Fudan University	KY2022-681
02	Beijing Tiantan Hospital, Capital Medical University	Institutional Review Board of Beijing Tiantan Hospital, Capital Medical University	KY2023-146-02
03	Xuanwu Hospital, Capital Medical University	Institutional Review Board of Xuanwu Hospital, Capital Medical University	2022-181
04	Sun Yat-Sen University Cancer Centre	Institutional Review Board of Sun Yat-Sen University Cancer Centre	B2022-695-01
05	Xijing Hospital, Fourth Military Medical University	Institutional Review Board of Xijing Hospital, Fourth Military Medical University	2022-11-29
07	Shanghai Proton and Heavy Ion Centre, Fudan University Cancer Hospital	Institutional Review Board of Shanghai Proton and Heavy Ion Centre, Fudan University Cancer Hospital	2301-61-01
08	The Second Affiliated Hospital, School of Medicine, Zhejiang University	Institutional Review Board of The Second Affiliated Hospital, School of Medicine, Zhejiang University	2023-0438
09	The First Affiliated Hospital of Nanjing Medical University	Institutional Review Board of The First Affiliated Hospital of Nanjing Medical University	2022-SR-482
10	Tongji Hospital, Tongji Medical College, Huazhong University of Science and Technology	Institutional Review Board of Tongji Hospital, Tongji Medical College, Huazhong University of Science and Technology	2023-S024
12	Guangdong Sanjiu Brain Hospital	Institutional Review Board of Guangdong Sanjiu Brain Hospital	2022-020-022
13	The First Affiliated Hospital of Kunming Medical University	Institutional Review Board of The First Affiliated Hospital of Kunming Medical University	2022-10-29
15	Changhai Hospital, Naval Medical University (Second Military Medical University)	Institutional Review Board of Changhai Hospital, Naval Medical University (Second Military Medical University)	B2023-020
16	Shanghai East Hospital, Tongji University School of Medicine	Institutional Review Board of Shanghai East Hospital, Tongji University School of Medicine	2023-016

Table S2. Anchor Linkages Among PRO-CTCAE, CTCAE 5·0, QLQ-C30, and GIC.

NO.	PRO-CTCAE item	PRO-CTCAE System	CTCAE5·0	QLQ-C30 Functional or Symptom Domain	QLQ-C30 Overall	QLQ-C30 Item Numbers	GIC Overall	GIC Special Functional Domain
1a	Difficulty swallowing S	Oral	Dysphagia	Physical functioning	Global health status/QOL	1,2,3,4,5+29,30	Global quality of life	Physical condition
2a	Taste changes S	Gastrointestinal	Gustatory disturbance	Physical functioning	Global health status/QOL	1,2,3,4,5+29,30	Global quality of life	Physical condition
3a	Decreased appetite S	Gastrointestinal	Anorexia	Appetite loss	Global health status/QOL	13+29,30	Global quality of life	Emotional state
3b	Decreased appetite I	Gastrointestinal	Anorexia	Appetite loss	Global health status/QOL	13+29,30	Global quality of life	Emotional state
4a	Nausea F	Gastrointestinal	Nausea	Nausea and vomiting	Global health status/QOL	14,15+29,30	Global quality of life	Physical condition
4b	Nausea S	Gastrointestinal	Nausea	Nausea and vomiting	Global health status/QOL	14,15+29,30	Global quality of life	Physical condition
5a	Vomiting F	Gastrointestinal	Vomiting	Nausea and vomiting	Global health status/QOL	14,15+29,30	Global quality of life	Physical condition
5b	Vomiting S	Gastrointestinal	Vomiting	Nausea and vomiting	Global health status/QOL	14,15+29,30	Global quality of life	Physical condition
6a	Constipation S	Gastrointestinal	Constipation	Constipation	Global health status/QOL	16+29,30	Global quality of life	Physical condition
7a	Shortness of breath S	Respiratory	Dyspnea	Dyspnoea	Global health status/QOL	8+29,30	Global quality of life	Physical condition
7b	Shortness of breath I	Respiratory	Dyspnea	Dyspnoea	Global health status/QOL	8+29,30	Global quality of life	Physical condition
8a	Heart palpitations F	Cardio/Circulatory	Palpitations	Physical functioning	Global health status/QOL	1,2,3,4,5+29,30	Global quality of life	Physical condition
8b	Heart palpitations S	Cardio/Circulatory	Palpitations	Physical functioning	Global health status/QOL	1,2,3,4,5+29,30	Global quality of life	Physical condition
9a	Rash P	Cutaneous	Maculopapular rash	Physical functioning	Global health status/QOL	1,2,3,4,5+29,30	Global quality of life	Skin condition
10a	Hair loss A	Cutaneous	Hair loss	Physical functioning	Global health status/QOL	1,2,3,4,5+29,30	Global quality of life	Skin condition
11a	Itching S	Cutaneous	Pruritus	Physical functioning	Global health status/QOL	1,2,3,4,5+29,30	Global quality of life	Skin condition
12a	Hives P	Cutaneous	Urticaria	Physical functioning	Global health status/QOL	1,2,3,4,5+29,30	Global quality of life	Skin condition
13a	Bed/pressure sores P	Cutaneous	Skin ulcer	Physical functioning	Global health status/QOL	1,2,3,4,5+29,30	Global quality of life	Skin condition
14a	Radiation skin reaction S	Cutaneous	Radiation dermatitis	Physical functioning	Global health status/QOL	1,2,3,4,5+29,30	Global quality of life	Skin condition
15a	Numbness & tingling S	Neurological	Peripheral sensory neuropathy	Physical functioning	Global health status/QOL	1,2,3,4,5+29,30	Global quality of life	Physical condition
15b	Numbness & tingling I	Neurological	Peripheral sensory neuropathy	Physical functioning	Global health status/QOL	1,2,3,4,5+29,30	Global quality of life	Physical condition

NO.	PRO-CTCAE item	PRO-CTCAE System	CTCAE5·0	QLQ-C30 Functional or Symptom Domain	QLQ-C30 Overall	QLQ-C30 Item Numbers	GIC Overall	GIC Special Functional Domain
16a	Dizziness S	Neurological	Dizziness	Physical functioning	Global health status/QOL	1,2,3,4,5+29,30	Global quality of life	Physical condition
16b	Dizziness I	Neurological	Dizziness	Physical functioning	Global health status/QOL	1,2,3,4,5+29,30	Global quality of life	Physical condition
17a	Blurred vision S	Visual/Perceptual	Blurred vision	Physical functioning	Global health status/QOL	1,2,3,4,5+29,30	Global quality of life	Physical condition
17b	Blurred vision I	Visual/Perceptual	Blurred vision	Physical functioning	Global health status/QOL	1,2,3,4,5+29,30	Global quality of life	Physical condition
18a	Flashing lights P	Visual/Perceptual	Photopsia	Physical functioning	Global health status/QOL	1,2,3,4,5+29,30	Global quality of life	Physical condition
19a	Ringing in ears S	Visual/Perceptual	Tinnitus	Physical functioning	Global health status/QOL	1,2,3,4,5+29,30	Global quality of life	Physical condition
20a	Concentration S	Attention/Memory	Concentration disturbance	Cognitive functioning	Global health status/QOL	20,25+29,30	Global quality of life	Emotional state
20b	Concentration I	Attention/Memory	Concentration disturbance	Cognitive functioning	Global health status/QOL	20,25+29,30	Global quality of life	Emotional state
21a	Memory S	Attention/Memory	Memory impairment	Cognitive functioning	Global health status/QOL	20,25+29,30	Global quality of life	Emotional state
21b	Memory I	Attention/Memory	Memory impairment	Cognitive functioning	Global health status/QOL	20,25+29,30	Global quality of life	Emotional state
22a	General pain F	Pain	General pain	Pain	Global health status/QOL	9,19+29,30	Global quality of life	Emotional state
22b	General pain S	Pain	General pain	Pain	Global health status/QOL	9,19+29,30	Global quality of life	Emotional state
22c	General pain I	Pain	General pain	Pain	Global health status/QOL	9,19+29,30	Global quality of life	Emotional state
23a	Headache F	Pain	Headache	Pain	Global health status/QOL	9,19+29,30	Global quality of life	Emotional state
23b	Headache S	Pain	Headache	Pain	Global health status/QOL	9,19+29,30	Global quality of life	Emotional state
23c	Headache I	Pain	Headache	Pain	Global health status/QOL	9,19+29,30	Global quality of life	Emotional state
24a	Insomnia S	Sleep/Wake	Insomnia	Insomnia	Global health status/QOL	11+29,30	Global quality of life	Emotional state
24b	Insomnia I	Sleep/Wake	Insomnia	Insomnia	Global health status/QOL	11+29,30	Global quality of life	Emotional state
25a	Fatigue S	Sleep/Wake	Fatigue, somnolence	Fatigue	Global health status/QOL	10,12,18+29,30	Global quality of life	Emotional state
25b	Fatigue I	Sleep/Wake	Fatigue, somnolence	Fatigue	Global health status/QOL	10,12,18+29,30	Global quality of life	Emotional state
26a	Anxious F	Mood	Anxiety	Emotional functioning	Global health status/QOL	21,22,23,24+29,30	Global quality of life	Emotional state
26b	Anxious S	Mood	Anxiety	Emotional functioning	Global health status/QOL	21,22,23,24+29,30	Global quality of life	Emotional state

NO.	PRO-CTCAE item	PRO-CTCAE System	CTCAE5·0	QLQ-C30 Functional or Symptom Domain	QLQ-C30 Overall	QLQ-C30 Item Numbers	GIC Overall	GIC Special Functional Domain
26c	Anxious I	Mood	Anxiety	Emotional functioning	Global health status/QOL	21,22,23,24+29,30	Global quality of life	Emotional state
27a	Discouraged F	Mood	Depression	Emotional functioning	Global health status/QOL	21,22,23,24+29,30	Global quality of life	Emotional state
27b	Discouraged S	Mood	Depression	Emotional functioning	Global health status/QOL	21,22,23,24+29,30	Global quality of life	Emotional state
27c	Discouraged I	Mood	Depression	Emotional functioning	Global health status/QOL	21,22,23,24+29,30	Global quality of life	Emotional state
28a	Urinary incontinence F	Gynecologic/Urinary	Incontinence	Physical functioning	Global health status/QOL	1,2,3,4,5+29,30	Global quality of life	Physical condition
28b	Urinary incontinence I	Gynecologic/Urinary	Incontinence	Physical functioning	Global health status/QOL	1,2,3,4,5+29,30	Global quality of life	Physical condition
29a	Decreased libido S	Sexual	Decreased libido	Emotional functioning	Global health status/QOL	21,22,23,24+29,30	Global quality of life	Emotional state
30a	Decreased sweating P	Miscellaneous	Hypohidrosis	Physical functioning	Global health status/QOL	1,2,3,4,5+29,30	Global quality of life	Physical condition
31a	Hot flashes F	Miscellaneous	Hyperhidrosis	Physical functioning	Global health status/QOL	1,2,3,4,5+29,30	Global quality of life	Physical condition
31b	Hot flashes S	Miscellaneous	Hyperhidrosis	Physical functioning	Global health status/QOL	1,2,3,4,5+29,30	Global quality of life	Physical condition

Abbreviations: F, Frequency; I, Interference; S, Severity; P, Presence/Absence; A, Amount.

Table S3. Baseline Characteristics of Participants in the Patient Pilot Testing.

Variables	Total (n = 30)
**Age, Mean ± SD**	48·0±16·0
**Sex, n(%)**	
Female	14 (53·3)
Male	16 (46·7)
**WHO CNS5 Grade, n(%)**	
3	6 (20·0)
4	24 (80·0)
**KPS, n(%)**	
≥80	25 (83·3)
50-70	4 (13·3)
≤40	1 (3·3)
**IDH mutation status, n(%)**	
MUT	9 (30·0)
WT	21 (70·0)

Table S4. Results of 1st Round Delphi Survey and the Patient Pilot Testing.

PRO-CTCAE System	PRO-CTCAE Symptom	Category [a]	Mean Importance Rating [b]	Spearman Rank Correlations (r_s) [c]	Patient-Reported Positivity Rate [d] (n=30)	Conclusion
Visual/Perceptual	Blurred vision	T2	5·00	0·38	0·40	Confirmed
Gastrointestinal	Vomiting	T2	4·83	0·41	0·37	Confirmed
Neurological	Dizziness	T2	4·50	0·58	0·37	Confirmed
Pain	General pain	T3	4·50	0·52	0·47	Confirmed
Sleep/Wake	Fatigue	T2	4·33	0·74	0·67	Confirmed
Mood	Sad [e]	T3	4·17	0·56	0·50	Pending
Attention/Memory	Memory	T2	4·17	0·50	0·53	Confirmed
Gastrointestinal	Decreased appetite	T2	4·00	0·66	0·37	Confirmed
Mood	Anxious	T3	4·00	0·61	0·47	Confirmed
Gastrointestinal	Nausea	T2	4·00	0·49	0·33	Confirmed
Respiratory	Shortness of breath	T2	4·00	0·44	0·13	Confirmed
Visual/Perceptual	Flashing lights	SD	4·00	0·42	0·10	Confirmed
Attention/Memory	Concentration	T2	3·83	0·61	0·60	Confirmed
Visual/Perceptual	Ringing in ears	S5	3·83	0·40	0·23	Confirmed
Mood	Discouraged [e]	T3	3·83	0·39	0·53	Pending
Cardio/Circulatory	Heart palpitations	T2	3·50	0·59	0·13	Confirmed
Miscellaneous	Hot flashes	T2	3·50	0·39	0·47	Confirmed
Cutaneous	Itching	S5	3·33	0·59	0·47	Confirmed
Cutaneous	Hives	SD	3·33	0·48	0·27	Confirmed
Miscellaneous	Decreased sweating	SD	3·33	0·38	0·27	Confirmed
Gastrointestinal	Taste changes	S5	3·17	0·41	0·17	Confirmed
Pain	Headache [f]	T3	5·00	0·26	0·37	Confirmed
Neurological	Numbness & tingling	T2	4·83	0·23	0·17	Pending

PRO-CTCAE System	PRO-CTCAE Symptom	Category [a]	Mean Importance Rating [b]	Spearman Rank Correlations (r_s) [c]	Patient-Reported Positivity Rate [d] (n=30)	Conclusion
Oral	Difficulty swallowing	S5	4·50	0·05	0·13	Pending
Sleep/Wake	Insomnia	T2	4·17	0·30	0·57	Pending
Cutaneous	Radiation skin reaction	S5	4·17	0·29	0·13	Pending
Genitourinary	Urinary incontinence	T2	4·17	0·25	0·13	Pending
Cutaneous	Bed/pressure sores	SD	4·17	0·25	0·03	Pending
Gastrointestinal	Hiccups	T2	4·00	0·18	0·30	Pending
Cutaneous	Hair loss	S5	3·67	0·30	0·47	Pending
Visual/Perceptual	Visual floaters	SD	3·67	0·23	0·13	Pending
Miscellaneous	Bruising	SD	3·67	0·13	0·17	Pending
Cutaneous	Skin darkening	SD	3·50	0·19	0·20	Pending
Cutaneous	Rash [f]	SD	3·50	0·16	0·30	Confirmed
Miscellaneous	Increased sweating	T2	3·33	0·29	0·40	Pending
Respiratory	Cough	T2	3·33	0·24	0·10	Pending
Gastrointestinal	Constipation	S5	3·17	0·11	0·17	Pending
Cardio/Circulatory	Swelling	T3	3·00	0·28	0·17	Pending
Oral	Hoarseness	S5	3·00	0·04	0·33	Pending
Oral	Dry mouth	S5	2·83	0·29	0·70	Pending
Gastrointestinal	Diarrhea	S5	2·83	0·17	0·27	Pending
Oral	Mouth/throat sores	T2	2·83	0·16	0·13	Pending
Cutaneous	Stretch marks	SD	2·67	0·25	0·03	Pending
Oral	Voice quality changes	SD	2·67	0·13	0·17	Pending

[a] **Category**: Symptoms were grouped by PRO-CTCAE item structure for pilot-testing analyses: S5, single item with five response options; T2, two-item symptoms; T3, three-item symptoms; SD, single dichotomous (presence/absence) item.

[b] **Mean Importance Rating**: Mean rating from 1st round Delphi survey using a five-point Likert scale (higher scores indicate greater clinical importance).

[c] **Spearman Rank Correlations (r_s)**: Symptom–total associations were estimated in the pilot testing using Spearman rank correlation between each symptom score and the

total score (sum of all symptom scores). For this analysis only, PRO-CTCAE response options were prespecified to be analytically recoded into standardised numeric scores to allow comparability across symptom structures, with the maximum possible symptom score standardised to 6. Recoding schemes were: S5 (0, 1·5, 3, 4·5, 6); T2 (each item: 0, 0·75, 1·5, 2·25, 3; summed across two items); T3 (each item: 0, 0·5, 1, 1·5, 2; summed across three items); SD (No=0, Yes=6). This analytical recoding was used solely for correlation/ranking purposes and did not modify the original PRO-CTCAE item wording, response options, scoring format, or administration.

[d] **Patient-Reported Positivity Rate**: Proportion of pilot-testing participants reporting at least mild symptom burden (i.e., any response above "none/not at all", where applicable).

[e] In the official Simplified Chinese version of the PRO-CTCAE® item library, "discouraged" and "sad" are translated as "没有任何事情可以让你高兴/振作起来的感觉" and "悲伤或不开心的感觉", respectively. Based on patient pilot testing and expert feedback, these items were difficult to clearly differentiate in the Chinese context and were considered conceptually overlapping; both also map to the same CTCAE v5·0 adverse event domain (depression). Therefore, although both ranked highly in item prioritisation, only one was retained as a confirmed symptom in the final instrument to reduce redundancy and respondent burden. So, they were classified as pending symptoms in this round.

[f] Following the 1st round Delphi survey and subsequent discussion, "headache" and "rash" were additionally classified as confirmed symptoms.

Table S5. Results of 2nd Round Delphi Survey.

No.	PRO-CTCAE Symptom	Mean Importance Rating	CVI	CV	Conclusion
1	Insomnia	4·69	1	0·1	Confirmed
2	Discouraged [a]	4·44	1	0·16	Confirmed
3	Urinary incontinence	4·25	1	0·14	Confirmed
4	Hair loss	4·25	0·88	0·26	Confirmed
5	Sad [a]	4·06	0·94	0·26	Excluded
6	Difficulty swallowing	4·06	0·88	0·32	Confirmed
7	Bed/Pressure sores	4·06	0·88	0·26	Confirmed
8	Decreased libido	4	1	0·20	Confirmed
9	Radiation skin reaction	3·94	0·88	0·30	Confirmed
10	Numbness/tingling	3·94	0·88	0·29	Confirmed
11	Constipation	3·63	0·88	0·30	Confirmed
12	Swelling Bloating	3·56	0·81	0·38	Excluded
13	Bruising	3·50	0·94	0·26	Excluded
14	Swelling	3·44	0·81	0·32	Excluded
15	Mouth/throat sores	3·31	0·69	0·38	Excluded
16	Increased sweating	3·25	0·81	0·33	Excluded
17	Hiccup	3·19	0·81	0·29	Excluded
18	Cough	3·19	0·75	0·37	Excluded
19	Skin darkening	3·19	0·69	0·35	Excluded
20	Visual floats	3·13	0·75	0·35	Excluded
21	Diarrhea	3	0·75	0·37	Excluded
22	Dry mouth	3	0·69	0·34	Excluded
23	Hoarseness	2·94	0·63	0·4	Excluded
24	Stretch marks	2·63	0·56	0·36	Excluded
25	Voice quality changes	2·38	0·44	0·34	Excluded

[a] In the official Simplified Chinese version of the PRO-CTCAE® item library, “discouraged” and “sad” are translated as “没有任何事情可以让你高兴/振作起来的感觉” and “悲伤或不开心的感觉”, respectively. Based on patient pilot testing and expert feedback, these items were difficult to clearly differentiate in the Chinese context and were considered conceptually overlapping; both also map to the same CTCAE v5·0 adverse event domain (depression). Therefore, although both ranked highly in item prioritisation, only one was retained as a confirmed symptom in the final instrument to reduce redundancy and respondent burden.

Table S6a. VERONICA Patient Baseline and Follow-up Data Summary (Upper Part).

No.	Patient ID	Centre ID [a]	Integrated Diagnosis	CNS5 WHO Grade	Radiographic Gross Total Resection	Disease Stage (Initial/Recurrence)	Baseline Visit Date	Age at Baseline	Sex	Weight (kg)	Height (m)	V2-KPS	V2-PRO	V2b-PRO	V2-QLQ-C30
1	01-001	01	A	4	1	Initial	2022/9/21	30	Male	70	1·74	80	1	1	1
2	01-002	01	O	2	1	Initial	2022/9/22	56	Female	53	1·51	50	1	1	1
3	01-003	01	GBM	4	1	Initial	2022/9/22	37	Male	54	1·65	80	1	1	1
4	01-004	01	GBM	4	1	Initial	2022/9/23	59	Female	62	1·62	80	1	1	1
5	01-005	01	GBM	4	1	Recurrence	2022/9/23	60	Female	58	1·65	80	1	1	1
6	01-006	01	GBM	4	0	Initial	2022/9/30	59	Female	63	1·65	90	1	1	1
7	01-007	01	A	2	1	Recurrence	2022/9/30	39	Male	70	1·80	90	1	1	1
8	01-008	01	O	2	0	Initial	2022/10/17	36	Female	48	1·67	90	1	1	1
9	01-011	01	O	2	0	Initial	2022/10/26	43	Female	78	1·65	50	1	N/A	N/A
10	01-012	01	GBM	4	1	Initial	2022/10/27	39	Male	70	1·7	80	1	1	1
11	01-013	01	GBM	4	1	Initial	2022/10/30	64	Female	72	1·65	60	1	1	1
12	01-014	01	GBM	4	1	Initial	2022/10/30	54	Male	70	1·7	60	1	1	1
13	01-015	01	GBM	4	0	Initial	2022/10/30	66	Male	85	1·8	50	1	1	1
14	01-016	01	O	3	0	Initial	2022/10/30	49	Female	52	1·58	90	1	1	1
15	01-017	01	O	3	1	Initial	2022/10/30	27	Female	45	1·62	50	1	1	1
16	01-019	01	O	2	1	Initial	2022/11/3	48	Male	70	1·7	60	1	1	1
17	01-020	01	O	3	1	Initial	2022/11/10	55	Female	52.5	1·58	80	1	1	1
18	01-021	01	A	2	1	Initial	2022/11/10	27	Female	53	1·65	70	1	1	1
19	01-022	01	O	2	1	Initial	2022/11/11	34	Female	45	1·55	60	1	1	1
20	01-023	01	GBM	4	0	Initial	2022/11/11	57	Female	58.5	1·58	90	1	1	1
21	01-024	01	A	4	1	Recurrence	2022/11/13	52	Female	56	1·53	40	1	1	1

No.	Patient ID	Centre ID [a]	Integrated Diagnosis	CNS5 WHO Grade	Radiographic Gross Total Resection	Disease Stage (Initial/Recurrence)	Baseline Visit Date	Age at Baseline	Sex	Weight (kg)	Height (m)	V2-KPS	V2-PRO	V2b-PRO	V2-QLQ-C30
22	01-025	01	A	2	1	Initial	2022/11/17	33	Male	68	1·75	50	1	1	1
23	01-027	01	O	2	0	Initial	2022/11/23	42	Male	65	1·72	90	1	1	1
24	01-028	01	GBM	4	1	Initial	2022/11/23	65	Male	60	1·68	60	1	N/A	1
25	01-029	01	A	2	1	Recurrence	2022/11/23	28	Female	52	1·55	60	1	1	1
26	01-030	01	GBM	4	1	Initial	2022/12/2	46	Male	69	1·75	90	1	1	1
27	01-031	01	GBM	4	0	Initial	2022/11/27	68	Female	67.5	1·63	50	1	1	1
28	01-032	01	A	4	1	Initial	2022/12/2	41	Female	65	1·68	80	1	N/A	N/A
29	01-033	01	A	4	1	Initial	2022/11/27	20	Female	63	1·7	90	1	1	1
30	01-034	01	O	2	0	Initial	2022/12/1	46	Male	75	1·71	90	1	1	1
31	01-035	01	GBM	4	1	Initial	2022/12/2	56	Female	61	1·65	90	1	1	1
32	01-036	01	A	3	0	Recurrence	2022/12/5	38	Male	99	1·7	50	1	1	1
33	01-037	01	O	3	1	Initial	2022/12/6	52	Female	47.5	1·57	80	1	1	1
34	01-038	01	GBM	4	1	Initial	2022/12/13	55	Male	91	1·76	40	1	N/A	1
35	01-039	01	A	4	1	Initial	2022/12/7	35	Female	55	1·6	80	1	1	1
36	01-041	01	A	3	1	Recurrence	2022/12/11	46	Male	80	1·76	60	1	N/A	1
37	01-042	01	GBM	4	0	Initial	2022/12/11	41	Female	56	1·55	60	1	N/A	1
38	01-044	01	A	2	0	Initial	2022/12/11	39	Female	60	1·63	70	1	1	1
39	01-045	01	O	2	1	Initial	2022/12/29	29	Male	70	1·72	70	1	N/A	1
40	01-046	01	GBM	4	1	Recurrence	2022/12/29	39	Male	56	1·70	70	1	N/A	1
41	01-047	01	GBM	4	1	Initial	2022/12/29	37	Male	77	1·78	50	1	N/A	1
42	01-049	01	GBM	4	1	Initial	2022/12/25	48	Female	55	1·56	90	1	1	1
43	01-050	01	A	4	1	Initial	2022/12/12	36	Male	67	1·7	60	1	N/A	1

No.	Patient ID	Centre ID [a]	Integrated Diagnosis	CNS5 WHO Grade	Radiographic Gross Total Resection	Disease Stage (Initial/Recurrence)	Baseline Visit Date	Age at Baseline	Sex	Weight (kg)	Height (m)	V2-KPS	V2-PRO	V2b-PRO	V2-QLQ-C30
44	01-052	01	GBM	4	1	Initial	2022/12/16	67	Male	80	1·67	80	1	1	1
45	01-053	01	A	3	0	Initial	2023/1/12	34	Male	75	1·71	90	1	1	1
46	01-054	01	O	3	0	Initial	2023/1/14	70	Male	75	1·72	60	1	N/A	1
47	01-055	01	O	3	0	Initial	2023/2/1	45	Male	60	1·68	90	1	1	1
48	01-057	01	GBM	4	0	Initial	2023/2/8	73	Male	65	1·7	30	1	N/A	1
49	01-058	01	GBM	4	1	Initial	2023/2/8	34	Male	80	1·70	70	1	1	1
50	01-059	01	GBM	4	0	Initial	2023/2/12	60	Male	62	1·6	60	1	1	1
51	01-060	01	A	2	0	Initial	2023/2/11	26	Female	60	1·70	70	N/A	N/A	N/A
52	01-061	01	GBM	4	1	Initial	2023/2/12	56	Female	60	1·60	60	1	1	1
53	01-062	01	O	2	0	Recurrence	2023/2/12	33	Male	80	1·75	60	N/A	1	N/A
54	01-063	01	O	2	1	Initial	2023/2/14	48	Female	63.7	1·60	60	1	1	1
55	01-064	01	GBM	4	1	Initial	2023/2/14	44	Female	53	1·63	60	1	1	1
56	01-065	01	GBM	4	1	Recurrence	2023/2/15	27	Male	58	1·65	50	1	1	1
57	01-066	01	GBM	4	1	Recurrence	2023/2/15	51	Female	70	1·65	30	1	1	1
58	01-067	01	A	2	0	Initial	2023/2/16	44	Male	66.5	1·77	70	1	1	1
59	01-068	01	GBM	4	1	Initial	2023/2/26	67	Male	72	1·74	80	1	1	1
60	01-069	01	GBM	4	1	Initial	2023/2/26	52	Female	55	1·60	30	1	1	1
61	01-070	01	A	2	1	Initial	2023/2/24	37	Male	75	1·7	70	1	1	1
62	01-071	01	A	2	0	Initial	2023/2/24	21	Male	89	1·70	60	1	1	1
63	01-072	01	O	2	0	Initial	2023/2/26	35	Female	70	1·67	30	1	1	1
64	01-074	01	O	2	1	Initial	2023/3/7	42	Female	82	1·60	90	1	1	1
65	01-075	01	A	3	1	Recurrence	2023/3/5	39	Male	69	1·76	60	1	1	1

No.	Patient ID	Centre ID [a]	Integrated Diagnosis	CNS5 WHO Grade	Radiographic Gross Total Resection	Disease Stage (Initial/Recurrence)	Baseline Visit Date	Age at Baseline	Sex	Weight (kg)	Height (m)	V2-KPS	V2-PRO	V2b-PRO	V2-QLQ-C30
66	01-076	01	A	4	1	Recurrence	2023/3/15	41	Male	88	1·7	80	1	1	1
67	01-077	01	GBM	4	1	Initial	2023/3/15	65	Male	80	1·7	60	1	1	1
68	01-078	01	GBM	4	1	Initial	2023/3/17	59	Female	54	1·63	40	1	1	1
69	01-079	01	A	4	0	Recurrence	2023/3/28	42	Male	83	1·72	40	1	1	1
70	01-080	01	GBM	4	1	Initial	2023/4/22	49	Female	50	1·6	60	1	1	1
71	01-081	01	O	2	1	Initial	2023/3/18	50	Female	80	1·63	80	1	1	1
72	01-082	01	A	2	1	Recurrence	2023/3/29	31	Male	68	1·80	60	1	1	1
73	01-083	01	GBM	4	1	Initial	2023/3/23	69	Male	54	1·65	70	1	1	1
74	01-084	01	O	3	0	Initial	2023/4/12	36	Male	90	1·78	90	1	1	1
75	01-085	01	A	2	1	Initial	2023/4/12	27	Male	75	1·72	90	1	1	1
76	01-086	01	GBM	4	0	Initial	2023/4/12	73	Male	67.5	1·68	50	1	N/A	N/A
77	01-087	01	GBM	4	0	Initial	2023/4/14	69	Male	57	1·69	60	1	1	1
78	01-088	01	O	3	1	Initial	2023/4/14	49	Male	65	1·68	60	1	1	1
79	01-089	01	GBM	4	0	Recurrence	2023/4/22	53	Male	75	1·78	30	1	N/A	1
80	01-090	01	A	4	0	Recurrence	2023/4/18	45	Male	75	1·77	50	1	1	1
81	01-091	01	GBM	4	1	Initial	2023/5/13	36	Male	75	1·81	70	1	1	1
82	01-092	01	A	2	1	Recurrence	2023/5/13	39	Male	65	1·76	80	1	1	1
83	01-094	01	GBM	4	1	Initial	2023/5/13	65	Female	60	1·6	50	1	1	1
84	01-095	01	A	3	0	Initial	2023/5/13	56	Male	81	1·75	50	1	1	1
85	01-096	01	GBM	4	1	Initial	2023/6/1	48	Female	56	1·61	40	1	N/A	1
86	01-097	01	A	3	1	Recurrence	2023/6/1	39	Female	72	1·63	40	1	1	1
87	01-098	01	GBM	4	0	Initial	2023/6/1	47	Male	60	1·63	90	N/A	1	1

No.	Patient ID	Centre ID [a]	Integrated Diagnosis	CNS5 WHO Grade	Radiographic Gross Total Resection	Disease Stage (Initial/Recurrence)	Baseline Visit Date	Age at Baseline	Sex	Weight (kg)	Height (m)	V2-KPS	V2-PRO	V2b-PRO	V2-QLQ-C30
88	01-099	01	A	3	1	Initial	2023/6/1	43	Male	70	1·7	40	1	1	1
89	01-100	01	GBM	4	0	Initial	2023/7/12	64	Male	60	1·7	40	1	N/A	1
90	01-101	01	GBM	4	1	Initial	2023/7/12	54	Male	57.5	1·73	60	1	N/A	1
91	01-102	01	A	2	1	Initial	2023/7/12	56	Female	64	1·68	40	1	N/A	1
92	01-103	01	GBM	4	0	Initial	2023/12/11	51	Female	54	1·6	90	1	1	1
93	01-106	01	GBM	4	1	Initial	2023/12/3	60	Female	62	1·68	70	1	1	1
94	01-107	01	A	4	1	Initial	2023/12/3	35	Male	87.7	1·83	70	1	1	1
95	01-108	01	O	2	1	Initial	2024/1/7	47	Female	55	1·65	60	1	1	1
96	01-109	01	O	3	0	Initial	2023/12/20	46	Male	65	1·65	80	1	1	1
97	01-110	01	A	4	1	Initial	2024/1/7	57	Female	49	1·6	50	1	1	1
98	01-111	01	O	2	1	Initial	2024/1/7	47	Male	63	1·75	80	1	1	1
99	01-112	01	A	3	1	Initial	2024/1/7	33	Female	70	1·65	60	1	1	1
100	01-113	01	GBM	4	0	Initial	2024/1/7	60	Male	76	1·76	60	1	1	1
101	01-116	01	A	4	1	Initial	2024/1/20	37	Female	69	1·52	90	1	1	1
102	01-117	01	GBM	4	1	Initial	2024/1/20	25	Male	70	1·75	80	1	1	1
103	01-118	01	GBM	4	1	Initial	2024/1/20	44	Male	80	1·72	80	1	1	1
104	01-119	01	O	2	1	Initial	2024/1/20	59	Male	63.7	1·69	50	1	1	1
105	01-120	01	GBM	4	1	Initial	2024/1/30	64	Female	45	1·5	80	1	1	1
106	01-121	01	GBM	4	0	Initial	2024/1/30	59	Female	45	1·5	80	1	1	1
107	01-122	01	A	4	1	Recurrence	2024/1/30	35	Female	45	1·5	50	1	1	1
108	01-123	01	O	2	1	Initial	2024/1/30	52	Female	50	1·5	70	1	1	1
109	01-124	01	GBM	4	1	Initial	2024/1/30	70	Female	50	1·5	40	1	1	1

No.	Patient ID	Centre ID [a]	Integrated Diagnosis	CNS5 WHO Grade	Radiographic Gross Total Resection	Disease Stage (Initial/Recurrence)	Baseline Visit Date	Age at Baseline	Sex	Weight (kg)	Height (m)	V2-KPS	V2-PRO	V2b-PRO	V2-QLQ-C30
110	01-125	01	O	2	0	Initial	2024/2/6	48	Male	70	1·75	90	1	1	1
111	01-126	01	GBM	4	1	Initial	2024/2/6	62	Male	80	1·75	40	1	N/A	1
112	01-127	01	A	2	0	Initial	2024/2/6	24	Male	90	1·73	90	1	1	1
113	01-128	01	O	3	1	Initial	2024/2/6	38	Male	94.5	1·73	90	1	1	1
114	01-130	01	A	4	0	Initial	2024/2/26	54	Male	76	1·7	70	1	1	1
115	01-131	01	GBM	4	1	Initial	2024/2/26	63	Female	50	1·6	60	1	1	1
116	01-132	01	A	3	0	Initial	2024/2/26	64	Male	75	1·7	50	1	1	1
117	01-133	01	GBM	4	1	Initial	2024/2/26	42	Male	75	1·72	60	1	1	1
118	01-134	01	GBM	4	1	Initial	2024/2/26	77	Female	40	1·55	40	1	1	1
119	01-135	01	GBM	4	1	Initial	2024/2/26	54	Male	75	1·75	70	1	1	1
120	01-136	01	O	2	1	Initial	2024/2/26	34	Female	55	1·57	60	1	1	1
121	01-137	01	GBM	4	1	Recurrence	2024/3/7	43	Male	75	1·72	80	1	1	1
122	01-138	01	O	3	1	Recurrence	2024/3/7	47	Female	52	1·6	50	1	1	1
123	01-139	01	GBM	4	0	Initial	2024/3/7	59	Female	50	1·62	50	1	1	1
124	01-140	01	GBM	4	1	Initial	2024/3/7	67	Female	50	1·52	60	1	1	1
125	01-141	01	GBM	4	1	Initial	2024/3/8	56	Male	70	1·70	60	1	1	1
126	01-142	01	A	2	1	Initial	2024/3/11	47	Female	58	1·62	60	1	1	1
127	01-143	01	GBM	4	1	Initial	2024/3/11	37	Female	65	1·60	60	1	1	1
128	01-144	01	O	3	1	Initial	2024/3/11	45	Male	65	1·65	70	1	1	1
129	01-145	01	A	2	0	Initial	2024/3/11	41	Female	52.5	1·55	60	1	1	1
130	01-146	01	A	4	1	Recurrence	2024/3/20	54	Female	71	1·70	70	1	1	1
131	01-147	01	GBM	4	0	Initial	2024/3/20	32	Male	65	1·80	80	1	1	1

No.	Patient ID	Centre ID [a]	Integrated Diagnosis	CNS5 WHO Grade	Radiographic Gross Total Resection	Disease Stage (Initial/Recurrence)	Baseline Visit Date	Age at Baseline	Sex	Weight (kg)	Height (m)	V2-KPS	V2-PRO	V2b-PRO	V2-QLQ-C30
132	01-148	01	GBM	4	0	Initial	2024/3/20	49	Female	64	1·60	70	1	1	1
133	01-149	01	A	2	1	Initial	2024/3/20	26	Female	57	1·70	60	1	1	1
134	01-150	01	GBM	4	1	Initial	2024/3/21	45	Female	54	1·61	70	1	N/A	1
135	01-152	01	GBM	4	0	Initial	2024/3/26	39	Male	85	1·74	40	1	1	1
136	01-153	01	GBM	4	0	Initial	2024/3/27	39	Male	85	1·74	40	1	1	1
137	01-154	01	GBM	4	1	Initial	2024/3/29	61	Male	82	1·76	40	1	1	1
138	01-155	01	GBM	4	0	Initial	2024/4/1	62	Male	65	1·65	70	1	1	1
139	01-156	01	GBM	4	1	Initial	2024/4/7	64	Male	75	1·74	60	1	1	1
140	01-157	01	O	3	1	Recurrence	2024/4/7	56	Male	69	1·68	60	1	1	1
141	01-158	01	A	3	0	Initial	2024/4/7	35	Female	58	1·68	70	1	1	1
142	01-159	01	O	3	0	Initial	2024/4/7	66	Female	55	1·58	60	1	1	1
143	01-160	01	GBM	4	0	Initial	2024/4/15	57	Female	57	1·6	60	1	1	1
144	01-161	01	GBM	4	0	Initial	2024/4/15	76	Female	54	1·56	60	1	1	1
145	01-162	01	A	2	1	Initial	2024/4/15	31	Male	72.5	1·68	70	1	1	1
146	01-163	01	A	2	1	Initial	2024/4/1	44	Male	63	1·7	70	1	1	1
147	01-164	01	GBM	4	1	Recurrence	2024/4/15	53	Male	86	1·73	40	1	1	1
148	01-165	01	O	2	0	Initial	2024/4/17	34	Female	55	1·55	60	1	1	1
149	01-167	01	O	3	0	Initial	2024/4/22	37	Female	50	1·63	70	1	1	1
150	01-168	01	GBM	4	0	Initial	2024/4/22	60	Female	60	1·67	90	1	1	1
151	01-169	01	GBM	4	1	Initial	2024/4/22	32	Male	66	1·72	70	1	1	1
152	01-170	01	GBM	4	0	Recurrence	2024/4/22	31	Male	60	1·8	60	1	1	1
153	01-171	01	GBM	4	0	Initial	2024/4/22	68	Male	80	1·8	50	1	1	1

No.	Patient ID	Centre ID [a]	Integrated Diagnosis	CNS5 WHO Grade	Radiographic Gross Total Resection	Disease Stage (Initial/Recurrence)	Baseline Visit Date	Age at Baseline	Sex	Weight (kg)	Height (m)	V2-KPS	V2-PRO	V2b-PRO	V2-QLQ-C30
154	01-172	01	GBM	4	0	Recurrence	2024/4/29	45	Male	80	1·85	50	1	1	1
155	01-173	01	A	4	1	Initial	2024/4/29	34	Female	58	1·66	60	1	1	1
156	01-174	01	A	3	1	Recurrence	2024/4/29	52	Male	69.5	1·7	40	1	1	1
157	01-175	01	GBM	4	0	Initial	2024/5/8	57	Male	58	1·17	90	1	1	1
158	01-176	01	GBM	4	1	Initial	2024/5/9	50	Male	75	1·72	40	1	1	1
159	01-177	01	O	2	1	Initial	2024/5/8	66	Female	60	1·55	80	1	1	1
160	01-178	01	GBM	4	0	Initial	2024/5/8	45	Female	77	1·59	80	1	1	1
161	01-179	01	A	2	1	Initial	2024/5/8	51	Male	78	1·78	90	1	1	1
162	01-180	01	O	2	1	Initial	2024/5/8	61	Female	62	1·5	80	1	1	1
163	01-181	01	GBM	4	1	Initial	2024/5/10	48	Male	65.8	1·7	60	1	1	1
164	01-182	01	GBM	4	1	Initial	2024/5/21	46	Male	80	1·82	50	1	1	1
165	01-183	01	A	2	0	Recurrence	2024/5/20	30	Male	68	1·58	70	1	1	1
166	01-184	01	GBM	4	1	Initial	2024/5/20	62	Female	57.5	1·6	90	1	1	1
167	01-185	01	O	2	1	Initial	2024/6/6	37	Female	55	1·63	90	1	1	1
168	01-186	01	A	4	1	Recurrence	2024/6/6	36	Female	52	1·58	100	1	1	1
169	01-187	01	GBM	4	0	Initial	2024/6/6	54	Male	64	1·65	80	1	1	1
170	01-188	01	A	2	1	Initial	2024/6/12	31	Female	80	1·6	80	1	1	1
171	01-189	01	GBM	4	1	Initial	2024/6/12	72	Female	60	1·59	50	1	1	1
172	01-190	01	O	2	1	Initial	2024/7/12	57	Female	64	1·55	60	1	1	1
173	01-191	01	GBM	4	1	Initial	2024/7/12	66	Female	60	1·68	90	1	1	1
174	01-192	01	O	2	1	Initial	2024/7/12	48	Female	71	1·63	100	1	1	1
175	01-193	01	GBM	4	0	Initial	2024/7/12	67	Female	48	1·58	90	1	1	1

No.	Patient ID	Centre ID [a]	Integrated Diagnosis	CNS5 WHO Grade	Radiographic Gross Total Resection	Disease Stage (Initial/Recurrence)	Baseline Visit Date	Age at Baseline	Sex	Weight (kg)	Height (m)	V2-KPS	V2-PRO	V2b-PRO	V2-QLQ-C30
176	01-194	01	GBM	4	0	Initial	2024/7/18	51	Female	51	1·55	50	1	1	1
177	01-195	01	GBM	4	0	Initial	2024/7/18	59	Female	67	1·65	70	1	1	1
178	01-196	01	O	2	1	Initial	2024/7/18	46	Male	85	1·75	80	1	1	1
179	01-197	01	O	2	1	Initial	2024/7/25	39	Male	80	1·8	90	1	1	1
180	01-198	01	GBM	4	0	Initial	2024/7/25	57	Male	66	1·65	60	1	1	1
181	01-199	01	GBM	4	0	Initial	2024/7/26	34	Female	55	1·68	100	1	1	1
182	01-200	01	GBM	4	1	Recurrence	2024/7/31	44	Male	70	1·7	100	1	1	1
183	01-201	01	A	4	0	Initial	2024/7/31	34	Male	55	1·69	100	1	1	1
184	01-202	01	GBM	4	1	Initial	2024/8/8	59	Female	55	1·61	50	1	1	1
185	01-203	01	GBM	4	0	Initial	2024/8/7	23	Female	50	1·65	50	1	1	1
186	01-204	01	O	3	0	Initial	2024/8/7	56	Male	67	1·71	60	1	1	1
187	01-205	01	O	2	0	Initial	2024/8/7	36	Male	90	1·72	90	1	1	1
188	01-206	01	GBM	4	0	Initial	2024/8/7	71	Male	75	1·72	50	1	1	1
189	01-208	01	O	3	0	Initial	2024/8/9	46	Female	62.5	1·55	60	1	1	1
190	01-209	01	GBM	4	1	Initial	2024/8/14	49	Male	72	1·7	60	1	1	1
191	01-210	01	O	2	1	Initial	2024/8/16	28	Male	55	1·75	70	1	1	1
192	01-211	01	O	3	0	Initial	2024/8/26	29	Male	74	1·74	100	1	1	1
193	01-212	01	A	2	0	Initial	2024/8/26	43	Male	79	1·65	90	1	1	1
194	01-213	01	A	4	1	Initial	2024/8/26	39	Female	56	1·65	40	1	1	1
195	01-214	01	A	2	1	Initial	2024/8/26	47	Female	60	1·68	50	1	1	1
196	01-215	01	O	3	1	Initial	2024/8/26	35	Male	70	1·78	80	1	1	1
197	01-216	01	GBM	4	1	Initial	2024/8/27	56	Male	102	1·68	50	1	1	1

No.	Patient ID	Centre ID [a]	Integrated Diagnosis	CNS5 WHO Grade	Radiographic Gross Total Resection	Disease Stage (Initial/Recurrence)	Baseline Visit Date	Age at Baseline	Sex	Weight (kg)	Height (m)	V2-KPS	V2-PRO	V2b-PRO	V2-QLQ-C30
198	01-217	01	O	2	1	Initial	2024/8/26	43	Female	49	1·58	80	1	1	1
199	01-218	01	A	2	0	Initial	2024/9/5	35	Male	75	1·83	90	1	1	1
200	01-219	01	O	2	1	Initial	2024/9/5	41	Female	53	1·6	90	1	1	1
201	01-220	01	O	2	1	Initial	2024/9/5	52	Female	59	1·59	60	1	1	1
202	01-221	01	A	4	1	Initial	2024/9/5	58	Female	60	1·58	100	1	1	1
203	01-222	01	GBM	4	0	Initial	2024/9/6	65	Male	90	1·82	90	1	1	1
204	01-223	01	O	2	0	Initial	2024/9/13	49	Male	62.5	1·67	90	1	1	1
205	01-224	01	O	2	1	Recurrence	2024/9/19	38	Female	51	1·58	90	1	1	1
206	01-225	01	GBM	4	0	Initial	2024/9/13	66	Male	77.5	1·75	90	1	1	1
207	01-226	01	GBM	4	1	Initial	2024/9/18	61	Male	75	1·68	100	1	1	1
208	01-227	01	GBM	4	1	Initial	2024/9/18	60	Male	80	1·72	60	1	1	1
209	01-228	01	O	3	0	Initial	2024/9/18	27	Female	74	1·63	70	1	1	1
210	01-229	01	A	4	1	Recurrence	2024/9/19	56	Male	65	1·61	50	1	1	1
211	01-230	01	GBM	4	0	Initial	2024/9/19	57	Female	47	1·6	90	1	1	1
212	01-231	01	GBM	4	1	Initial	2024/9/19	46	Male	60	1·65	40	1	1	1
213	01-232	01	GBM	4	1	Recurrence	2024/9/25	50	Female	57	1·58	50	1	1	1
214	01-233	01	GBM	4	1	Initial	2024/9/26	69	Female	64	1·57	100	1	1	1
215	01-234	01	A	2	1	Initial	2024/9/26	43	Male	53	1·69	80	1	1	1
216	01-235	01	A	2	1	Initial	2024/9/26	35	Male	59	1·63	100	1	1	1
217	01-236	01	O	2	0	Initial	2024/9/27	31	Female	55	1·6	70	1	1	1
218	01-237	01	GBM	4	0	Initial	2024/9/30	53	Female	54	1·58	90	1	1	1
219	01-238	01	GBM	4	0	Initial	2024/9/29	47	Female	45	1·54	60	1	1	1

No.	Patient ID	Centre ID [a]	Integrated Diagnosis	CNS5 WHO Grade	Radiographic Gross Total Resection	Disease Stage (Initial/Recurrence)	Baseline Visit Date	Age at Baseline	Sex	Weight (kg)	Height (m)	V2-KPS	V2-PRO	V2b-PRO	V2-QLQ-C30
220	01-239	01	A	4	1	Recurrence	2024/9/29	54	Female	70	1·61	80	1	1	1
221	01-240	01	A	2	1	Initial	2024/9/30	36	Female	65	1·64	90	1	1	1
222	01-241	01	GBM	4	1	Initial	2024/10/8	82	Female	44	1·6	100	1	1	1
223	02-002	02	GBM	4	1	Initial	2024/3/5	65	Female	62	1·7	90	1	1	1
224	02-003	02	GBM	4	1	Initial	2024/3/5	41	Male	83	1·8	90	1	1	1
225	02-004	02	O	3	1	Initial	2024/3/12	44	Male	83	1·76	90	1	1	1
226	02-005	02	O	3	1	Initial	2024/3/12	44	Female	75	1·58	90	1	1	1
227	02-006	02	O	2	1	Initial	2024/3/19	47	Female	75	1·58	100	1	1	1
228	02-008	02	O	3	1	Initial	2024/4/12	29	Female	45	1·50	100	1	1	1
229	02-009	02	O	2	1	Initial	2024/4/12	26	Male	90	1·8	90	1	1	1
230	02-010	02	O	3	1	Initial	2024/4/23	37	Male	78	1·75	100	1	1	1
231	02-011	02	O	2	1	Initial	2024/5/14	40	Male	95.1	1·78	90	1	1	1
232	02-012	02	A	2	1	Initial	2024/5/14	27	Male	80	1·73	90	1	1	1
233	02-013	02	A	3	1	Initial	2024/5/28	28	Male	75	1·79	90	1	1	1
234	03-001	03	O	2	1	Initial	2023/2/8	44	Female	63	1·58	80	1	1	1
235	03-002	03	A	3	1	Initial	2023/3/8	41	Female	51	1·62	80	1	1	1
236	03-003	03	A	2	0	Initial	2023/3/13	45	Male	75	1·7	100	1	1	1
237	03-004	03	O	2	1	Initial	2023/3/15	48	Male	95	1·64	100	1	1	1
238	03-005	03	O	2	1	Initial	2023/3/21	27	Male	65	1·72	100	1	1	1
239	03-006	03	A	4	1	Initial	2023/3/21	52	Male	70	1·73	100	1	1	1
240	03-007	03	O	2	1	Initial	2023/3/30	31	Female	60	1·58	100	1	1	1
241	03-008	03	GBM	4	1	Initial	2023/4/18	70	Female	52	1·58	40	1	1	1

No.	Patient ID	Centre ID [a]	Integrated Diagnosis	CNS5 WHO Grade	Radiographic Gross Total Resection	Disease Stage (Initial/Recurrence)	Baseline Visit Date	Age at Baseline	Sex	Weight (kg)	Height (m)	V2-KPS	V2-PRO	V2b-PRO	V2-QLQ-C30
242	03-009	03	NOS	4	1	Initial	2023/5/8	51	Female	53	1·58	100	1	1	1
243	03-010	03	O	2	1	Initial	2023/5/8	59	Male	67	1·7	100	1	1	1
244	03-011	03	GBM	4	1	Initial	2023/6/14	43	Male	80	1·7	100	1	1	1
245	03-012	03	O	3	1	Initial	2023/6/12	46	Male	72	1·68	90	1	1	1
246	03-013	03	GBM	4	1	Initial	2023/6/14	46	Female	55	1·6	90	1	N/A	1
247	03-014	03	GBM	4	1	Initial	2024/6/27	36	Male	85	1·75	100	1	N/A	1
248	03-015	03	GBM	4	1	Initial	2024/4/29	51	Male	70	1·66	100	1	1	1
249	03-016	03	GBM	4	1	Initial	2024/6/27	74	Female	43	1·5	100	1	1	1
250	04-001	04	GBM	4	1	Recurrence	2023/3/23	51	Male	76	1·7	60	N/A	1	N/A
251	04-002	04	A	3	1	Initial	2023/3/27	36	Male	70	1·73	70	1	1	1
252	04-003	04	GBM	4	1	Initial	2023/3/28	53	Male	58	1·58	90	1	1	1
253	04-004	04	O	3	1	Initial	2023/4/20	50	Female	63	1·63	80	1	N/A	1
254	04-005	04	GBM	4	0	Recurrence	2023/4/20	32	Female	47	1·55	80	1	N/A	1
255	04-006	04	GBM	4	1	Initial	2023/4/20	67	Female	60	1·58	60	N/A	N/A	N/A
256	04-007	04	GBM	4	1	Initial	2023/4/21	54	Male	52	1·54	80	1	N/A	N/A
257	04-011	04	A	2	1	Initial	2023/5/16	36	Male	89.1	1·78	90	1	1	1
258	04-013	04	O	3	1	Recurrence	2023/5/9	40	Female	80	1·6	70	1	1	1
259	04-014	04	GBM	4	1	Recurrence	2023/5/9	44	Female	54	1·58	40	1	N/A	1
260	04-015	04	O	3	1	Initial	2023/5/17	43	Male	66	1·75	70	1	1	1
261	04-016	04	GBM	4	1	Initial	2023/5/25	36	Male	76	1·67	80	1	1	1
262	04-017	04	A	4	1	Recurrence	2023/5/30	34	Male	60	1·68	60	N/A	N/A	N/A
263	04-018	04	GBM	4	1	Initial	2023/6/8	59	Female	51	1·54	70	1	1	1

No.	Patient ID	Centre ID [a]	Integrated Diagnosis	CNS5 WHO Grade	Radiographic Gross Total Resection	Disease Stage (Initial/Recurrence)	Baseline Visit Date	Age at Baseline	Sex	Weight (kg)	Height (m)	V2-KPS	V2-PRO	V2b-PRO	V2-QLQ-C30
264	04-019	04	GBM	4	1	Recurrence	2023/6/2	59	Male	74	1·7	70	1	1	1
265	04-020	04	GBM	4	1	Recurrence	2023/6/2	50	Male	88	1·73	70	1	1	1
266	04-024	04	A	4	1	Recurrence	2023/9/28	45	Female	64	1·55	60	1	1	1
267	04-025	04	GBM	4	1	Initial	2023/9/28	58	Male	70	1·71	40	1	1	1
268	04-026	04	GBM	4	0	Initial	2023/10/13	56	Female	57	1·63	40	N/A	1	1
269	04-027	04	GBM	4	1	Initial	2023/10/8	56	Female	72	1·68	90	1	1	1
270	04-028	04	GBM	4	1	Initial	2023/10/19	69	Male	67.5	1·68	80	1	1	1
271	05-002	05	GBM	4	1	Initial	2023/5/17	60	Female	40	1·5	90	N/A	1	N/A
272	05-003	05	O	3	0	Initial	2023/6/2	58	Male	50	1·65	90	1	1	1
273	05-004	05	GBM	4	1	Initial	2023/5/18	45	Male	70	1·72	90	1	1	1
274	05-011	05	O	2	1	Initial	2023/6/11	36	Male	70	1·75	90	1	1	1
275	05-018	05	GBM	4	1	Initial	2023/8/18	56	Male	60	1·65	90	1	1	1
276	05-020	05	A	3	0	Initial	2023/7/7	57	Female	40	1·5	90	1	1	1
277	07-004	07	A	2	0	Initial	2023/4/27	25	Female	55	1·55	90	1	1	1
278	07-005	07	A	2	1	Initial	2023/5/17	46	Female	65	1·67	90	1	1	1
279	07-006	07	O	2	1	Initial	2023/5/18	40	Male	74.2	1·78	90	1	1	1
280	07-008	07	GBM	4	1	Initial	2023/11/29	57	Female	57.2	1·57	90	1	1	1
281	07-009	07	A	3	1	Initial	2023/11/29	31	Male	71.2	1·64	90	N/A	1	N/A
282	07-010	07	GBM	4	0	Initial	2023/12/4	71	Male	70.2	1·76	90	1	1	1
283	07-011	07	O	2	1	Initial	2023/12/4	34	Female	47.6	1·59	90	N/A	1	N/A
284	07-018	07	GBM	4	1	Initial	2024/3/14	63	Male	76.2	1·75	80	1	1	1
285	07-019	07	GBM	4	1	Recurrence	2024/12/3	29	Male	84.4	1·68	60	1	1	1

No.	Patient ID	Centre ID [a]	Integrated Diagnosis	CNS5 WHO Grade	Radiographic Gross Total Resection	Disease Stage (Initial/Recurrence)	Baseline Visit Date	Age at Baseline	Sex	Weight (kg)	Height (m)	V2-KPS	V2-PRO	V2b-PRO	V2-QLQ-C30
286	08-001	08	GBM	4	1	Recurrence	2023/8/1	45	Male	52	1·6	80	1	1	1
287	08-002	08	GBM	4	1	Recurrence	2023/8/25	69	Male	74.5	1·7	60	1	1	1
288	08-003	08	GBM	4	1	Recurrence	2024/1/18	32	Female	65	1·74	50	1	N/A	1
289	08-004	08	GBM	4	1	Initial	2024/3/4	61	Male	56	1·7	70	1	1	1
290	09-001	09	GBM	4	1	Initial	2023/3/19	50	Male	50	1·7	80	1	1	1
291	09-003	09	GBM	4	0	Recurrence	2023/2/27	70	Female	55	1·6	50	1	1	1
292	09-004	09	GBM	4	1	Initial	2023/4/15	46	Male	70	1·6	70	1	1	1
293	09-006	09	GBM	4	1	Initial	2023/3/5	59	Male	70	1·65	80	1	1	1
294	09-007	09	GBM	4	1	Initial	2022/11/25	57	Female	67.5	1·67	60	1	1	1
295	09-015	09	GBM	4	1	Initial	2022/11/9	64	Male	65	1·7	80	1	1	1
296	09-016	09	GBM	4	1	Initial	2022/11/12	66	Male	65	1·62	60	1	1	1
297	09-017	09	GBM	4	1	Initial	2023/4/10	51	Male	70	1·7	60	1	1	1
298	09-019	09	GBM	4	1	Initial	2022/12/14	35	Male	67	1·68	70	1	1	1
299	09-020	09	GBM	4	1	Recurrence	2022/12/6	59	Male	70	1·75	70	1	1	1
300	09-022	09	GBM	4	0	Initial	2022/12/8	56	Male	65	1·70	50	1	1	1
301	09-023	09	GBM	4	1	Initial	2023/1/15	60	Female	77	1·65	60	1	1	1
302	09-024	09	GBM	4	1	Initial	2022/12/14	43	Male	91	1·69	60	1	1	1
303	09-025	09	GBM	4	1	Initial	2023/2/27	53	Female	65	1·65	50	1	1	1
304	09-027	09	GBM	4	1	Initial	2022/12/14	52	Male	71	1·75	50	1	1	1
305	09-028	09	GBM	4	1	Initial	2022/12/15	60	Male	80	1·78	50	1	1	1
306	09-029	09	GBM	4	1	Initial	2022/12/17	57	Male	78	1·75	60	1	N/A	1
307	09-030	09	GBM	4	1	Initial	2023/7/12	59	Male	70	1·75	80	1	1	1

No.	Patient ID	Centre ID [a]	Integrated Diagnosis	CNS5 WHO Grade	Radiographic Gross Total Resection	Disease Stage (Initial/Recurrence)	Baseline Visit Date	Age at Baseline	Sex	Weight (kg)	Height (m)	V2-KPS	V2-PRO	V2b-PRO	V2-QLQ-C30
308	09-031	09	GBM	4	1	Initial	2022/11/14	56	Male	86	1·81	60	1	1	1
309	09-038	09	O	2	1	Initial	2023/3/5	24	Male	85	1·7	70	1	1	1
310	09-039	09	GBM	4	1	Initial	2023/4/16	67	Male	70	1·70	70	1	1	1
311	09-040	09	GBM	4	1	Initial	2023/4/16	65	Male	70	1·80	70	1	1	1
312	09-041	09	GBM	4	1	Initial	2022/11/14	61	Female	61	1·52	80	1	1	1
313	09-042	09	GBM	4	1	Initial	2023/4/17	67	Female	55	1·60	70	1	1	1
314	09-043	09	GBM	4	1	Initial	2023/4/17	53	Male	55	1·70	70	1	1	1
315	09-099	09	GBM	4	1	Initial	2023/2/18	37	Female	55	1·65	70	1	1	1
316	09-100	09	O	3	1	Initial	2023/2/18	43	Female	56	1·57	80	1	1	1
317	09-101	09	GBM	4	0	Initial	2023/5/15	70	Female	70	1·65	80	1	1	1
318	09-102	09	GBM	4	0	Initial	2023/5/15	71	Female	68	1·65	70	1	1	1
319	09-103	09	GBM	4	1	Initial	2023/5/15	74	Male	69	1·70	70	1	1	1
320	09-104	09	GBM	4	0	Initial	2023/5/7	51	Male	76	1·65	70	1	N/A	1
321	09-105	09	GBM	4	1	Initial	2023/5/7	60	Male	76	1·70	70	1	1	1
322	09-106	09	GBM	4	1	Initial	2023/5/7	57	Male	80	1·73	80	1	N/A	1
323	09-107	09	GBM	4	0	Initial	2023/4/17	73	Male	68	1·65	80	1	1	1
324	09-109	09	GBM	4	1	Initial	2023/4/9	70	Male	86	1·74	70	1	1	1
325	09-110	09	GBM	4	1	Initial	2023/4/13	57	Male	84	1·75	80	1	1	1
326	09-112	09	GBM	4	0	Initial	2024/2/4	71	Female	65	1·60	80	1	1	1
327	09-113	09	GBM	4	1	Initial	2024/1/8	35	Male	70	1·65	80	1	1	1
328	09-114	09	GBM	4	1	Initial	2024/1/8	27	Female	65	1·71	80	1	1	1
329	09-115	09	GBM	4	0	Initial	2024/2/4	40	Male	65	1·70	80	1	N/A	1

No.	Patient ID	Centre ID [a]	Integrated Diagnosis	CNS5 WHO Grade	Radiographic Gross Total Resection	Disease Stage (Initial/Recurrence)	Baseline Visit Date	Age at Baseline	Sex	Weight (kg)	Height (m)	V2-KPS	V2-PRO	V2b-PRO	V2-QLQ-C30
330	09-117	09	GBM	4	1	Initial	2024/2/16	67	Male	70	1·70	80	1	1	1
331	09-119	09	GBM	4	1	Initial	2024/4/8	66	Male	80	1·70	80	1	1	1
332	09-121	09	GBM	4	1	Initial	2024/4/6	63	Female	65	1·60	80	1	1	1
333	09-122	09	GBM	4	1	Initial	2024/4/8	51	Male	75	1·75	80	1	N/A	1
334	09-124	09	GBM	4	0	Initial	2023/7/1	65	Male	76	1·74	90	1	1	1
335	09-125	09	GBM	4	1	Initial	2023/6/29	60	Female	75	1·58	90	1	1	1
336	09-126	09	GBM	4	1	Initial	2023/6/22	61	Male	70	1·70	80	1	1	1
337	09-127	09	GBM	4	0	Initial	2023/6/10	52	Male	70	1·70	80	1	1	1
338	09-136	09	GBM	4	1	Initial	2023/7/2	55	Male	76	1·75	70	1	1	1
339	09-137	09	GBM	4	0	Initial	2024/1/27	61	Male	76	1·7	80	1	1	1
340	09-147	09	GBM	4	1	Initial	2023/5/9	28	Female	68	1·68	80	1	1	1
341	09-149	09	GBM	4	1	Initial	2023/5/9	59	Female	56	1·60	70	1	1	1
342	09-150	09	GBM	4	0	Initial	2023/5/9	74	Male	70	1·75	80	1	1	1
343	09-300	09	GBM	4	1	Initial	2023/12/17	76	Male	70	1·65	80	1	N/A	1
344	09-301	09	GBM	4	1	Initial	2023/12/17	46	Male	65	1·80	80	1	N/A	1
345	09-401	09	GBM	4	0	Initial	2024/1/23	53	Male	70	1·70	80	1	1	1
346	09-402	09	GBM	4	0	Initial	2024/1/30	52	Male	70	1·50	80	1	1	1
347	09-403	09	GBM	4	1	Initial	2024/1/30	49	Female	65	1·65	80	1	1	1
348	09-404	09	GBM	4	1	Initial	2024/2/4	49	Female	60	1·60	80	1	1	1
349	09-405	09	GBM	4	0	Initial	2024/2/18	56	Female	60	1·60	90	1	1	1
350	09-406	09	GBM	4	1	Initial	2024/2/18	57	Female	65	1·61	80	1	1	1
351	09-407	09	GBM	4	1	Initial	2024/2/18	57	Female	62	1·68	80	1	1	1

No.	Patient ID	Centre ID [a]	Integrated Diagnosis	CNS5 WHO Grade	Radiographic Gross Total Resection	Disease Stage (Initial/Recurrence)	Baseline Visit Date	Age at Baseline	Sex	Weight (kg)	Height (m)	V2-KPS	V2-PRO	V2b-PRO	V2-QLQ-C30
352	09-408	09	GBM	4	0	Initial	2024/2/18	37	Female	67	1·56	90	1	1	1
353	09-409	09	GBM	4	0	Initial	2024/2/19	51	Male	75	1·72	80	1	1	1
354	09-410	09	GBM	4	1	Initial	2024/3/9	55	Male	70	1·7	80	1	1	1
355	09-411	09	GBM	4	1	Initial	2024/3/9	56	Male	60	1·70	80	1	1	1
356	09-412	09	GBM	4	1	Initial	2024/4/15	56	Male	80	1·65	90	1	1	1
357	09-413	09	GBM	4	1	Initial	2024/4/15	57	Male	80	1·75	80	1	1	1
358	09-414	09	GBM	4	0	Initial	2024/4/15	58	Male	80	1·58	70	1	1	1
359	09-415	09	GBM	4	1	Initial	2024/4/15	60	Male	87	1·80	90	1	1	1
360	09-416	09	GBM	4	1	Initial	2024/4/15	57	Male	75	1·74	90	1	1	1
361	09-417	09	GBM	4	1	Initial	2024/4/15	56	Male	80	1·70	80	1	1	1
362	09-418	09	GBM	4	0	Initial	2024/4/15	55	Female	56	1·56	90	1	1	1
363	09-419	09	GBM	4	1	Initial	2024/4/15	54	Female	60	1·60	90	1	1	1
364	09-420	09	GBM	4	1	Initial	2024/4/15	54	Female	65	1·70	80	1	1	1
365	09-421	09	GBM	4	0	Initial	2024/4/28	57	Female	68	1·65	80	1	1	1
366	09-422	09	GBM	4	1	Initial	2024/4/28	53	Female	70	1·65	90	1	1	1
367	09-423	09	GBM	4	1	Initial	2024/4/28	51	Female	70	1·65	80	1	1	1
368	09-424	09	GBM	4	0	Initial	2024/4/28	58	Male	80	1·75	80	1	1	1
369	09-500	09	GBM	4	1	Initial	2024/4/27	53	Male	75	1·75	70	1	N/A	1
370	09-501	09	GBM	4	1	Initial	2024/5/2	67	Male	70	1·75	80	1	1	1
371	09-502	09	GBM	4	1	Initial	2024/6/1	53	Male	70	1·75	80	1	N/A	1
372	10-001	10	O	2	0	Initial	2023/3/24	44	Female	50	1·58	90	1	1	1
373	10-002	10	GBM	4	1	Initial	2023/3/26	48	Male	68	1·67	100	1	1	1

No.	Patient ID	Centre ID [a]	Integrated Diagnosis	CNS5 WHO Grade	Radiographic Gross Total Resection	Disease Stage (Initial/Recurrence)	Baseline Visit Date	Age at Baseline	Sex	Weight (kg)	Height (m)	V2-KPS	V2-PRO	V2b-PRO	V2-QLQ-C30
374	10-003	10	A	3	1	Initial	2023/4/14	40	Male	62	1·7	90	1	1	1
375	10-004	10	O	2	0	Initial	2023/4/28	33	Male	65	1·78	90	1	1	1
376	10-005	10	A	2	0	Initial	2023/6/19	36	Male	105	1·81	90	1	1	1
377	10-006	10	GBM	4	1	Initial	2023/6/19	65	Male	70	1·8	70	1	1	1
378	10-007	10	GBM	4	1	Initial	2023/6/20	57	Male	60	1·68	80	1	1	1
379	10-008	10	GBM	4	1	Initial	2023/8/19	56	Male	75	1·76	80	1	1	1
380	10-009	10	GBM	4	1	Initial	2023/9/9	67	Female	56	1·58	80	1	1	1
381	10-010	10	A	4	1	Recurrence	2023/9/13	52	Female	58	1·6	60	1	1	1
382	10-011	10	O	2	0	Initial	2023/10/19	50	Male	85	1·72	90	1	1	1
383	12-003	12	GBM	4	1	Initial	2023/2/14	44	Female	51	1·58	80	1	1	1
384	12-004	12	GBM	4	0	Initial	2023/3/1	50	Male	65	1·73	80	1	1	1
385	12-005	12	O	2	1	Initial	2023/3/1	33	Male	62	1·69	90	1	1	1
386	12-006	12	A	3	0	Initial	2023/3/10	48	Female	55	1·58	80	1	1	1
387	12-007	12	A	4	0	Initial	2023/3/14	42	Male	65	1·7	70	1	1	1
388	12-008	12	GBM	4	0	Initial	2023/3/20	61	Female	60	1·57	70	1	1	1
389	12-009	12	GBM	4	0	Initial	2023/3/20	41	Female	58	1·6	80	1	1	1
390	12-010	12	GBM	4	1	Initial	2023/3/20	39	Male	68	1·7	80	1	1	1
391	12-011	12	NOS	3	0	Initial	2023/3/20	58	Male	64	1·67	70	1	1	1
392	12-012	12	NOS	4	0	Initial	2023/3/20	19	Male	63	1·7	80	1	1	1
393	12-013	12	GBM	4	0	Initial	2023/3/28	57	Female	58	1·58	50	1	1	1
394	12-014	12	GBM	4	0	Initial	2023/3/28	68	Male	69	1·71	80	1	1	1
395	12-015	12	GBM	4	0	Initial	2023/4/3	59	Male	70	1·74	70	1	1	1

No.	Patient ID	Centre ID [a]	Integrated Diagnosis	CNS5 WHO Grade	Radiographic Gross Total Resection	Disease Stage (Initial/Recurrence)	Baseline Visit Date	Age at Baseline	Sex	Weight (kg)	Height (m)	V2-KPS	V2-PRO	V2b-PRO	V2-QLQ-C30
396	12-016	12	GBM	4	1	Initial	2023/4/10	59	Female	53	1·55	50	1	1	1
397	12-017	12	NOS	4	0	Initial	2023/4/10	21	Male	59	1·68	90	1	1	1
398	12-018	12	O	2	0	Initial	2023/4/10	44	Female	58	1·58	50	1	1	1
399	12-019	12	A	2	0	Initial	2023/4/10	32	Male	64	1·72	70	1	1	1
400	12-020	12	GBM	4	1	Initial	2023/4/17	64	Female	65	1·58	70	1	1	1
401	12-021	12	GBM	4	0	Initial	2023/5/4	48	Male	65	1·7	70	1	1	1
402	12-022	12	A	4	0	Initial	2023/5/4	41	Male	68	1·74	70	1	1	1
403	12-023	12	GBM	4	1	Initial	2023/5/4	60	Female	58	1·56	70	1	1	1
404	12-024	12	A	4	0	Recurrence	2023/5/6	49	Male	67	1·72	80	1	1	1
405	12-025	12	A	4	1	Initial	2023/5/6	34	Female	56	1·58	90	1	1	1
406	12-026	12	GBM	4	1	Initial	2023/5/6	30	Female	57	1·6	90	1	1	1
407	12-027	12	GBM	4	0	Initial	2023/5/6	68	Male	65	1·68	70	1	1	1
408	12-028	12	GBM	4	0	Initial	2023/5/23	69	Female	53	1·57	70	1	1	1
409	12-029	12	GBM	4	0	Initial	2023/6/3	65	Female	53	1·57	80	1	1	1
410	12-030	12	GBM	4	0	Initial	2023/6/8	53	Male	64	1·72	70	1	1	1
411	12-031	12	GBM	4	0	Initial	2023/6/19	62	Male	62	1·68	60	1	1	1
412	12-033	12	GBM	4	0	Initial	2024/2/2	57	Male	58.6	1·6	90	1	1	1
413	12-034	12	GBM	4	1	Initial	2024/2/6	23	Male	75	1·85	40	1	1	1
414	12-035	12	A	3	1	Initial	2024/2/23	43	Female	49.7	1·63	80	1	1	1
415	12-036	12	A	2	0	Initial	2024/3/2	31	Female	61.2	1·57	70	1	1	1
416	12-037	12	GBM	4	1	Initial	2024/3/1	61	Female	55	1·56	60	1	N/A	N/A
417	12-038	12	O	3	1	Initial	2024/3/25	35	Male	93.46	1·72	100	1	1	1

No.	Patient ID	Centre ID [a]	Integrated Diagnosis	CNS5 WHO Grade	Radiographic Gross Total Resection	Disease Stage (Initial/Recurrence)	Baseline Visit Date	Age at Baseline	Sex	Weight (kg)	Height (m)	V2-KPS	V2-PRO	V2b-PRO	V2-QLQ-C30
418	12-039	12	O	2	1	Initial	2024/3/25	48	Female	56.8	1·55	80	1	1	1
419	12-040	12	A	2	0	Initial	2024/4/8	45	Male	60.2	1·74	80	N/A	N/A	N/A
420	13-001	13	O	2	0	Initial	2022/11/4	60	Female	55	1·58	90	1	1	1
421	13-002	13	GBM	4	1	Initial	2022/11/13	57	Male	78	1·8	50	1	1	1
422	13-003	13	GBM	4	1	Initial	2022/11/13	37	Female	100	1·6	60	1	1	1
423	13-004	13	A	4	1	Initial	2022/12/6	35	Female	58	1·52	50	1	1	1
424	13-006	13	GBM	4	1	Initial	2023/3/14	48	Male	61	1·64	40	1	1	1
425	13-007	13	GBM	4	1	Initial	2023/3/14	60	Male	54	1·70	40	1	1	1
426	13-008	13	A	3	1	Initial	2023/4/5	45	Male	60	1·68	90	1	1	1
427	13-009	13	GBM	4	1	Recurrence	2023/4/7	55	Female	50	1·5	40	1	1	1
428	13-010	13	GBM	4	1	Initial	2023/4/12	31	Male	79	1·8	50	1	1	1
429	13-011	13	A	3	1	Initial	2023/4/18	37	Female	60	1·59	50	1	1	1
430	13-012	13	NOS	3	1	Initial	2023/4/28	70	Male	49	1·65	50	1	1	1
431	13-013	13	GBM	4	1	Initial	2023/5/15	50	Male	53	1·7	80	1	1	1
432	13-014	13	A	4	1	Initial	2023/5/16	56	Male	70	1·75	60	1	1	1
433	13-015	13	A	3	0	Initial	2023/6/9	36	Male	65	1·75	80	1	1	1
434	13-016	13	O	3	1	Initial	2023/6/16	39	Male	70	1·7	90	1	1	1
435	13-017	13	O	2	0	Initial	2024/3/20	30	Female	53	1·58	80	1	1	1
436	13-018	13	GBM	4	1	Initial	2024/5/17	34	Male	71	1·62	90	1	1	1
437	13-019	13	GBM	4	0	Initial	2024/6/12	39	Female	70	1·58	60	1	1	1
438	13-020	13	A	4	0	Recurrence	2024/7/8	27	Male	72	1·78	40	1	1	1
439	13-021	13	GBM	4	1	Initial	2024/8/18	55	Male	70	1·61	90	1	1	1

No.	Patient ID	Centre ID [a]	Integrated Diagnosis	CNS5 WHO Grade	Radiographic Gross Total Resection	Disease Stage (Initial/Recurrence)	Baseline Visit Date	Age at Baseline	Sex	Weight (kg)	Height (m)	V2-KPS	V2-PRO	V2b-PRO	V2-QLQ-C30
440	13-022	13	O	2	1	Initial	2024/11/5	36	Male	80	1·7	80	1	1	1
441	15-001	15	O	3	1	Recurrence	2023/5/10	56	Male	60	1·73	90	1	N/A	N/A
442	15-003	15	A	3	1	Initial	2023/5/24	32	Male	65	1·75	50	1	1	1
443	15-004	15	A	4	1	Initial	2023/5/29	36	Male	70	1·76	70	1	1	1
444	15-005	15	GBM	4	1	Initial	2023/5/30	53	Male	75	1·73	70	1	1	1
445	15-007	15	GBM	4	1	Initial	2023/6/15	50	Female	80	1·6	70	1	1	1
446	15-009	15	GBM	4	1	Recurrence	2024/3/20	62	Male	70	1·7	50	N/A	1	N/A
447	16-001	16	GBM	4	1	Initial	2023/5/24	49	Female	45	1·5	80	1	1	1
448	16-002	16	GBM	4	1	Initial	2023/7/10	53	Male	78	1·78	40	1	1	1
449	16-003	16	GBM	4	1	Initial	2023/8/1	60	Female	47	1·56	90	1	1	1
450	16-004	16	GBM	4	1	Initial	2023/9/7	50	Male	66	1·62	90	1	1	1

Abbreviations and coding: GBM, Glioblastoma; A, Astrocytoma; O, Oligodendroglioma; NOS, Not Otherwise Specified. Binary variables are coded as 1 = Yes/Received and 0 = No/Not received. Questionnaire completion variables are coded as 1 = completed; N/A = not completed or not applicable (e.g., missed visit).

[a] **Centre ID corresponds to participating institutions:** 01 = Huashan Hospital, Fudan University; 02 = Beijing Tiantan Hospital, Capital Medical University; 03 = Xuanwu Hospital, Capital Medical University; 04 = Sun Yat-Sen University Cancer Centre; 05 = Xijing Hospital, Fourth Military Medical University; 07 = Shanghai Proton and Heavy Ion Centre, Fudan University Cancer Hospital; 08 = The Second Affiliated Hospital, School of Medicine, Zhejiang University; 09 = The First Affiliated Hospital of Nanjing Medical University; 10 = Tongji Hospital, Tongji Medical College, Huazhong University of Science and Technology; 12 = Guangdong Sanjiu Brain Hospital; 13 = The First Affiliated Hospital of Kunming Medical University; 15 = Changhai Hospital, Naval Medical University (Second Military Medical University); 16 = Shanghai East Hospital, Tongji University School of Medicine.

Table S6b. VERONICA Patient Baseline and Follow-up Data Summary (Lower Part).

No.	Patient ID	Centre ID [a]	V3-KPS	V3-QLQ-C30	V3-GIC	V3-PRO	V4-KPS	V4-PRO	V4-QLQ-C30	V4-GIC	Eligible for Analysis	Eligible for Test-Retest Reliability	Eligible for Convergent Validity	Eligible for Known-Groups Validity	Eligible for Responsiveness Analysis	Radiotherapy Received	Chemotherapy Received	Stupp Regimen Received	TTFields Treatment
1	01-001	01	90	1	1	1	90	1	1	1	1	1	1	1	1	1	1	1	0
2	01-002	01	50	1	1	1	60	1	1	1	1	1	1	1	1	0	0	0	0
3	01-003	01	90	1	1	1	90	1	1	1	1	1	1	1	1	1	1	0	0
4	01-004	01	60	1	1	1	60	1	1	1	1	1	1	1	1	1	1	1	0
5	01-005	01	80	N/A	N/A	N/A	40	1	1	1	1	1	1	1	0	1	1	0	0
6	01-006	01	90	N/A	N/A	N/A	80	1	1	1	1	1	1	1	0	1	0	0	0
7	01-007	01	90	N/A	1	1	90	1	1	1	1	1	1	1	1	1	0	0	0
8	01-008	01	70	N/A	N/A	N/A	70	1	1	1	1	1	1	1	0	1	0	0	0
9	01-011	01	N/A	N/A	N/A	N/A	N/A	N/A	N/A	N/A	0	0	0	0	0	1	0	0	0
10	01-012	01	90	1	1	1	90	1	1	1	1	1	1	1	1	1	1	1	1
11	01-013	01	60	1	1	1	N/A	N/A	N/A	N/A	1	1	0	0	0	1	1	1	0
12	01-014	01	N/A	N/A	N/A	N/A	70	N/A	N/A	N/A	1	1	0	0	0	1	1	1	0
13	01-015	01	60	N/A	N/A	N/A	60	1	1	1	1	1	1	1	0	1	1	1	1
14	01-016	01	100	1	1	1	100	1	1	1	1	1	1	1	1	1	1	1	0
15	01-017	01	50	1	1	1	50	1	1	1	1	1	1	1	1	1	1	1	0
16	01-019	01	60	1	1	1	60	1	1	1	1	1	1	1	1	N/A	N/A	N/A	N/A
17	01-020	01	80	1	1	1	80	1	1	1	1	1	1	1	1	N/A	N/A	N/A	N/A
18	01-021	01	90	1	1	1	90	1	1	1	1	1	1	1	1	1	1	1	0
19	01-022	01	70	1	1	1	70	1	1	1	1	1	1	1	1	1	1	1	0

No.	Patient ID	Centre ID [a]	V3-KPS	V3-QLQ-C30	V3-GIC	V3-PRO	V4-KPS	V4-PRO	V4-QLQ-C30	V4-GIC	Eligible for Analysis	Eligible for Test-Retest Reliability	Eligible for Convergent Validity	Eligible for Known-Groups Validity	Eligible for Responsiveness Analysis	Radiotherapy Received	Chemotherapy Received	Stupp Regimen Received	TTFields Treatment
20	01-023	01	90	1	1	1	90	1	1	1	1	1	1	1	1	1	1	1	0
21	01-024	01	N/A	N/A	N/A	N/A	60	1	1	1	1	1	1	1	0	1	1	1	0
22	01-025	01	50	1	1	1	50	1	1	1	1	1	1	1	1	1	0	0	0
23	01-027	01	80	1	1	1	80	1	1	1	1	1	1	1	1	1	1	0	0
24	01-028	01	N/A	N/A	N/A	N/A	N/A	N/A	N/A	N/A	0	0	0	0	0	N/A	N/A	N/A	N/A
25	01-029	01	60	1	1	1	60	1	1	1	1	1	1	1	1	1	1	0	0
26	01-030	01	80	1	1	1	80	1	1	1	1	1	1	1	1	1	1	1	1
27	01-031	01	50	1	1	1	50	1	1	1	1	1	1	1	1	1	1	1	0
28	01-032	01	N/A	N/A	N/A	N/A	70	1	1	1	1	0	1	1	0	N/A	N/A	N/A	N/A
29	01-033	01	70	1	1	1	60	1	1	1	1	1	1	1	1	1	1	1	0
30	01-034	01	100	1	1	1	100	1	1	1	1	1	1	1	1	0	0	0	0
31	01-035	01	N/A	N/A	N/A	N/A	90	1	1	1	1	1	1	1	0	1	1	0	0
32	01-036	01	50	1	1	1	50	1	1	1	1	1	1	1	1	N/A	N/A	N/A	N/A
33	01-037	01	N/A	N/A	N/A	N/A	30	N/A	N/A	N/A	1	1	0	0	0	1	1	0	0
34	01-038	01	60	1	1	1	60	1	1	1	1	0	1	1	1	1	1	0	1
35	01-039	01	N/A	N/A	N/A	N/A	70	N/A	N/A	N/A	1	1	0	0	0	1	1	0	0
36	01-041	01	60	1	1	1	60	N/A	N/A	N/A	0	0	0	0	0	1	1	0	0
37	01-042	01	70	1	1	1	70	1	1	1	1	0	1	1	1	1	1	1	0
38	01-044	01	70	1	1	1	70	1	1	1	1	1	1	1	1	1	1	1	0
39	01-045	01	70	1	1	1	N/A	N/A	N/A	N/A	0	0	0	0	0	1	1	0	0

No.	Patient ID	Centre ID [a]	V3-KPS	V3-QLQ-C30	V3-GIC	V3-PRO	V4-KPS	V4-PRO	V4-QLQ-C30	V4-GIC	Eligible for Analysis	Eligible for Test-Retest Reliability	Eligible for Convergent Validity	Eligible for Known-Groups Validity	Eligible for Responsiveness Analysis	Radiotherapy Received	Chemotherapy Received	Stupp Regimen Received	TTFields Treatment
40	01-046	01	70	1	1	1	60	1	1	1	1	0	1	1	1	0	1	0	0
41	01-047	01	50	1	1	1	50	1	1	1	1	0	1	1	1	1	1	0	1
42	01-049	01	N/A	N/A	N/A	N/A	80	N/A	N/A	N/A	1	1	0	0	0	1	1	1	0
43	01-050	01	60	1	1	1	50	1	1	1	1	0	1	1	1	1	1	1	0
44	01-052	01	80	1	1	1	60	1	1	1	1	1	1	1	1	1	1	1	1
45	01-053	01	90	1	1	1	80	1	1	1	1	1	1	1	1	1	0	0	0
46	01-054	01	60	1	1	1	50	1	1	1	1	0	1	1	1	1	1	0	0
47	01-055	01	90	1	1	1	80	1	1	1	1	1	1	1	1	1	1	0	0
48	01-057	01	N/A	N/A	N/A	N/A	N/A	N/A	N/A	N/A	0	0	0	0	0	N/A	N/A	N/A	N/A
49	01-058	01	70	1	1	1	70	1	1	1	1	1	1	1	1	1	1	0	0
50	01-059	01	60	1	1	1	80	1	1	1	1	1	1	1	1	0	0	0	0
51	01-060	01	90	1	1	1	100	1	1	1	1	0	1	1	1	0	1	0	0
52	01-061	01	60	1	1	1	60	1	1	1	1	1	1	1	1	1	1	1	0
53	01-062	01	60	1	1	1	50	1	1	1	1	0	1	1	1	1	0	0	0
54	01-063	01	60	1	1	1	60	1	1	1	1	1	1	1	1	1	0	0	0
55	01-064	01	60	1	1	1	60	1	1	1	1	1	1	1	1	N/A	N/A	N/A	N/A
56	01-065	01	30	1	1	1	50	1	1	1	1	1	1	1	1	1	0	0	0
57	01-066	01	40	1	1	1	40	1	1	1	1	1	1	1	1	1	1	1	0
58	01-067	01	70	1	1	1	70	1	1	1	1	1	1	1	1	N/A	N/A	N/A	N/A
59	01-068	01	90	1	1	1	80	1	1	1	1	1	1	1	1	N/A	N/A	N/A	N/A

No.	Patient ID	Centre ID [a]	V3-KPS	V3-QLQ-C30	V3-GIC	V3-PRO	V4-KPS	V4-PRO	V4-QLQ-C30	V4-GIC	Eligible for Analysis	Eligible for Test-Retest Reliability	Eligible for Convergent Validity	Eligible for Known-Groups Validity	Eligible for Responsiveness Analysis	Radiotherapy Received	Chemotherapy Received	Stupp Regimen Received	TTFields Treatment
60	01-069	01	50	1	1	1	80	1	1	1	1	1	1	1	1	1	1	0	0
61	01-070	01	60	1	1	1	100	1	1	1	1	1	1	1	1	0	0	0	0
62	01-071	01	60	1	1	1	60	1	1	1	1	1	1	1	1	1	0	0	0
63	01-072	01	60	1	1	1	80	1	1	1	1	1	1	1	1	0	1	0	0
64	01-074	01	90	1	1	1	N/A	N/A	N/A	N/A	1	1	0	0	0	1	0	0	0
65	01-075	01	60	1	1	1	90	1	1	1	1	1	1	1	1	0	1	0	0
66	01-076	01	60	1	1	1	80	1	1	1	1	1	1	1	1	N/A	N/A	N/A	N/A
67	01-077	01	60	1	1	1	60	1	1	1	1	1	1	1	1	N/A	N/A	N/A	N/A
68	01-078	01	50	1	1	1	50	1	1	1	1	1	1	1	1	1	1	0	0
69	01-079	01	50	1	1	1	50	1	1	1	1	1	1	1	1	N/A	N/A	N/A	N/A
70	01-080	01	N/A	N/A	N/A	N/A	N/A	N/A	N/A	N/A	1	1	0	0	0	N/A	N/A	N/A	N/A
71	01-081	01	90	1	1	1	80	1	1	1	1	1	1	1	1	0	0	0	0
72	01-082	01	60	1	1	1	60	1	1	1	1	1	1	1	1	1	0	0	0
73	01-083	01	N/A	1	1	1	N/A	N/A	N/A	N/A	1	1	0	0	0	N/A	N/A	N/A	N/A
74	01-084	01	N/A	N/A	N/A	N/A	100	1	1	1	1	1	1	1	0	N/A	N/A	N/A	N/A
75	01-085	01	N/A	N/A	N/A	N/A	N/A	N/A	N/A	N/A	1	1	0	0	0	N/A	N/A	N/A	N/A
76	01-086	01	N/A	N/A	N/A	N/A	N/A	N/A	N/A	N/A	0	0	0	0	0	N/A	N/A	N/A	N/A
77	01-087	01	60	N/A	N/A	N/A	60	1	1	1	1	1	1	1	0	N/A	N/A	N/A	N/A
78	01-088	01	60	1	1	1	60	1	1	1	1	1	1	1	1	N/A	N/A	N/A	N/A
79	01-089	01	N/A	N/A	N/A	N/A	60	1	1	1	1	0	1	1	0	N/A	N/A	N/A	N/A

No.	Patient ID	Centre ID [a]	V3-KPS	V3-QLQ-C30	V3-GIC	V3-PRO	V4-KPS	V4-PRO	V4-QLQ-C30	V4-GIC	Eligible for Analysis	Eligible for Test-Retest Reliability	Eligible for Convergent Validity	Eligible for Known-Groups Validity	Eligible for Responsiveness Analysis	Radiotherapy Received	Chemotherapy Received	Stupp Regimen Received	TTFields Treatment
80	01-090	01	50	1	1	1	50	1	1	1	1	1	1	1	1	1	1	0	0
81	01-091	01	70	1	1	1	N/A	N/A	N/A	N/A	1	1	0	0	0	N/A	N/A	N/A	N/A
82	01-092	01	100	1	1	1	100	1	1	1	1	1	1	1	1	N/A	N/A	N/A	N/A
83	01-094	01	N/A	N/A	N/A	N/A	N/A	N/A	N/A	N/A	1	1	0	0	0	N/A	N/A	N/A	N/A
84	01-095	01	N/A	1	1	1	N/A	N/A	N/A	N/A	1	1	0	0	0	N/A	N/A	N/A	N/A
85	01-096	01	N/A	N/A	N/A	N/A	N/A	N/A	N/A	N/A	0	0	0	0	0	N/A	N/A	N/A	N/A
86	01-097	01	N/A	N/A	N/A	N/A	100	1	1	1	1	1	1	1	0	N/A	N/A	N/A	N/A
87	01-098	01	N/A	N/A	N/A	N/A	N/A	N/A	N/A	N/A	0	0	0	0	0	N/A	N/A	N/A	N/A
88	01-099	01	50	1	1	1	40	1	1	1	1	1	1	1	1	N/A	N/A	N/A	N/A
89	01-100	01	N/A	N/A	N/A	N/A	N/A	N/A	N/A	N/A	0	0	0	0	0	N/A	N/A	N/A	N/A
90	01-101	01	60	1	1	1	60	1	1	1	1	0	1	1	1	N/A	N/A	N/A	N/A
91	01-102	01	40	1	1	1	40	1	1	1	1	0	1	1	1	N/A	N/A	N/A	N/A
92	01-103	01	90	1	1	1	90	1	1	1	1	1	1	1	1	0	1	0	0
93	01-106	01	60	1	1	1	60	1	1	1	1	1	1	1	1	1	1	1	1
94	01-107	01	90	1	1	1	90	1	1	1	1	1	1	1	1	1	1	1	0
95	01-108	01	60	1	1	1	80	1	1	1	1	1	1	1	1	1	1	1	0
96	01-109	01	70	1	1	1	80	1	1	1	1	1	1	1	1	1	1	1	0
97	01-110	01	50	1	1	1	70	1	1	1	1	1	1	1	1	1	1	1	1
98	01-111	01	90	1	1	1	90	1	1	1	1	1	1	1	1	1	0	0	0
99	01-112	01	90	1	1	1	90	1	1	1	1	1	1	1	1	1	1	0	0

No.	Patient ID	Centre ID [a]	V3-KPS	V3-QLQ-C30	V3-GIC	V3-PRO	V4-KPS	V4-PRO	V4-QLQ-C30	V4-GIC	Eligible for Analysis	Eligible for Test-Retest Reliability	Eligible for Convergent Validity	Eligible for Known-Groups Validity	Eligible for Responsiveness Analysis	Radiotherapy Received	Chemotherapy Received	Stupp Regimen Received	TTFields Treatment
100	01-113	01	90	1	1	1	90	1	1	1	1	1	1	1	1	1	1	1	1
101	01-116	01	90	1	1	1	90	1	1	1	1	1	1	1	1	N/A	N/A	N/A	N/A
102	01-117	01	80	1	1	1	80	1	1	1	1	1	1	1	1	1	1	0	1
103	01-118	01	90	1	1	1	60	1	1	1	1	1	1	1	1	1	1	0	1
104	01-119	01	70	1	1	1	90	1	1	1	1	1	1	1	1	N/A	N/A	N/A	N/A
105	01-120	01	70	1	1	1	70	1	1	1	1	1	1	1	1	1	1	1	1
106	01-121	01	60	1	1	1	60	1	1	1	1	1	1	1	1	1	1	0	0
107	01-122	01	70	1	1	1	90	1	1	1	1	1	1	1	1	0	1	0	1
108	01-123	01	90	1	1	1	100	1	1	1	1	1	1	1	1	N/A	N/A	N/A	N/A
109	01-124	01	70	1	1	1	80	1	1	1	1	1	1	1	1	N/A	N/A	N/A	N/A
110	01-125	01	70	1	1	1	90	1	1	1	1	1	1	1	1	N/A	N/A	N/A	N/A
111	01-126	01	40	1	1	1	60	1	1	1	1	0	1	1	1	N/A	N/A	N/A	N/A
112	01-127	01	90	1	1	1	100	1	1	1	1	1	1	1	1	N/A	N/A	N/A	N/A
113	01-128	01	90	1	1	1	100	1	1	1	1	1	1	1	1	1	1	0	0
114	01-130	01	70	1	1	1	90	1	1	1	1	1	1	1	1	1	1	0	0
115	01-131	01	60	1	1	1	60	1	1	1	1	1	1	1	1	1	1	0	0
116	01-132	01	70	1	1	1	60	1	1	1	1	1	1	1	1	1	1	1	0
117	01-133	01	60	1	1	1	60	1	1	1	1	1	1	1	1	1	1	1	1
118	01-134	01	40	1	1	1	60	1	1	1	1	1	1	1	1	1	1	1	0
119	01-135	01	90	1	1	1	100	1	1	1	1	1	1	1	1	1	1	0	0

No.	Patient ID	Centre ID [a]	V3-KPS	V3-QLQ-C30	V3-GIC	V3-PRO	V4-KPS	V4-PRO	V4-QLQ-C30	V4-GIC	Eligible for Analysis	Eligible for Test-Retest Reliability	Eligible for Convergent Validity	Eligible for Known-Groups Validity	Eligible for Responsiveness Analysis	Radiotherapy Received	Chemotherapy Received	Stupp Regimen Received	TTFields Treatment
120	01-136	01	70	1	1	1	90	1	1	1	1	1	1	1	1	0	1	0	0
121	01-137	01	70	1	1	1	80	1	1	1	1	1	1	1	1	0	1	0	0
122	01-138	01	50	1	1	1	90	1	1	1	1	1	1	1	1	1	1	1	0
123	01-139	01	60	1	1	1	40	1	1	1	1	1	1	1	1	0	1	0	0
124	01-140	01	90	1	1	1	80	1	1	1	1	1	1	1	1	0	1	0	0
125	01-141	01	90	1	1	1	90	1	1	1	1	1	1	1	1	1	1	1	0
126	01-142	01	60	1	1	1	80	1	1	1	1	1	1	1	1	1	0	0	0
127	01-143	01	70	1	1	1	100	1	1	1	1	1	1	1	1	1	1	1	0
128	01-144	01	90	1	1	1	100	1	1	1	1	1	1	1	1	1	0	0	0
129	01-145	01	80	1	1	1	90	1	1	1	1	1	1	1	1	N/A	N/A	N/A	N/A
130	01-146	01	60	1	1	1	90	1	1	1	1	1	1	1	1	0	1	0	0
131	01-147	01	90	1	1	1	90	1	1	1	1	1	1	1	1	1	0	0	0
132	01-148	01	70	1	1	1	70	1	1	1	1	1	1	1	1	1	1	1	0
133	01-149	01	90	1	1	1	90	1	1	1	1	1	1	1	1	0	1	0	0
134	01-150	01	N/A	N/A	N/A	N/A	N/A	N/A	N/A	N/A	0	0	0	0	0	N/A	N/A	N/A	N/A
135	01-152	01	40	1	1	1	40	1	1	1	1	1	1	1	1	1	1	1	0
136	01-153	01	90	1	1	1	100	1	1	1	1	1	1	1	1	0	1	0	0
137	01-154	01	70	N/A	1	1	40	1	1	1	1	1	1	1	1	N/A	N/A	N/A	N/A
138	01-155	01	70	1	1	1	80	1	1	1	1	1	1	1	1	1	1	1	0
139	01-156	01	60	1	1	1	60	1	1	1	1	1	1	1	1	1	1	1	0

No.	Patient ID	Centre ID [a]	V3-KPS	V3-QLQ-C30	V3-GIC	V3-PRO	V4-KPS	V4-PRO	V4-QLQ-C30	V4-GIC	Eligible for Analysis	Eligible for Test-Retest Reliability	Eligible for Convergent Validity	Eligible for Known-Groups Validity	Eligible for Responsiveness Analysis	Radiotherapy Received	Chemotherapy Received	Stupp Regimen Received	TTFields Treatment
140	01-157	01	70	1	1	1	70	1	1	1	1	1	1	1	1	1	1	0	0
141	01-158	01	80	1	1	1	70	1	1	1	1	1	1	1	1	1	1	0	0
142	01-159	01	70	1	1	1	70	1	1	1	1	1	1	1	1	N/A	N/A	N/A	N/A
143	01-160	01	70	1	1	1	80	1	1	1	1	1	1	1	1	1	1	1	0
144	01-161	01	90	1	1	1	90	1	1	1	1	1	1	1	1	1	1	1	0
145	01-162	01	90	1	1	1	90	1	1	1	1	1	1	1	1	N/A	N/A	N/A	N/A
146	01-163	01	90	1	1	1	90	1	1	1	1	1	1	1	1	1	1	1	0
147	01-164	01	80	1	1	1	50	1	1	1	1	1	1	1	1	N/A	N/A	N/A	N/A
148	01-165	01	90	1	1	1	90	1	1	1	1	1	1	1	1	1	0	0	0
149	01-167	01	80	1	1	1	80	1	1	1	1	1	1	1	1	1	1	1	0
150	01-168	01	90	1	1	1	90	1	1	1	1	1	1	1	1	1	1	1	0
151	01-169	01	90	1	1	1	90	1	1	1	1	1	1	1	1	1	1	0	0
152	01-170	01	60	1	1	1	80	1	1	1	1	1	1	1	1	1	1	1	0
153	01-171	01	70	1	1	1	90	1	1	1	1	1	1	1	1	1	1	0	0
154	01-172	01	N/A	N/A	N/A	N/A	N/A	N/A	N/A	N/A	1	1	0	0	0	N/A	N/A	N/A	N/A
155	01-173	01	90	1	1	1	90	1	1	1	1	1	1	1	1	1	1	0	0
156	01-174	01	60	1	1	1	50	1	1	1	1	1	1	1	1	0	1	0	0
157	01-175	01	90	1	1	1	90	1	1	1	1	1	1	1	1	0	1	0	0
158	01-176	01	N/A	N/A	N/A	N/A	N/A	N/A	N/A	N/A	1	1	0	0	0	N/A	N/A	N/A	N/A
159	01-177	01	90	1	1	1	90	1	1	1	1	1	1	1	1	1	0	0	0

No.	Patient ID	Centre ID [a]	V3-KPS	V3-QLQ-C30	V3-GIC	V3-PRO	V4-KPS	V4-PRO	V4-QLQ-C30	V4-GIC	Eligible for Analysis	Eligible for Test-Retest Reliability	Eligible for Convergent Validity	Eligible for Known-Groups Validity	Eligible for Responsiveness Analysis	Radiotherapy Received	Chemotherapy Received	Stupp Regimen Received	TTFields Treatment
160	01-178	01	100	1	1	1	90	1	1	1	1	1	1	1	1	1	1	1	0
161	01-179	01	100	1	1	1	90	1	1	1	1	1	1	1	1	1	0	0	0
162	01-180	01	100	1	1	1	90	1	1	1	1	1	1	1	1	N/A	N/A	N/A	N/A
163	01-181	01	50	1	1	1	50	1	1	1	1	1	1	1	1	1	0	0	0
164	01-182	01	70	1	1	1	80	1	1	1	1	1	1	1	1	1	1	1	0
165	01-183	01	90	1	1	1	80	1	1	1	1	1	1	1	1	0	1	0	0
166	01-184	01	80	1	1	1	80	1	1	1	1	1	1	1	1	1	1	0	0
167	01-185	01	90	1	1	1	90	1	1	1	1	1	1	1	1	N/A	N/A	N/A	N/A
168	01-186	01	80	1	1	1	90	1	1	1	1	1	1	1	1	1	1	1	0
169	01-187	01	80	1	1	1	90	1	1	1	1	1	1	1	1	1	1	1	0
170	01-188	01	90	1	1	1	80	1	1	1	1	1	1	1	1	N/A	N/A	N/A	N/A
171	01-189	01	90	1	1	1	50	1	1	1	1	1	1	1	1	1	1	0	0
172	01-190	01	70	1	1	1	N/A	N/A	N/A	N/A	1	1	0	0	0	0	1	0	0
173	01-191	01	70	1	1	1	60	1	1	1	1	1	1	1	1	N/A	N/A	N/A	N/A
174	01-192	01	90	1	1	1	100	1	1	1	1	1	1	1	1	N/A	N/A	N/A	N/A
175	01-193	01	80	1	1	1	90	1	1	1	1	1	1	1	1	0	1	0	0
176	01-194	01	60	1	1	1	N/A	N/A	N/A	N/A	1	1	0	0	0	1	1	0	0
177	01-195	01	80	1	1	1	40	1	1	1	1	1	1	1	1	0	1	0	0
178	01-196	01	80	1	1	1	90	1	1	1	1	1	1	1	1	N/A	N/A	N/A	N/A
179	01-197	01	100	1	1	1	90	1	1	1	1	1	1	1	1	N/A	N/A	N/A	N/A

No.	Patient ID	Centre ID [a]	V3-KPS	V3-QLQ-C30	V3-GIC	V3-PRO	V4-KPS	V4-PRO	V4-QLQ-C30	V4-GIC	Eligible for Analysis	Eligible for Test-Retest Reliability	Eligible for Convergent Validity	Eligible for Known-Groups Validity	Eligible for Responsiveness Analysis	Radiotherapy Received	Chemotherapy Received	Stupp Regimen Received	TTFields Treatment
180	01-198	01	60	1	1	1	80	1	1	1	1	1	1	1	1	0	1	0	0
181	01-199	01	70	1	1	1	N/A	N/A	N/A	N/A	1	1	0	0	0	N/A	N/A	N/A	N/A
182	01-200	01	N/A	N/A	N/A	N/A	N/A	N/A	N/A	N/A	1	1	0	0	0	N/A	N/A	N/A	N/A
183	01-201	01	100	1	1	1	100	1	1	1	1	1	1	1	1	1	1	0	0
184	01-202	01	90	1	1	1	N/A	N/A	N/A	N/A	1	1	0	0	0	0	1	0	1
185	01-203	01	60	1	1	1	80	1	1	1	1	1	1	1	1	0	1	0	0
186	01-204	01	50	1	1	1	80	1	1	1	1	1	1	1	1	1	1	0	0
187	01-205	01	100	1	1	1	100	1	1	1	1	1	1	1	1	0	1	0	0
188	01-206	01	60	1	1	1	50	1	1	1	1	1	1	1	1	N/A	N/A	N/A	N/A
189	01-208	01	100	1	1	1	100	1	1	1	1	1	1	1	1	N/A	N/A	N/A	N/A
190	01-209	01	60	1	1	1	70	1	1	1	1	1	1	1	1	0	1	0	0
191	01-210	01	90	1	1	1	100	1	1	1	1	1	1	1	1	N/A	N/A	N/A	N/A
192	01-211	01	90	1	1	1	100	1	1	1	1	1	1	1	1	1	1	0	0
193	01-212	01	90	1	1	1	100	1	1	1	1	1	1	1	1	N/A	N/A	N/A	N/A
194	01-213	01	40	1	1	1	70	1	1	1	1	1	1	1	1	N/A	N/A	N/A	N/A
195	01-214	01	80	1	1	1	100	1	1	1	1	1	1	1	1	N/A	N/A	N/A	N/A
196	01-215	01	80	1	1	1	100	1	1	1	1	1	1	1	1	0	1	0	0
197	01-216	01	40	1	1	1	40	N/A	N/A	N/A	1	1	0	0	0	1	1	0	0
198	01-217	01	80	1	1	1	90	1	1	1	1	1	1	1	1	N/A	N/A	N/A	N/A
199	01-218	01	80	1	1	1	90	1	1	1	1	1	1	1	1	N/A	N/A	N/A	N/A

No.	Patient ID	Centre ID [a]	V3-KPS	V3-QLQ-C30	V3-GIC	V3-PRO	V4-KPS	V4-PRO	V4-QLQ-C30	V4-GIC	Eligible for Analysis	Eligible for Test-Retest Reliability	Eligible for Convergent Validity	Eligible for Known-Groups Validity	Eligible for Responsiveness Analysis	Radiotherapy Received	Chemotherapy Received	Stupp Regimen Received	TTFields Treatment
200	01-219	01	90	1	1	1	100	1	1	1	1	1	1	1	1	N/A	N/A	N/A	N/A
201	01-220	01	90	1	1	1	100	1	1	1	1	1	1	1	1	N/A	N/A	N/A	N/A
202	01-221	01	90	1	1	1	100	1	1	1	1	1	1	1	1	N/A	N/A	N/A	N/A
203	01-222	01	90	1	1	1	90	1	1	1	1	1	1	1	1	N/A	N/A	N/A	N/A
204	01-223	01	90	1	1	1	100	1	1	1	1	1	1	1	1	N/A	N/A	N/A	N/A
205	01-224	01	N/A	N/A	N/A	N/A	N/A	N/A	N/A	N/A	1	1	0	0	0	N/A	N/A	N/A	N/A
206	01-225	01	90	1	1	1	100	1	1	1	1	1	1	1	1	N/A	N/A	N/A	N/A
207	01-226	01	90	1	1	1	100	1	1	1	1	1	1	1	1	1	1	1	0
208	01-227	01	50	1	1	1	80	1	1	1	1	1	1	1	1	N/A	N/A	N/A	N/A
209	01-228	01	60	1	1	1	60	1	1	1	1	1	1	1	1	N/A	N/A	N/A	N/A
210	01-229	01	80	1	1	1	70	1	1	1	1	1	1	1	1	N/A	N/A	N/A	N/A
211	01-230	01	90	1	1	1	100	1	1	1	1	1	1	1	1	N/A	N/A	N/A	N/A
212	01-231	01	80	1	1	1	60	1	1	1	1	1	1	1	1	N/A	N/A	N/A	N/A
213	01-232	01	70	1	1	1	70	1	1	1	1	1	1	1	1	N/A	N/A	N/A	N/A
214	01-233	01	90	1	1	1	90	1	1	1	1	1	1	1	1	1	1	0	0
215	01-234	01	90	1	1	1	100	1	1	1	1	1	1	1	1	N/A	N/A	N/A	N/A
216	01-235	01	100	1	1	1	100	1	1	1	1	1	1	1	1	N/A	N/A	N/A	N/A
217	01-236	01	90	1	1	1	90	1	1	1	1	1	1	1	1	N/A	N/A	N/A	N/A
218	01-237	01	N/A	N/A	N/A	N/A	100	1	1	1	1	1	1	1	0	N/A	N/A	N/A	N/A
219	01-238	01	90	1	1	1	100	1	1	1	1	1	1	1	1	1	1	0	0

No.	Patient ID	Centre ID [a]	V3-KPS	V3-QLQ-C30	V3-GIC	V3-PRO	V4-KPS	V4-PRO	V4-QLQ-C30	V4-GIC	Eligible for Analysis	Eligible for Test-Retest Reliability	Eligible for Convergent Validity	Eligible for Known-Groups Validity	Eligible for Responsiveness Analysis	Radiotherapy Received	Chemotherapy Received	Stupp Regimen Received	TTFields Treatment
220	01-239	01	90	1	1	1	N/A	N/A	N/A	N/A	1	1	0	0	0	N/A	N/A	N/A	N/A
221	01-240	01	100	1	1	1	100	1	1	1	1	1	1	1	1	N/A	N/A	N/A	N/A
222	01-241	01	80	1	1	1	80	1	1	1	1	1	1	1	1	0	1	0	0
223	02-002	02	90	1	1	1	90	1	1	1	1	1	1	1	1	N/A	N/A	N/A	N/A
224	02-003	02	90	1	1	1	90	1	1	1	1	1	1	1	1	N/A	N/A	N/A	N/A
225	02-004	02	90	1	1	1	90	1	1	1	1	1	1	1	1	N/A	N/A	N/A	N/A
226	02-005	02	90	1	1	1	90	1	1	1	1	1	1	1	1	N/A	N/A	N/A	N/A
227	02-006	02	90	1	1	1	90	1	1	1	1	1	1	1	1	N/A	N/A	N/A	N/A
228	02-008	02	100	1	1	1	100	1	1	1	1	1	1	1	1	N/A	N/A	N/A	N/A
229	02-009	02	90	1	1	1	90	1	1	1	1	1	1	1	1	N/A	N/A	N/A	N/A
230	02-010	02	N/A	1	1	1	90	1	1	1	1	1	1	1	1	N/A	N/A	N/A	N/A
231	02-011	02	90	1	1	1	90	1	1	1	1	1	1	1	1	N/A	N/A	N/A	N/A
232	02-012	02	90	1	1	1	90	1	1	1	1	1	1	1	1	N/A	N/A	N/A	N/A
233	02-013	02	N/A	1	1	1	90	1	1	1	1	1	1	1	1	N/A	N/A	N/A	N/A
234	03-001	03	80	1	1	1	90	1	1	1	1	1	1	1	1	1	1	0	0
235	03-002	03	90	1	1	1	90	1	1	1	1	1	1	1	1	1	1	0	0
236	03-003	03	100	1	1	1	100	1	1	1	1	1	1	1	1	1	0	0	0
237	03-004	03	100	1	1	1	100	1	1	1	1	1	1	1	1	1	1	0	0
238	03-005	03	100	1	1	1	100	1	1	1	1	1	1	1	1	1	0	0	0
239	03-006	03	100	1	1	1	100	1	1	1	1	1	1	1	1	1	1	1	0

No.	Patient ID	Centre ID [a]	V3-KPS	V3-QLQ-C30	V3-GIC	V3-PRO	V4-KPS	V4-PRO	V4-QLQ-C30	V4-GIC	Eligible for Analysis	Eligible for Test-Retest Reliability	Eligible for Convergent Validity	Eligible for Known-Groups Validity	Eligible for Responsiveness Analysis	Radiotherapy Received	Chemotherapy Received	Stupp Regimen Received	TTFields Treatment
240	03-007	03	100	1	1	1	100	1	1	1	1	1	1	1	1	0	0	0	0
241	03-008	03	50	1	1	1	10	N/A	N/A	N/A	1	1	0	0	0	1	1	0	0
242	03-009	03	100	1	1	1	100	1	1	1	1	1	1	1	1	1	0	0	0
243	03-010	03	100	1	1	1	100	1	1	1	1	1	1	1	1	1	0	0	0
244	03-011	03	100	1	1	1	100	1	1	1	1	1	1	1	1	N/A	N/A	N/A	N/A
245	03-012	03	N/A	N/A	N/A	N/A	N/A	N/A	N/A	N/A	1	1	0	0	0	N/A	N/A	N/A	N/A
246	03-013	03	100	N/A	N/A	N/A	N/A	N/A	N/A	N/A	0	0	0	0	0	1	0	0	0
247	03-014	03	N/A	N/A	N/A	N/A	N/A	N/A	N/A	N/A	0	0	0	0	0	N/A	N/A	N/A	N/A
248	03-015	03	100	1	1	1	100	1	1	1	1	1	1	1	1	N/A	N/A	N/A	N/A
249	03-016	03	N/A	1	1	1	90	1	1	1	1	1	1	1	1	N/A	N/A	N/A	N/A
250	04-001	04	N/A	N/A	N/A	N/A	N/A	N/A	N/A	N/A	0	0	0	0	0	0	0	0	0
251	04-002	04	N/A	N/A	N/A	N/A	N/A	N/A	N/A	N/A	1	1	0	0	0	0	0	0	0
252	04-003	04	N/A	1	1	1	N/A	N/A	N/A	1	1	1	0	0	0	0	0	0	0
253	04-004	04	90	1	1	1	N/A	N/A	1	1	0	0	0	0	0	1	0	0	0
254	04-005	04	N/A	N/A	N/A	N/A	N/A	N/A	N/A	N/A	0	0	0	0	0	0	0	0	0
255	04-006	04	N/A	N/A	N/A	N/A	N/A	N/A	N/A	N/A	0	0	0	0	0	N/A	N/A	N/A	N/A
256	04-007	04	N/A	N/A	N/A	N/A	N/A	N/A	N/A	N/A	0	0	0	0	0	N/A	N/A	N/A	N/A
257	04-011	04	N/A	N/A	N/A	N/A	N/A	N/A	N/A	N/A	1	1	0	0	0	N/A	N/A	N/A	N/A
258	04-013	04	90	1	1	1	90	1	1	1	1	1	1	1	1	1	1	0	0
259	04-014	04	90	1	1	1	90	1	1	1	1	0	1	1	1	0	1	0	0

No.	Patient ID	Centre ID [a]	V3-KPS	V3-QLQ-C30	V3-GIC	V3-PRO	V4-KPS	V4-PRO	V4-QLQ-C30	V4-GIC	Eligible for Analysis	Eligible for Test-Retest Reliability	Eligible for Convergent Validity	Eligible for Known-Groups Validity	Eligible for Responsiveness Analysis	Radiotherapy Received	Chemotherapy Received	Stupp Regimen Received	TTFields Treatment
260	04-015	04	80	1	1	1	80	1	1	1	1	1	1	1	1	N/A	N/A	N/A	N/A
261	04-016	04	80	1	1	1	90	N/A	N/A	1	1	1	0	0	0	1	1	0	0
262	04-017	04	N/A	N/A	N/A	N/A	N/A	N/A	N/A	N/A	0	0	0	0	0	N/A	N/A	N/A	N/A
263	04-018	04	90	1	1	1	90	1	1	1	1	1	1	1	1	1	1	1	0
264	04-019	04	90	1	1	1	80	1	1	1	1	1	1	1	1	1	1	1	0
265	04-020	04	N/A	N/A	N/A	N/A	N/A	N/A	N/A	N/A	1	1	0	0	0	N/A	N/A	N/A	N/A
266	04-024	04	N/A	1	N/A	1	N/A	N/A	N/A	N/A	1	1	0	0	0	N/A	N/A	N/A	N/A
267	04-025	04	60	1	1	1	60	1	1	1	1	1	1	1	1	1	1	0	0
268	04-026	04	50	1	1	1	50	1	1	1	1	0	1	1	1	1	1	0	0
269	04-027	04	80	1	1	1	80	1	1	1	1	1	1	1	1	1	1	0	1
270	04-028	04	80	1	1	1	80	1	1	1	1	1	1	1	1	1	1	0	0
271	05-002	05	90	1	1	1	80	1	1	1	1	0	1	1	1	N/A	N/A	N/A	N/A
272	05-003	05	90	1	1	1	80	1	1	1	1	1	1	1	1	N/A	N/A	N/A	N/A
273	05-004	05	90	1	1	1	90	1	1	1	1	1	1	1	1	N/A	N/A	N/A	N/A
274	05-011	05	90	N/A	1	1	100	1	1	1	1	1	1	1	1	N/A	N/A	N/A	N/A
275	05-018	05	N/A	N/A	N/A	N/A	N/A	N/A	N/A	N/A	1	1	0	0	0	N/A	N/A	N/A	N/A
276	05-020	05	N/A	N/A	N/A	N/A	N/A	N/A	N/A	N/A	1	1	0	0	0	N/A	N/A	N/A	N/A
277	07-004	07	90	1	1	1	90	1	1	1	1	1	1	1	1	0	0	0	0
278	07-005	07	N/A	N/A	N/A	N/A	N/A	N/A	N/A	N/A	1	1	0	0	0	N/A	N/A	N/A	N/A
279	07-006	07	N/A	N/A	N/A	N/A	N/A	N/A	N/A	N/A	1	1	0	0	0	N/A	N/A	N/A	N/A

No.	Patient ID	Centre ID [a]	V3-KPS	V3-QLQ-C30	V3-GIC	V3-PRO	V4-KPS	V4-PRO	V4-QLQ-C30	V4-GIC	Eligible for Analysis	Eligible for Test-Retest Reliability	Eligible for Convergent Validity	Eligible for Known-Groups Validity	Eligible for Responsiveness Analysis	Radiotherapy Received	Chemotherapy Received	Stupp Regimen Received	TTFields Treatment
280	07-008	07	90	1	1	1	N/A	N/A	N/A	N/A	1	1	0	0	0	N/A	N/A	N/A	N/A
281	07-009	07	90	1	1	1	90	1	1	1	1	0	1	1	1	N/A	N/A	N/A	N/A
282	07-010	07	90	1	1	1	90	1	1	1	1	1	1	1	1	N/A	N/A	N/A	N/A
283	07-011	07	90	1	1	1	90	1	1	1	1	0	1	1	1	N/A	N/A	N/A	N/A
284	07-018	07	80	N/A	1	1	N/A	N/A	N/A	N/A	1	1	0	0	0	N/A	N/A	N/A	N/A
285	07-019	07	N/A	N/A	N/A	N/A	N/A	N/A	N/A	N/A	1	1	0	0	0	N/A	N/A	N/A	N/A
286	08-001	08	N/A	1	1	1	N/A	N/A	N/A	N/A	1	1	0	0	0	1	1	0	0
287	08-002	08	N/A	1	1	1	N/A	N/A	N/A	N/A	1	1	0	0	0	N/A	N/A	N/A	N/A
288	08-003	08	80	1	1	1	90	1	1	1	1	0	1	1	1	1	0	0	0
289	08-004	08	60	1	1	1	60	1	1	1	1	1	1	1	1	N/A	N/A	N/A	N/A
290	09-001	09	80	1	1	1	80	1	1	1	1	1	1	1	1	1	1	1	0
291	09-003	09	80	1	1	1	80	1	1	1	1	1	1	1	1	1	1	0	0
292	09-004	09	80	1	1	1	90	1	1	1	1	1	1	1	1	1	1	0	0
293	09-006	09	N/A	N/A	N/A	N/A	N/A	N/A	N/A	N/A	1	1	0	0	0	N/A	N/A	N/A	N/A
294	09-007	09	50	1	1	1	60	1	1	1	1	1	1	1	1	1	1	0	0
295	09-015	09	70	1	1	1	70	1	1	1	1	1	1	1	1	1	1	0	0
296	09-016	09	70	1	1	1	70	1	1	1	1	1	1	1	1	1	1	0	0
297	09-017	09	90	1	1	1	N/A	N/A	N/A	N/A	1	1	0	0	0	1	1	0	0
298	09-019	09	80	1	1	1	80	1	1	1	1	1	1	1	1	1	1	1	0
299	09-020	09	80	1	1	1	80	1	1	1	1	1	1	1	1	1	1	0	0

No.	Patient ID	Centre ID [a]	V3-KPS	V3-QLQ-C30	V3-GIC	V3-PRO	V4-KPS	V4-PRO	V4-QLQ-C30	V4-GIC	Eligible for Analysis	Eligible for Test-Retest Reliability	Eligible for Convergent Validity	Eligible for Known-Groups Validity	Eligible for Responsiveness Analysis	Radiotherapy Received	Chemotherapy Received	Stupp Regimen Received	TTFields Treatment
300	09-022	09	80	1	1	1	80	1	1	1	1	1	1	1	1	1	1	0	0
301	09-023	09	60	1	1	1	70	1	1	1	1	1	1	1	1	1	1	1	0
302	09-024	09	80	1	1	1	80	1	1	1	1	1	1	1	1	1	1	0	0
303	09-025	09	80	1	1	1	80	1	1	1	1	1	1	1	1	1	1	1	0
304	09-027	09	N/A	N/A	N/A	N/A	N/A	N/A	N/A	N/A	1	1	0	0	0	N/A	N/A	N/A	N/A
305	09-028	09	80	1	1	1	80	1	1	1	1	1	1	1	1	1	1	0	0
306	09-029	09	70	1	1	1	50	1	1	1	1	0	1	1	1	1	1	0	1
307	09-030	09	80	1	1	1	90	1	1	1	1	1	1	1	1	1	1	1	0
308	09-031	09	80	1	1	1	80	1	1	1	1	1	1	1	1	1	1	0	0
309	09-038	09	80	1	1	1	90	1	1	1	1	1	1	1	1	1	1	0	1
310	09-039	09	70	1	1	1	80	1	1	1	1	1	1	1	1	1	1	1	0
311	09-040	09	N/A	N/A	N/A	N/A	N/A	N/A	N/A	N/A	1	1	0	0	0	N/A	N/A	N/A	N/A
312	09-041	09	70	1	1	1	80	1	1	1	1	1	1	1	1	1	1	1	1
313	09-042	09	70	1	1	1	80	1	1	1	1	1	1	1	1	1	1	0	1
314	09-043	09	70	1	1	1	80	1	1	1	1	1	1	1	1	1	1	1	1
315	09-099	09	80	1	1	1	80	1	1	1	1	1	1	1	1	1	1	1	1
316	09-100	09	70	1	1	1	80	1	1	1	1	1	1	1	1	1	1	1	0
317	09-101	09	90	1	1	1	90	1	1	1	1	1	1	1	1	1	1	0	0
318	09-102	09	80	1	1	1	N/A	N/A	N/A	N/A	1	1	0	0	0	1	1	0	0
319	09-103	09	90	1	1	1	80	1	1	1	1	1	1	1	1	1	1	0	0

No.	Patient ID	Centre ID [a]	V3-KPS	V3-QLQ-C30	V3-GIC	V3-PRO	V4-KPS	V4-PRO	V4-QLQ-C30	V4-GIC	Eligible for Analysis	Eligible for Test-Retest Reliability	Eligible for Convergent Validity	Eligible for Known-Groups Validity	Eligible for Responsiveness Analysis	Radiotherapy Received	Chemotherapy Received	Stupp Regimen Received	TTFields Treatment
320	09-104	09	80	1	1	1	90	1	1	1	1	0	1	1	1	1	1	1	0
321	09-105	09	80	1	1	1	80	1	1	1	1	1	1	1	1	1	1	0	0
322	09-106	09	80	1	1	1	N/A	N/A	N/A	N/A	0	0	0	0	0	1	1	0	1
323	09-107	09	80	1	1	1	80	1	1	1	1	1	1	1	1	1	1	1	0
324	09-109	09	90	1	1	1	90	1	1	1	1	1	1	1	1	1	1	0	0
325	09-110	09	90	1	1	1	90	1	1	1	1	1	1	1	1	1	1	0	0
326	09-112	09	90	1	1	1	90	1	1	1	1	1	1	1	1	1	1	1	0
327	09-113	09	N/A	N/A	N/A	N/A	N/A	N/A	N/A	N/A	1	1	0	0	0	N/A	N/A	N/A	N/A
328	09-114	09	80	1	1	1	80	1	1	1	1	1	1	1	1	1	1	0	0
329	09-115	09	N/A	N/A	N/A	N/A	N/A	N/A	N/A	N/A	0	0	0	0	0	N/A	N/A	N/A	N/A
330	09-117	09	90	1	1	1	90	1	1	1	1	1	1	1	1	1	1	0	0
331	09-119	09	90	1	1	1	N/A	N/A	N/A	N/A	1	1	0	0	0	1	1	0	0
332	09-121	09	90	1	1	1	80	1	1	1	1	1	1	1	1	1	1	1	0
333	09-122	09	N/A	N/A	N/A	N/A	N/A	N/A	N/A	N/A	0	0	0	0	0	N/A	N/A	N/A	N/A
334	09-124	09	90	1	1	1	90	1	1	1	1	1	1	1	1	1	1	0	0
335	09-125	09	90	1	1	1	90	1	1	1	1	1	1	1	1	1	1	0	0
336	09-126	09	90	1	1	1	90	1	1	1	1	1	1	1	1	1	1	0	0
337	09-127	09	90	1	1	1	90	1	1	1	1	1	1	1	1	1	1	0	0
338	09-136	09	80	1	1	1	90	1	1	1	1	1	1	1	1	1	1	0	0
339	09-137	09	N/A	N/A	N/A	N/A	N/A	N/A	N/A	N/A	1	1	0	0	0	N/A	N/A	N/A	N/A

No.	Patient ID	Centre ID [a]	V3-KPS	V3-QLQ-C30	V3-GIC	V3-PRO	V4-KPS	V4-PRO	V4-QLQ-C30	V4-GIC	Eligible for Analysis	Eligible for Test-Retest Reliability	Eligible for Convergent Validity	Eligible for Known-Groups Validity	Eligible for Responsiveness Analysis	Radiotherapy Received	Chemotherapy Received	Stupp Regimen Received	TTFields Treatment
340	09-147	09	80	1	1	1	90	1	1	1	1	1	1	1	1	1	1	0	0
341	09-149	09	80	1	1	1	N/A	N/A	N/A	N/A	1	1	0	0	0	N/A	N/A	N/A	N/A
342	09-150	09	90	1	1	1	90	1	1	1	1	1	1	1	1	1	1	0	0
343	09-300	09	N/A	N/A	N/A	N/A	N/A	N/A	N/A	N/A	0	0	0	0	0	N/A	N/A	N/A	N/A
344	09-301	09	N/A	N/A	N/A	N/A	N/A	N/A	N/A	N/A	0	0	0	0	0	N/A	N/A	N/A	N/A
345	09-401	09	90	1	1	1	90	1	1	1	1	1	1	1	1	1	1	1	1
346	09-402	09	90	1	1	1	90	1	1	1	1	1	1	1	1	1	1	1	1
347	09-403	09	90	1	1	1	90	1	1	1	1	1	1	1	1	1	1	1	1
348	09-404	09	90	1	1	1	90	1	1	1	1	1	1	1	1	1	1	1	1
349	09-405	09	90	1	1	1	90	1	1	1	1	1	1	1	1	1	1	1	1
350	09-406	09	90	1	1	1	80	1	1	1	1	1	1	1	1	1	1	1	1
351	09-407	09	90	1	1	1	90	1	1	1	1	1	1	1	1	1	1	1	1
352	09-408	09	90	1	1	1	90	1	1	1	1	1	1	1	1	1	1	1	1
353	09-409	09	80	1	1	1	90	1	1	1	1	1	1	1	1	1	1	1	1
354	09-410	09	90	1	1	1	90	1	1	1	1	1	1	1	1	1	1	1	1
355	09-411	09	90	1	1	1	80	1	1	1	1	1	1	1	1	1	1	1	1
356	09-412	09	90	1	1	1	80	1	1	1	1	1	1	1	1	1	1	1	1
357	09-413	09	90	1	1	1	80	1	1	1	1	1	1	1	1	1	1	1	1
358	09-414	09	90	1	1	1	80	1	1	1	1	1	1	1	1	1	1	1	1
359	09-415	09	90	1	1	1	90	1	1	1	1	1	1	1	1	1	1	1	1

No.	Patient ID	Centre ID [a]	V3-KPS	V3-QLQ-C30	V3-GIC	V3-PRO	V4-KPS	V4-PRO	V4-QLQ-C30	V4-GIC	Eligible for Analysis	Eligible for Test-Retest Reliability	Eligible for Convergent Validity	Eligible for Known-Groups Validity	Eligible for Responsiveness Analysis	Radiotherapy Received	Chemotherapy Received	Stupp Regimen Received	TTFields Treatment
360	09-416	09	80	1	1	1	80	1	1	1	1	1	1	1	1	1	1	1	1
361	09-417	09	80	1	1	1	90	1	1	1	1	1	1	1	1	1	1	1	1
362	09-418	09	90	1	1	1	90	1	1	1	1	1	1	1	1	1	1	1	1
363	09-419	09	90	1	1	1	90	1	1	1	1	1	1	1	1	1	1	1	1
364	09-420	09	90	1	1	1	90	1	1	1	1	1	1	1	1	1	1	1	1
365	09-421	09	90	1	1	1	80	1	1	1	1	1	1	1	1	1	1	1	1
366	09-422	09	90	1	1	1	80	1	1	1	1	1	1	1	1	1	1	0	1
367	09-423	09	90	1	1	1	80	1	1	1	1	1	1	1	1	1	1	1	1
368	09-424	09	100	1	1	1	80	1	1	1	1	1	1	1	1	1	1	1	1
369	09-500	09	N/A	N/A	N/A	N/A	N/A	N/A	N/A	N/A	0	0	0	0	0	N/A	N/A	N/A	N/A
370	09-501	09	N/A	N/A	N/A	N/A	N/A	N/A	N/A	N/A	1	1	0	0	0	N/A	N/A	N/A	N/A
371	09-502	09	N/A	N/A	N/A	N/A	N/A	N/A	N/A	N/A	0	0	0	0	0	N/A	N/A	N/A	N/A
372	10-001	10	90	1	1	1	100	1	1	1	1	1	1	1	1	N/A	N/A	N/A	N/A
373	10-002	10	100	1	1	1	100	1	1	1	1	1	1	1	1	1	0	0	0
374	10-003	10	90	1	1	1	90	1	1	1	1	1	1	1	1	N/A	N/A	N/A	N/A
375	10-004	10	90	1	1	1	100	1	1	1	1	1	1	1	1	N/A	N/A	N/A	N/A
376	10-005	10	90	1	1	1	90	1	1	1	1	1	1	1	1	N/A	N/A	N/A	N/A
377	10-006	10	N/A	N/A	N/A	N/A	N/A	N/A	N/A	N/A	1	1	0	0	0	N/A	N/A	N/A	N/A
378	10-007	10	80	1	1	1	N/A	N/A	N/A	N/A	1	1	0	0	0	N/A	N/A	N/A	N/A
379	10-008	10	80	1	1	1	90	1	1	1	1	1	1	1	1	N/A	N/A	N/A	N/A

No.	Patient ID	Centre ID [a]	V3-KPS	V3-QLQ-C30	V3-GIC	V3-PRO	V4-KPS	V4-PRO	V4-QLQ-C30	V4-GIC	Eligible for Analysis	Eligible for Test-Retest Reliability	Eligible for Convergent Validity	Eligible for Known-Groups Validity	Eligible for Responsiveness Analysis	Radiotherapy Received	Chemotherapy Received	Stupp Regimen Received	TTFields Treatment
380	10-009	10	80	1	1	1	90	1	1	1	1	1	1	1	1	N/A	N/A	N/A	N/A
381	10-010	10	90	1	1	1	90	1	1	1	1	1	1	1	1	N/A	N/A	N/A	N/A
382	10-011	10	90	1	1	1	90	1	1	1	1	1	1	1	1	N/A	N/A	N/A	N/A
383	12-003	12	80	1	1	1	80	1	1	1	1	1	1	1	1	1	1	0	0
384	12-004	12	90	1	1	1	90	1	1	1	1	1	1	1	1	1	1	0	0
385	12-005	12	90	1	1	1	90	1	1	1	1	1	1	1	1	1	1	0	0
386	12-006	12	80	1	1	1	80	1	1	1	1	1	1	1	1	1	1	0	0
387	12-007	12	80	1	1	1	70	1	1	1	1	1	1	1	1	1	1	0	0
388	12-008	12	70	1	1	1	70	1	1	1	1	1	1	1	1	1	1	0	0
389	12-009	12	80	1	1	1	80	1	1	1	1	1	1	1	1	1	1	0	0
390	12-010	12	80	1	1	1	80	1	1	1	1	1	1	1	1	1	1	0	0
391	12-011	12	N/A	N/A	N/A	N/A	N/A	N/A	N/A	N/A	1	1	0	0	0	1	1	0	0
392	12-012	12	80	1	1	1	80	1	1	1	1	1	1	1	1	1	1	0	0
393	12-013	12	50	1	1	1	50	1	1	1	1	1	1	1	1	1	1	0	0
394	12-014	12	80	1	1	1	80	1	1	1	1	1	1	1	1	1	1	0	0
395	12-015	12	70	1	1	1	70	1	1	1	1	1	1	1	1	1	1	0	0
396	12-016	12	50	1	1	1	50	1	1	1	1	1	1	1	1	1	1	0	0
397	12-017	12	90	1	1	1	90	1	1	1	1	1	1	1	1	1	1	0	0
398	12-018	12	50	1	1	1	50	1	1	1	1	1	1	1	1	1	1	0	0
399	12-019	12	80	1	1	1	90	1	1	1	1	1	1	1	1	1	1	0	0

No.	Patient ID	Centre ID [a]	V3-KPS	V3-QLQ-C30	V3-GIC	V3-PRO	V4-KPS	V4-PRO	V4-QLQ-C30	V4-GIC	Eligible for Analysis	Eligible for Test-Retest Reliability	Eligible for Convergent Validity	Eligible for Known-Groups Validity	Eligible for Responsiveness Analysis	Radiotherapy Received	Chemotherapy Received	Stupp Regimen Received	TTFields Treatment
400	12-020	12	80	1	1	1	80	1	1	1	1	1	1	1	1	1	1	0	0
401	12-021	12	N/A	N/A	N/A	N/A	N/A	N/A	N/A	N/A	1	1	0	0	0	1	1	0	0
402	12-022	12	80	1	1	1	80	1	1	1	1	1	1	1	1	1	1	0	0
403	12-023	12	80	1	1	1	70	1	1	1	1	1	1	1	1	1	1	0	0
404	12-024	12	80	1	1	1	80	1	1	1	1	1	1	1	1	1	1	0	0
405	12-025	12	90	1	1	1	90	1	1	1	1	1	1	1	1	1	1	0	0
406	12-026	12	90	1	1	1	90	1	1	1	1	1	1	1	1	1	1	0	0
407	12-027	12	80	1	1	1	80	1	1	1	1	1	1	1	1	1	1	0	0
408	12-028	12	60	1	1	1	60	1	1	1	1	1	1	1	1	1	1	0	1
409	12-029	12	80	1	1	1	80	1	1	1	1	1	1	1	1	1	1	0	1
410	12-030	12	70	1	1	1	70	1	1	1	1	1	1	1	1	1	1	0	1
411	12-031	12	50	1	1	1	50	1	1	1	1	1	1	1	1	1	1	0	1
412	12-033	12	90	1	1	1	80	1	N/A	1	1	1	0	1	1	N/A	N/A	N/A	N/A
413	12-034	12	N/A	N/A	N/A	1	80	1	N/A	1	1	1	0	1	1	N/A	N/A	N/A	N/A
414	12-035	12	N/A	N/A	N/A	1	70	1	N/A	1	1	1	0	1	1	N/A	N/A	N/A	N/A
415	12-036	12	N/A	N/A	N/A	1	70	1	N/A	1	1	1	0	1	1	N/A	N/A	N/A	N/A
416	12-037	12	N/A	N/A	N/A	1	80	1	N/A	1	1	0	0	1	1	N/A	N/A	N/A	N/A
417	12-038	12	80	N/A	N/A	1	80	1	N/A	1	1	1	0	1	1	N/A	N/A	N/A	N/A
418	12-039	12	80	N/A	N/A	1	80	1	N/A	1	1	1	0	1	1	N/A	N/A	N/A	N/A
419	12-040	12	N/A	N/A	N/A	1	70	1	N/A	1	1	0	0	1	1	N/A	N/A	N/A	N/A

No.	Patient ID	Centre ID [a]	V3-KPS	V3-QLQ-C30	V3-GIC	V3-PRO	V4-KPS	V4-PRO	V4-QLQ-C30	V4-GIC	Eligible for Analysis	Eligible for Test-Retest Reliability	Eligible for Convergent Validity	Eligible for Known-Groups Validity	Eligible for Responsiveness Analysis	Radiotherapy Received	Chemotherapy Received	Stupp Regimen Received	TTFields Treatment
420	13-001	13	90	1	1	1	90	1	1	1	1	1	1	1	1	1	1	1	0
421	13-002	13	80	1	1	1	90	1	1	1	1	1	1	1	1	1	1	1	0
422	13-003	13	90	1	1	1	90	1	1	1	1	1	1	1	1	1	1	1	0
423	13-004	13	50	N/A	1	N/A	50	1	1	1	1	1	1	1	0	1	1	1	0
424	13-006	13	90	1	1	1	90	1	1	1	1	1	1	1	1	1	1	1	0
425	13-007	13	50	1	1	1	50	1	1	1	1	1	1	1	1	1	1	1	0
426	13-008	13	80	1	1	1	80	1	1	1	1	1	1	1	1	1	1	1	0
427	13-009	13	40	1	1	1	30	1	1	1	1	1	1	1	1	N/A	N/A	N/A	N/A
428	13-010	13	50	1	1	1	60	N/A	N/A	N/A	1	1	0	0	0	1	1	1	0
429	13-011	13	60	1	1	1	40	1	1	1	1	1	1	1	1	1	1	1	0
430	13-012	13	50	1	1	1	50	1	1	1	1	1	1	1	1	1	1	0	0
431	13-013	13	90	1	1	1	90	1	1	1	1	1	1	1	1	1	1	1	0
432	13-014	13	90	1	1	1	90	1	1	1	1	1	1	1	1	1	1	1	0
433	13-015	13	80	1	1	1	90	1	1	1	1	1	1	1	1	1	1	1	0
434	13-016	13	90	1	1	1	90	1	1	1	1	1	1	1	1	1	1	1	0
435	13-017	13	90	1	1	1	90	N/A	N/A	N/A	1	1	0	0	0	1	1	1	0
436	13-018	13	90	1	1	1	90	1	1	1	1	1	1	1	1	1	1	1	0
437	13-019	13	N/A	1	1	1	N/A	N/A	N/A	N/A	1	1	0	0	0	1	1	1	0
438	13-020	13	40	1	1	1	60	1	1	1	1	1	1	1	1	N/A	N/A	N/A	N/A
439	13-021	13	N/A	N/A	N/A	N/A	80	1	1	N/A	1	1	1	1	0	N/A	N/A	N/A	N/A

No.	Patient ID	Centre ID [a]	V3-KPS	V3-QLQ-C30	V3-GIC	V3-PRO	V4-KPS	V4-PRO	V4-QLQ-C30	V4-GIC	Eligible for Analysis	Eligible for Test-Retest Reliability	Eligible for Convergent Validity	Eligible for Known-Groups Validity	Eligible for Responsiveness Analysis	Radiotherapy Received	Chemotherapy Received	Stupp Regimen Received	TTFields Treatment
440	13-022	13	80	1	1	1	80	N/A	1	1	1	1	0	0	0	N/A	N/A	N/A	N/A
441	15-001	15	90	1	1	1	90	1	1	1	1	0	1	1	1	N/A	N/A	N/A	N/A
442	15-003	15	60	1	1	1	60	1	1	1	1	1	1	1	1	N/A	N/A	N/A	N/A
443	15-004	15	70	1	1	1	70	1	1	1	1	1	1	1	1	N/A	N/A	N/A	N/A
444	15-005	15	70	1	1	1	70	1	1	1	1	1	1	1	1	N/A	N/A	N/A	N/A
445	15-007	15	N/A	N/A	N/A	N/A	N/A	N/A	N/A	N/A	1	1	0	0	0	N/A	N/A	N/A	N/A
446	15-009	15	N/A	N/A	N/A	N/A	N/A	N/A	N/A	N/A	0	0	0	0	0	N/A	N/A	N/A	N/A
447	16-001	16	90	1	1	1	N/A	N/A	N/A	N/A	1	1	0	0	0	1	1	0	0
448	16-002	16	50	1	1	1	50	1	1	1	1	1	1	1	1	1	1	0	1
449	16-003	16	90	1	1	1	90	1	1	1	1	1	1	1	1	1	1	0	0
450	16-004	16	N/A	N/A	N/A	N/A	N/A	N/A	N/A	N/A	1	1	0	0	0	1	1	0	0

Abbreviations and coding: GBM, Glioblastoma; A, Astrocytoma; O, Oligodendroglioma; NOS, Not Otherwise Specified; TTFields, Tumor Treating Fields. Binary variables are coded as 1 = Yes/Received and 0 = No/Not received. Questionnaire completion variables are coded as 1 = completed; N/A = not completed or not applicable (e.g., missed visit). Eligibility variables indicate whether participants met prespecified criteria for each psychometric analysis (1 = eligible; 0 = not eligible).

[a] **Centre ID corresponds to participating institutions:** 01 = Huashan Hospital, Fudan University; 02 = Beijing Tiantan Hospital, Capital Medical University; 03 = Xuanwu Hospital, Capital Medical University; 04 = Sun Yat-Sen University Cancer Centre; 05 = Xijing Hospital, Fourth Military Medical University; 07 = Shanghai Proton and Heavy Ion Centre, Fudan University Cancer Hospital; 08 = The Second Affiliated Hospital, School of Medicine, Zhejiang University; 09 = The First Affiliated Hospital of Nanjing Medical University; 10 = Tongji Hospital, Tongji Medical College, Huazhong University of Science and Technology; 12 = Guangdong Sanjiu Brain Hospital; 13 = The First Affiliated Hospital of Kunming Medical University; 15 = Changhai Hospital, Naval Medical University (Second Military Medical University); 16 = Shanghai East Hospital, Tongji University School of Medicine.

Table S7. Test-Retest Reliability Analysis Results.

NO.	PRO-CTCAE Item	ICC	95%CI_Lower	95%CI_Upper	P
1a	Difficulty swallowing S	0·751701964	0·69778088	0·796002599	3·131E-41
2a	Taste changes S	0·640297988	0·562184112	0·704475008	7·96177E-24
3a	Decreased appetite S	0·74977907	0·695440405	0·794422783	7·92705E-41
3b	Decreased appetite I	0·780673833	0·73304436	0·81980539	6·1508E-48
4a	Nausea F	0·810044272	0·768792965	0·843935638	3·39827E-56
4b	Nausea S	0·833602746	0·797467461	0·863290875	3·18335E-64
5a	Vomiting F	0·816598875	0·776770983	0·849320787	2·75485E-58
5b	Vomiting S	0·854298425	0·822657471	0·880294089	1·20034E-72
6a	Constipation S	0·883496289	0·858196023	0·904282553	1·41483E-87
7a	Shortness of breath S	0·593914821	0·505728248	0·666367394	4·39576E-19
7b	Shortness of breath I	0·66380421	0·590795009	0·723787316	1·10839E-26
8a	Heart palpitations F	0·73717902	0·680104095	0·784070799	2·68918E-38
8b	Heart palpitations S	0·756019983	0·703036613	0·79955021	3·73419E-42
9a	Rash P	0·746742728	0·691744684	0·791928177	3·36197E-40
10a	Hair loss A	0·821108828	0·782260329	0·853026086	8·72019E-60
11a	Itching S	0·822504837	0·7839595	0·854173024	2·923E-60
12a	Hives P	0·695228862	0·629043924	0·749605269	4·32927E-31
13a	Bed/pressure sores P	0·750968992	0·696888735	0·795400403	4·46706E-41
14a	Radiation skin reaction S	0·720056566	0·659263281	0·770003284	3·80222E-35
15a	Numbness & tingling S	0·773993933	0·724913835	0·814317299	2·80324E-46
15b	Numbness & tingling I	0·758546059	0·706111258	0·801625591	1·04809E-42
16a	Dizziness S	0·826322191	0·788605841	0·857309296	1·38487E-61
16b	Dizziness I	0·788366358	0·742407415	0·826125436	6·09945E-50
17a	Blurred vision S	0·853675313	0·821899042	0·879782152	2·26663E-72
17b	Blurred vision I	0·841624572	0·807231329	0·869881469	2·57465E-67
18a	Flashing lights P	0·569294886	0·47576178	0·646140078	5·75662E-17
19a	Ringing in ears S	0·773934783	0·724841839	0·814268702	2·89752E-46
20a	Concentration S	0·749946397	0·695644069	0·794560256	7·31466E-41
20b	Concentration I	0·741353997	0·68518572	0·787500888	4·09493E-39
21a	Memory S	0·827040309	0·789479907	0·85789929	7·72402E-62
21b	Memory I	0·819651955	0·780487079	0·851829145	2·69496E-59
22a	General pain F	0·797467866	0·753485431	0·833603078	1·88406E-52
22b	General pain S	0·81677249	0·776982301	0·849463426	2·41719E-58
22c	General pain I	0·828310049	0·791025387	0·858942486	2·72895E-62
23a	Headache F	0·861720824	0·831691738	0·886392205	4·66562E-76
23b	Headache S	0·879408079	0·853220006	0·90092375	3·22953E-85
23c	Headache I	0·873943079	0·846568211	0·896433799	3·24312E-82
24a	Insomnia S	0·876898113	0·850164969	0·898861605	8·09963E-84
24b	Insomnia I	0·860458793	0·830155641	0·885355342	1·84148E-75
25a	Fatigue S	0·828106575	0·790777727	0·858775315	3·22632E-62
25b	Fatigue I	0·83591531	0·800282227	0·865190838	4·28709E-65

NO.	PRO-CTCAE Item	ICC	95%CI_Lower	95%CI_Upper	P
26a	Anxious F	0·806631101	0·76463858	0·841131435	3·81032E-55
26b	Anxious S	0·842289115	0·808040186	0·870427446	1·3972E-67
26c	Anxious I	0·820481397	0·781496644	0·8525106	1·41982E-59
27a	Discouraged F	0·757790531	0·705191657	0·801004862	1·53584E-42
27b	Discouraged S	0·780226687	0·732500111	0·819438022	7·98472E-48
27c	Discouraged I	0·791364492	0·746056632	0·828588652	9·45613E-51
28a	Urinary incontinence F	0·895551055	0·872868635	0·914186541	3·61294E-95
28b	Urinary incontinence I	0·880470574	0·854513235	0·901796677	8·05072E-86
29a	Decreased libido S	0·868215394	0·839596687	0·891728032	3·0673E-79
30a	Decreased sweating P	0·549946744	0·452211943	0·630243978	1·81933E-15
31a	Hot flashes F	0·772212306	0·722745305	0·812853545	7·55033E-46
31b	Hot flashes S	0·80633942	0·764283556	0·840891795	4·67161E-55

Abbreviations: F, Frequency; I, Interference; S, Severity; P, Presence/Absence; A, Amount.

Table S8. Convergent Validity Analysis Results.

NO.	PRO-CTCAE Item	QLQ-C30 Domain	r	95%CI	P
1a	Difficulty swallowing S	Physical functioning	0·317	0·22, 0·407	9·50715E-10
2a	Taste changes S	Physical functioning	0·319	0·222, 0·409	7·43003E-10
3a	Decreased appetite S	Appetite loss	0·826	0·79, 0·856	4·26636E-90
3b	Decreased appetite I	Appetite loss	0·743	0·693, 0·787	8·48774E-64
4a	Nausea F	Nausea and vomiting	0·736	0·685, 0·781	5·03757E-62
4b	Nausea S	Nausea and vomiting	0·747	0·698, 0·79	7·8284E-65
5a	Vomiting F	Nausea and vomiting	0·749	0·7, 0·791	2·75619E-65
5b	Vomiting S	Nausea and vomiting	0·651	0·587, 0·707	2·75133E-44
6a	Constipation S	Constipation	0·793	0·751, 0·828	3·94742E-78
7a	Shortness of breath S	Dyspnoea	0·49	0·407, 0·565	6·71443E-23
7b	Shortness of breath I	Dyspnoea	0·499	0·417, 0·573	7·87307E-24
8a	Heart palpitations F	Physical functioning	0·285	0·186, 0·377	4·68384E-08
8b	Heart palpitations S	Physical functioning	0·306	0·209, 0·398	3·56599E-09
9a	Rash P	Physical functioning	0·084	-0·02, 0·186	0·112968031
10a	Hair loss A	Physical functioning	0·276	0·177, 0·369	1·20902E-07
11a	Itching S	Physical functioning	0·195	0·093, 0·293	0·000213101
12a	Hives P	Physical functioning	0·04	-0·064, 0·144	0·446544147
13a	Bed/pressure sores P	Physical functioning	0·041	-0·063, 0·144	0·441088342
14a	Radiation skin reaction S	Physical functioning	0·133	0·03, 0·234	0·011763372
15a	Numbness & tingling S	Physical functioning	0·526	0·447, 0·598	9·21296E-27
15b	Numbness & tingling I	Physical functioning	0·57	0·495, 0·636	4·69039E-32
16a	Dizziness S	Physical functioning	0·329	0·233, 0·419	1·96538E-10
16b	Dizziness I	Physical functioning	0·38	0·288, 0·466	1·09605E-13
17a	Blurred vision S	Physical functioning	0·266	0·166, 0·36	3·69659E-07
17b	Blurred vision I	Physical functioning	0·275	0·176, 0·369	1·29452E-07
18a	Flashing lights P	Physical functioning	0·035	-0·069, 0·139	0·505262972
19a	Ringing in ears S	Physical functioning	0·175	0·072, 0·274	0·000933176
20a	Concentration S	Cognitive functioning	0·644	0·578, 0·701	4·86252E-43
20b	Concentration I	Cognitive functioning	0·623	0·555, 0·683	1·02191E-39
21a	Memory S	Cognitive functioning	0·782	0·738, 0·819	9·9591E-75
21b	Memory I	Cognitive functioning	0·294	0·196, 0·386	1·50921E-08
22a	General pain F	Pain	0·716	0·661, 0·763	3·16009E-57
22b	General pain S	Pain	0·734	0·682, 0·779	1·62495E-61
22c	General pain I	Pain	0·747	0·697, 0·79	9·94807E-65
23a	Headache F	Pain	0·568	0·493, 0·635	7·90422E-32
23b	Headache S	Pain	0·63	0·563, 0·689	8·74551E-41
23c	Headache I	Pain	0·174	0·071, 0·273	0·001012094
24a	Insomnia S	Insomnia	0·715	0·66, 0·762	5·99741E-57
24b	Insomnia I	Insomnia	0·429	0·341, 0·511	2·11054E-17
25a	Fatigue S	Fatigue	0·785	0·741, 0·822	1·70234E-75
25b	Fatigue I	Fatigue	0·527	0·448, 0·598	7·29109E-27

NO.	PRO-CTCAE Item	QLQ-C30 Domain	r	95%CI	P
26a	Anxious F	Emotional functioning	0·744	0·694, 0·787	4·81382E-64
26b	Anxious S	Emotional functioning	0·747	0·698, 0·79	7·53528E-65
26c	Anxious I	Emotional functioning	0·709	0·653, 0·757	1·13276E-55
27a	Discouraged F	Emotional functioning	0·658	0·595, 0·713	1·55951E-45
27b	Discouraged S	Emotional functioning	0·647	0·582, 0·704	1·23646E-43
27c	Discouraged I	Emotional functioning	0·435	0·347, 0·516	6·83221E-18
28a	Urinary incontinence F	Physical functioning	0·43	0·341, 0·511	1·8639E-17
28b	Urinary incontinence I	Physical functioning	0·295	0·197, 0·387	1·35996E-08
29a	Decreased libido S	Emotional functioning	0·044	-0·06, 0·147	0·410228343
30a	Decreased sweating P	Physical functioning	0·179	0·076, 0·278	0·000692359
31a	Hot flashes F	Physical functioning	0·25	0·15, 0·345	1·80994E-06
31b	Hot flashes S	Physical functioning	0·305	0·208, 0·397	4·08083E-09
1a	Difficulty swallowing S	Global health status/QOL	0·373	0·28, 0·459	3·53061E-13
2a	Taste changes S	Global health status/QOL	0·343	0·248, 0·432	2·71145E-11
3a	Decreased appetite S	Global health status/QOL	0·353	0·259, 0·441	6·93664E-12
3b	Decreased appetite I	Global health status/QOL	0·377	0·284, 0·463	1·78372E-13
4a	Nausea F	Global health status/QOL	0·207	0·106, 0·305	8·10358E-05
4b	Nausea S	Global health status/QOL	0·232	0·132, 0·328	9·515E-06
5a	Vomiting F	Global health status/QOL	0·2	0·098, 0·298	0·00014493
5b	Vomiting S	Global health status/QOL	0·158	0·055, 0·257	0·002863162
6a	Constipation S	Global health status/QOL	0·271	0·172, 0·365	2·0663E-07
7a	Shortness of breath S	Global health status/QOL	0·28	0·181, 0·373	7·93646E-08
7b	Shortness of breath I	Global health status/QOL	0·284	0·185, 0·377	5·03662E-08
8a	Heart palpitations F	Global health status/QOL	0·307	0·21, 0·398	3·27227E-09
8b	Heart palpitations S	Global health status/QOL	0·32	0·223, 0·41	6·54365E-10
9a	Rash P	Global health status/QOL	0·059	-0·045, 0·162	0·26910018
10a	Hair loss A	Global health status/QOL	0·147	0·044, 0·248	0·005309445
11a	Itching S	Global health status/QOL	0·16	0·057, 0·26	0·002432999
12a	Hives P	Global health status/QOL	0·015	-0·089, 0·119	0·774124041
13a	Bed/pressure sores P	Global health status/QOL	0·056	-0·048, 0·159	0·29257739
14a	Radiation skin reaction S	Global health status/QOL	0·17	0·067, 0·269	0·001316004
15a	Numbness & tingling S	Global health status/QOL	0·377	0·284, 0·463	1·90725E-13
15b	Numbness & tingling I	Global health status/QOL	0·41	0·32, 0·493	6·98069E-16
16a	Dizziness S	Global health status/QOL	0·347	0·252, 0·435	1·73248E-11
16b	Dizziness I	Global health status/QOL	0·41	0·32, 0·493	7·28391E-16
17a	Blurred vision S	Global health status/QOL	0·318	0·221, 0·408	8·41556E-10
17b	Blurred vision I	Global health status/QOL	0·356	0·262, 0·444	4·31637E-12
18a	Flashing lights P	Global health status/QOL	0·09	-0·014, 0·192	0·088934659
19a	Ringing in ears S	Global health status/QOL	0·095	-0·009, 0·197	0·071962066
20a	Concentration S	Global health status/QOL	0·404	0·313, 0·488	2·00526E-15
20b	Concentration I	Global health status/QOL	0·43	0·342, 0·511	1·79092E-17
21a	Memory S	Global health status/QOL	0·413	0·323, 0·496	4·09737E-16

NO.	PRO-CTCAE Item	QLQ-C30 Domain	r	95%CI	P
21b	Memory I	Global health status/QOL	0·449	0·362, 0·528	4·59807E-19
22a	General pain F	Global health status/QOL	0·341	0·245, 0·429	4·08747E-11
22b	General pain S	Global health status/QOL	0·395	0·303, 0·479	1·03444E-14
22c	General pain I	Global health status/QOL	0·401	0·31, 0·485	3·42903E-15
23a	Headache F	Global health status/QOL	0·272	0·173, 0·366	1·83356E-07
23b	Headache S	Global health status/QOL	0·329	0·233, 0·418	2·0169E-10
23c	Headache I	Global health status/QOL	0·377	0·284, 0·462	1·91918E-13
24a	Insomnia S	Global health status/QOL	0·26	0·16, 0·354	6·75054E-07
24b	Insomnia I	Global health status/QOL	0·285	0·186, 0·378	4·5E-08
25a	Fatigue S	Global health status/QOL	0·474	0·389, 0·55	2·65096E-21
25b	Fatigue I	Global health status/QOL	0·48	0·396, 0·557	5·82412E-22
26a	Anxious F	Global health status/QOL	0·32	0·223, 0·41	6·48076E-10
26b	Anxious S	Global health status/QOL	0·387	0·294, 0·472	3·91755E-14
26c	Anxious I	Global health status/QOL	0·449	0·362, 0·529	4·18186E-19
27a	Discouraged F	Global health status/QOL	0·376	0·284, 0·462	1·97121E-13
27b	Discouraged S	Global health status/QOL	0·423	0·334, 0·505	6·6061E-17
27c	Discouraged I	Global health status/QOL	0·421	0·332, 0·503	9·69335E-17
28a	Urinary incontinence F	Global health status/QOL	0·297	0·2, 0·389	1·04799E-08
28b	Urinary incontinence I	Global health status/QOL	0·318	0·221, 0·408	8·54897E-10
29a	Decreased libido S	Global health status/QOL	0·087	-0·017, 0·19	0·099860076
30a	Decreased sweating P	Global health status/QOL	0·158	0·055, 0·258	0·002799193
31a	Hot flashes F	Global health status/QOL	0·209	0·108, 0·306	6·95742E-05
31b	Hot flashes S	Global health status/QOL	0·274	0·175, 0·367	1·53143E-07

Abbreviations: F, Frequency; I, Interference; S, Severity; P, Presence/Absence; A, Amount.

Table S9. Known-Groups Validity Analysis Results.

NO.	PRO-CTCAE Item	Cohens_d	P
1a	Difficulty swallowing S	0·388036769	0·000880676
2a	Taste changes S	0·589889568	5·52346E-07
3a	Decreased appetite S	0·537691186	4·7111E-06
3b	Decreased appetite I	0·505028527	1·6591E-05
4a	Nausea F	0·431413545	0·000223081
4b	Nausea S	0·482357388	3·82718E-05
5a	Vomiting F	0·438440921	0·000176611
5b	Vomiting S	0·410186377	0·000443333
6a	Constipation S	0·639081475	6·34249E-08
7a	Shortness of breath S	0·447355469	0·000130739
7b	Shortness of breath I	0·448179724	0·000127122
8a	Heart palpitations F	0·395848726	0·000693779
8b	Heart palpitations S	0·459987494	8·46546E-05
9a	Rash P	0·289452573	0·013021676
10a	Hair loss A	0·418345115	0·00034162
11a	Itching S	0·233264125	0·044513293
12a	Hives P	0·192908429	0·096416759
13a	Bed/pressure sores P	0·034717771	0·765383529
14a	Radiation skin reaction S	0·402106229	0·000571519
15a	Numbness & tingling S	0·760606952	1·70825E-10
15b	Numbness & tingling I	0·818390597	7·8391E-12
16a	Dizziness S	0·503446299	1·76055E-05
16b	Dizziness I	0·626266751	1·12945E-07
17a	Blurred vision S	0·384523294	0·000979177
17b	Blurred vision I	0·340146937	0·003491936
18a	Flashing lights P	0·239573295	0·039363594
19a	Ringing in ears S	0·106524254	0·357793468
20a	Concentration S	0·702684075	3·16294E-09
20b	Concentration I	0·76548603	1·3254E-10
21a	Memory S	0·693031049	5·05675E-09
21b	Memory I	0·715883678	1·65177E-09
22a	General pain F	0·390629646	0·000813988
22b	General pain S	0·479329836	4·26897E-05
22c	General pain I	0·510168063	1·36676E-05
23a	Headache F	0·350705317	0·002609911
23b	Headache S	0·395050321	0·000711027
23c	Headache I	0·476809998	4·67331E-05
24a	Insomnia S	0·442003699	0·000156702
24b	Insomnia I	0·571867537	1·17886E-06
25a	Fatigue S	0·736952962	5·74559E-10
25b	Fatigue I	0·862971995	6·51284E-13

NO.	PRO-CTCAE Item	Cohens_d	P
26a	Anxious F	0·586984172	6·2495E-07
26b	Anxious S	0·58611026	6·48539E-07
26c	Anxious I	0·676139837	1·13556E-08
27a	Discouraged F	0·586294692	6·4349E-07
27b	Discouraged S	0·602039209	3·2782E-07
27c	Discouraged I	0·704804784	2·85123E-09
28a	Urinary incontinence F	0·698015592	3·97114E-09
28b	Urinary incontinence I	0·704393999	2·90916E-09
29a	Decreased libido S	0·051559326	0·656111184
30a	Decreased sweating P	0·204960248	0·077496218
31a	Hot flashes F	0·287126434	0·013525022
31b	Hot flashes S	0·261225782	0·024545245

Abbreviations: F, Frequency; I, Interference; S, Severity; P, Presence/Absence; A, Amount.

Table S10. Responsiveness Analysis Results.

NO.	PRO-CTCAE Item	GIC Domain	Improved	Improved_P	No_Change	No_Change_P	Worse	Worse_P
1a	Difficulty swallowing S	Physical condition	-0·104174057	0·679662037	0·100636757	0·467507528	0·181766426	0·421811515
2a	Taste changes S	Physical condition	0·007661436	0·535698158	0·221855758	0·118972675	0·134546119	0·517269311
3a	Decreased appetite S	Emotional state	0·03112728	0·266575783	0·226624349	0·058506644	0·280988643	0·137331611
3b	Decreased appetite I	Emotional state	-0·037217979	0·584653753	0·237364732	0·075053157	0·353213736	0·084675403
4a	Nausea F	Physical condition	-0·033059552	0·405479916	-0·039910913	0·518255227	0·048546904	0·484648495
4b	Nausea S	Physical condition	-0·053441744	0·633367888	0·048953162	0·359244665	0·25950668	0·227911008
5a	Vomiting F	Physical condition	-0·058924138	0·711400384	-0·016061724	0·664526865	0·122377708	0·384575202
5b	Vomiting S	Physical condition	-0·056198146	0·729678627	0·019245905	0·740118771	0·065560279	0·46167092
6a	Constipation S	Physical condition	-0·187523817	0·959750384	-0·029613091	0·625134797	0·172406729	0·253360257
7a	Shortness of breath S	Physical condition	-0·08741716	0·743389472	-0·147031825	0·774961989	0·493265523	0·058385949
7b	Shortness of breath I	Physical condition	0·068387474	0·393174479	-0·077019887	0·600493126	0·430676303	0·126088417
8a	Heart palpitations F	Physical condition	-0·075087625	0·732523855	0·136416592	0·275896461	0·312474718	0·135279367
8b	Heart palpitations S	Physical condition	-0·038192481	0·672520629	0·069316118	0·308303599	0·294900944	0·136328234
9a	Rash P	Skin condition	0	1	-0·252910892	1	0·723934913	0·307597989
10a	Hair loss A	Skin condition	-0·078001291	0·65370134	0·123657882	0·186350651	0·343441198	0·082304531
11a	Itching S	Skin condition	0·017718848	0·585386301	-0·018245367	0·513125591	0·521750832	0·064835112
12a	Hives P	Skin condition	-0·108284877	1	-0·510921554	1	0·358785831	0·930355317
13a	Bed/pressure sores P	Skin condition	-1·475346254	1	-1·761473507	1	-0·277443895	0·999989336
14a	Radiation skin reaction S	Skin condition	-0·029745802	0·572014388	-0·096233407	0·747598813	0·493355186	0·056832374
15a	Numbness & tingling S	Physical condition	-0·028492462	0·532866936	-0·052945501	0·519965286	0·459474754	0·046563644
15b	Numbness & tingling I	Physical condition	0	0·448543735	0·110461633	0·265009687	0·335213877	0·048469094
16a	Dizziness S	Physical condition	-0·091939538	0·832539681	0·057417806	0·303787231	0·363027125	0·075821357
16b	Dizziness I	Physical condition	-0·007107635	0·566396269	0·039273046	0·324594354	0·549976716	0·007456321

NO.	PRO-CTCAE Item	GIC Domain	Improved	Improved_P	No_Change	No_Change_P	Worse	Worse_P
17a	Blurred vision S	Physical condition	0·060167079	0·438032451	0·093398056	0·329239064	0·28034351	0·141655918
17b	Blurred vision I	Physical condition	-0·013353831	0·755317991	0·04625733	0·362458249	0·277323096	0·133198264
18a	Flashing lights P	Physical condition	-0·531705182	1	-0·860293793	1	-0·517770322	0·99999994
19a	Ringing in ears S	Physical condition	0·151191103	0·094606721	0·086206879	0·309311222	0·254006016	0·244211158
20a	Concentration S	Emotional state	0·095696517	0·216107874	0·04152334	0·320361817	0·358724584	0·094160493
20b	Concentration I	Emotional state	0·019729576	0·479574267	0·027708246	0·312102518	0·458257569	0·040553859
21a	Memory S	Emotional state	0·085098929	0·169597255	0·06495063	0·320361817	0·280927534	0·113168709
21b	Memory I	Emotional state	0·056745044	0·16225076	0·010731513	0·532132625	0·289282849	0·104407263
22a	General pain F	Emotional state	0·010405959	0·550947385	0·091687516	0·235140655	0·026052826	0·504685905
22b	General pain S	Emotional state	0	0·545415035	0·299562542	0·035772758	0·125209282	0·453247405
22c	General pain I	Emotional state	0·006362371	0·652300457	0·273619052	0·02618676	-0·05288055	0·635520159
23a	Headache F	Emotional state	-0·120971009	0·85359873	-0·034554881	0·487817255	-0·088399748	0·586013775
23b	Headache S	Emotional state	-0·064741178	0·613299971	-0·028870536	0·509259494	-0·035052634	0·532765167
23c	Headache I	Emotional state	-0·020874505	0·527842811	0·059479221	0·299677386	0·110069524	0·298550449
24a	Insomnia S	Emotional state	-0·085663174	0·554016708	-0·023669553	0·530675002	0·20255325	0·202148228
24b	Insomnia I	Emotional state	-0·070167018	0·570378215	0	0·345665039	0·241389363	0·169206285
25a	Fatigue S	Emotional state	0·058160426	0·305879381	0·134511495	0·188871265	0·561102067	0·013612891
25b	Fatigue I	Emotional state	0·060011361	0·323235325	0·136027497	0·155581507	0·38550666	0·077618101
26a	Anxious F	Emotional state	0·010289086	0·459510051	0·056292901	0·262622545	0·435745607	0·034330473
26b	Anxious S	Emotional state	-0·025881676	0·446750327	0·053490662	0·228434633	0·333053103	0·084675403
26c	Anxious I	Emotional state	-0·029157798	0·542799403	0·077924423	0·246169848	0·351637174	0·065601764
27a	Discouraged F	Emotional state	-0·080288193	0·756712618	0·100700575	0·402538363	0·462351991	0·035692133
27b	Discouraged S	Emotional state	-0·041971937	0·537408563	0·136309178	0·295867371	0·370700167	0·089324316
27c	Discouraged I	Emotional state	0·006154149	0·402082068	0·144078636	0·332696353	0·34187973	0·091236131

NO.	PRO-CTCAE Item	GIC Domain	Improved	Improved_P	No_Change	No_Change_P	Worse	Worse_P
28a	Urinary incontinence F	Physical condition	0·035201141	0·376696524	0·079318746	0·354975364	0·207387516	0·208079896
28b	Urinary incontinence I	Physical condition	0·084122422	0·195922032	0·14578403	0·232398648	0·2498726	0·160813992
29a	Decreased libido S	Emotional state	0·0235766	0·3019027	0·2754351	0·0117215	0·0835984	0·3591643
30a	Decreased sweating P	Physical condition	-0·517857506	1	-0·619932499	1	0·195830321	0·991927611
31a	Hot flashes F	Physical condition	-0·037116305	0·669944874	-0·084312602	0·522244788	0·09770787	0·351939702
31b	Hot flashes S	Physical condition	-0·038622284	0·606104488	-0·04324958	0·481174715	0·118826553	0·265833681
1a	Difficulty swallowing S	Global quality of life	-0·029640506	0·561597934	-0·102776847	0·673696114	0·281338348	0·344880479
2a	Taste changes S	Global quality of life	0·02591878	0·544765773	0·190915252	0·188257738	0·218129881	0·342161293
3a	Decreased appetite S	Global quality of life	0·047486318	0·222803495	0·144568595	0·138658268	0·524804156	0·039259326
3b	Decreased appetite I	Global quality of life	0·005454783	0·429706539	0·10426131	0·283516984	0·571004982	0·022972544
4a	Nausea F	Global quality of life	-0·044350674	0·429578907	-0·073255745	0·632504112	0·23363051	0·264891245
4b	Nausea S	Global quality of life	-0·040282213	0·586945831	0·069344515	0·443628024	0·29471332	0·197633506
5a	Vomiting F	Global quality of life	-0·072114758	0·756726284	0·055408092	0·546556555	0·117341218	0·403637475
5b	Vomiting S	Global quality of life	-0·057066328	0·803470322	0·020954884	0·662762662	0·138894036	0·336747526
6a	Constipation S	Global quality of life	-0·2005602	0·969818733	-0·017328923	0·478093675	0·389880512	0·148635845
7a	Shortness of breath S	Global quality of life	-0·098526789	0·782299822	-0·112459436	0·704971386	0·597976385	0·037413504
7b	Shortness of breath I	Global quality of life	0·032047816	0·461810711	-0·056162738	0·554971745	0·586706091	0·062985097
8a	Heart palpitations F	Global quality of life	-0·082840706	0·81409325	0·169093201	0·192591821	0·41580497	0·061176463
8b	Heart palpitations S	Global quality of life	-0·089382455	0·76289169	0·176167066	0·195025781	0·431596197	0·065775806
9a	Rash P	Global quality of life	-0·104311548	1	0·052492434	0·999998225	0·341875586	0·922094919
10a	Hair loss A	Global quality of life	0·060107526	0·211534431	-0·011413893	0·617722312	0·146208111	0·216644898
11a	Itching S	Global quality of life	0·051591712	0·44186321	0·057777496	0·478799654	0·109451795	0·386559971
12a	Hives P	Global quality of life	-0·192010956	1	-0·351411313	1	-0·090408141	0·999160122
13a	Bed/pressure sores P	Global quality of life	-1·381449	1	-1·609636666	1	-0·82635971	0·999999956

NO.	PRO-CTCAE Item	GIC Domain	Improved	Improved_P	No_Change	No_Change_P	Worse	Worse_P
14a	Radiation skin reaction S	Global quality of life	-0·026755296	0·460133288	-0·104589694	0·789470464	0·235002572	0·260072306
15a	Numbness & tingling S	Global quality of life	-0·013452644	0·449570171	-0·036385488	0·540231202	0·490176881	0·062985097
15b	Numbness & tingling I	Global quality of life	0·013040292	0·366093445	0·017392115	0·376208441	0·485168003	0·02638736
16a	Dizziness S	Global quality of life	-0·089301079	0·786106318	0·062343343	0·393819965	0·492637778	0·051163134
16b	Dizziness I	Global quality of life	0	0·48600029	0·039370827	0·349621446	0·692163666	0·007355222
17a	Blurred vision S	Global quality of life	0·012624932	0·63561713	0·173138693	0·17107981	0·502849026	0·035639183
17b	Blurred vision I	Global quality of life	-0·005735057	0·778937307	-0·036205033	0·488689337	0·536741725	0·022972544
18a	Flashing lights P	Global quality of life	-0·553387357	1	-0·805856725	1	-0·583249672	0·999999574
19a	Ringing in ears S	Global quality of life	0·113346488	0·170819864	0·166675068	0·204444085	0·430511689	0·127476589
20a	Concentration S	Global quality of life	0·072348834	0·262914179	0·098296169	0·229949461	0·369069176	0·105780618
20b	Concentration I	Global quality of life	0·017917979	0·480430167	0·076824013	0·22941175	0·429583301	0·069644683
21a	Memory S	Global quality of life	0·115572969	0·093126089	-0·057857872	0·553570828	0·356014831	0·115548373
21b	Memory I	Global quality of life	0·071628643	0·138423952	-0·101317969	0·686369877	0·413906413	0·065775806
22a	General pain F	Global quality of life	-0·027690077	0·628267495	0·156690357	0·216720006	0·179392916	0·248225774
22b	General pain S	Global quality of life	0·022753418	0·433922439	0·240680842	0·126871322	0·280300653	0·225422901
22c	General pain I	Global quality of life	0·017076765	0·529921864	0·1968443	0·122861568	0·11831157	0·323362289
23a	Headache F	Global quality of life	-0·130922078	0·887659533	0·017179009	0·424106452	-0·033338042	0·461721066
23b	Headache S	Global quality of life	-0·061649326	0·641339873	-0·019575967	0·426190038	-0·039994577	0·558767158
23c	Headache I	Global quality of life	0	0·419647316	0·039879565	0·302408285	0·08242161	0·447073032
24a	Insomnia S	Global quality of life	-0·045187516	0·496758697	-0·123522229	0·640486493	0·316311546	0·150355524
24b	Insomnia I	Global quality of life	-0·05166914	0·524359713	-0·100142437	0·569636658	0·508287	0·039259326
25a	Fatigue S	Global quality of life	0·086925228	0·183739297	0·077586914	0·360839764	0·660884388	0·007979292
25b	Fatigue I	Global quality of life	0·090496894	0·19250369	0·039412694	0·414411981	0·513146554	0·033381307
26a	Anxious F	Global quality of life	0·022555894	0·432644077	0·202111956	0·037886359	0·095285104	0·35034283

NO.	PRO-CTCAE Item	GIC Domain	Improved	Improved_P	No_Change	No_Change_P	Worse	Worse_P
26b	Anxious S	Global quality of life	-0·022870139	0·486648177	0·219482916	0·026479331	0	0·485258026
26c	Anxious I	Global quality of life	-0·015517616	0·550429829	0·190836356	0·059224056	0·131561383	0·328690214
27a	Discouraged F	Global quality of life	-0·047986315	0·737615656	0·065859654	0·329504673	0·531826893	0·039259326
27b	Discouraged S	Global quality of life	0·005208269	0·452143539	0·05944366	0·272813786	0·401362866	0·135352669
27c	Discouraged I	Global quality of life	0·021208151	0·412927612	0·061729306	0·275174858	0·545575009	0·03449507
28a	Urinary incontinence F	Global quality of life	0·040045069	0·404469504	0·077570597	0·257144206	0·242115789	0·212331102
28b	Urinary incontinence I	Global quality of life	0·094575414	0·202525926	0·122183171	0·215681011	0·298176091	0·127476589
29a	Decreased libido S	Global quality of life	0·1088327	0·1249833	0·1759811	0·1030428	0·0835984	0·3591643
30a	Decreased sweating P	Global quality of life	-0·557289259	1	-0·401313413	1	0·237443471	0·953329602
31a	Hot flashes F	Global quality of life	-0·062074348	0·73655544	0·041213161	0·246404837	0	0·535342438
31b	Hot flashes S	Global quality of life	-0·063639679	0·610565874	0·082896162	0·330146342	0·04172749	0·447073032

Abbreviations: F, Frequency; I, Interference; S, Severity; P, Presence/Absence; A, Amount.

Table S11a. Subgroup Analysis Data (Test-Retest Reliability).

NO.	PRO-CTCAE Item	Astrocytoma	Glioblastoma	Oligodendroglioma	IDHmut	Stupp	nonStupp	StandardStupp	StuppTTfields
1a	Difficulty swallowing S	0·642072214	0·793034213	0·658268734	0·647569444	0·877956181	0·345273169	0·870578558	0·789808917
2a	Taste changes S	0·552442997	0·63397754	0·729090909	0·645924765	0·628513376	0·600052729	0·612980769	0·426229508
3a	Decreased appetite S	0·788969521	0·74777967	0·687396989	0·747533271	0·674584747	0·601375696	0·568835098	0·815508021
3b	Decreased appetite I	0·613287905	0·74250076	0·657981999	0·636181109	0·65244563	0·541516245	0·546869211	0·82259887
4a	Nausea F	0·856404365	0·794816347	0·811669129	0·836223507	0·778188138	0·787804476	0·748274764	0·83499006
4b	Nausea S	0·741509249	0·782507725	0·793821839	0·766739931	0·527816831	0·770024437	0·441660141	0·75
5a	Vomiting F	0·708850621	0·841946278	0·841415465	0·765192027	0·847435263	0·795692492	0·837484171	0·795180723
5b	Vomiting S	0·708960843	0·850284532	0·843589744	0·752635278	0·822310757	0·776450274	0·800181036	1
6a	Constipation S	0·853027523	0·901762663	0·821331354	0·840226748	0·856157731	0·890918753	0·825267554	0·922252011
7a	Shortness of breath S	0·675656494	0·505402917	0·84079602	0·752976791	0·516944285	0·605440595	0·438129496	1
7b	Shortness of breath I	0·700994318	0·505564388	0·649499285	0·690226265	0·702904343	0·48052618	0·679020819	1
8a	Heart palpitations F	0·76583277	0·730163001	0·648113791	0·735574833	0·719148936	0·77860639	0·767765568	0·456296296
8b	Heart palpitations S	0·764519953	0·643379442	0·765730881	0·769672916	0·63994503	0·689010255	0·635312259	0·563968668
9a	Rash P	0·795837463	0·636792453	0·855630074	0·821239962	0·696225285	0·624828218	0·678481013	0·789808917
10a	Hair loss A	0·801356141	0·789985935	0·886678066	0·844678218	0·864354697	0·884418564	0·86908078	0·847953216
11a	Itching S	0·733798604	0·855945654	0·827440633	0·776127049	0·87130696	0·773757066	0·860634425	0·898809524
12a	Hives P	0·724087591	0·65176591	0·748280605	0·733238737	0·830809743	0·586327046	0·770169492	1
13a	Bed/pressure sores P	0·781725888	0·834922964	-0·070175439	0·585558853	0·781276596	0·693533271	0·776315789	0·795180723
14a	Radiation skin reaction S	0·956012064	0·614275864	0·711610487	0·814359001	0·585674157	0·837355449	0·482487357	0·635620915
15a	Numbness & tingling S	0·759417378	0·795953615	0·574170666	0·727346525	0·723955018	0·767415184	0·714888233	0·707165109
15b	Numbness & tingling I	0·763320123	0·752955236	0·342169748	0·700574748	0·672740936	0·791422723	0·668067227	0·609022556
16a	Dizziness S	0·852619844	0·824797699	0·782171799	0·822738121	0·845182811	0·775190201	0·765610292	0·977520873
16b	Dizziness I	0·760018958	0·750183707	0·697144505	0·731968677	0·756368657	0·665498793	0·69599695	0·869236584
17a	Blurred vision S	0·834965035	0·848807237	0·872716515	0·856135599	0·924139781	0·773921995	0·904015605	0·983574879
17b	Blurred vision I	0·805447756	0·817305714	0·834912346	0·820109338	0·819839533	0·803755493	0·767761194	0·968955786
18a	Flashing lights P	0·622329706	0·495000877	0·669371197	0·641581489	0·504025765	0·450845335	0·61627907	1
19a	Ringing in ears S	0·805665722	0·691996025	0·887706044	0·858276044	0·792897767	0·753083923	0·793885721	0·617977528
20a	Concentration S	0·663719649	0·796379263	0·660130719	0·665306978	0·79656739	0·650753705	0·751345809	0·88127295
20b	Concentration I	0·668916385	0·748493516	0·564866267	0·625652174	0·682277545	0·64706669	0·643209089	0·577889447

NO.	PRO-CTCAE Item	Astrocytoma	Glioblastoma	Oligodendroglioma	IDHmut	Stupp	nonStupp	StandardStupp	StuppTTfields
21a	Memory S	0·869158879	0·835786382	0·741101092	0·814138205	0·792347314	0·829173212	0·760044643	0·811428571
21b	Memory I	0·87915899	0·778432923	0·750261362	0·829813438	0·79622661	0·750319635	0·785201488	0·691796009
22a	General pain F	0·832240039	0·801198838	0·734476124	0·791259344	0·815626833	0·783369248	0·744920196	0·899793814
22b	General pain S	0·840587175	0·806227975	0·756514343	0·813363251	0·777145624	0·775016164	0·6761868	0·892742453
22c	General pain I	0·85545224	0·798217294	0·784568905	0·828637778	0·801282714	0·787869296	0·758691985	0·860195904
23a	Headache F	0·85119303	0·869585941	0·844928982	0·847975246	0·90306603	0·794380864	0·869713721	0·93557231
23b	Headache S	0·854610337	0·852779096	0·832608696	0·844253718	0·87112578	0·798275201	0·832610157	0·908015769
23c	Headache I	0·86795995	0·845194656	0·790408958	0·836473787	0·827551124	0·804698321	0·787059606	0·882220555
24a	Insomnia S	0·856603354	0·898539897	0·82151218	0·843701799	0·859557048	0·886777676	0·841091157	0·877338877
24b	Insomnia I	0·809352901	0·859678377	0·721825963	0·780978704	0·881807419	0·776568657	0·867142306	0·911983033
25a	Fatigue S	0·797804836	0·848609109	0·767636555	0·784831161	0·889692913	0·784830372	0·827952642	0·962148962
25b	Fatigue I	0·84529703	0·811652866	0·813903889	0·831578844	0·87012987	0·777749351	0·835499515	0·896670494
26a	Anxious F	0·907360448	0·788194007	0·734553776	0·831745493	0·832039028	0·829915561	0·788728632	0·852444444
26b	Anxious S	0·873261206	0·809435583	0·79772224	0·841196234	0·866655087	0·798882038	0·846717405	0·870655271
26c	Anxious I	0·827928893	0·753906042	0·804867449	0·816524442	0·776574689	0·741175041	0·738406123	0·816326531
27a	Discouraged F	0·701745878	0·777858311	0·759532595	0·728455285	0·746699849	0·766185201	0·705251455	0·758782201
27b	Discouraged S	0·741198271	0·78847732	0·641337386	0·701277283	0·74820588	0·735955745	0·718691338	0·733153639
27c	Discouraged I	0·812798472	0·767383405	0·648207703	0·748945741	0·756058607	0·75493452	0·72352534	0·753295669
28a	Urinary incontinence F	0·90835047	0·895028315	0·856958168	0·895769567	0·926302806	0·870932591	0·916728222	0·949416342
28b	Urinary incontinence I	0·882918433	0·873892174	0·829801966	0·873678248	0·897090751	0·838004342	0·88287976	0·952251023
29a	Decreased libido S	0·947607095	0·845298314	0·838182898	0·895346666	0·876732436	0·888802416	0·859808996	0·855497275
30a	Decreased sweating P	0·586248492	0·474673988	0·647594278	0·608450704	0·563921172	0·519160182	0·574506284	1
31a	Hot flashes F	0·772367515	0·78527236	0·710892711	0·743350074	0·814954354	0·779033133	0·744886191	0·876478318
31b	Hot flashes S	0·79584604	0·853316245	0·764265669	0·779392074	0·865338536	0·802789159	0·84387452	0·779443255

Abbreviations: F, Frequency; I, Interference; S, Severity; P, Presence/Absence; A, Amount.

Table S11b. Subgroup Analysis Data (Convergent Validity-QoL).

NO.	PRO-CTCAE Item	Astrocytoma	Glioblastoma	Oligodendroglioma	IDHmut	Stupp	nonStupp	StandardStupp	StuppTTfields
1a	Difficulty swallowing S	0·152	0·449	0·395	0·283	0·255	0·384	0·253	0·13
2a	Taste changes S	0·138	0·451	0·268	0·204	0·298	0·399	0·271	0·221
3a	Decreased appetite S	0·192	0·439	0·288	0·242	0·371	0·357	0·337	0·248
3b	Decreased appetite I	0·229	0·445	0·302	0·266	0·388	0·351	0·397	0·212
4a	Nausea F	0·152	0·303	0·081	0·12	0·19	0·251	0·07	0·33
4b	Nausea S	0·243	0·303	0·113	0·188	0·337	0·206	0·25	0·513
5a	Vomiting F	0·156	0·263	0·12	0·141	0·227	0·241	0·167	0·328
5b	Vomiting S	0·135	0·213	0·085	0·113	0·205	0·176	0·154	0·276
6a	Constipation S	0·136	0·403	0·108	0·124	0·252	0·217	0·145	0·42
7a	Shortness of breath S	0·325	0·347	0·058	0·21	0·285	0·18	0·195	0·548
7b	Shortness of breath I	0·376	0·369	0·023	0·208	0·259	0·23	0·124	0·597
8a	Heart palpitations F	0·319	0·316	0·334	0·324	0·347	0·196	0·303	0·434
8b	Heart palpitations S	0·315	0·366	0·25	0·285	0·286	0·291	0·252	0·324
9a	Rash P	0·284	0·035	0·158	0·09	0·149	0·059	0·009	0·473
10a	Hair loss A	0·116	0·231	0·125	0·121	0·189	0·174	0·122	0·084
11a	Itching S	0·201	0·132	0·206	0·202	0·209	0·193	0·056	0·436
12a	Hives P	0·281	0·038	0·133	0·092	0·16	0·147	0·03	0·473
13a	Bed/pressure sores P	0·315	0·007	0·104	0·128	0·014	0·073	0·011	
14a	Radiation skin reaction S	0·09	0·177	0·158	0·121	0·194	0·25	0·085	0·346
15a	Numbness & tingling S	0·396	0·459	0·051	0·204	0·272	0·424	0·235	0·232
15b	Numbness & tingling I	0·37	0·494	0·02	0·226	0·337	0·482	0·304	0·446
16a	Dizziness S	0·252	0·418	0·263	0·257	0·385	0·369	0·393	0·18
16b	Dizziness I	0·262	0·514	0·244	0·253	0·44	0·393	0·43	0·336
17a	Blurred vision S	0·277	0·366	0·235	0·25	0·367	0·296	0·351	0·279
17b	Blurred vision I	0·383	0·391	0·241	0·3	0·414	0·278	0·407	0·318
18a	Flashing lights P	0·337	0·072	0·039	0·163	0·166	0·026	0·148	0·155
19a	Ringing in ears S	0·093	0·196	0·063	0·01	0·125	0·151	0·065	0·221
20a	Concentration S	0·293	0·458	0·422	0·355	0·415	0·398	0·398	0·327
20b	Concentration I	0·287	0·51	0·398	0·343	0·447	0·4	0·446	0·323

NO.	PRO-CTCAE Item	Astrocytoma	Glioblastoma	Oligodendroglioma	IDHmut	Stupp	nonStupp	StandardStupp	StuppTTfields
21a	Memory S	0·434	0·461	0·341	0·391	0·339	0·519	0·296	0·313
21b	Memory I	0·42	0·491	0·393	0·403	0·381	0·499	0·343	0·34
22a	General pain F	0·271	0·384	0·24	0·256	0·199	0·457	0·219	0·183
22b	General pain S	0·335	0·414	0·321	0·329	0·321	0·477	0·345	0·279
22c	General pain I	0·452	0·417	0·213	0·344	0·32	0·475	0·371	0·338
23a	Headache F	0·207	0·294	0·275	0·24	0·173	0·412	0·124	0·32
23b	Headache S	0·323	0·318	0·303	0·312	0·242	0·447	0·234	0·359
23c	Headache I	0·385	0·411	0·227	0·307	0·36	0·471	0·381	0·452
24a	Insomnia S	0·406	0·201	0·413	0·409	0·347	0·185	0·218	0·558
24b	Insomnia I	0·376	0·289	0·27	0·327	0·297	0·316	0·215	0·382
25a	Fatigue S	0·334	0·552	0·507	0·413	0·42	0·455	0·349	0·449
25b	Fatigue I	0·405	0·549	0·44	0·421	0·437	0·459	0·425	0·32
26a	Anxious F	0·223	0·411	0·262	0·241	0·346	0·308	0·328	0·187
26b	Anxious S	0·335	0·473	0·292	0·31	0·393	0·412	0·361	0·275
26c	Anxious I	0·399	0·58	0·262	0·322	0·453	0·49	0·411	0·426
27a	Discouraged F	0·292	0·435	0·291	0·292	0·26	0·397	0·23	0·24
27b	Discouraged S	0·351	0·494	0·3	0·32	0·343	0·467	0·334	0·306
27c	Discouraged I	0·355	0·513	0·224	0·286	0·386	0·46	0·372	0·402
28a	Urinary incontinence F	0·396	0·284	0·188	0·311	0·296	0·235	0·27	0·374
28b	Urinary incontinence I	0·367	0·331	0·178	0·289	0·287	0·287	0·227	0·461
29a	Decreased libido S	0·071	0·085	0·152	0·112	0·08	0·06	0·038	0·164
30a	Decreased sweating P	0·407	0·126	0·014	0·204	0·067	0·09	0·191	0·297
31a	Hot flashes F	0·088	0·245	0·218	0·151	0·337	0·208	0·262	0·391
31b	Hot flashes S	0·196	0·307	0·266	0·231	0·301	0·28	0·295	0·284

Abbreviations: F, Frequency; I, Interference; S, Severity; P, Presence/Absence; A, Amount.

Table S11c. Subgroup Analysis Data (Convergent Validity-Symptom/Function).

NO.	PRO-CTCAE Item	Astrocytoma	Glioblastoma	Oligodendroglioma	IDHmut	Stupp	nonStupp	StandardStupp	StuppTTfields
1a	Difficulty swallowing S	0·1	0·383	0·317	0·196	0·058	0·433	0·023	0·144
2a	Taste changes S	0·19	0·373	0·27	0·222	0·148	0·437	0·044	0·331
3a	Decreased appetite S	0·742	0·848	0·825	0·787	0·793	0·861	0·742	0·898
3b	Decreased appetite I	0·651	0·789	0·665	0·659	0·745	0·787	0·654	0·936
4a	Nausea F	0·754	0·704	0·774	0·764	0·709	0·762	0·689	0·677
4b	Nausea S	0·751	0·755	0·72	0·739	0·754	0·762	0·757	0·679
5a	Vomiting F	0·693	0·791	0·643	0·677	0·718	0·799	0·735	0·61
5b	Vomiting S	0·655	0·685	0·512	0·598	0·606	0·746	0·613	0·468
6a	Constipation S	0·81	0·81	0·676	0·771	0·77	0·866	0·74	0·838
7a	Shortness of breath S	0·295	0·578	0·258	0·297	0·358	0·462	0·394	0·11
7b	Shortness of breath I	0·345	0·595	0·206	0·311	0·448	0·436	0·466	0·349
8a	Heart palpitations F	0·238	0·312	0·202	0·231	0·12	0·355	0·021	0·396
8b	Heart palpitations S	0·261	0·365	0·079	0·194	0·097	0·413	0·028	0·226
9a	Rash P	0·292	0·02	0·026	0·199	0·092	0·059	0·003	0·261
10a	Hair loss A	0·144	0·344	0·348	0·232	0·311	0·292	0·161	0·443
11a	Itching S	0·206	0·176	0·241	0·228	0·152	0·277	0·011	0·279
12a	Hives P	0·201	0·027	0·1	0·166	0·104	0·013	0·028	0·261
13a	Bed/pressure sores P	0·013	0·084	0·022	0·019	0·2	0·08	0·196	
14a	Radiation skin reaction S	0·123	0·119	0·108	0·118	0·064	0·25	0·127	0·48
15a	Numbness & tingling S	0·511	0·569	0·207	0·405	0·504	0·544	0·513	0·169
15b	Numbness & tingling I	0·49	0·626	0·216	0·403	0·569	0·6	0·578	0·343
16a	Dizziness S	0·149	0·433	0·155	0·148	0·404	0·361	0·305	0·6
16b	Dizziness I	0·24	0·459	0·19	0·214	0·441	0·392	0·376	0·528
17a	Blurred vision S	0·201	0·334	0·077	0·131	0·27	0·282	0·225	0·205
17b	Blurred vision I	0·217	0·323	0·132	0·165	0·242	0·284	0·186	0·263
18a	Flashing lights P	0·134	0·051	0·031	0·072	0·145	0·024	0·122	0·125
19a	Ringing in ears S	0·174	0·244	0·028	0·073	0·281	0·302	0·166	0·613
20a	Concentration S	0·591	0·676	0·591	0·587	0·613	0·656	0·525	0·752
20b	Concentration I	0·466	0·67	0·669	0·555	0·572	0·634	0·499	0·745

NO.	PRO-CTCAE Item	Astrocytoma	Glioblastoma	Oligodendroglioma	IDHmut	Stupp	nonStupp	StandardStupp	StuppTTfields
21a	Memory S	0·774	0·83	0·637	0·717	0·831	0·804	0·785	0·925
21b	Memory I	0·26	0·308	0·26	0·278	0·122	0·386	0·164	0·016
22a	General pain F	0·728	0·74	0·628	0·678	0·746	0·761	0·778	0·694
22b	General pain S	0·754	0·758	0·583	0·678	0·758	0·794	0·787	0·689
22c	General pain I	0·762	0·776	0·564	0·685	0·775	0·78	0·817	0·709
23a	Headache F	0·577	0·605	0·415	0·519	0·508	0·653	0·482	0·59
23b	Headache S	0·608	0·631	0·636	0·619	0·576	0·658	0·579	0·613
23c	Headache I	0·401	0·09	0·226	0·31	0·147	0·157	0·201	0·142
24a	Insomnia S	0·726	0·733	0·671	0·697	0·73	0·767	0·702	0·762
24b	Insomnia I	0·626	0·413	0·229	0·453	0·469	0·552	0·323	0·662
25a	Fatigue S	0·804	0·841	0·573	0·697	0·777	0·844	0·666	0·864
25b	Fatigue I	0·384	0·648	0·313	0·343	0·575	0·531	0·474	0·579
26a	Anxious F	0·704	0·759	0·742	0·725	0·722	0·752	0·693	0·629
26b	Anxious S	0·65	0·765	0·775	0·721	0·712	0·778	0·653	0·672
26c	Anxious I	0·643	0·752	0·689	0·669	0·675	0·724	0·648	0·59
27a	Discouraged F	0·513	0·694	0·696	0·608	0·617	0·672	0·572	0·68
27b	Discouraged S	0·589	0·65	0·693	0·649	0·599	0·681	0·504	0·748
27c	Discouraged I	0·419	0·484	0·259	0·339	0·311	0·548	0·155	0·7
28a	Urinary incontinence F	0·635	0·406	0·075	0·473	0·386	0·429	0·305	0·702
28b	Urinary incontinence I	0·134	0·393	0·15	0·123	0·21	0·298	0·05	0·612
29a	Decreased libido S	0·038	0·058	0·112	0·025	0·045	0·09	0·133	0·141
30a	Decreased sweating P	0·192	0·182	0·131	0·166	0·016	0·202	0·087	0·125
31a	Hot flashes F	0·259	0·257	0·143	0·216	0·202	0·426	0·007	0·612
31b	Hot flashes S	0·341	0·322	0·169	0·267	0·191	0·48	0·053	0·472

Abbreviations: F, Frequency; I, Interference; S, Severity; P, Presence/Absence; A, Amount.

Table S11d. Subgroup Analysis Data (Known-Groups Validity).

NO.	PRO-CTCAE Item	Astrocytoma	Glioblastoma	Oligodendroglioma	IDHmut	Stupp	nonStupp	StandardStupp	StuppTTfields
1a	Difficulty swallowing S	0·0048088	0·541338857	0·351440311	0·135326446	0·279342205	0·341029925	0·231919328	0·201594628
2a	Taste changes S	0·68102334	0·650649847	0·221485436	0·472518773	0·582941988	0·475434743	0·418059881	1·112929546
3a	Decreased appetite S	0·731874594	0·583668081	0·036063699	0·414091964	0·522217144	0·48063426	0·152008489	1·99127139
3b	Decreased appetite I	0·62149006	0·527446257	0·195173951	0·405174065	0·448625228	0·406302431	0·170870432	1·235176096
4a	Nausea F	0·26663737	0·543405165	0·019630996	0·231252402	0·329564122	0·411390734	0·006469236	1·267589796
4b	Nausea S	0·430470391	0·486824167	0·349681609	0·437230068	0·553917605	0·421938237	0·273834189	1·593887391
5a	Vomiting F	0·422414328	0·387515868	0·250722694	0·430149211	0·535205481	0·382082038	0·337181368	1·166350741
5b	Vomiting S	0·422366665	0·353535276	0·039642739	0·429925599	0·38782205	0·442175768	0·234648066	1·132284843
6a	Constipation S	0·509192305	0·772096557	0·045231605	0·451459637	0·789472935	0·614092712	0·507147474	1·611100744
7a	Shortness of breath S	0·360740779	0·342405783	0·781748948	0·534970008	0·424147988	0·417370091	0·282593622	0·849577069
7b	Shortness of breath I	0·316670733	0·35661084	0·840208696	0·515959888	0·37336366	0·418336535	0·202816685	0·828008682
8a	Heart palpitations F	0·104921636	0·377451881	0·708632558	0·341151195	0·479308067	0·221347265	0·231919328	1·389020035
8b	Heart palpitations S	0·195303763	0·425228193	0·920481134	0·449614111	0·653088621	0·24292424	0·360555128	1·703094391
9a	Rash P	0·599230134	0·161185784	0·04363811	0·467270212	0·10301249	0·35684759	0·152752523	0·849577069
10a	Hair loss A	0·380426729	0·548065903	0·189514896	0·286118659	0·591496592	0·388735966	0·336223505	1·038745418
11a	Itching S	0·137729279	0·284935543	0·116330407	0·126789601	0·340927831	0·340329065	0·006799193	1·235176096
12a	Hives P	0·572203544	0·032270002	0·04363811	0·410893179	0·217424058	0·127705881	0	0·849577069
13a	Bed/pressure sores P	0·254920247	0·037903815	0·192661457	0·120983625	0·176950564	0·114004131	0·158795901	0
14a	Radiation skin reaction S	0·514756417	0·405086974	0·087134423	0·358112756	0·436094508	0·363366614	0·030348849	2·354487647
15a	Numbness & tingling S	0·303279073	0·990369105	0·431984256	0·382025797	0·871179489	0·798545035	0·731563521	1·350410096
15b	Numbness & tingling I	0·394008928	1·005802754	0·526619908	0·490765416	1·196924456	0·81306153	1·071701306	2·34520788
16a	Dizziness S	0·451483695	0·558305181	0·308752812	0·401742744	0·937991431	0·384900023	0·826421789	1·038745418
16b	Dizziness I	0·388028864	0·728690784	0·491923454	0·440443159	0·948304921	0·492072334	0·90298364	0·789059485
17a	Blurred vision S	0·474678053	0·473486498	0·019895043	0·232246555	0·469142713	0·217752836	0·43203886	0·25533109
17b	Blurred vision I	0·282539683	0·408881361	0·166070496	0·189956442	0·17101952	0·326167481	0·130104512	0·009967342
18a	Flashing lights P	0·245537609	0·289566778	0·142806022	0·22501762	0·31500726	0·192888947	0·123358791	1·048808848
19a	Ringing in ears S	0·18513319	0·019492436	0·260769049	0·205883156	0·131812955	0·195658711	0·119299477	1·112929546
20a	Concentration S	0·174928395	0·905842974	0·928239143	0·391786955	0·959150057	0·694924048	0·70924863	1·539461913
20b	Concentration I	0·296665046	0·978827803	0·854850414	0·449988289	1·080732792	0·63399892	0·895156806	1·593887391

NO.	PRO-CTCAE Item	Astrocytoma	Glioblastoma	Oligodendroglioma	IDHmut	Stupp	nonStupp	StandardStupp	StuppTTfields
21a	Memory S	0·370959002	0·953159247	0·377203156	0·374806855	1·025899799	0·797215732	0·734962621	1·911521504
21b	Memory I	0·403547494	0·960856832	0·154375613	0·371817263	1·043323632	0·734530353	0·836660027	1·668921336
22a	General pain F	0·198359866	0·378616513	0·600651974	0·359251087	0·261262366	0·581227828	0·437149344	0·157755754
22b	General pain S	0·204065868	0·464631151	0·853029645	0·445310549	0·312307905	0·637433011	0·352783687	0·179937835
22c	General pain I	0·368716972	0·532668512	0·480999442	0·425453555	0·408485872	0·640639277	0·608654245	0·083939761
23a	Headache F	0·066945374	0·502847404	0·23988009	0·099203743	0·292891881	0·723303742	0·364646449	0·21164119
23b	Headache S	0·158501643	0·391082079	0·476853664	0·328256947	0·305376225	0·685519377	0·503232122	0·014114447
23c	Headache I	0·285114876	0·511135622	0·335662387	0·365449722	0·575594889	0·580454105	0·863636364	0·189240541
24a	Insomnia S	0·531992682	0·400773126	0·490290338	0·526897609	0·964734799	0·449642357	0·547527047	3·336935731
24b	Insomnia I	0·662988026	0·543904493	0·450801246	0·581785418	0·994599772	0·680533008	0·698245764	2·209007928
25a	Fatigue S	0·601338676	0·839836379	0·619314267	0·569387325	0·891457211	0·71381835	0·400687483	2·431679357
25b	Fatigue I	0·829051739	0·802401695	1·104869806	0·91680116	1·029410712	0·685770948	0·704444097	1·842249258
26a	Anxious F	0·339374827	0·659879089	0·906308497	0·492578723	0·817810788	0·546980062	0·572306999	1·290427587
26b	Anxious S	0·3552351	0·633095503	0·946666191	0·503417312	0·892931262	0·582988902	0·616793955	1·593887391
26c	Anxious I	0·432800431	0·692921635	1·078072465	0·634965979	0·886925739	0·503217586	0·632455532	1·528678803
27a	Discouraged F	0·226662697	0·660306768	0·836299704	0·443248744	0·647284715	0·552594095	0·082803268	2·510030036
27b	Discouraged S	0·357877671	0·638102137	0·832269341	0·507062267	0·810540984	0·612616834	0·170216068	3·185508271
27c	Discouraged I	0·605549859	0·635899252	1·101782709	0·756603495	0·981289365	0·592474027	0·463778944	2·947913494
28a	Urinary incontinence F	0·718733276	0·643537782	0·604470525	0·742727268	1·056646284	0·534909466	0·534670655	4·028859307
28b	Urinary incontinence I	0·650284544	0·708013984	0·381638734	0·633095767	1·154101219	0·549779494	0·665925269	4·028859307
29a	Decreased libido S	0·151607004	0·15304956	0·064416444	0·121606084	0·251229241	0·038682008	0·067366443	1·057071879
30a	Decreased sweating P	0·471889675	0·019223841	0·200794179	0·389392095	0·036303305	0·123939799	0·208841639	1·048808848
31a	Hot flashes F	0·296055623	0·314713772	0·054246612	0·189263906	0·694074863	0·151220031	0·202585007	3·026684484
31b	Hot flashes S	0·357422485	0·311138995	0·175628385	0·147440655	0·714708138	0·088326882	0·340743337	1·709550465

Abbreviations: F, Frequency; I, Interference; S, Severity; P, Presence/Absence; A, Amount.

Table S11e. Subgroup Analysis Data (Global Responsiveness)

NO.	PRO-CTCAE Item	Astrocytoma	Glioblastoma	Oligodendroglioma	IDHmut	Stupp	nonStupp
1a	Difficulty swallowing S	-0·287297202	0·437882279	0	-0·204348334	0	0·659532212
2a	Taste changes S	0·191768366	0·375013736	-0·608580619	-0·117550763	0·296318879	0·091007637
3a	Decreased appetite S	-0·160049254	0·756111427	0·442807443	0	0·632455532	0·516615383
3b	Decreased appetite I	0·155808784	0·722375334	0·442807443	0·232992949	0·603361371	0·664770029
4a	Nausea F	-0·232013421	0·446135657	-0·442807443	-0·3082207	-0·271052371	0·35323814
4b	Nausea S	0	0·444706465	0	0	0·379473319	0·273022912
5a	Vomiting F	0	0·305246659	-1·195228609	-0·496027384	-0·379473319	0·233735261
5b	Vomiting S	-0·225567343	0·366471377	-0·816496581	-0·350115188	-0·379473319	0·091405053
6a	Constipation S	0	0·504207936	0·608580619	0·131725465	-0·379473319	0·61354744
7a	Shortness of breath S	0·769055765	0·649538889	0	0·605636332	1·054092553	0·604624346
7b	Shortness of breath I	0·9141741	0·642322392	-0·816496581	0·605636332	0·888956637	0·704751061
8a	Heart palpitations F	0·461433459	0·381245509	0·608580619	0·487339717	0	0·478443082
8b	Heart palpitations S	0·377964473	0·531440909	0	0·290593263	0	0·639602149
9a	Rash P	0·984349582	0·240902798	-1·825741858	0·587753814	0	0·077665197
10a	Hair loss A	-0·562955217	0·437061226	-0·250784931	-0·460893003	0·566385098	0·666314213
11a	Itching S	0·346143999	0·051350989	-0·816496581	0·139596488	-0·848528137	-0·095688616
12a	Hives P	0·4062996	-0·14509525	-1·825741858	0·082745676	0	-1·477097892
13a	Bed/pressure sores P	-0·407812825	-0·899781664	-1·825741858	-0·64978629	0	-1·78376517
14a	Radiation skin reaction S	-0·191768366	0·451271948	-0·816496581	-0·304438708	0·282842712	0·34542464
15a	Numbness & tingling S	0·519740195	0·628705807	-1·290994449	0·195917938	0·242932899	0·655909803
15b	Numbness & tingling I	0·211542975	0·646838551	-0·816496581	0·08157813	0·543214476	0·691922305
16a	Dizziness S	0·429753697	0·528609055	0	0·342820322	1·005602285	0·374321739
16b	Dizziness I	0·429753697	0·790881198	0·816496581	0·43319086	0·943975163	0·662950181
17a	Blurred vision S	0·36421568	0·575353976	0·816496581	0·381486454	1·144155107	0·583295828
17b	Blurred vision I	0·155808784	0·617929563	0·816496581	0·223606798	1·176696811	0·777727771
18a	Flashing lights P	0·210280021	-0·971989145	-1·825741858	-0·084594969	-1·897366596	-0·604624346
19a	Ringing in ears S	0·6094494	0·444074167	0	0·487339717	0·632455532	0·461994302
20a	Concentration S	0·246758849	0·288603643	1·328422328	0·64978629	0	0·189790893
20b	Concentration I	0·587047228	0·279656159	1·290994449	0·745355992	-0·296318879	0·436495172
21a	Memory S	-0·27817432	0·586939186	0·816496581	-0·112734334	0	0·306833748
21b	Memory I	-0·237227857	0·632525313	0·816496581	-0·096154782	0·159787092	0·561482608
22a	General pain F	0·130830134	0·226795147	-0·285132974	0	0·32071349	0·463435785
22b	General pain S	0·132052872	0·390266761	-0·442807443	0	0·943975163	0·506404036
22c	General pain I	0·111830284	0·092519518	0	0·085899015	0·141030148	0·28130642
23a	Headache F	0·297380857	-0·150043093	-0·398409536	0·107146454	-0·235339362	0·532913684
23b	Headache S	0·178174161	-0·121923779	-0·816496581	0	0	0·394771017
23c	Headache I	0·442141066	0	-0·816496581	0·165342461	-0·632455532	0·535129551
24a	Insomnia S	0·708470537	0·194665705	0	0·535732268	0	0·69084928
24b	Insomnia I	0·876317573	0·353188988	0	0·700922874	0·271052371	0·893954324
25a	Fatigue S	0·779043921	0·632930884	0·816496581	0·750025176	0	0·722595684
25b	Fatigue I	0·679688042	0·549452676	0	0·476162063	0·242932899	0·420048728

NO.	PRO-CTCAE Item	Astrocytoma	Glioblastoma	Oligodendroglioma	IDHmut	Stupp	nonStupp
26a	Anxious F	0·312640946	0·047516507	0·339031752	0·321439361	-0·617394907	0
26b	Anxious S	0·575305097	-0·167524795	0	0·363381799	-0·632455532	-0·098567111
26c	Anxious I	0·661040687	-0·05213339	0·285132974	0·476162063	-0·424264069	0·163977451
27a	Discouraged F	0·615636862	0·534590721	0	0·4549414	0	0·497212636
27b	Discouraged S	0·732629845	0·306143126	0	0·506028349	-0·6	0·35237553
27c	Discouraged I	0·83452296	0·489115988	0	0·581905372	-0·6	0·710297972
28a	Urinary incontinence F	0·474455715	0·158133918	0·816496581	0·476162063	0	0·388325984
28b	Urinary incontinence I	0·670981706	0·171285971	0	0·54592968	0	0·535129551
29a	Decreased libido S	-0·853845769	-1·195636246	-1·208080899	-0·99546385	-1·180740454	-1·534719128
30a	Decreased sweating P	1·043021113	-0·276999033	-1·825741858	0·6440863	-1·549193338	0·154478595
31a	Hot flashes F	0	0	0	0	0·372104204	0·101722054
31b	Hot flashes S	0·393397896	0	0	0·27296484	0	0·347237905

Abbreviations: F, Frequency; I, Interference; S, Severity; P, Presence/Absence; A, Amount.

Table S11f. Subgroup Analysis Data (Specific Responsiveness).

NO.	PRO-CTCAE Item	Astrocytoma	Glioblastoma	Oligodendroglioma	IDHmut	Stupp	nonStupp
1a	Difficulty swallowing S	0	0·252151121	-1	-0·261116484	-0·131725465	0·452444342
2a	Taste changes S	0	0·270118085	-1·192079121	-0·411822765	0·134199399	0·131384568
3a	Decreased appetite S	-0·235339362	0·39679235	0·282842712	0	0	0·327498886
3b	Decreased appetite I	0	0·477505238	0	0	-0·114160332	0·482903882
4a	Nausea F	-0·268133222	0·122443128	-0·522232968	-0·365962527	-0·48077391	0·229363706
4b	Nausea S	0·249292785	0·28860122	0	0·183803656	0·109833929	0·34739384
5a	Vomiting F	0	0·201093493	-1	-0·403786427	-0·149071198	0·323972805
5b	Vomiting S	0	0·087079392	0	0	-0·249589827	0·146974453
6a	Constipation S	-0·249292785	0·215033408	1	0	-0·608877416	0·483277402
7a	Shortness of breath S	0·737027731	0·53669859	-1	0·460721338	0·670996995	0·548834097
7b	Shortness of breath I	0·925579721	0·414766687	-1	0·620173673	0·613045003	0·502950794
8a	Heart palpitations F	0·6770032	0·253661021	1	0·783349452	0	0·28235631
8b	Heart palpitations S	0·6770032	0·28824351	0	0·559016994	0	0·244738084
9a	Rash P	1·164638561	0·481039	0·626907569	0·986715755	-0·167053814	0·92148442
10a	Hair loss A	0·586052851	0·304404503	0	0·389069055	0·641426981	0·757699689
11a	Itching S	0·588167459	0·492592183	0·507092553	0·561854936	-0·210818511	0·73678208
12a	Hives P	0·573334096	0·134586856	0·626907569	0·606835046	-0·167053814	0·639175036
13a	Bed/pressure sores P	-0·5	-0·211245702	-0·167053814	-0·363178028	0·401489763	-1·738453975
14a	Radiation skin reaction S	0·306186218	0·386421101	1·144155107	0·606835046	0	0·647883544
15a	Numbness & tingling S	0·429368771	0·521083749	-1	0·22981928	0·298142397	0·634647759
15b	Numbness & tingling I	0·115470054	0·403239759	-1	0	0·407454804	0·576242858
16a	Dizziness S	0·349602949	0·389056248	-1	0·146385011	0·479898979	0·392024355
16b	Dizziness I	0·323669437	0·569319823	1	0·396202908	0·560144149	0·608811044
17a	Blurred vision S	0	0·305880314	1	0·198679854	0·619894881	0·309945564
17b	Blurred vision I	-0·19245009	0·28107997	1	0	0·743456302	0·371346524
18a	Flashing lights P	-0·146432562	-0·531970496	-1·732050808	-0·369648044	-1·763261441	0
19a	Ringing in ears S	0·368513866	0·257745768	0	0·310086836	0·530548284	0·214550101
20a	Concentration S	0·352332132	0·258705789	0·8705715	0·658627326	-0·346143999	0·692396869
20b	Concentration I	0·6	0·371118496	0·8705715	0·761354725	0	0·707106781
21a	Memory S	-0·167053814	0·358314549	0·632455532	0·107146454	-0·55634864	0·692396869
21b	Memory I	-0·141030148	0·376162296	0·632455532	0·093464604	-0·360655323	0·692396869
22a	General pain F	-0·157567719	0·150464919	-0·372104204	-0·261795864	0·259870097	0·370639633
22b	General pain S	0	0·329562714	-0·813157113	-0·288464346	0·562236091	0·505912052
22c	General pain I	-0·139497166	0·077985771	-0·685248289	-0·34063255	0·117506398	0·33211456
23a	Headache F	0	-0·082748531	-0·282842712	-0·114273441	-0·575305097	0·863844725
23b	Headache S	0	-0·051321911	0	0	-0·246758849	0·506083518
23c	Headache I	0	0·15314403	0	0	0·232013421	0·461337784
24a	Insomnia S	0·517463617	0·084929466	0·210818511	0·387936914	-0·250906347	0·915617914
24b	Insomnia I	0·641426981	0·156580571	0·210818511	0·458958621	0	0·865770803
25a	Fatigue S	1·054092553	0·531036722	0·572077554	0·750025176	0·335490853	0·915617914
25b	Fatigue I	0·828078671	0·418152114	0	0·389871774	0·160049254	0·561083608

NO.	PRO-CTCAE Item	Astrocytoma	Glioblastoma	Oligodendroglioma	IDHmut	Stupp	nonStupp
26a	Anxious F	0·685248289	0·40946981	0·566385098	0·581905372	0·178174161	0·592532353
26b	Anxious S	0·848528137	0·241492119	0·55549206	0·584807661	0	0·647883544
26c	Anxious I	0·728798697	0·264725373	0·681554201	0·587753814	0·178174161	0·589015089
27a	Discouraged F	0·592637758	0·495194297	0·159787092	0·379681202	0·377964473	0·651920241
27b	Discouraged S	0·744208408	0·331823892	0·172487872	0·422974847	-0·232013421	0·740531631
27c	Discouraged I	0·744208408	0·290191163	0·145951277	0·399579611	-0·661040687	0·73678208
28a	Urinary incontinence F	0·52605091	0·12912379	1	0·549041843	-0·295561029	0·350050298
28b	Urinary incontinence I	0·737027731	0·138339203	0	0·620173673	-0·295561029	0·550697715
29a	Decreased libido S	-0·809494224	-1·133107409	-1·609325342	-1·213276776	-1·875845674	-1·146255501
30a	Decreased sweating P	0·876037591	-0·027776109	-1·732050808	0·573334096	-1·591644852	0
31a	Hot flashes F	0·19245009	0·06067506	0·522232968	0·291937104	0·557592226	0·142282564
31b	Hot flashes S	0·428174419	0·075304223	0·522232968	0·472768372	0·670996995	0·244738084

Abbreviations: F, Frequency; I, Interference; S, Severity; P, Presence/Absence; A, Amount.

Supplementary Scale. Customized PRO-CTCAE Scale (English Version).

Customized PRO-CTCAE Scale
for Adult-Type Diffuse Gliomas

Item subset derived from PRO-CTCAE® Item Library Version 1.0

English

https://healthcaredelivery.cancer.gov/pro-ctcae/builder.html

As individuals go through treatment for their cancer they sometimes experience different symptoms and side effects. For each question, please select the one response that best describes your experiences over the past 7 days…

**1a.** In the last 7 days, what was the SEVERITY of your DIFFICULTY SWALLOWING at its WORST?				
O None	O Mild	O Moderate	O Severe	O Very severe

**2a.** In the last 7 days, what was the SEVERITY of your PROBLEMS WITH TASTING FOOD OR DRINK at their WORST?				
O None	O Mild	O Moderate	O Severe	O Very severe

**3a.** In the last 7 days, what was the SEVERITY of your DECREASED APPETITE at its WORST?				
O None	O Mild	O Moderate	O Severe	O Very severe
**3b.** In the last 7 days, how much did DECREASED APPETITE INTERFERE with your usual or daily activities?				
O Not at all	O A little bit	O Somewhat	O Quite a bit	O Very much

**4a.** In the last 7 days, how OFTEN did you have NAUSEA?				
O Never	O Rarely	O Occasionally	O Frequently	O Almost constantly
**4b.** In the last 7 days, what was the SEVERITY of your NAUSEA at its WORST?				
O None	O Mild	O Moderate	O Severe	O Very severe

**5a.** In the last 7 days, how OFTEN did you have VOMITING?				
O Never	O Rarely	O Occasionally	O Frequently	O Almost constantly
**5b.** In the last 7 days, what was the SEVERITY of your VOMITING at its WORST?				
O None	O Mild	O Moderate	O Severe	O Very severe

**6a.** In the last 7 days, what was the SEVERITY of your CONSTIPATION at its WORST?				
O None	O Mild	O Moderate	O Severe	O Very severe

**7a.** In the last 7 days, what was the SEVERITY of your SHORTNESS OF BREATH at its WORST?				
O None	O Mild	O Moderate	O Severe	O Very severe
**7b.** In the last 7 days, how much did your SHORTNESS OF BREATH INTERFERE with your usual or daily activities?				
O Not at all	O A little bit	O Somewhat	O Quite a bit	O Very much

**8a.** In the last 7 days, how OFTEN did you feel a POUNDING OR RACING HEARTBEAT (PALPITATIONS)?				
O Never	O Rarely	O Occasionally	O Frequently	O Almost constantly
**8b.** In the last 7 days, what was the SEVERITY of your POUNDING OR RACING HEARTBEAT (PALPITATIONS) at its WORST?				
O None	O Mild	O Moderate	O Severe	O Very severe

**9a.** In the last 7 days, did you have any RASH?	
O Yes	O No

**10a.** In the last 7 days, did you have any HAIR LOSS?				
O Not at all	O A little bit	O Somewhat	O Quite a bit	O Very much

**11a.** In the last 7 days, what was the SEVERITY of your ITCHY SKIN at its WORST?				
O None	O Mild	O Moderate	O Severe	O Very severe

**12a.** In the last 7 days, did you have any HIVES (ITCHY RED BUMPS ON THE SKIN)?	
O Yes	O No

**13a.** In the last 7 days, did you have any BED SORES?	
O Yes	O No

**14a.** In the last 7 days, what was the SEVERITY of your SKIN BURNS FROM RADIATION at their WORST?					
O None	O Mild	O Moderate	O Severe	O Very severe	O Not applicable

**15a.** In the last 7 days, what was the SEVERITY of your NUMBNESS OR TINGLING IN YOUR HANDS OR FEET at its WORST?				
O None	O Mild	O Moderate	O Severe	O Very severe
**15b.** In the last 7 days, how much did NUMBNESS OR TINGLING IN YOUR HANDS OR FEET INTERFERE with your usual or daily activities?				
O Not at all	O A little bit	O Somewhat	O Quite a bit	O Very much

**16a.** In the last 7 days, what was the SEVERITY of your DIZZINESS at its WORST?				
O None	O Mild	O Moderate	O Severe	O Very severe
**16b.** In the last 7 days, how much did DIZZINESS INTERFERE with your usual or daily activities?				
O Not at all	O A little bit	O Somewhat	O Quite a bit	O Very much

**17a.** In the last 7 days, what was the SEVERITY of your BLURRY VISION at its WORST?				
O None	O Mild	O Moderate	O Severe	O Very severe
**17b.** In the last 7 days, how much did BLURRY VISION INTERFERE with your usual or daily activities?				
O Not at all	O A little bit	O Somewhat	O Quite a bit	O Very much

**18a.** In the last 7 days, did you have any FLASHING LIGHTS IN FRONT OF YOUR EYES?	
O Yes	O No

**19a.** In the last 7 days, what was the SEVERITY of RINGING IN YOUR EARS at its WORST?				
O None	O Mild	O Moderate	O Severe	O Very severe

**20a.** In the last 7 days, what was the SEVERITY of your PROBLEMS WITH CONCENTRATION at their WORST?				
O None	O Mild	O Moderate	O Severe	O Very severe
**20b.** In the last 7 days, how much did PROBLEMS WITH CONCENTRATION INTERFERE with your usual or daily activities?				
O Not at all	O A little bit	O Somewhat	O Quite a bit	O Very much

**21a.** In the last 7 days, what was the SEVERITY of your PROBLEMS WITH MEMORY at their WORST?				
O None	O Mild	O Moderate	O Severe	O Very severe
**21b.** In the last 7 days, how much did PROBLEMS WITH MEMORY INTERFERE with your usual or daily activities?				
O Not at all	O A little bit	O Somewhat	O Quite a bit	O Very much

**22a.** In the last 7 days, how OFTEN did you have PAIN?				
O Never	O Rarely	O Occasionally	O Frequently	O Almost constantly
**22b.** In the last 7 days, what was the SEVERITY of your PAIN at its WORST?				
O None	O Mild	O Moderate	O Severe	O Very severe
**22c.** In the last 7 days, how much did PAIN INTERFERE with your usual or daily activities?				
O Not at all	O A little bit	O Somewhat	O Quite a bit	O Very much

**23a.** In the last 7 days, how OFTEN did you have a HEADACHE?				
O Never	O Rarely	O Occasionally	O Frequently	O Almost constantly
**23b.** In the last 7 days, what was the SEVERITY of your HEADACHE at its WORST?				
O None	O Mild	O Moderate	O Severe	O Very severe
**23c.** In the last 7 days, how much did your HEADACHE INTERFERE with your usual or daily activities?				
O Not at all	O A little bit	O Somewhat	O Quite a bit	O Very much

**24a.** In the last 7 days, what was the SEVERITY of your INSOMNIA (INCLUDING DIFFICULTY FALLING ASLEEP, STAYING ASLEEP, OR WAKING UP EARLY) at its WORST?				
O None	O Mild	O Moderate	O Severe	O Very severe
**24b.** In the last 7 days, how much did INSOMNIA (INCLUDING DIFFICULTY FALLING ASLEEP, STAYING ASLEEP, OR WAKING UP EARLY) INTERFERE with your usual or daily activities?				
O Not at all	O A little bit	O Somewhat	O Quite a bit	O Very much

**25a.** In the last 7 days, what was the SEVERITY of your FATIGUE, TIREDNESS, OR LACK OF ENERGY at its WORST?				
O None	O Mild	O Moderate	O Severe	O Very severe
**25b.** In the last 7 days, how much did FATIGUE, TIREDNESS, OR LACK OF ENERGY INTERFERE with your usual or daily activities?				
O Not at all	O A little bit	O Somewhat	O Quite a bit	O Very much

**26a.** In the last 7 days, how OFTEN did you feel ANXIETY?				
O Never	O Rarely	O Occasionally	O Frequently	O Almost constantly
**26b.** In the last 7 days, what was the SEVERITY of your ANXIETY at its WORST?				
O None	O Mild	O Moderate	O Severe	O Very severe
**26c.** In the last 7 days, how much did ANXIETY INTERFERE with your usual or daily activities?				
O Not at all	O A little bit	O Somewhat	O Quite a bit	O Very much

**27a.** In the last 7 days, how OFTEN did you FEEL THAT NOTHING COULD CHEER YOU UP?				
O Never	O Rarely	O Occasionally	O Frequently	O Almost constantly
**27b.** In the last 7 days, what was the SEVERITY of your FEELINGS THAT NOTHING COULD CHEER YOU UP at their WORST?				
O None	O Mild	O Moderate	O Severe	O Very severe
**27c.** In the last 7 days, how much did FEELING THAT NOTHING COULD CHEER YOU UP INTERFERE with your usual or daily activities?				
O Not at all	O A little bit	O Somewhat	O Quite a bit	O Very much

**28a.** In the last 7 days, how OFTEN did you have LOSS OF CONTROL OF URINE (LEAKAGE)?				
O Never	O Rarely	O Occasionally	O Frequently	O Almost constantly
**28b.** In the last 7 days, how much did LOSS OF CONTROL OF URINE (LEAKAGE) INTERFERE with your usual or daily activities?				
O Not at all	O A little bit	O Somewhat	O Quite a bit	O Very much

**29a.** In the last 7 days, what was the SEVERITY of your DECREASED SEXUAL INTEREST at its WORST?						
O None	O Mild	O Moderate	O Severe	O Very severe	O Not sexually active	O Prefer not to answer

**30a.** In the last 7 days, did you have an UNEXPECTED DECREASE IN SWEATING?	
O Yes	O No

**31a.** In the last 7 days, how OFTEN did you have HOT FLASHES/FLUSHES?				
O Never	O Rarely	O Occasionally	O Frequently	O Almost constantly
**31b.** In the last 7 days, what was the SEVERITY of your HOT FLASHES/FLUSHES at their WORST?				
O None	O Mild	O Moderate	O Severe	O Very severe

OT HER SYMPTOMS	
Do you have any other symptoms that you wish to report?	
O Yes	O No
**Please list any other symptoms:**	
1.	In the last 7 days, what was the SEVERITY of this symptom at its WORST?<br>O None O Mild O Moderate O Severe O Very Severe
2.	In the last 7 days, what was the SEVERITY of this symptom at its WORST?<br>O None O Mild O Moderate O Severe O Very Severe
3.	In the last 7 days, what was the SEVERITY of this symptom at its WORST?<br>O None O Mild O Moderate O Severe O Very Severe
4.	In the last 7 days, what was the SEVERITY of this symptom at its WORST?<br>O None O Mild O Moderate O Severe O Very Severe
5.	In the last 7 days, what was the SEVERITY of this symptom at its WORST?<br>O None O Mild O Moderate O Severe O Very Severe

Supplementary Scale. Customized PRO-CTCAE Scale (Simplified Chinese Version).

Customized PRO-CTCAE Scale
for Adult-Type Diffuse Gliomas

Item subset derived from PRO-CTCAE® Item Library Version 1.0
Chinese (Simplified)
https://healthcaredelivery.cancer.gov/pro-ctcae/builder.html

病人进行癌症治疗时，会经历不同症状和副作用。请仔细阅读以下问题，并选择一个最能准确描述你过去七天的经历的答案。

**1a**. 在过去的 7 天里，**吞咽困难**在**最严重**时的程度：				
O 没有	O 轻微	O 中度	O 严重	O 很严重

**2a**. 在过去的 7 天里，**品尝食物或饮料时出现的味觉问题**在**最严重**时的程度：				
O 没有	O 轻微	O 中度	O 严重	O 很严重

**3a**. 在过去的 7 天里，**食欲下降**在**最严重**时的程度：				
O 没有	O 轻微	O 中度	O 严重	O 很严重
**3b**. 在过去的 7 天里，**食欲下降影响**你日常活动的程度：				
O 没有	O 少许	O 有些	O 蛮多	O 很多

**4a**. 在过去的 7 天里，**作呕(恶心/反胃)** 是否**常常**出现：				
O 从来没有	O 很少	O 偶尔	O 经常	O 几乎是持续的
**4b**. 在过去的 7 天里，**作呕(恶心/反胃)**在**最严重**时的程度：				
O 没有	O 轻微	O 中度	O 严重	O 很严重

**5a**. 在过去的 7 天里，**呕吐**是否**常常**出现：				
O 从来没有	O 很少	O 偶尔	O 经常	O 几乎是持续的
**5b**. 在过去的 7 天里，**呕吐**在**最严重**时的程度：				
O 没有	O 轻微	O 中度	O 严重	O 很严重

**6a**. 在过去的 7 天里，**便秘**在**最严重**时的程度：				
O 没有	O 轻微	O 中度	O 严重	O 很严重

**7a**. 在过去的 7 天里，**呼吸急促**在**最严重**时的程度：				
O 没有	O 轻微	O 中度	O 严重	O 很严重
**7b**. 在过去的 7 天里，**呼吸急促影响**你日常活动的程度：				
O 没有	O 少许	O 有些	O 蛮多	O 很多

**8a**. 在过去的 7 天里，你是否**常常**感到**心跳怦怦直跳或心跳加速（心悸）：**				
O 从来没有	O 很少	O 偶尔	O 经常	O 几乎是持续的
**8b**. 在过去的 7 天里，**心跳怦怦直跳或心跳加速（心悸）**在**最严重**时的程度:				
O 没有	O 轻微	O 中度	O 严重	O 很严重

**9a**. 在过去的 7 天里，你是否有任何**皮肤红疹**：	
O 是	O 否

**10a**. 在过去的 7 天里，你是否有**脱发：**				
O 没有	O 少许	O 有些	O 蛮多	O 很多

**11a**. 在过去的 7 天里，**皮肤瘙痒**在**最严重**时的程度：				
O 没有	O 轻微	O 中度	O 严重	O 很严重

**12a**. 在过去的 7 天里，你是否有任何**麻疹**（皮肤上发痒的红疙瘩）：	
O 是	O 否

**13a**. 在过去的 7 天里，你是否有**褥疮/压疮** (皮肤和皮下组织在长时间受压下所出现的损伤)：	
O 是	O 否

**14a**. 在过去的 7 天里，**皮肤被辐射线灼伤**在**最严重**时的程度：					
O 没有	O 轻微	O 中度	O 严重	O 很严重	O 不适用

**15a**. 在过去的 7 天里，**手或脚出现麻痹或有针刺感**在**最严重**时的程度：				
O 没有	O 轻微	O 中度	O 严重	O 很严重
**15b**. 在过去的 7 天里，**手或脚出现麻痹或有针刺感影响**你日常活动的程度：				
O 没有	O 少许	O 有些	O 蛮多	O 很多

**16a**. 在过去的 7 天里，**头晕**在**最严重**时的程度：				
O 没有	O 轻微	O 中度	O 严重	O 很严重
**16b**. 在过去的 7 天里，**头晕影响**你日常活动的程度：				
O 没有	O 少许	O 有些	O 蛮多	O 很多

**17a**. 在过去的 7 天里，**视力模糊**在**最严重**时的程度：				
O 没有	O 轻微	O 中度	O 严重	O 很严重
**17b**. 在过去的 7 天里，**视力模糊影响**你日常活动的程度：				
O 没有	O 少许	O 有些	O 蛮多	O 很多

**18a**. 在过去的 7 天里，你是否**眼前出现闪光感：**	
O 是	O 否

**19a**. 在过去的 7 天里，**耳鸣** (耳朵有响杂声音) 在**最严重**时的程度：				
O 没有	O 轻微	O 中度	O 严重	O 很严重

**20a**. 在过去的 7 天里，**不能集中注意力**在**最严重**时的程度：				
O 没有	O 轻微	O 中度	O 严重	O 很严重
**20b**. 在过去的 7 天里，**不能集中注意力影响**你日常活动的程度：				
O 没有	O 少许	O 有些	O 蛮多	O 很多

**21a.** 在过去的 7 天里，**记忆力 (记性) 问题**在**最严重**时的程度：				
O 没有	O 轻微	O 中度	O 严重	O 很严重
**21b**. 在过去的 7 天里，**记忆力 (记性) 问题影响**你日常活动的程度：				
O 没有	O 少许	O 有些	O 蛮多	O 很多

**22a**. 在过去的 7 天里，**疼痛** （可出现在身体任何部位） 是否**常常**出现：				
O 从来没有	O 很少	O 偶尔	O 经常	O 几乎是持续的
**22b**. 在过去的 7 天里，**疼痛** （可出现在身体任何部位）在**最严重**时的程度：				
O 没有	O 轻微	O 中度	O 严重	O 很严重
**22c**. 在过去的 7 天里，**疼痛** （可出现在身体任何部位）**影响**你日常活动的程度：				
O 没有	O 少许	O 有些	O 蛮多	O 很多

**23a**. 在过去的 7 天里，**头痛**是否**常常**出现：				
O 从来没有	O 很少	O 偶尔	O 经常	O 几乎是持续的
**23b**. 在过去的 7 天里，**头痛**在**最严重**时的程度：				
O 没有	O 轻微	O 中度	O 严重	O 很严重
**23c**. 在过去的 7 天里，**头痛影响**你日常活动的程度：				
O 没有	O 少许	O 有些	O 蛮多	O 很多

**24a**. 在过去的 7 天里，**失眠（包括难于入睡、难于保持睡眠状态或早醒）**在**最严重**时的程度：				
O 没有	O 轻微	O 中度	O 严重	O 很严重
**24b**. 在过去的 7 天里，**失眠（包括难于入睡、难于保持睡眠状态或早醒）影**响你日常活的程度：				
O 没有	O 少许	O 有些	O 蛮多	O 很多

**25a**. 在过去的 7 天里，**疲乏(容易累)、疲倦或精力不足**在**最严重**时的程度：				
O 没有	O 轻微	O 中度	O 严重	O 很严重
**25b**. 在过去的 7 天里，**疲乏(容易累)、疲倦或精力不足影响**你日常活动的程度：				
O 没有	O 少许	O 有些	O 蛮多	O 很多

**26a**. 在过去的 7 天里，你是否**常常**感到**焦虑：**				
O 从来没有	O 很少	O 偶尔	O 经常	O 几乎是持续的
**26b**. 在过去的 7 天里，**焦虑**在**最严重**时的程度：				
O 没有	O 轻微	O 中度	O 严重	O 很严重
**26c**. 在过去的 7 天里，**焦虑影响**你日常活动的程度：				
O 没有	O 少许	O 有些	O 蛮多	O 很多

**27a**. 在过去的 7 天里，你是否**常常**感到**没有任何事情可以让你高兴/振作起来：**				
O 从来没有	O 很少	O 偶尔	O 经常	O 几乎是持续的
**27b**. 在过去的 7 天里，**没有任何事情可以让你高兴/振作起来的感觉**在**最严重**时的程度：				
O 没有	O 轻微	O 中度	O 严重	O 很严重
**27c**. 在过去的 7 天里，**没有任何事情/东西可以让你高兴/振作起来的感觉影响**你日常活动的程度：				
O 没有	O 少许	O 有些	O 蛮多	O 很多

**28a**. 在过去的 7 天里，**小便失去控制（尿液漏出）**是**否常常**出现：				
O 从来没有	O 很少	O 偶尔	O 经常	O 几乎是持续的
**28b**. 在过去的 7 天里，**小便失去控制（尿液漏出）影响**你日常活动的程度：				
O 没有	O 少许	O 有些	O 蛮多	O 很多

**29a**. 在过去的 7 天里，**性兴趣下降**在**最严重**时的程度：						
O 没有	O 轻微	O 中度	O 严重	O 很严重	O 没有性活跃	O 不想回答

**30a**. 在过去的 7 天里，你是否**出汗异常减少：**	
O 是	O 否

**31a**. 在过去的 7 天里，**潮热 (感觉热，出汗)** 是否**常常**出现：				
O 从来没有	O 很少	O 偶尔	O 经常	O 几乎是持续的
**31b**. 在过去的 7 天里，**潮热 (感觉热，出汗)** 在**最严重**时的程度：				
O 没有	O 轻微	O 中度	O 严重	O 很严重

**其他症状**	
除了以上症状，你是否有其他症状希望告诉我们？	
O 是	O 否
**请列出其他症状：**	
1.	在过去的 7 天里，这症状在**最严重**时的程度：<br><br>O 没有　O 轻微　O 中度　O 严重　O 很严重
2.	在过去的 7 天里，这症状在**最严重**时的程度：<br><br>O 没有　O 轻微　O 中度　O 严重　O 很严重
3.	在过去的 7 天里，这症状在**最严重**时的程度：<br><br>O 没有　O 轻微　O 中度　O 严重　O 很严重
4.	在过去的 7 天里，这症状在**最严重**时的程度：<br><br>O 没有　O 轻微　O 中度　O 严重　O 很严重
5.	在过去的 7 天里，这症状在**最严重**时的程度：<br><br>O 没有　O 轻微　O 中度　O 严重　O 很严重
