# Supplementary material for: Development and validation of a customised PRO-CTCAE scale for adult-type diffuse gliomas (VERONICA): a multicentre, prospective, observational cohort study in China
Source: eClinicalMedicine. 2026 Apr 10;94:103879. doi: 10.1016/j.eclinm.2026.103879 (PMC13091834; doi:10.1016/j.eclinm.2026.103879)
Supplement: Translated Abstract [file mmc2.pdf]

The following translations in Chinese were submitted by the authors and we reproduce them as supplied. They have not been peer reviewed. Our editorial processes have only been applied to the original abstract in English, which should serve as reference for this manuscript.

Translated Abstract (Simplified Chinese Version)

背景

患者报告结局（patient-reported outcomes, PROs）对于从患者视角评估症状性不良事件（adverse events, AEs）至关重要，而此类不良事件会显著影响胶质瘤患者的生活质量和临床结局。然而，目前尚无经过验证的患者报告结局测量工具（patient-reported outcome measures, PROMs）可用于量化成人型弥漫性胶质瘤中的症状性不良事件。

方法

本研究分为两部分。第一部分，我们基于简体中文版 PRO-CTCAE 条目库，结合初筛、患者预实验和两轮 Delphi 专家咨询，开发了适用于成人型弥漫性胶质瘤的定制化患者报告版不良事件通用术语标准（Patient-Reported Outcomes version of the Common Terminology Criteria for Adverse Events, PRO-CTCAE）量表。Delphi 专家通过国家胶质瘤多学科诊疗联盟（National Glioma Multidisciplinary Team Alliance, NGMA）招募，并分别于 2022 年 6 月（第 1 轮）和 2022 年 8 月（第 2 轮）通过电子邮件邀请参加。第二部分，我们于 2022 年 9 月至 2025 年 3 月在中国 13 家胶质瘤诊疗中心开展了多中心、前瞻性、观察性队列研究（VERONICA）。符合条件的受试者为年龄 18 岁及以上、经诊断为成人型弥漫性胶质瘤且能够理解并完成问卷的患者；严重认知障碍、严重语言功能障碍或其他无法完成问卷评估者予以排除。本研究的主要结局为该定制化 PRO-CTCAE 量表的心理测量学表现，包括重测信度、聚合效度、已知组效度和反应度，并通过研究过程中多次访视的纵向评估进行检验。VERONICA 已在 ClinicalTrials.gov 注册，注册号为 NCT05486923。

结果

在 Delphi 专家咨询部分，第 1 轮中，来自 6 家中心的 7 位受邀专家全部参与（应答率 100.0%），症状排序一致性为中等（Kendall’s W=0.415；p<0.001）。第 2 轮中，来自 14 家中心的 20 位受邀专家中有 16 位参与（应答率 80.0%），专家评分一致性良好（Kendall’s W=0.351；p<0.001）。最终定制化 PRO-CTCAE 量表共包含 53 个条目，覆盖 31 个症状，另设 1 个开放式自由文本条目。

在 VERONICA 研究中，共有 450 例受试者来自 13 家胶质瘤诊疗中心纳入研究。平均年龄为 49.1 岁（标准差 12.8），基线时（第 2 次访视）Karnofsky 体能状态评分（Karnofsky Performance Status, KPS）平均为 72.2（标准差 17.1）。其中，424 例至少提供了 1 项预设心理测量学分析所需的数据。重测信度良好，53 个条目中有 47 个条目的组内相关系数（intraclass correlation coefficient, ICC）≥0.70。聚合效度得到支持：该量表与欧洲癌症研究与治疗组织生活质量核心问卷（European Organisation for Research and Treatment of Cancer Quality of Life Questionnaire-Core 30, EORTC QLQ-C30）对应领域之间呈预期方向相关，且多数相关性达到中等至较强水平（25 个条目 r≥0.50）。已知组效度得到支持：在 KPS<70 与 KPS≥70 的患者之间，该量表能够有效区分，53 个条目中有 49 个条目的 Cohen’s d≥0.20，其中 49 个条目中有 43 个达到统计学显著性（p<0.05）。在以总体变化印象（Global Impression of Change, GIC）为锚定的反应度分析中，自报告总体状态恶化的受试者中有 37 个条目的标准化反应均值（standardised response means, SRMs）≥0.20。

解释

该定制化 PRO-CTCAE 量表在成人型弥漫性胶质瘤中显示出稳健的心理测量学表现。其远程、纵向施测方式有助于在临床试验和常规神经肿瘤临床实践中以较低负担量化患者报告的症状性不良事件。未来研究仍需在常规医疗和临床试验中进一步评估其实施应用，并在不同语言中继续开展翻译、文化适配和验证。

资助来源

北京医学奖励基金会；上海市卫生健康委员会；宁夏回族自治区科学技术厅；复旦大学附属华山医院（临床研究项目）。

关键词

患者报告结局；PRO-CTCAE；成人型弥漫性胶质瘤；症状性不良事件；心理测量学验证；观察性队列研究
