# Supplementary material for: Development and validation of a customised PRO-CTCAE scale for adult-type diffuse gliomas (VERONICA): a multicentre, prospective, observational cohort study in China
Source: eClinicalMedicine. 2026 Apr 10;94:103879. doi: 10.1016/j.eclinm.2026.103879 (PMC13091834; doi:10.1016/j.eclinm.2026.103879)
Supplement: Veronica Protocol [file mmc3.pdf]

Supplementary Study Protocol:

Clinical Trial Protocol

Development and psychometric validation of a customised Patient-Reported Outcomes version of the Common Terminology Criteria for Adverse Events (PRO-CTCAE) scale in Chinese adults with adult-type diffuse glioma (VERONICA)

Version 4.6
2022-May

CONTENTS

i. Investigator Statement 4

ii. Abbreviations and Definitions of Terms Table 5

iii. Protocol Synopsis 6

iv. Text 8

1 Study background 8

2 Study objectives 9

3 Study subjects 9

3.1 Criteria for enrolment 9

3.2 Exclusion criteria 10

3.3 Criteria for discontinuation 10

4 Study Design and Study Personnel 10

4.1 Study design 10

4.2 Study personnel 20

5 Study plan and data collection 21

5.1 Basic information and clinical information 21

5.2 Study procedures 25

5.4 Primary endpoint evaluation 29

5.5 Secondary and exploratory endpoint evaluations 33

5.6 Serious adverse events 33

6 Study conducts and management 34

6.1 Study procedures 34

6.2 Study schedule and expected completion time 35

6.3 Potential benefits to participants 35

7 Statistical analysis 36

v. Investigator Responsibilities 38

vi. Ethical, legal, and administrative provisions 40

1. Ethics Committee 40

2 Informed consent 40

3 Protocol amendment 41

4 Confidentiality agreement and study subject privacy 41

VPRONICA 倾听患者之声

5 Data archiving and data management 41
vii. References 43

i. Investigator Statement

I declare:

Be responsible for the correct implementation of the clinical study in this site;

Correctly carry out the study according to the requirements of the study protocol, protocol amendment and the Ethics Committee for clinical trial;

The clinical study must be conducted in compliance with GCP and regulatory requirements;

Ensure that all staff at this site participating in this study are fully familiar with study procedures, study methods, study scales and their individual responsibilities in the study;

The study protocol shall not be modified and implemented without the review and approval of the Ethics Committee, unless emergency measures are taken to reduce the harm to subjects or for the purpose of study management (with the permission of relevant regulatory authorities);

____

Investigator Signature/Date

____

Investigator Name (print)

____

Study site

____

Contact number

VIPRONICA 倾听患者之声

ii. Abbreviations and Definitions of Terms Table

Abbreviation	Chinese term
CNS5	Fifth World Health Organisation Classification of Central Nervous System Tumours
PRO	Patient Reported Outcomes
COA	Clinical Outcome Assessments
NCI	National Institute
QLQ-C30	European Organisation for Research and Treatment of Cancer Quality of Life Questionnaire
CTCAE	Common Terminology Criteria for Adverse Events
KPS	Karnofsky score
GIC	Global impression of change
GCP	Good Clinical Practice
ICF	Informed Consent Form
MRI	Magnetic resonance imaging
RANO	Criteria for evaluation of neuro-neoplastic response
RT	Radiotherapy
AE	Adverse Events
SAE	Serious Adverse Events
HRQOL	Health-related Quality of Life Questionnaire
OS	Overall survival
PFS	Progression-free survival

VPRONICA
倾听患者之声

iii. Protocol Synopsis

Study name	Validity, Reliability, and Responsiveness Assessment Based on Common Terminology for Adverse Events Patient Self Report Version (PRO-CTCAE ™) in Chinese Patients with Adult Type Diffuse Glioma
Type of study	Prospective, observational study
Study objectives	To evaluate the validity, reliability and responsiveness of customized PRO-CTCAE in Chinese patients with adult-type diffuse glioma
Study Design	1. This study is an observational, prospective, open-label, multicenter clinical study<br>2. This study is a prospective, multicenter clinical trial and is expected to enroll 450 patients with high-grade glioma from approximately 17 centers.<br>3. In this study, 450 patients underwent 5 visits (6 visits for some patients) with customized PRO-CTCAE scale, QLQ-C30 scale and GIC scalar.<br>4. The data were statistically analyzed to evaluate the reliability, validity, and responsiveness of the customized PRO-CTCAE scale.
Selection of Subjects (Inclusion/Exclusion Criteria)	**Criteria for inclusion:**<br>1) Patients with pathologically confirmed adult-type diffuse glioma (including astrocytoma, IDH mutant; oligodendroglioma, IDH mutant with combined 1p/19q deletion; glioblastoma, IDH wild-type; and other adult-type diffuse gliomas NEC/NOS.)<br>2) For newly diagnosed patients, the patient has not yet undergone the first non-surgical treatment<br>3) For patients who have relapsed, the patient has not yet undergone the first nonoperative treatment following this relapse<br>4) 18 to 85 years<br>5) No significant cognitive impairment based on investigator judgment<br>6) Able to use mobile phones or computers on their own or with the help of others, able to read and understand Chinese, at least with primary school culture<br>7) Ongoing antineoplastic therapy and continuing for the next 28 days<br>8) Patient signed written informed consent<br><br>**Exclusion Criteria:**<br>1) Patients considered unsuitable for this study by the investigator<br>2) Patient has been treated non-operatively since this disease diagnosis<br>3) Questionnaire not completed within 28 days of informed consent<br><br>**Criteria for discontinuation:**<br>1) For investigators: This study is a prospective study. If significant errors are found in the clinical study protocol, or serious deviations occur during the implementation of the protocol; government departments or ethics committees require suspension or termination of the study; the study is invalid; the investigator considers it inappropriate to continue the study or considers it difficult to continue the study.<br>2) For a subject: the subject requested withdrawal; the investigator judged continuation of the trial to be detrimental to the subject;

	the subject died.
Planned Cases	450 cases
Treatment Regimen	Including standard treatment regimens for adult-type diffuse glioma as well as those considered appropriate by other studies
Outcome Measures	Validity, reliability, and responsiveness of a customized version of the patient-reported outcome version of the Common Terminology Criteria for Adverse Events (PRO-CTCAE ™) in Chinese patients with adult-type diffuse glioma.
Duration of Study	Protocol Finalized: 2022-06<br>First subject enrolled:<br>Last Subject Out:<br>Database lock:<br>Statistical Analysis:<br>Study Summary Report:

VERONICA 倾听患者之声

iv. Text

1 Study background

Glioma is the most common primary malignant intracranial tumour in adults. In China, the annual incidence is approximately 5-8 per 100,000 population. Adult-type diffuse glioma, as defined in the 2021 fifth edition of the World Health Organisation Classification of Central Nervous System Tumours (WHO CNS5), includes IDH-mutant astrocytoma, IDH-mutant and 1p/19q-codeleted oligodendroglioma, and IDH-wildtype glioblastoma. Current management remains centred on surgery, radiotherapy, systemic therapy, tumour treating fields (TTFields), and selected targeted therapies.

In glioma trials, efficacy assessment has traditionally relied on tumour-based and survival-based endpoints, such as Response Assessment in Neuro-Oncology (RANO) criteria, progression-free survival, and overall survival. Although these endpoints remain essential, they do not fully capture the effect of disease and treatment on patients' symptoms, functioning, and day-to-day wellbeing.

Clinical outcome assessments (COAs) provide structured measures of a patient's health status from the patient, clinician, observer, or performance perspective. COAs include patient-reported outcomes (PROs), clinician-reported outcomes such as Karnofsky Performance Status (KPS), observer-reported outcomes, and performance outcomes. Appropriate COAs can directly capture the patient experience and may therefore improve the evaluation of treatment benefit and treatment burden.

In December 2021, the Centre for Drug Evaluation in China issued guidance on the use of patient-reported outcomes in drug clinical studies, reflecting the growing regulatory and clinical importance of PRO data. Accumulating evidence from oncology trials suggests that systematic symptom monitoring using PROs may improve patient outcomes, reduce unplanned healthcare use, and support more patient-centred care.

These considerations are particularly relevant in neuro-oncology. Many patients with glioma experience a substantial symptom burden at diagnosis and during treatment, including impairments in cognition, communication, physical functioning, emotional wellbeing, and seizure control. International initiatives such as the Response Assessment in Neuro-Oncology Patient-Reported Outcomes (RANO-PRO) working group have emphasised the need for robust patient-reported assessment of key symptoms and functions in glioma research and practice.

The Common Terminology Criteria for Adverse Events (CTCAE), issued by the US National Cancer Institute (NCI), is the standard framework for clinician-reported adverse event reporting in oncology. To complement clinician reporting for symptomatic adverse events, the NCI developed the Patient-Reported Outcomes version of the Common Terminology Criteria for Adverse Events (PRO-CTCAE), a modular system that allows direct patient reporting of the frequency, severity, and interference of treatment-related symptoms. The official Simplified Chinese version became available in 2019, but a glioma-specific customised version had not been formally developed or psychometrically

validated in Chinese patients with adult-type diffuse glioma.

Any new clinical measurement tool must be shown to be accurate, reliable, and clinically informative before routine use. For PRO instruments, validity, reliability, and responsiveness are key psychometric properties used to evaluate whether the instrument measures the intended constructs, produces stable results when health status is unchanged, and is sensitive to meaningful clinical change over time.

The present study therefore aimed to develop a customised PRO-CTCAE scale for adults with adult-type diffuse glioma and to evaluate its psychometric performance in a prospective, multicentre, non-interventional cohort study in China.

2 Study objectives

To develop a customised Patient-Reported Outcomes version of the Common Terminology Criteria for Adverse Events (PRO-CTCAE) scale for adult-type diffuse glioma and to evaluate its reliability, validity, and responsiveness in a two-part study comprising scale development through patient pilot testing and Delphi consensus and psychometric validation in the multicentre prospective VERONICA cohort.

3 Study subjects

To ensure geographic and clinical diversity, the psychometric validation stage was conducted across 13 glioma treatment centres in China. The scale-development stage comprised expert consultation through the National Glioma Multidisciplinary Team (MDT) Alliance and patient pilot testing at the lead centre, whereas the VERONICA cohort enrolled adults with adult-type diffuse glioma who met prespecified eligibility criteria.

3.1 Criteria for enrolment

1) Adults aged 18 to 85 years.
2) Integrated diagnosis of adult-type diffuse glioma according to the 2021 WHO CNS5 classification, including IDH-mutant astrocytoma, IDH-mutant and 1p/19q-codeleted oligodendroglioma, and IDH-wildtype glioblastoma.
3) For newly diagnosed patients, no non-surgical anti-tumour treatment had been started at enrolment.
4) For recurrent patients, recurrence had been confirmed according to RANO criteria within 42 days before enrolment, and no non-surgical treatment for that recurrence had yet been initiated.
5) No severe cognitive impairment or language dysfunction that would preclude questionnaire completion, based on investigator judgement.
6) Able to read and understand Chinese and to complete the questionnaire independently or with assistance.

7) Planned to initiate or continue anti-tumour therapy within 28 days.
8) Written informed consent obtained before study procedures.

3.2 Exclusion criteria

1) Patients considered unsuitable for participation by the investigator.
2) Receipt of non-surgical anti-tumour treatment before baseline questionnaire completion for the relevant disease stage (new diagnosis or recurrence).
3) Failure to complete the baseline questionnaire within 42 days after informed consent.

3.3 Criteria for discontinuation

1) For investigators: this was a prospective study. Study suspension or termination could occur if major protocol errors were identified, serious protocol deviations occurred during implementation, regulatory authorities or ethics committees required suspension or termination, the study was judged to be scientifically invalid, or the investigator considered continued conduct inappropriate or infeasible.
2) For participants: study participation could be discontinued at the participant's request, if the investigator judged continued participation to be detrimental to the participant, or in the event of death.

4 Study Design and Study Personnel

4.1 Study design

This study was conducted in two parts. Part 1 comprised development of a customised PRO-CTCAE scale for adult-type diffuse glioma through initial screening of the official Simplified Chinese PRO-CTCAE item library, patient pilot testing, and a two-round Delphi survey. Part 2 was a multicentre, prospective, observational cohort study (VERONICA; ClinicalTrials.gov NCT05486923) conducted at 13 glioma treatment centres in China between September 2022 and March 2025 to evaluate the reliability, validity, and responsiveness of the customised scale.

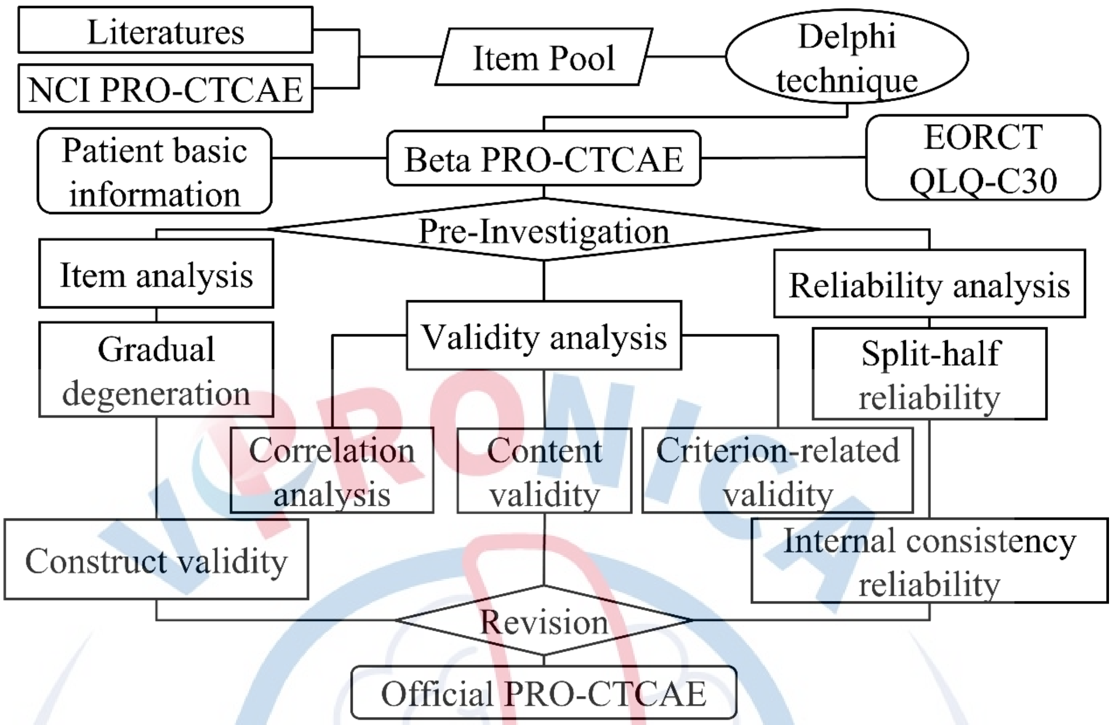

4.1.1 Expert survey and patient pilot testing (content validity)

Part 1 evaluated the content relevance and clinical importance of PRO-CTCAE items for adults with diffuse glioma through patient pilot testing and expert Delphi consensus, informed by literature review and review of treatment pathways relevant to neurosurgery and neuro-oncology.

All candidate items were selected from the official Simplified Chinese version of the NCI PRO-CTCAE item library and were administered without modification to item wording, response options, or scoring. Initial screening removed items considered less relevant to adult-type diffuse glioma and to neurosurgical or neuro-oncology care pathways, thereby generating a beta customised scale.

倾听患者之声

PRO-CTCAE COMPLETE ENTRY TABLE

Oral	
Dry mouth	S
Difficulty swallowing	S
Mouth/throat sores	SI
Cracking at the corners of the mouth (cheilosis/ cheilitis)	S
Voice quality changes	P
Hoarseness	S

Gastrointestinal	
Taste changes	S
Decreased appetite	SI
Nausea	FS
Vomiting	FS
Heartburn	FS
Gas	P
Bloating	FS
Hiccups	FS
Constipation	S
Diarrhea	F
Abdominal pain	FSI
Fecal incontinence	FI

Respiratory	
Shortness of breath	SI
Cough	SI
Wheezing	S

Cardio/Circulatory	
Swelling	FSI
Heart palpitations	FS

Cutaneous	
Rash	P
Skin dryness	S
Acne	S
Hair loss	A
Itching	S
Hives	P
Hand- foot syndrome	S
Nail loss	P
Nail ridging	P
Nail discoloration	P
Sensitivity to sunlight	P
Bed/pressure sores	P
Radiation skin reaction	S
Skin darkening	P
Stretch marks	P

Neurological	
Numbness & tingling	SI
Dizziness	SI

Visual/Perceptual	
Blurred vision	SI
Flashing lights	P
Visual floaters	P
Watery eyes	SI
Ringing in ears	S

Attention/Memory	
Concentration	SI
Memory	SI

Pain	
General pain	FSI
Headache	FSI
Muscle pain	FSI
Joint pain	FSI

Sleep/Wake	
Insomnia	SI
Fatigue	SI

Mood	
Anxious	FSI
Discouraged	FSI
Sad	FSI

Genitourinary	
Irregular periods/vaginal bleeding	P
Missed expected menstrual period	P
Vaginal discharge	A
Vaginal dryness	S
Painful urination	S
Urinary urgency	FI
Urinary frequency	FI
Change in usual urine color	P
Urinary incontinence	FI

Sexual	
Achieve and maintain erection	S
Ejaculation	F
Decreased libido	S
Delayed orgasm	P
Unable to have orgasm	P
Pain w/sexual intercourse	S

Miscellaneous	
Breast swelling and tenderness	S
Bruising	P
Chills	FS
Increased sweating	FS
Decreased sweating	P
Hot flashes	FS
Nosebleed	FS
Pain and swelling at injection site	P
Body odor	S

Attributes	
F: Frequency	I: Interference
S: Severity	P: Presence/Absence
A: Amount	

NIH National Cancer Institute

*Complete library of items available at: https://healthcaredelivery.cancer.gov/pro-ctcae

Version date: 3/11/2020

*Note: Each letter corresponds to a questionnaire entry (F , **frequency; S, degree; A, number; I, interference with life; P, present**)*

The first Delphi round invited seven experts from six centres in the field of glioma care in China, and all seven participated (response rate 100%). Experts used a five-point Likert scale to rate clinical importance and could provide free-text comments and suggestions. The overall degree of agreement was assessed using Kendall's W; in parallel, 30 patients completed the beta version in pilot testing at the lead centre.

Pilot testing assessed feasibility and informed expert consensus. For correlation-based screening, PRO-CTCAE responses were analytically recoded into standardised numeric scores to enable symptom-total association analyses across different item structures, without modification of original wording, response options, or scoring format.

Baseline Characteristics of Participants in the Patient Pilot Testing.

Variables	Total (n = 30)
**Age, Mean ± SD**	48·0±16·0
**Sex, n(%)**	
Female	14 (53·3)
Male	16 (46·7)
**WHO CNS5 Grade, n(%)**	
3	6 (20·0)
4	24 (80·0)
**KPS, n(%)**	
≥80	25 (83·3)
50-70	4 (13·3)
≤40	1 (3·3)
**IDH mutation status, n(%)**	
MUT	9 (30·0)
WT	21 (70·0)

VIPRONICA 倾听患者之声

The results of the first Delphi analysis were as follows:

PRO-CTCAE System	PRO-CTCAE Symptom	Category [a]	Mean Importance Rating [b]	Spearman Rank Correlations (r_s) [c]	Patient-Reported Positivity Rate [d] (n=30)	Conclusion
Visual/Perceptual	Blurred vision	T2	5·00	0·38	0·40	Confirmed
Gastrointestinal	Vomiting	T2	4·83	0·41	0·37	Confirmed
Neurological	Dizziness	T2	4·50	0·58	0·37	Confirmed
Pain	General pain	T3	4·50	0·52	0·47	Confirmed
Sleep/Wake	Fatigue	T2	4·33	0·74	0·67	Confirmed
Mood	Sad [e]	T3	4·17	0·56	0·50	Pending
Attention/Memory	Memory	T2	4·17	0·50	0·53	Confirmed
Gastrointestinal	Decreased appetite	T2	4·00	0·66	0·37	Confirmed
Mood	Anxious	T3	4·00	0·61	0·47	Confirmed
Gastrointestinal	Nausea	T2	4·00	0·49	0·33	Confirmed
Respiratory	Shortness of breath	T2	4·00	0·44	0·13	Confirmed
Visual/Perceptual	Flashing lights	SD	4·00	0·42	0·10	Confirmed
Attention/Memory	Concentration	T2	3·83	0·61	0·60	Confirmed
Visual/Perceptual	Ringing in ears	S5	3·83	0·40	0·23	Confirmed
Mood	Discouraged [e]	T3	3·83	0·39	0·53	Pending
Cardio/Circulatory	Heart palpitations	T2	3·50	0·59	0·13	Confirmed
Miscellaneous	Hot flashes	T2	3·50	0·39	0·47	Confirmed
Cutaneous	Itching	S5	3·33	0·59	0·47	Confirmed
Cutaneous	Hives	SD	3·33	0·48	0·27	Confirmed
Miscellaneous	Decreased sweating	SD	3·33	0·38	0·27	Confirmed
Gastrointestinal	Taste changes	S5	3·17	0·41	0·17	Confirmed
Pain	Headache [f]	T3	5·00	0·26	0·37	Confirmed
Neurological	Numbness & tingling	T2	4·83	0·23	0·17	Pending
Oral	Difficulty swallowing	S5	4·50	0·05	0·13	Pending
Sleep/Wake	Insomnia	T2	4·17	0·30	0·57	Pending
Cutaneous	Radiation skin reaction	S5	4·17	0·29	0·13	Pending
Genitourinary	Urinary incontinence	T2	4·17	0·25	0·13	Pending
Cutaneous	Bed/pressure sores	SD	4·17	0·25	0·03	Pending

VERONICA
倾听患者之声

PRO-CTCAE System	PRO-CTCAE Symptom	Category [a]	Mean Importance Rating [b]	Spearman Rank Correlations (r_s) [c]	Patient-Reported Positivity Rate [d] (n=30)	Conclusion
Gastrointestinal	Hiccups	T2	4·00	0·18	0·30	Pending
Cutaneous	Hair loss	S5	3·67	0·30	0·47	Pending
Visual/Perceptual	Visual floaters	SD	3·67	0·23	0·13	Pending
Miscellaneous	Bruising	SD	3·67	0·13	0·17	Pending
Cutaneous	Skin darkening	SD	3·50	0·19	0·20	Pending
Cutaneous	Rash [f]	SD	3·50	0·16	0·30	Confirmed
Miscellaneous	Increased sweating	T2	3·33	0·29	0·40	Pending
Respiratory	Cough	T2	3·33	0·24	0·10	Pending
Gastrointestinal	Constipation	S5	3·17	0·11	0·17	Pending
Cardio/Circulatory	Swelling	T3	3·00	0·28	0·17	Pending
Oral	Hoarseness	S5	3·00	0·04	0·33	Pending
Oral	Dry mouth	S5	2·83	0·29	0·70	Pending
Gastrointestinal	Diarrhea	S5	2·83	0·17	0·27	Pending
Oral	Mouth/throat sores	T2	2·83	0·16	0·13	Pending
Cutaneous	Stretch marks	SD	2·67	0·25	0·03	Pending
Oral	Voice quality changes	SD	2·67	0·13	0·17	Pending

[a] **Category**: Symptoms were grouped by PRO-CTCAE item structure for pilot-testing analyses: S5, single item with five response options; T2, two-item symptoms; T3, three-item symptoms; SD, single dichotomous (presence/absence) item.

[b] **Mean Importance Rating**: Mean rating from 1st round Delphi survey using a five-point Likert scale (higher scores indicate greater clinical importance).

[c] **Spearman Rank Correlations (r_s)**: Symptom–total associations were estimated in the pilot testing using Spearman rank correlation between each symptom score and the total score (sum of all symptom scores). For this analysis only, PRO-CTCAE response options were prespecified to be analytically recoded into standardised numeric scores to allow comparability across symptom structures, with the maximum possible symptom score standardised to 6. Recoding schemes were: S5 (0, 1·5, 3, 4·5, 6); T2 (each item: 0, 0·75, 1·5, 2·25, 3; summed across two items); T3 (each item: 0, 0·5, 1, 1·5, 2; summed across three items); SD (No=0, Yes=6). This analytical recoding was used solely for correlation/ranking purposes and did not modify the original PRO-CTCAE item wording, response options, scoring format, or administration.

[d] **Patient-Reported Positivity Rate**: Proportion of pilot-testing participants reporting at least mild symptom burden (i.e., any response above “none/not at all”, where applicable).

[e] In the official Simplified Chinese version of the PRO-CTCAE® item library, “discouraged” and “sad” are translated as “没有任何事情可以让你高兴/振作起来的感觉” and “悲伤或不开心的感觉”, respectively. Based on patient pilot testing and expert feedback, these items were difficult to clearly differentiate in the Chinese context and were considered conceptually overlapping; both also map to the same CTCAE v5·0 adverse event domain (depression). Therefore, although both ranked highly in item prioritisation, only one was retained as a confirmed symptom in the final instrument to reduce redundancy and respondent burden. So, they were classified as pending symptoms in this round.

Score 2: High expert score and low proportion of patients with high score

Score 3: Very high expert score and low proportion of patients with high score

4 points: High expert score, high proportion of patients with high score

5 points: High expert score, good test result

VERONICA
倾听患者之声

After the first Delphi round and pilot testing, the pending items, pilot-testing metrics, and the full PRO-CTCAE item library were fed back to experts for comments and refinement before the second Delphi round.

2nd round investigation version of P RO-CTCAE for glioma patients

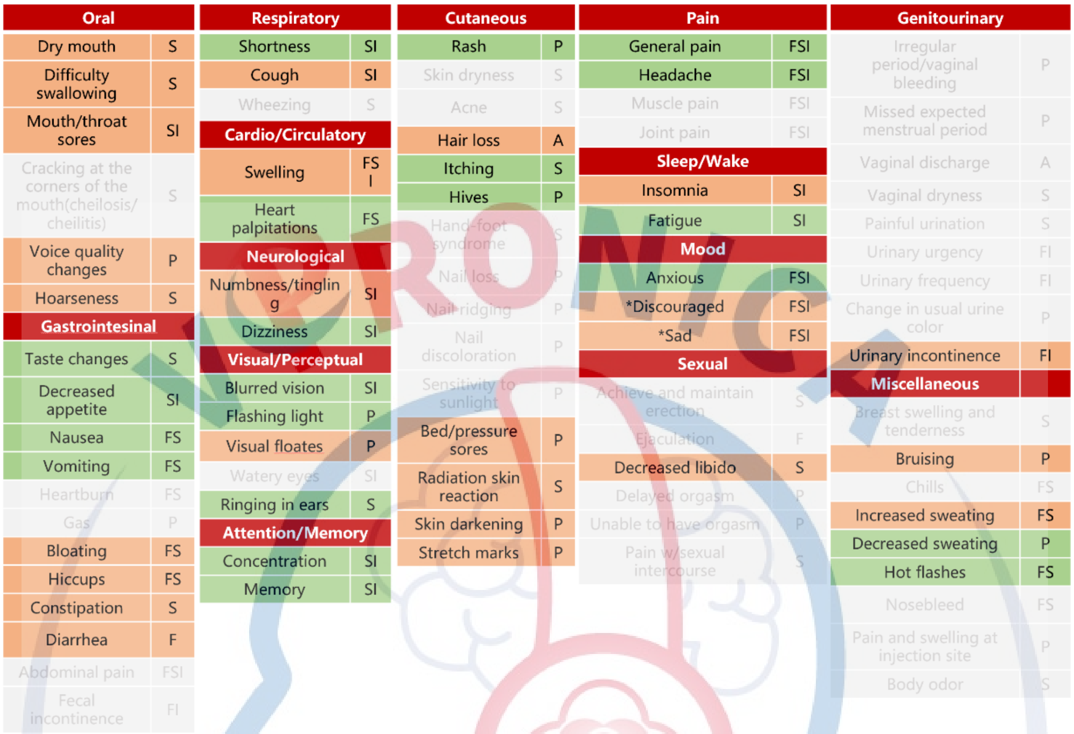

Content validity was evaluated using the mean importance score and the item-level content validity index (I-CVI), defined as the proportion of experts rating an item 3 to 5 on the Likert scale.

Items were prioritised using the prespecified hierarchy of mean importance score, I-CVI, and CV. The final customised glioma-specific PRO-CTCAE scale was assembled by combining confirmed symptoms from both rounds.

The results of the second Delphi analysis were as follows:

No.	*PRO-CTCAE Symptom*	*Mean Importance Rating*	*CVI*	*CV*	*Conclusion*
1	Insomnia	4·69	1	0·1	Confirmed
2	Discouraged a	4·44	1	0·16	Confirmed
3	Urinary incontinence	4·25	1	0·14	Confirmed
4	Hair loss	4·25	0·88	0·26	Confirmed
5	Sad a	4·06	0·94	0·26	Excluded
6	Difficulty swallowing	4·06	0·88	0·32	Confirmed
7	Bed/Pressure sores	4·06	0·88	0·26	Confirmed
8	Decreased libido	4	1	0·20	Confirmed

倾听患者之声

9	Radiation skin reaction	3·94	0·88	0·30	Confirmed
10	Numbness/tingling	3·94	0·88	0·29	Confirmed
11	Constipation	3·63	0·88	0·30	Confirmed
12	Swelling Bloating	3·56	0·81	0·38	Excluded
13	Bruising	3·50	0·94	0·26	Excluded
14	Swelling	3·44	0·81	0·32	Excluded
15	Mouth/throat sores	3·31	0·69	0·38	Excluded
16	Increased sweating	3·25	0·81	0·33	Excluded
17	Hiccup	3·19	0·81	0·29	Excluded
18	Cough	3·19	0·75	0·37	Excluded
19	Skin darkening	3·19	0·69	0·35	Excluded
20	Visual floats	3·13	0·75	0·35	Excluded
21	Diarrhea	3	0·75	0·37	Excluded
22	Dry mouth	3	0·69	0·34	Excluded
23	Hoarseness	2·94	0·63	0·4	Excluded
24	Stretch marks	2·63	0·56	0·36	Excluded
25	Voice quality changes	2·38	0·44	0·34	Excluded

After two Delphi rounds and integration of patient pilot-testing information, the final customised PRO-CTCAE scale comprised 53 items covering 31 symptoms, together with one open-ended free-text item (see retained operational item table/appendices).

Customized PRO-CTCAE Entry Table for Adult-type diffuse glioma Patients

Category	Item	Entry
Oral	Dry mouth	S
Oral	Difficulty swallowing	S
Oral	Mouth/throat sores	SI
Oral	Cracking at the corners of the mouth(cheilosis/cheilitis)	S
Oral	Voice quality changes	P
Oral	Hoarseness	S
Gastrointesinal	Taste changes	S
Gastrointesinal	Decreased appetite	SI
Gastrointesinal	Nausea	FS
Gastrointesinal	Vomiting	FS
Gastrointesinal	Heartburn	FS
Gastrointesinal	Gas	P
Gastrointesinal	Bloating	FS
Gastrointesinal	Hiccups	FS
Gastrointesinal	Constipation	S
Gastrointesinal	Diarrhea	F
Gastrointesinal	Abdominal pain	FSI
Gastrointesinal	Fecal incontinence	FI
Respiratory	Shortness	SI
Respiratory	Cough	SI
Respiratory	Wheezing	S
Cardio/Circulatory	Swelling	FSI
Cardio/Circulatory	Heart palpitations	FS
Neurological	Numbness/tingling	SI
Neurological	Dizziness	SI
Visual/Perceptual	Blurred vision	SI
Visual/Perceptual	Flashing light	P
Visual/Perceptual	Visual floates	P
Visual/Perceptual	Watery eyes	SI
Visual/Perceptual	Ringing in ears	S
Attention/Memory	Concentration	SI
Attention/Memory	Memory	SI
Cutaneous	Rash	P
Cutaneous	Skin dryness	S
Cutaneous	Acne	S
Cutaneous	Hair loss	A
Cutaneous	Itching	S
Cutaneous	Hives	P
Cutaneous	Hand-foot syndrome	S
Cutaneous	Nail loss	P
Cutaneous	Nail ridging	P
Cutaneous	Nail discoloration	P
Cutaneous	Sensitivity to sunlight	P
Cutaneous	Bed/pressure sores	P
Cutaneous	Radiation skin reaction	S
Cutaneous	Skin darkening	P
Cutaneous	Stretch marks	P
Pain	General pain	FSI
Pain	Headache	FSI
Pain	Muscle pain	FSI
Pain	Joint pain	FSI
Sleep/Wake	Insomnia	SI
Sleep/Wake	Fatigue	SI
Mood	Anxious	FSI
Mood	*Discouraged	FSI
Mood	*Sad	FSI
Sexual	Achieve and maintain erection	S
Sexual	Ejaculation	F
Sexual	Decreased libido	S
Sexual	Delayed orgasm	P
Sexual	Unable to have orgasm	P
Sexual	Pain w/sexual intercourse	S
Genitourinary	Irregular period/vaginal bleeding	P
Genitourinary	Missed expected menstrual period	P
Genitourinary	Vaginal discharge	A
Genitourinary	Vaginal dryness	S
Genitourinary	Painful urination	S
Genitourinary	Urinary urgency	FI
Genitourinary	Urinary frequency	FI
Genitourinary	Change in usual urine color	P
Genitourinary	Urinary incontinence	FI
Miscellaneous	Breast swelling and tenderness	S
Miscellaneous	Bruising	P
Miscellaneous	Chills	FS
Miscellaneous	Increased sweating	FS
Miscellaneous	Decreased sweating	P
Miscellaneous	Hot flashes	FS
Miscellaneous	Nosebleed	FS
Miscellaneous	Pain and swelling at injection site	P
Miscellaneous	Body odor	S

Items confirm to retain

Items confirm to remove

*Note: Each letter corresponds to a questionnaire entry (**F , frequency; S, degree; A, number; I, interference with life; P, presence**)*

4.1.2 Formal study design

Part 2 (VERONICA) evaluated the psychometric performance of the customised PRO-CTCAE scale in Chinese adults with adult-type diffuse glioma. The study was a multicentre, prospective, observational cohort study conducted at 13 centres, with a target enrolment of 450 participants.

This was a non-interventional study. Anti-tumour treatment was not assigned by protocol; instead, treatment regimens were recorded observationally and questionnaire assessments were undertaken according to the study schedule.

Karnofsky Performance Status (KPS) was prespecified as an anchor for known-groups validity, comparing participants with KPS <70 and KPS >=70; accordingly, an enrichment strategy targeted approximately 15% of participants with impaired performance status.

Objective: To evaluate the validity,reliability,and responsiveness of the customized Patient-Reported Outcomes version of the Common Teminology Criteria for Adverse Events (PRO-CTCAE)in Chinese patients with Adult-type Difuse Glioma.

Two-round Delphi study, expert advisory meeting and a preliminary test were conducted to jointly determine the feasibility of customized PRO-CTCAE for Chinese patients with Adult-type Difuse Glioma, and to determine customized PRO-CTCAE items.

Indusion criteria:

1. Pathological evidence of Adult-type Diffuse Glioma using 2021 WHO classification criteria.
2. Ages 18 to 85 years.
3. For newly diagnosed patients, from diagnosis to first radiotherapy/chemotherapy.
4. For recurrent patients, from RANO criteria confirm recurrence to first non-surgical treatment.
5. No significant cognitive impairment based on the investigator's judgment.
6. Be able to use mobile phone or computer on their own or with the help of others, be able to read and understand Chinese, have at least elementary school education.
7. Is receiving antitumor therapy and will continue to receive treatment
8. Informed consent completed

Adult-type Diffuse Glioma(n=450)

Screening

V1

Time-window:

1. For newly diagnosed patients, from diagnosis to first radiotherapy/chemotherapy.
2. For recurrent patients, from RANO criteria confirm recurrence to first non-surgical treatment.
3. The proportion of patients with KPS < 70 was > 15%.

Within 42 days from signing ICF

Enrollment

V2

1. Therapies for the past 2 weeks
2. AEs judged by investigator based on CTCAE v5.0
3. Customized PRO-CTCAE
4. QLQ-C30

V2b

One day after V2:
Customized PRO-CTCAE (Order disrupted)

V3-6

Follow-up performs at 6±2 week intervals:

1. Therapies for the past 2 weeks
2. AEs judged by investigator based on CTCAE v5.0
3. Customized PRO-CTCAE
4. QLQ-C30
5. GIC

Long-time follow-up

Follow-up performs at 12±2 week intervals:
Until the study is terminated or the patient dies

Follow-up Schedule

	Visit 1	Visit 2	Visit 2b *	Visit 3	Visit 4	Visit 5	Visit 6	Long term follow up
**Time**	-42 to 0 days	0 day	1 day	6 ± 2 weeks	12 ± 2 weeks	18 ± 2 weeks	24 ± 2 weeks	After Visit 6 Every 12 weeks ± 2 weeks
**Medical data (to be completed by study personnel)**								
**Signed informed consent and investigator confirmed eligibility criteria**	√							
**Basic information** ***(Demographic information such as educational background, economic conditions, living conditions, etc.)***	√							
**Diagnostic Information** ***(Diagnosis and changes in condition)***	√			√	√	√	√	√
**Antineoplastic regimens within the last 2 weeks** ***(Including but not limited to surgery, radiotherapy, drugs, tumour treating fields therapy, etc.)***		√		√	√	√	√	√
**Special treatment regimen within last 2 weeks** ***(Including but not limited to dehydrating agents, oral/injectable steroids, antiepileptics, narcotic analgesics, laxatives, antiemetics, anti-insomnia agents, antidiarrheals, acid suppressants, bronchodilators, inhaled corticosteroids, anxiolytics, antidepressants, etc.)***		√		√	√	√	√	√
**Physician Assessed**		√		√	√	√	√	√

**Adverse Reactions (CTCAE v5.0)** ***(Only include PRO-CTCAE entries)***								
**Physician assessed KPS score**		√		√	√	√	√	√
**Questionnaire form (to be completed by patient)**								
**Customized PRO-CTCAE (51 questions)**		√	√ *(scrambled)*	√	√	√	√	√
**QLQ-C30 (30 questions)**		√		√	√	√	√	√
**GIC (4 questions)**				√	√	√	√	

** Visit 2b only for the randomised 100-patient test-retest subset*

4.2 **Study personnel**

Main site: one statistician, one evaluator, one consultant (from neurosurgery, radiation oncology, or medical oncology), one outpatient nurse, one observer, and one device maintainer.

Subsites: one designated outpatient physician (from neurosurgery, radiation oncology, or medical oncology), one designated outpatient nurse, one designated observer, and one device maintainer.

倾听患者之声

5 Study plan and data collection

The original data collected during the study were derived from the original medical data filled in by the investigator and the original records filled in by the study subjects. The investigator should guide the study subjects to conduct the questionnaire specified in the protocol according to the protocol, conduct follow-up according to the time specified in the protocol, and timely sort out and preserve all relevant records or results. The subjects shall fill in the corresponding data timely and in detail according to the investigator 's requirements and timely deliver it to the investigator for preservation.

5.1 Basic information and clinical information

5 .1.1 General Information

During the screening period, the investigator should evaluate the patient 's current basic information.

Including: patient number, name, date of birth, gender, ethnicity, height, weight, telephone, educational background, annual family income, living conditions.

5 .1.2 Diagnostic information

Visit 1, Visit 3 - 6 and long-term follow-up, the investigator should evaluate the patient 's current diagnostic information.

Including: date of pathological diagnosis, pathological diagnosis, site of primary tumour, size of primary tumour, extent of resection, metastasis, recurrence, W HO grade, molecular diagnostic information (IDH mutation, MGMT promoter mutation, 1p/19q combined deletion, TERT promoter mutation, E GFR amplification, 7 +/10 -, C DKN2A/B mutation, others), concomitant diseases, allergic history, clinical changes (R ANO criteria)

RANO Criteria for Response Assessment incorporating MRI and clinical factors.

Criterion	CR	PR	SD	PD
T1-Gd +	None	≥50% ↓	<50% ↓ to <25% ↑	≥25% ↑†
T2/FLAIR	Stable or ↓	Stable or ↓	Stable or ↓	↑†
New lesion	None	None	None	Present†
Corticosteroids	None	Stable or ↓	Stable or ↓	NA‡
Clinical status	Stable or ↑	Stable or ↑	Stable or ↑	↓†
Requirement for response	All	All	All	Any‡
Summary of HGG response criteria	Requires all of the following: complete disappearance of all enhancing measurable and nonmeasurable disease sustained for at least 4 weeks; no new lesions; stable or improved nonenhancing (T2/FLAIR) lesions; patients must be off corticosteroids (or on physiologic replacement doses only); and stable or improved clinically. Note: Patients with nonmeasurable disease only cannot have achieved CR; the best response possible is SD	Requires all of the following: ≥50% decrease compared with baseline in the sum of products of perpendicular diameters of all measurable enhancing lesions sustained for at least 4 weeks; no progression of nonmeasurable disease; no new lesions; stable or improved nonenhancing (T2/FLAIR) lesions on same or lower dose of corticosteroids compared with baseline scan; the corticosteroid dose at the time of scan evaluation should be no greater than the dose at time of baseline scan; and stable or improved clinically	Requires all of the following: Does not qualify for CR, PR or progression; stable nonenhancing (T2/FLAIR) lesions on the same or lower dose of corticosteroids compared with baseline scan. In the event that the corticosteroid dose was increased for new symptoms and signs without confirmation of disease progression on neuroimaging, and subsequent follow-up imaging shows that this increase in corticosteroids was required because of disease progression, the last scan considered to show SD will be the scan obtained when the corticosteroid dose was equivalent to the baseline dose	Defined by any of the following: ≥25% increase in the sum of the products of perpendicular diameters of enhancing lesions compared with the smallest tumor measurement obtained either at baseline (if no decrease) or best response on stable or increasing doses of corticosteroids†; significant increase in T2/FLAIR nonenhancing lesion on stable or increasing doses of corticosteroids compared with baseline scan or best response after initiation of therapy† not caused by comorbid events (e.g., radiation therapy, demyelination, ischemic injury, infection, seizures, postoperative changes or other treatment effects); any new lesion; clear clinical deterioration not attributable to other causes apart from the tumor (e.g., seizures, medication adverse effects, complications of therapy, cerebrovascular events, infection, etc.) or changes in corticosteroid dose; failure to return for evaluation as a result of death or deteriorating condition; or clear progression of nonmeasurable disease

†Progression occurs when this criterion is met.
‡Increase in corticosteroids alone will not be taken into account in determining progression in the absence of persistent clinical deterioration.
↓: decrease; ↑: increase; CR: Complete response; FLAIR: Fluid-attenuated inversion recovery; HGG: High-grade glioma; NA: Not applicable; PD: Progressive disease; PR: Partial response; SD: Stable disease; T1-Gd +: T1 postgadolinium.
Modified with permission from [4] © American Society of Clinical Oncology (2017). All rights reserved.

5 .1.3 Information on treatment regimen within the last 2 weeks

During Visit 2 - 6 and long-term follow-up, the investigator should evaluate the patient 's anti-tumour treatment regimen and other special treatment regimens within the past 2 weeks.

Anti-tumour treatment regimen within the past 2 weeks, including surgery, radiotherapy, anti-tumour drugs, tumour treating fields therapy, etc.

Special treatment regimen within the last 2 weeks, including: dehydrating drugs, oral/injectable steroids, antiepileptics, narcotic analgesics, laxatives, antiemetics, anti-insomnia drugs, antidiarrheals, acid suppressants, bronchodilators, inhaled corticosteroids, anxiolytics, antidepressants, etc.

**Type of treatment/drug**	
Antineoplastic agents	Chemotherapy drugs, targeted drugs, immune-related drugs
Chemotherapy agents causing skin reactions	Epidermal growth factor receptor inhibitors, docetaxel, gemcitabine, paclitaxel, and pemetrexed
Dehydration drugs	Osmotic dehydrating drugs, diuretic dehydrating drugs

5 .1.4 Physician Assessed Adverse Reactions (CTCAE v5.0)

During Visit 1 - 6 and long-term follow-up, the investigator needs to assess C TCAE v

5.0 entries corresponding to customized P RO-CTCAE entries.

P RO-CTCAE	C TCAE v5.0
**Dysphagia**	Dysphagia
**Taste problems when tasting food or drink**	Dysgeusia
**Decreased appetite**	Anorexia
**Queasy (nausea/regurgitation)**	Nausea
**Vomiting**	Vomiting
**Constipation**	Constipation
**Tachypnea**	Dyspnoea
**Beating or racing heart (palpitations)**	Palpitations
**Paralysis or tingling in hands or feet**	Peripheral sensory nerve disorder
**Dizziness**	Vertigo
**Blurred vision**	Blurred vision
**Flare in front of eyes**	Flash
**Tinnitus (noise in ears)**	Tinnitus
**Inability to concentrate**	Attention disturbance
**Memory (memory) problems**	Memory impairment
**Skin erythema**	Maculopapular rash
**Alopecia**	Alopecia
**Skin pruritus**	Pruritus
**Measles (itchy red pimples on the skin)**	Urticaria
**Bedsores/Pressure sores**	Skin ulcer
**Burn of skin by radiation**	Radiation dermatitis
**Pain (may appear anywhere on the body)**	Pain * (may occur anywhere on the body)
**Headache**	Headache
**Insomnia (including difficulty falling asleep, staying asleep or waking up early)**	Insomnia
**Fatigue (tiredness), tiredness, or lack of energy**	Fatigue
**Anxiety**	Anxiety
**Nothing can cheer you up/cheer you up**	Depression
**Decreased sexual interest**	Libido decreased
**Loss of control of urination (urine leakage)**	Urinary incontinence
**Abnormal decreased sweating**	Hypohidrosis
**Hot flashes (sensation of heat and sweating or rapid heartbeat)**	Hyperhidrosis

** CTCAE 5.0 grades are detailed in Appendix 4.*

5 .1.5 KPS score assessed by physician

During Visit 1 - 6 and long-term follow-up, the investigator should assess the patient 's current K PS score.

Karnofsky Scoring Criteria Table (KPS Score)

**Performance status**	**Score**
**Normal, no symptoms and signs**	100 points

**Able to perform normal activities with minor symptoms and signs**	90 points
**Normal activity with reluctance, with some symptoms or signs**	80 points
**Cares for self but does not maintain normal life and work**	70 points
**Capable of most self-care, but occasionally requires assistance**	60 points
**Often require care**	50 points
**Cannot carry on any selfcare and requires special care and assistance**	40 points
**SEVERE CARE ABSENT**	30 points
**Very ill, requiring hospitalization and aggressive supportive care**	20 points
**Critically ill, near death**	10 points
**Death**	0 point

5 .1.5 Questionnaire Form

- **Customized P RO-CTCAE Entry Table for Adult-type diffuse glioma Patients**

Oral	
Dry mouth	S
Difficulty swallowing	S
Mouth/throat sores	SI
Cracking at the corners of the mouth(cheilosis/cheilitis)	S
Voice quality changes	P
Hoarseness	S
**Gastrointesinal**	
Taste changes	S
Decreased appetite	SI
Nausea	FS
Vomiting	FS
Heartburn	FS
Gas	P
Bloating	FS
Hiccups	FS
Constipation	S
Diarrhea	F
Abdominal pain	FSI
Fecal incontinence	FI

Respiratory	
Shortness	SI
Cough	SI
Wheezing	S
**Cardio/Circulatory**	
Swelling	FSI
Heart palpitations	FS
**Neurological**	
Numbness/tingling	SI
Dizziness	SI
**Visual/Perceptual**	
Blurred vision	SI
Flashing light	P
Visual floates	P
Watery eyes	SI
Ringing in ears	S
**Attention/Memory**	
Concentration	SI
Memory	SI

Cutaneous	
Rash	P
Skin dryness	S
Acne	S
Hair loss	A
Itching	S
Hives	P
Hand-foot syndrome	S
Nail loss	P
Nail ridging	P
Nail discoloration	P
Sensitivity to sunlight	P
Bed/pressure sores	P
Radiation skin reaction	S
Skin darkening	P
Stretch marks	P

Pain	
General pain	FSI
Headache	FSI
Muscle pain	FSI
Joint pain	FSI
**Sleep/Wake**	
Insomnia	SI
Fatigue	SI
**Mood**	
Anxious	FSI
*Discouraged	FSI
*Sad	FSI
**Sexual**	
Achieve and maintain erection	S
Ejaculation	F
Decreased libido	S
Delayed orgasm	P
Unable to have orgasm	P
Pain w/sexual intercourse	S

Genitourinary	
Irregular period/vaginal bleeding	P
Missed expected menstrual period	P
Vaginal discharge	A
Vaginal dryness	S
Painful urination	S
Urinary urgency	FI
Urinary frequency	FI
Change in usual urine color	P
Urinary incontinence	FI
**Miscellaneous**	
Breast swelling and tenderness	S
Bruising	P
Chills	FS
Increased sweating	FS
Decreased sweating	P
Hot flashes	FS
Nosebleed	FS
Pain and swelling at injection site	P
Body odor	S

pro-ctcae_chinese-simplified_629C

VIPRONICA

- **EORTC QLQ-C30 (V3.0) Chinese Scale**
  See Attachment 1.

- **Global Impression of Change**

1. How has your overall quality of life changed from the last time you filled out the questionnaire?
2. How did your mood state change compared to the last time you filled out the questionnaire?
3. Compared with the last time you filled out the questionnaire, how has your physical condition changed?
4. How did your skin condition change compared to the last time you filled out the questionnaire?
Option: "markedly improved", "somewhat improved", "slightly improved", "unchanged", "slightly aggravated", "somewhat aggravated", "markedly aggravated"

5.2 Study procedures

After the investigator has explained the study in detail and obtained written informed consent, screening assessments are completed to confirm eligibility and to record baseline demographic and clinical information (see section 5.1).

Participants may complete the customised PRO-CTCAE using a tablet, mobile phone, personal computer, or paper form through a centrally managed secure PRO platform. For electronic completion, selected multi-item symptoms may be administered using a collapsed-question design to reduce respondent burden while preserving the original item wording, response options, and scoring structure.

Participants are encouraged to complete the questionnaire independently; however, study staff may provide technical assistance with device use where required.

5.2.1 Visit schedule

	**Visit 1**	**Visit 2**	**Visit 2b** *	**Visit 3**	**Visit 4**	**Visit 5**	**Visit 6**	**Long term follow up**
**Time**	-42 to 0 days	0 day	1 day	6 ± 2 weeks	12 ± 2 weeks	18 ± 2 weeks	24 ± 2 weeks	After Visit 6 Every 12 weeks ± 2 weeks
**Medical data (to be completed by study personnel)**								
**Signed informed consent and investigator confirmed eligibility criteria**	√							
**Basic information** ***(Demographic information such as educational background, economic conditions, living conditions, etc.)***	√							
**Diagnostic Information** ***(Diagnosis and changes in condition)***	√			√	√	√	√	√
**Antineoplastic regimens within the last 2 weeks** ***(Including but not limited to surgery, radiotherapy, drugs, tumour treating fields***		√		√	√	√	√	√

therapy, etc.)								
**Special treatment regimen within last 2 weeks** ***(Including but not limited to dehydrating agents, oral/injectable steroids, antiepileptics, narcotic analgesics, laxatives, antiemetics, anti-insomnia agents, antidiarrheals, acid suppressants, bronchodilators, inhaled corticosteroids, anxiolytics, antidepressants, etc.)***		√		√	√	√	√	√
**Physician Assessed Adverse Reactions (CTCAE v5.0)** ***(Only include PRO-CTCAE entries)***		√		√	√	√	√	√
**Physician assessed KPS score**		√		√	√	√	√	√
**Questionnaire form (to be completed by patient)**								
**Customized PRO-CTCAE (51 questions)**		√	√ *(scrambled)*	√	√	√	√	√
**QLQ-C30 (30 questions)**		√		√	√	√	√	√
**GIC (4 questions)**				√	√	√	√	

** Visit 2b only for the randomised 100-patient test-retest subset*

5.2.2 Screening and baseline assessments

Visit 1 (screening): Day -42 to Day 0

Participants must provide written informed consent before any screening procedures are undertaken.

At Visit 1, the investigator completes the following assessments and procedures:

- Basic information: participant number, name, date of birth, sex, ethnicity, height, weight, telephone number, educational level, annual household income, and living circumstances.
- Diagnostic information: date of pathological diagnosis, integrated diagnosis, primary tumour site, tumour size, extent of resection, metastasis, recurrence status, WHO grade, molecular diagnostic information (including IDH, MGMT promoter, 1p/19q, TERT promoter, EGFR, chromosome 7 gain/10 loss, CDKN2A/B, and other relevant markers), comorbidities, allergy history, and clinical change according to RANO criteria.

Participants who meet screening requirements proceed to enrolment.

5.2.3 Follow-up assessments

Visit 2 (V2, baseline): Day 0

Before Visit 2, participants are reconfirmed to meet inclusion criteria and not to meet exclusion or discontinuation criteria. Visit 2 should be completed within 42 days of screening.

At Visit 2, the investigator completes the following assessments and procedures:

- Anti-tumour treatment received within the previous 2 weeks, including surgery, radiotherapy, anti-tumour drugs, tumour treating fields therapy, and other relevant treatments.
- Supportive or concomitant treatments received within the previous 2 weeks, including dehydrating agents, oral or injectable steroids, antiepileptics, opioid

VPRONICA
倾听患者之声

analgesics, laxatives, antiemetics, hypnotics, antidiarrhoeals, acid suppressants, bronchodilators, inhaled corticosteroids, anxiolytics, and antidepressants; date and time of questionnaire completion are also recorded.

- Clinician-reported adverse events (CTCAE version 5.0).
- KPS score.

At Visit 2, participants complete the following questionnaires and assessments:

- Customised PRO-CTCAE.
- EORTC QLQ-C30.

Questionnaires may be completed on a tablet, mobile phone, personal computer, or paper form. For paper completion, study staff confirm completeness, record the completion time, and enter the data into the online PRO platform.

Visit 2b (V2b): Day 1

Visit 2b is performed on the first working day after Visit 2 in the randomised 100-patient test-retest subset. The customised PRO-CTCAE administered at Visit 2b is identical in content to that used at Visit 2, although the order of items is randomised.

At Visit 2b, the investigator completes the following procedures:

- Reminder to complete the questionnaire.
- Date and time of questionnaire completion.

At Visit 2b, participants complete the following assessment:

- Customised PRO-CTCAE (scrambled order).

Questionnaires may be completed on a tablet, mobile phone, personal computer, or paper form. For paper completion, study staff confirm completeness, record the completion time, and enter the data into the online PRO platform.

Visit 3 (V3): Week 6 (+/- 2 weeks)

Visit 3 is performed 6 (+/- 2) weeks after Visit 2.

At Visit 3, the investigator completes the following assessments and procedures:

- Updated diagnostic information, including recurrence status and clinical change according to RANO criteria where applicable.
- Anti-tumour treatment received within the previous 2 weeks, including surgery, radiotherapy, anti-tumour drugs, tumour treating fields therapy, and other relevant treatments.
- Supportive or concomitant treatments received within the previous 2 weeks; date and time of questionnaire completion are also recorded.
- Clinician-reported adverse events (CTCAE version 5.0).
- KPS score.

At Visit 3, participants complete the following questionnaires and assessments:

- Customised PRO-CTCAE.
- EORTC QLQ-C30.
- Global Impression of Change (GIC).

Questionnaires may be completed on a tablet, mobile phone, personal computer, or paper form. For paper completion, study staff confirm completeness, record the completion time, and enter the data into the online PRO platform.

Visits 4-6 (V4-V6) occur at Weeks 12 (+/- 2), 18 (+/- 2), and 24 (+/- 2), respectively. The assessments performed are the same as those undertaken at Visit 3.

5.2.4 Long-term follow-up

Participants enter long-term follow-up after Visit 6 (V6).

The investigator should complete the following assessments or procedures:

- Data Verification.

In long-term follow-up, the investigator should complete the following assessments or procedures:

- Diagnostic information: date of pathological diagnosis, pathological diagnosis, primary tumour site, primary tumour size, resection degree, metastasis, recurrence, WHO grade, molecular diagnostic information (IDH mutation, MGMT promoter mutation, 1p/19q combined deletion, TERT promoter mutation, E GFR amplification, 7 +/10 -, C DKN2A/B mutation, others), concomitant diseases, allergic history, clinical changes (RANO criteria)
- Anti-tumour treatment regimen within the past 2 weeks, including surgery, radiotherapy, anti-tumour drugs, tu mour treating fields therapy, etc.
- Special treatment regimen within the past 2 weeks, including: dehydrating drugs, oral/injectable steroids, antiepileptics, narcotic analgesics, laxatives, antiemetics, anti-insomnia drugs, antidiarrheals, acid suppressants, bronchodilators, inhaled corticosteroids, anxiolytics, antidepressants, etc. Time patient completed questionnaire.
- Physician assessed adverse reactions (CTCAE v5.0).
- KPS score

In long-term follow-up, subjects should complete the following assessments or procedures:

- Customized PRO-CTCAE

VERONICA 倾听患者之声

5.4 Primary endpoint evaluation

Primary endpoint evaluations: psychometric performance of the customised PRO-CTCAE scale in Chinese adults with adult-type diffuse glioma, including validity, reliability, and responsiveness.

Validity was primarily assessed at Visit 4 using prespecified anchors, including the EORTC QLQ-C30, clinician-reported KPS, and treatment-related clinical variables.

Reliability was assessed as test-retest reliability by comparing patient-completed PRO-CTCAE responses at Visit 2 and Visit 2b in the test-retest subset.

Responsiveness was assessed by examining the consistency of change in PRO-CTCAE scores between Visit 3 and Visit 4 with corresponding patient-reported Global Impression of Change (GIC) ratings.

In this study, the EORTC QLQ-C30, KPS score, and specific treatment regimen served as anchors for predefined psychometric analyses.

5.3.1 Construct validity

Construct validity was assessed using predefined clinical and questionnaire-based anchors. In psychometric studies, anchors are external criteria that support interpretation of whether a scale measures the intended construct.

In this study, the European Organisation for Research and Treatment of Cancer Quality of Life Questionnaire-Core 30 (EORTC QLQ-C30), together with clinician-reported KPS and treatment-related clinical variables, was used as an anchor framework for the assessment of convergent and known-groups validity. The official Chinese version of the EORTC QLQ-C30 was used throughout.

Clinician-reported KPS and treatment information were collected at each visit via the case report form, including surgery, radiotherapy, systemic anti-tumour drugs, tumour treating fields therapy, and selected supportive treatments.

Convergent validity analyses examined the associations between predefined customised PRO-CTCAE items and corresponding EORTC QLQ-C30 domains. The primary analysis of validity was conducted at Visit 4.

Predefined mappings between customised PRO-CTCAE items and EORTC QLQ-C30 domains are shown in Table S2.

NO.	PRO-CTCAE item	PRO-CTCAE System	QLQ-C30 Functional or Symptom Domain	QLQ-C30 Overall	QLQ-C30 Item Numbers
1a	Difficulty swallowing S	Oral	Physical functioning	Global health status/QOL	1,2,3,4,5+29,30
2a	Taste changes S	Gastrointestinal	Physical functioning	Global health status/QOL	1,2,3,4,5+29,30
3a	Decreased appetite S	Gastrointestinal	Appetite loss	Global health status/QOL	13+29,30
3b	Decreased appetite I	Gastrointestinal	Appetite loss	Global health status/QOL	13+29,30
4a	Nausea F	Gastrointestinal	Nausea and vomiting	Global health status/QOL	14,15+29,30
4b	Nausea S	Gastrointestinal	Nausea and vomiting	Global health status/QOL	14,15+29,30
5a	Vomiting F	Gastrointestinal	Nausea and vomiting	Global health status/QOL	14,15+29,30
5b	Vomiting S	Gastrointestinal	Nausea and vomiting	Global health status/QOL	14,15+29,30
6a	Constipation S	Gastrointestinal	Constipation	Global health status/QOL	16+29,30
7a	Shortness of breath S	Respiratory	Dyspnoea	Global health status/QOL	8+29,30

NO.	PRO-CTCAE item	PRO-CTCAE System	QLQ-C30 Functional or Symptom Domain	QLQ-C30 Overall	QLQ-C30 Item Numbers
7b	Shortness of breath I	Respiratory	Dyspnoea	Global health status/QOL	8+29,30
8a	Heart palpitations F	Cardio/Circulatory	Physical functioning	Global health status/QOL	1,2,3,4,5+29,30
8b	Heart palpitations S	Cardio/Circulatory	Physical functioning	Global health status/QOL	1,2,3,4,5+29,30
9a	Rash P	Cutaneous	Physical functioning	Global health status/QOL	1,2,3,4,5+29,30
10a	Hair loss A	Cutaneous	Physical functioning	Global health status/QOL	1,2,3,4,5+29,30
11a	Itching S	Cutaneous	Physical functioning	Global health status/QOL	1,2,3,4,5+29,30
12a	Hives P	Cutaneous	Physical functioning	Global health status/QOL	1,2,3,4,5+29,30
13a	Bed/pressure sores P	Cutaneous	Physical functioning	Global health status/QOL	1,2,3,4,5+29,30
14a	Radiation skin reaction S	Cutaneous	Physical functioning	Global health status/QOL	1,2,3,4,5+29,30
15a	Numbness & tingling S	Neurological	Physical functioning	Global health status/QOL	1,2,3,4,5+29,30
15b	Numbness & tingling I	Neurological	Physical functioning	Global health status/QOL	1,2,3,4,5+29,30
16a	Dizziness S	Neurological	Physical functioning	Global health status/QOL	1,2,3,4,5+29,30
16b	Dizziness I	Neurological	Physical functioning	Global health status/QOL	1,2,3,4,5+29,30
17a	Blurred vision S	Visual/Perceptual	Physical functioning	Global health status/QOL	1,2,3,4,5+29,30
17b	Blurred vision I	Visual/Perceptual	Physical functioning	Global health status/QOL	1,2,3,4,5+29,30
18a	Flashing lights P	Visual/Perceptual	Physical functioning	Global health status/QOL	1,2,3,4,5+29,30
19a	Ringing in ears S	Visual/Perceptual	Physical functioning	Global health status/QOL	1,2,3,4,5+29,30
20a	Concentration S	Attention/Memory	Cognitive functioning	Global health status/QOL	20,25+29,30
20b	Concentration I	Attention/Memory	Cognitive functioning	Global health status/QOL	20,25+29,30
21a	Memory S	Attention/Memory	Cognitive functioning	Global health status/QOL	20,25+29,30
21b	Memory I	Attention/Memory	Cognitive functioning	Global health status/QOL	20,25+29,30
22a	General pain F	Pain	Pain	Global health status/QOL	9,19+29,30
22b	General pain S	Pain	Pain	Global health status/QOL	9,19+29,30
22c	General pain I	Pain	Pain	Global health status/QOL	9,19+29,30
23a	Headache F	Pain	Pain	Global health status/QOL	9,19+29,30
23b	Headache S	Pain	Pain	Global health status/QOL	9,19+29,30
23c	Headache I	Pain	Pain	Global health status/QOL	9,19+29,30
24a	Insomnia S	Sleep/Wake	Insomnia	Global health status/QOL	11+29,30
24b	Insomnia I	Sleep/Wake	Insomnia	Global health status/QOL	11+29,30
25a	Fatigue S	Sleep/Wake	Fatigue	Global health status/QOL	10,12,18+29,30
25b	Fatigue I	Sleep/Wake	Fatigue	Global health status/QOL	10,12,18+29,30
26a	Anxious F	Mood	Emotional functioning	Global health status/QOL	21,22,23,24+29,30
26b	Anxious S	Mood	Emotional functioning	Global health status/QOL	21,22,23,24+29,30
26c	Anxious I	Mood	Emotional functioning	Global health status/QOL	21,22,23,24+29,30
27a	Discouraged F	Mood	Emotional functioning	Global health status/QOL	21,22,23,24+29,30
27b	Discouraged S	Mood	Emotional functioning	Global health status/QOL	21,22,23,24+29,30
27c	Discouraged I	Mood	Emotional functioning	Global health status/QOL	21,22,23,24+29,30
28a	Urinary incontinence F	Gynecologic/Urinary	Physical functioning	Global health status/QOL	1,2,3,4,5+29,30
28b	Urinary incontinence I	Gynecologic/Urinary	Physical functioning	Global health status/QOL	1,2,3,4,5+29,30
29a	Decreased libido S	Sexual	Emotional functioning	Global health status/QOL	21,22,23,24+29,30
30a	Decreased sweating P	Miscellaneous	Physical functioning	Global health status/QOL	1,2,3,4,5+29,30
31a	Hot flashes F	Miscellaneous	Physical functioning	Global health status/QOL	1,2,3,4,5+29,30
31b	Hot flashes S	Miscellaneous	Physical functioning	Global health status/QOL	1,2,3,4,5+29,30

Abbreviations: F, Frequency; I, Interference; S, Severity; P, Presence/Absence; A, Amount

5.3.2 Test-retest reliability

Test-retest reliability was used to evaluate score reproducibility when health status was expected to remain stable. In the test-retest subset, the customised PRO-CTCAE scale was administered again on the first working day after Visit 2 (Visit 2b). The Visit 2b questionnaire was identical in content to the Visit 2 questionnaire, although the order of items was randomised.

5.3.3 Responsiveness

Responsiveness refers to the ability of the scale to detect clinically meaningful change in patient-reported outcomes over time.

At Visits 3-6, patients completed four Global Impression of Change (GIC) items corresponding to overall quality of life, emotional status, physical condition, and skin status since the previous assessment. Each item used a seven-point ordered response scale ranging from markedly improved to markedly worsened. Standardised response means (SRMs) and ordered trend analyses were then used to compare changes in customised PRO-CTCAE scores across GIC-defined change groups.

The primary responsiveness analysis examined the consistency of change in patient-completed PRO-CTCAE scores between Visit 3 and Visit 4 with corresponding GIC ratings at Visit 4.

1. How has your overall quality of life changed from the last time you filled out the questionnaire?
2. How did your mood state change compared to the last time you filled out the questionnaire?
3. Compared with the last time you filled out the questionnaire, how has your physical condition changed?
4. How did your skin condition change compared to the last time you filled out the questionnaire?
Option: "markedly improved", "somewhat improved", "slightly improved", "unchanged", "slightly aggravated", "somewhat aggravated", "markedly aggravated"

NO.	PRO-CTCAE item	PRO-CTCAE System	GIC Overall	GIC Special Functional Domain
1a	Difficulty swallowing S	Oral	Global quality of life	Physical condition
2a	Taste changes S	Gastrointestinal	Global quality of life	Physical condition
3a	Decreased appetite S	Gastrointestinal	Global quality of life	Emotional state
3b	Decreased appetite I	Gastrointestinal	Global quality of life	Emotional state
4a	Nausea F	Gastrointestinal	Global quality of life	Physical condition
4b	Nausea S	Gastrointestinal	Global quality of life	Physical condition
5a	Vomiting F	Gastrointestinal	Global quality of life	Physical condition
5b	Vomiting S	Gastrointestinal	Global quality of life	Physical condition
6a	Constipation S	Gastrointestinal	Global quality of life	Physical condition
7a	Shortness of breath S	Respiratory	Global quality of life	Physical condition
7b	Shortness of breath I	Respiratory	Global quality of life	Physical condition
8a	Heart palpitations F	Cardio/Circulatory	Global quality of life	Physical condition
8b	Heart palpitations S	Cardio/Circulatory	Global quality of life	Physical condition
9a	Rash P	Cutaneous	Global quality of life	Skin condition

NO.	PRO-CTCAE item	PRO-CTCAE System	GIC Overall	GIC Special Functional Domain
10a	Hair loss A	Cutaneous	Global quality of life	Skin condition
11a	Itching S	Cutaneous	Global quality of life	Skin condition
12a	Hives P	Cutaneous	Global quality of life	Skin condition
13a	Bed/pressure sores P	Cutaneous	Global quality of life	Skin condition
14a	Radiation skin reaction S	Cutaneous	Global quality of life	Skin condition
15a	Numbness & tingling S	Neurological	Global quality of life	Physical condition
15b	Numbness & tingling I	Neurological	Global quality of life	Physical condition
16a	Dizziness S	Neurological	Global quality of life	Physical condition
16b	Dizziness I	Neurological	Global quality of life	Physical condition
17a	Blurred vision S	Visual/Perceptual	Global quality of life	Physical condition
17b	Blurred vision I	Visual/Perceptual	Global quality of life	Physical condition
18a	Flashing lights P	Visual/Perceptual	Global quality of life	Physical condition
19a	Ringing in ears S	Visual/Perceptual	Global quality of life	Physical condition
20a	Concentration S	Attention/Memory	Global quality of life	Emotional state
20b	Concentration I	Attention/Memory	Global quality of life	Emotional state
21a	Memory S	Attention/Memory	Global quality of life	Emotional state
21b	Memory I	Attention/Memory	Global quality of life	Emotional state
22a	General pain F	Pain	Global quality of life	Emotional state
22b	General pain S	Pain	Global quality of life	Emotional state
22c	General pain I	Pain	Global quality of life	Emotional state
23a	Headache F	Pain	Global quality of life	Emotional state
23b	Headache S	Pain	Global quality of life	Emotional state
23c	Headache I	Pain	Global quality of life	Emotional state
24a	Insomnia S	Sleep/Wake	Global quality of life	Emotional state
24b	Insomnia I	Sleep/Wake	Global quality of life	Emotional state
25a	Fatigue S	Sleep/Wake	Global quality of life	Emotional state
25b	Fatigue I	Sleep/Wake	Global quality of life	Emotional state
26a	Anxious F	Mood	Global quality of life	Emotional state
26b	Anxious S	Mood	Global quality of life	Emotional state
26c	Anxious I	Mood	Global quality of life	Emotional state
27a	Discouraged F	Mood	Global quality of life	Emotional state
27b	Discouraged S	Mood	Global quality of life	Emotional state
27c	Discouraged I	Mood	Global quality of life	Emotional state
28a	Urinary incontinence F	Gynecologic/Urinary	Global quality of life	Physical condition
28b	Urinary incontinence I	Gynecologic/Urinary	Global quality of life	Physical condition
29a	Decreased libido S	Sexual	Global quality of life	Emotional state
30a	Decreased sweating P	Miscellaneous	Global quality of life	Physical condition
31a	Hot flashes F	Miscellaneous	Global quality of life	Physical condition
31b	Hot flashes S	Miscellaneous	Global quality of life	Physical condition

Abbreviations: F, Frequency; I, Interference; S, Severity; P, Presence/Absence; A, Amount

5.5 Secondary and exploratory endpoint evaluations

- Secondary and exploratory analyses included changes in PRO-CTCAE scores in the context of tumour progression or recurrence according to RANO criteria.
- Additional validity analyses were undertaken at Visits 2, 3, 5, and 6.
- Additional responsiveness analyses compared changes between Visit 2 and Visit 3, Visit 4 and Visit 5, and Visit 5 and Visit 6.
- Exploratory analyses also examined changes in PRO-CTCAE scores according to treatment exposure, including radiotherapy, temozolomide-based regimens, and tumour treating fields.
- Exploratory longitudinal analyses assessed change in PRO-CTCAE scores during the first 6 months of non-surgical treatment in Chinese adults with adult-type diffuse glioma.
- Exploratory comparative analyses evaluated differences between clinician-reported adverse events (CTCAE) and patient-reported adverse events (PRO-CTCAE) during the first 6 months of non-surgical treatment.
- Changes in physician-reported adverse reactions (CTCAE) within 6 months of first non-surgical treatment in Chinese patients with adult-type diffuse glioma.

5.6 Serious adverse events

This study was observational and did not alter the patient's diagnostic or therapeutic management.

VOPRONICA
倾听患者之声

6 Study conducts and management

6.1 Study procedures

Visit 1 (V1, screening): from Day -42 to Day 0, conducted in the outpatient clinic or inpatient ward. After written informed consent was obtained, study staff completed screening, recorded baseline demographic and diagnostic information, and informed participants of the timing and mode of subsequent follow-up.

Visit 2 (V2, baseline): Day 0, conducted in the outpatient clinic or inpatient ward. Study staff recorded treatment information, clinician-reported adverse events (CTCAE), and KPS, and confirmed completion of the customised PRO-CTCAE and EORTC QLQ-C30.

Visit 2b (V2b): Day 1, conducted in the randomised 100-patient test-retest subset in the outpatient clinic, inpatient ward, or remotely. Study staff confirmed completion of the scrambled customised PRO-CTCAE.

Visits 3-6 (V3-V6): conducted at Weeks 6 +/- 2, 12 +/- 2, 18 +/- 2, and 24 +/- 2 after Visit 2 in the outpatient clinic, inpatient ward, or remotely. Study staff recorded treatment information, clinician-reported adverse events (CTCAE), and KPS, and confirmed completion of the customised PRO-CTCAE, EORTC QLQ-C30, and GIC.

Long-term follow-up: conducted every 12 +/- 2 weeks after Visit 6 until study discontinuation or death, in the outpatient clinic, inpatient ward, or remotely. Study staff recorded treatment information, clinician-reported adverse events (CTCAE), and KPS, and confirmed completion of the customised PRO-CTCAE and EORTC QLQ-C30.

	**V 1**	**V 2**	**V 2b**	**V 3-6**	**Long term follow up**
	- 7 - 0 days	0 day	1 day	6/12/18/24 weeks (± 2 weeks)	V 6 after Every 12 weeks ± 2 weeks
	Outpatient/inpatient ward	Outpatient/Inpatient Ward/Online	Outpatient/Inpatient Ward/Online	Outpatient/Inpatient Ward/Online	Outpatient/Inpatient Ward/Online
**Basic information**	● Screened Subjects<br>● Signed informed consent<br>● Inform the subject of the timing and manner of subsequent follow-up				
**Diagnosis and treatment information**	● Record clinic information	● Record clinic information<br>● Confirm subject completes corresponding questionnaire	● Confirm subject completes corresponding questionnaire	● Record clinic information<br>● Confirm subject completes corresponding questionnaire	● Record clinic information<br>● Confirm subject completes corresponding questionnaire
**Subjects**	● Signed informed consent	Complete the following questionnaires:<br>● PRO-CTCAE	Completed the following questionnaires (randomised 1 00	Complete the following questionnaires:<br>● PRO-CTCAE	Complete the following questionnaires:<br>● PRO-CTCAE

		● C30	patients):<br>● PRO-CTCAE (scrambled version)	● C30<br>● G IC	● C30

At each participating site, a designated outpatient physician, nurse, observer, and device maintainer were assigned according to local staffing arrangements.

6.2 Study schedule and expected completion time

Planned study timeline:

January 2022 to June 2022 for preparation and pilot work;

June 2022 to June 2023 for enrolment of prospective study participants;

June 2023 onwards for data cleaning, analysis, manuscript preparation, and submission.

Responsibilities were distributed across the coordinating centre, the electronic data capture provider, and participating sites according to predefined operational roles.Participating sites were responsible for providing clinical information and follow-up data in accordance with the study protocol and data-management plan.

Site G: ① Provide clinical information and follow-up data.

Site H: ① Clinical information and follow-up data provided.

Site I: ① Provide clinical information and follow-up data.

Site J: ① Clinical information and follow-up data provided.

Site K: ① Clinical information and follow-up data provided.

Site L: ① Clinical information and follow-up data provided.

Site M: ① Clinical information and follow-up data provided.

Site N: ① Clinical information and follow-up data provided.

Site O: ① Provide clinical information and follow-up data.

Site P: ① Clinical information and follow-up data provided.

6.3 Potential benefits to participants

This study was exploratory and was not expected to provide immediate direct benefit to participants. However, by informing development and validation of a disease-specific patient-reported adverse-event instrument, the study may improve future assessment of treatment burden, support more patient-centred care, and benefit subsequent patients with adult-type diffuse glioma.

倾听患者之声

7 Statistical analysis

7.1 Scale data: basic descriptive statistics

Baseline demographic and clinical characteristics were summarised descriptively. Continuous variables with an approximately normal distribution were described using mean (standard deviation), and non-normally distributed continuous variables were described using median (interquartile range). Categorical variables were summarised using frequencies and percentages.

7.2 Psychometric evaluation

7.2.1 Reliability analysis

Test-retest reliability was assessed using the intraclass correlation coefficient (ICC) for each item score between Visit 2 and Visit 2b. An ICC of 0.70 or greater was considered acceptable.

7.2.2 Validity analysis

Content validity for the customised scale was supported by the Delphi process in Part 1, including item-level content validity indices. In Part 2, convergent validity and known-groups validity were formally evaluated.

For convergent validity, Pearson correlation coefficients were calculated between each predefined customised PRO-CTCAE item and the corresponding EORTC QLQ-C30 total, symptom, or functional domain score. For known-groups validity, participants were divided according to KPS (<70 vs >=70); five-level items were compared using two-sample t tests, dichotomous items were compared using chi-square tests, and Cohen's d was used to quantify effect size (0.2, 0.5, and 0.8 representing small, medium, and large effects, respectively).

7.2.3 Responsiveness analysis

Responsiveness was assessed according to GIC-defined change groups. Participants were categorised as worsened, unchanged, or improved, and the Jonckheere-Terpstra test was used to examine ordered differences in customised PRO-CTCAE scores across groups. Standardised response means (SRMs) were also calculated. For predefined item-domain pairs, Pearson correlation coefficients were calculated between changes in customised PRO-CTCAE scores and changes in the corresponding EORTC QLQ-C30 scores.

The electronic data capture (EDC) system was used to build the study database. Unless otherwise specified, all p values were two-sided and the significance level was alpha=0.05. All statistical analyses were performed using R version 4.4.2.

7.3 Sample size calculation

Sample size was based on the precision of prevalence estimates for patient-reported

adverse events captured by the 31 symptoms included in the customised PRO-CTCAE scale, rather than on hypothesis testing for the psychometric endpoints.

Assuming the most conservative scenario of p=0.50, a two-sided 95% confidence interval with a half-width of 0.05 required 384 analysable questionnaires. Allowing for an anticipated 15.0% non-evaluable rate, the target enrolment was increased to 450 participants. An enrichment strategy was used to ensure a sufficient anchor subgroup for known-groups validity analyses based on KPS by targeting approximately 60-70 participants with KPS <70.

v. Investigator Responsibilities

This clinical study was conducted in accordance with the principles of Good Clinical Practice (GCP), the Declaration of Helsinki, and applicable regulatory requirements.

1. The investigator responsible for the clinical investigation shall meet the following conditions:
   (1) Having corresponding professional and technical positions and practicing qualification in medical institutions;
   (2) Having the professional knowledge and experience required in the study protocol;
   (3) Having rich experience in clinical research methods or being able to obtain academic guidance from experienced researchers of this unit;
   (4) Be familiar with the materials and literatures related to the clinical study provided by the sponsor;
   (5) Having the right to control the personnel participating in the research and use the equipment required for the research.

2. The investigator must thoroughly read and understand the contents of the study protocol and strictly follow the protocol.
3. The investigator should understand and be familiar with the role of the study scale (including relevant data on the scale), and should also master all new information related to the scale found during the conduct of the clinical study.
4. The investigator must conduct the clinical study in a medical institution equipped with good medical facilities, laboratory equipment and personnel, and shall have all facilities for dealing with emergencies to ensure the safety of the study subjects. Laboratory test results should be accurate and reliable.
5. The investigator was required to obtain institutional permission to conduct the study, ensure that adequate time and staffing were available, and explain study procedures and responsibilities to all site personnel.
6. The investigator was required to explain the study in detail to potential participants and to obtain informed consent before any study-specific procedures were undertaken.
7. The investigator is responsible for making medical decisions related to the clinical study to ensure that study subjects receive appropriate treatment in case of adverse events during the study.
8. The investigator is obliged to take necessary measures to ensure the safety of the subjects and record them. In case of any serious adverse event during the clinical study, the investigator should immediately take appropriate therapeutic measures for the study subjects and report to the drug regulatory authorities, health administrative authorities and ethics committee, and sign and date the report.
9. The investigator shall ensure that the data are factually, accurately, completely, timely and legally recorded in the medical records and case report forms.
10. The investigator should accept the monitoring and audit of the monitor or auditor dispatched by the sponsor as well as the audit and inspection by drug regulatory

authorities to ensure the quality of the clinical study.

11. The investigator shall agree with the sponsor on the expenses related to the clinical study and specify in the contract. During the clinical study, the investigator shall not charge the study subjects for the study scale.
12. Upon completion of the clinical study, the investigator must write the final report, sign and date it and send it to the sponsor.
13. In case of terminating a clinical study, the investigator must inform the study subject, the Ethics Committee and the drug regulatory authority and clarify the reasons.

VERONICA
倾听患者之声

vi. Ethical, legal, and administrative provisions

Throughout the conduct of the study, scientific validity and data reliability were to be ensured alongside full protection of the rights, safety, and wellbeing of study participants. Ethics review and informed consent were central safeguards.

1. Ethics Committee

The Ethics Committee was responsible for reviewing whether the study protocol and related study materials complied with ethical standards, the Declaration of Helsinki, and Good Clinical Practice, with particular attention to the protection of participants' rights and interests.

For publication purposes, the lead approving authority was the Huashan Institutional Review Board (HIRB), Huashan Hospital, Fudan University (approval number KY2022-681). The full names of all approving authorities and the corresponding approval numbers for the 13 participating centres are provided in Supplementary Table S1 of the manuscript.

1.1 Whether the investigator 's qualification, experience, sufficient time to participate in the clinical study, staffing and equipment conditions meet the study requirements;

1.2 Whether the study protocol fully considers ethical principles, including study purpose, possible risks and benefits to study subjects and other personnel, and scientificity of study design;

1.3 Selection method of study subjects, whether the information materials related to this study are complete and easy to understand when providing study subjects (or their families, guardians and legal representatives), and whether the method of obtaining informed consent is appropriate;

1.4 Treatment measures taken when the subjects are damaged or even die due to participation in the clinical study;

1.5 Whether the amendments proposed for the study protocol are acceptable;

1.6 Periodically review the risk degree of study subjects during the clinical study.

2 Informed consent

The investigator was responsible for explaining the objectives, methods, potential benefits, and possible risks of the study to each participant and for obtaining written informed consent before any study procedures were initiated. Where a participant could not sign independently, consent was to be obtained from a legally authorised representative in accordance with applicable regulations.

VOPRONICA 倾听患者之声

The original informed consent form was to be signed and dated by the participant or representative and retained securely by the investigator. Documentation of informed consent was also to be recorded in the case report form and source documents.

Participation required use of an Ethics Committee-approved informed consent form, and all participation was voluntary.

3 Protocol amendment

Any amendment to the study protocol was to be documented in detail and submitted to the Ethics Committee for review and written approval before implementation, unless immediate action was required to protect participant safety.

4 Confidentiality agreement and study subject privacy

Researchers must ensure that the privacy of study subjects is maintained.

The Investigator must properly keep the names, addresses and corresponding enrolment forms of the relevant study subjects.

5 Data archiving and data management

According to the requirements of relevant laws and regulations, the investigator should properly keep the source documents and records of the clinical study.

The raw data collected during the study are derived from the original medical data filled in by the investigator and the original records filled in by the subject. The investigator shall instruct the subject to receive protocol-specified treatment according to the protocol, follow up according to the time specified in the protocol, and timely sort out and preserve all the relevant records or results. Subjects shall fill in corresponding data timely and exhaustively according to the investigator 's requirements and timely deliver to the investigator for preservation.

The correctness of data is very important for the quality assurance of clinical study, and data management should be performed according to the GCP requirements of CFDA.

5.1 Data management for this study is the responsibility of the investigator initiating the study to ensure the authenticity, integrity, privacy, and traceability of the clinical study data.

5.2 The investigator shall record and keep the data correctly, completely, clearly and timely according to the original observation records of study subjects.

5.3 Data managers will establish corresponding logic check files to check the entered data according to the study protocol.

5.4 Data verification, lists of study subjects, summary tables, and statistical analyses were performed by a data manager or statistical analyst.

5.5 Data verification includes computerized programmatic verification, manual verification, and data review meetings, as necessary.

5.6 Any problem identified by DM shall be timely sent to the investigator for response in the form of Query Form, and all data queries and data corrections will be systematically tracked.

vii. References

1. 国家神经系统疾病临床医学研究中心等: **中国脑胶质瘤临床管理指南**: 人民卫生出版社; 2020.
2. Armstrong TS, Dirven L, Arons D, Bates A, Chang SM, Coens C, Espinasse C, Gilbert MR, Jenkinson D, Kluetz P *et al*: **Glioma patient-reported outcome assessment in clinical care and research: a Response Assessment in Neuro-Oncology collaborative report**. *LANCET ONCOL* 2020, **21**(2):e97-e103.
3. Group FBW: **BEST (Biomarkers, EndpointS, and other Tools) Resource**. Silver Spring (MD): Food and Drug Administration (US); 2016.
4. Basch E, Geoghegan C, Coons SJ, Gnanasakthy A, Slagle AF, Papadopoulos EJ, Kluetz PG: **Patient-Reported Outcomes in Cancer Drug Development and US Regulatory Review: Perspectives From Industry, the Food and Drug Administration, and the Patient**. *JAMA ONCOL* 2015, **1**(3):375-379.
5. Basch E, Deal AM, Kris MG, Scher HI, Hudis CA, Sabbatini P, Rogak L, Bennett AV, Dueck AC, Atkinson TM *et al*: **Symptom Monitoring With Patient-Reported Outcomes During Routine Cancer Treatment: A Randomised Controlled Trial**. *J CLIN ONCOL* 2016, **34**(6):557-565.
6. Yeung AR, Pugh SL, Klopp AH, Gil KM, Wenzel L, Westin SN, Gaffney DK, Small WJ, Thompson S, Doncals DE *et al*: **Improvement in Patient-Reported Outcomes With Intensity-Modulated Radiotherapy (RT) Compared With Standard RT: A Report From the NRG Oncology RTOG 1203 Study**. *J CLIN ONCOL* 2020, **38**(15):1685-1692.
7. Armstrong TS, Bishof AM, Brown PD, Klein M, Taphoorn MJ, Theodore-Oklota C: **Determining priority signs and symptoms for use as clinical outcomes assessments in trials including patients with malignant gliomas: Panel 1 Report**. *Neuro Oncol* 2016, **18 Suppl 2**:i1-i12.
8. Armstrong TS, Wefel JS, Wang M, Gilbert MR, Won M, Bottomley A, Mendoza TR, Coens C, Werner-Wasik M, Brachman DG *et al*: **Net clinical benefit analysis of radiation therapy oncology group 0525: a phase III trial comparing conventional adjuvant temozolomide with dose-intensive temozolomide in patients with newly diagnosed glioblastoma**. *J CLIN ONCOL* 2013, **31**(32):4076-4084.
9. Taphoorn M, Dirven L, Kanner AA, Lavy-Shahaf G, Weinberg U, Taillibert S, Toms SA, Honnorat J, Chen TC, Sroubek J *et al*: **Influence of Treatment With Tumour-Treating Fields on Health-Related Quality of Life of Patients With Newly Diagnosed Glioblastoma: A Secondary Analysis of a Randomised Clinical Trial**. *JAMA ONCOL* 2018, **4**(4):495-504.
10. Gilbert MR, Dignam JJ, Armstrong TS, Wefel JS, Blumenthal DT, Vogelbaum MA, Colman H, Chakravarti A, Pugh S, Won M *et al*: **A randomised trial of bevacizumab for newly diagnosed glioblastoma**. *N Engl J Med* 2014, **370**(8):699-708.
11. Armstrong TS, Dirven L, Arons D, Bates A, Chang SM, Coens C, Espinasse C, Gilbert MR, Jenkinson D, Kluetz P *et al*: **Glioma patient-reported outcome assessment in clinical care and research: a Response Assessment in Neuro-Oncology collaborative report**. *LANCET ONCOL* 2020, **21**(2):e97-e103.

12. Basch E, Reeve BB, Mitchell SA, Clauser SB, Minasian LM, Dueck AC, Mendoza TR, Hay J, Atkinson TM, Abernethy AP *et al*: **Development of the National Cancer Institute's patient-reported outcomes version of the common terminology criteria for adverse events (PRO-CTCAE)**. *J Natl Cancer Inst* 2014, **106**(9).
13. Dueck AC, Mendoza TR, Mitchell SA, Reeve BB, Castro KM, Rogak LJ, Atkinson TM, Bennett AV, Denicoff AM, O'Mara AM *et al*: **Validity and Reliability of the US National Cancer Institute's Patient-Reported Outcomes Version of the Common Terminology Criteria for Adverse Events (PRO-CTCAE)**. *JAMA ONCOL* 2015, **1**(8):1051-1059.
14. Trask PC, Dueck AC, Piault E, Campbell A: **Patient-Reported Outcomes version of the Common Terminology Criteria for Adverse Events: Methods for item selection in industry-sponsored oncology clinical trials**. *CLIN TRIALS* 2018, **15**(6):616-623.

VOPRONICA
倾听患者之声

Appendix 1 EORTC QLQ-C30 (V3.0) Chinese Scale and Scoring Rules

CHINESE MANDARIN (CHINA)

EORTC QLQ-C30 (V3)

我们想了解有关您和您的健康的一些情况，请您亲自回答下面所有问题，这里的答案并无“对”与“不对”之分，只要求在最能反映您情况的那个数字上画圈。您所提供的资料我们将会严格保密。

请填上您的姓名首位字母：____________

出生日期：____ 年___月___日

今天日期：____ 年___月___日

	没有	有点	相当	非常
1. 您从事一些费力的活动有困难吗，比如说提很重的购物袋或手提箱？	1	2	3	4
2. 长距离行走对您来说有困难吗？	1	2	3	4
3. 户外短距离行走对您来说有困难吗？	1	2	3	4
4. 您白天需要呆在床上或椅子上吗？	1	2	3	4
5. 您在吃饭、穿衣、洗澡或上厕所时需要他人帮忙吗？	1	2	3	4

**在过去的一星期内：**	没有	有点	相当	非常
6. 您在工作和其他日常活动中是否受到限制？	1	2	3	4
7. 您在从事您的爱好或休闲活动时是否受到限制？	1	2	3	4
8. 您有气短吗？	1	2	3	4
9. 您有疼痛吗？	1	2	3	4
10. 您需要休息吗？	1	2	3	4
11. 您睡眠有困难吗？	1	2	3	4
12. 您觉得虚弱吗？	1	2	3	4
13. 您食欲不振（没有胃口）吗？	1	2	3	4
14. 您觉得恶心吗？	1	2	3	4
15. 您有呕吐吗？	1	2	3	4
16. 您有便秘吗？	1	2	3	4

请接下页

VIRONICA 倾听患者之声

CHINESE MANDARIN (CHINA)

**在过去的一星期内:**	**没有**	**有点**	**相当**	**非常**
17．您有腹泻吗?	1	2	3	4
18．您觉得累吗?	1	2	3	4
19．疼痛影响您的日常活动吗?	1	2	3	4
20．您集中精力做事有困难吗，如读报纸或看电视?	1	2	3	4
21．您觉得紧张吗?	1	2	3	4
22．您觉得忧虑吗?	1	2	3	4
23．您觉得脾气急躁吗?	1	2	3	4
24．您觉得压抑（情绪低落）吗?	1	2	3	4
25．您感到记忆困难吗?	1	2	3	4
26．您的身体状况或治疗影响您的家庭生活吗?	1	2	3	4
27．您的身体状况或治疗影响您的社交活动吗?	1	2	3	4
28．您的身体状况或治疗使您陷入经济困难吗?	1	2	3	4

对下列问题，请在 1-7 之间选出一个最适合您的数字并画圈。

29．您如何评价在过去一星期内您总的健康情况?

1　2　3　4　5　6　7

非常差　　　　非常好

30．您如何评价在过去一星期内您总的生命质量?

1　2　3　4　5　6　7

非常差　　　　非常好

© Copyright 1995 EORTC Study Group on Quality of Life. All rights reserved. Version 3.0

The EORTC QLQ-C30 scale was introduced by the European Organisation for Research and Treatment of Cancer in 1993 as a cross-cultural and cross-national QOL-C30 (Quality of Life Questionnaire-Core 30). QOL was evaluated from a multidimensional point of view, which could better reflect the connotation of QOL and was applied to QOL measurement in cancer patients in multiple countries and regions in Europe.

Calculation of Scale Scores

The EORTC 's QLQ - C30 (V3.0) is a core scale for all cancer patients and consists of a multi-item scale and single-item measures. These scales include five functional scales (physical, role, cognitive, emotional, and social functioning), three symptom scales (fatigue, pain, nausea and vomiting), a global health status/quality of life scale, and six single item items (dyspnea, insomnia, loss of appetite, constipation, diarrhea, financial difficulties).

All scales and single item measures ranged between 0 - 100. A high score represents a more pronounced response:

A high score on the functional scale represents **a high level of functioning/well-being**,

High global health status/quality of life scores represent **high quality of life**,

But a high score on a symptom scale/item represents **more severe symptoms** presented by the patient.

- The **crude** score **(Raw Score, RS)** for each domain is obtained by summing the scores of the items included in each domain and dividing by the number of items included, i.e. RS = (Q1 + Q2 + Q3 + … Qn)/n.
- **Full distance (Rang e , R)** refers to the difference between the maximum possible value and the minimum possible value. Any item in QLQ-C30 has the same full range. Most entries have a score of 1- 4 (corresponding to none, a little, quite, very much, respectively), so the full distance is 3. However, the overall health status/quality of life score is 1- 7 (very bad - very good), so the full range is 6.
- A linear transformation is used to normalize the crude score to obtain **Score, S** , so that the score ranges from 0 to 100; a higher score indicates a higher level of functioning ("better"), or a higher level of symptoms ("worse"), i.e.:

Functional scale: S = [1- (RS-1)/R] × 100

Symptom Scale/Item: S = [(RS-1)/R] × 100

Global health status/quality of life: S = [(RS-1)/R] × 100

QLQ-C30 (V3.0) Scoring Method

Field	Scale Code	Number of entries	Full distance Range, R	V 3.0 Entry No.
**Global health status/quality of life**				
Global health status/quality of life	Q L2	2	6	2 9 , 30
**Functional scales**				
Physical function	P F2	5	3	1- 5
Role Function	R F2	2	3	6, 7
Emotional function	E F	4	3	2 1 - 24
Cognitive function	C F	2	3	2 0 , 25
Social functioning	S F	2	3	2 6 , 27
**Symptom scales/single item items**				

Fatigue	F A	3	3	1 0 , 12 , 18
Nausea and vomiting	N V	2	3	1 4 , 15
Pain	P A	2	3	9, 19
Dyspnoea	D Y	1	3	8
Insomnia	S L	1	3	1 1
Appetite loss	A P	1	3	1 3
Constipation	C O	1	3	1 6
Diarrhoea	D I	1	3	1 7
Economic difficulties	F I	1	3	2 8

VPRONICA 倾听患者之声

Appendix 2 NCI PRO-CTCAE Chinese Scale

NCI PRO-CTCAE Chinese version scale (full version) is provided in: https://healthcaredelivery.cancer.gov/pro-ctcae/

As individuals go through treatment for their cancer they sometimes experience different symptoms and side effects. For each question, please select the one response that best describes your experiences over the past 7 days…

**1a.** In the last 7 days, what was the SEVERITY of your DIFFICULTY SWALLOWING at its WORST?				
O None	O Mild	O Moderate	O Severe	O Very severe

**2a.** In the last 7 days, what was the SEVERITY of your PROBLEMS WITH TASTING FOOD OR DRINK at their WORST?				
O None	O Mild	O Moderate	O Severe	O Very severe

**3a.** In the last 7 days, what was the SEVERITY of your DECREASED APPETITE at its WORST?				
O None	O Mild	O Moderate	O Severe	O Very severe
**3b.** In the last 7 days, how much did DECREASED APPETITE INTERFERE with your usual or daily activities?				
O Not at all	O A little bit	O Somewhat	O Quite a bit	O Very much

**4a.** In the last 7 days, how OFTEN did you have NAUSEA?				
O Never	O Rarely	O Occasionally	O Frequently	O Almost constantly
**4b.** In the last 7 days, what was the SEVERITY of your NAUSEA at its WORST?				
O None	O Mild	O Moderate	O Severe	O Very severe

**5a.** In the last 7 days, how OFTEN did you have VOMITING?				
O Never	O Rarely	O Occasionally	O Frequently	O Almost constantly
**5b.** In the last 7 days, what was the SEVERITY of your VOMITING at its WORST?				
O None	O Mild	O Moderate	O Severe	O Very severe

**6a.** In the last 7 days, what was the SEVERITY of your CONSTIPATION at its WORST?				
O None	O Mild	O Moderate	O Severe	O Very severe

**7a.** In the last 7 days, what was the SEVERITY of your SHORTNESS OF BREATH at its WORST?				
O None	O Mild	O Moderate	O Severe	O Very severe
**7b.** In the last 7 days, how much did your SHORTNESS OF BREATH INTERFERE with your usual or daily activities?				
O Not at all	O A little bit	O Somewhat	O Quite a bit	O Very much

**8a.** In the last 7 days, how OFTEN did you feel a POUNDING OR RACING HEARTBEAT (PALPITATIONS)?				
O Never	O Rarely	O Occasionally	O Frequently	O Almost constantly
**8b.** In the last 7 days, what was the SEVERITY of your POUNDING OR RACING HEARTBEAT (PALPITATIONS) at its WORST?				
O None	O Mild	O Moderate	O Severe	O Very severe

**9a.** In the last 7 days, did you have any RASH?	
O Yes	O No

**10a.** In the last 7 days, did you have any HAIR LOSS?				
O Not at all	O A little bit	O Somewhat	O Quite a bit	O Very much

**11a.** In the last 7 days, what was the SEVERITY of your ITCHY SKIN at its WORST?				
O None	O Mild	O Moderate	O Severe	O Very severe

**12a.** In the last 7 days, did you have any HIVES (ITCHY RED BUMPS ON THE SKIN)?	
O Yes	O No

**13a.** In the last 7 days, did you have any BED SORES?	
O Yes	O No

**14a.** In the last 7 days, what was the SEVERITY of your SKIN BURNS FROM RADIATION at their WORST?					
O None	O Mild	O Moderate	O Severe	O Very severe	O Not applicable

VERONICA 倾听患者之声

15a. In the last 7 days, what was the SEVERITY of your NUMBNESS OR TINGLING IN YOUR HANDS OR FEET at its WORST?

O None	O Mild	O Moderate	O Severe	O Very severe

15b. In the last 7 days, how much did NUMBNESS OR TINGLING IN YOUR HANDS OR FEET INTERFERE with your usual or daily activities?

O Not at all	O A little bit	O Somewhat	O Quite a bit	O Very much

16a. In the last 7 days, what was the SEVERITY of your DIZZINESS at its WORST?

O None	O Mild	O Moderate	O Severe	O Very severe

16b. In the last 7 days, how much did DIZZINESS INTERFERE with your usual or daily activities?

O Not at all	O A little bit	O Somewhat	O Quite a bit	O Very much

17a. In the last 7 days, what was the SEVERITY of your BLURRY VISION at its WORST?

O None	O Mild	O Moderate	O Severe	O Very severe

17b. In the last 7 days, how much did BLURRY VISION INTERFERE with your usual or daily activities?

O Not at all	O A little bit	O Somewhat	O Quite a bit	O Very much

18a. In the last 7 days, did you have any FLASHING LIGHTS IN FRONT OF YOUR EYES?

O Yes	O No

19a. In the last 7 days, what was the SEVERITY of RINGING IN YOUR EARS at its WORST?

O None	O Mild	O Moderate	O Severe	O Very severe

20a. In the last 7 days, what was the SEVERITY of your PROBLEMS WITH CONCENTRATION at their WORST?

O None	O Mild	O Moderate	O Severe	O Very severe

20b. In the last 7 days, how much did PROBLEMS WITH CONCENTRATION INTERFERE with your usual or daily activities?

O Not at all	O A little bit	O Somewhat	O Quite a bit	O Very much

21a. In the last 7 days, what was the SEVERITY of your PROBLEMS WITH MEMORY at their WORST?

O None	O Mild	O Moderate	O Severe	O Very severe

21b. In the last 7 days, how much did PROBLEMS WITH MEMORY INTERFERE with your usual or daily activities?

O Not at all	O A little bit	O Somewhat	O Quite a bit	O Very much

22a. In the last 7 days, how OFTEN did you have PAIN?

O Never	O Rarely	O Occasionally	O Frequently	O Almost constantly

22b. In the last 7 days, what was the SEVERITY of your PAIN at its WORST?

O None	O Mild	O Moderate	O Severe	O Very severe

22c. In the last 7 days, how much did PAIN INTERFERE with your usual or daily activities?

O Not at all	O A little bit	O Somewhat	O Quite a bit	O Very much

23a. In the last 7 days, how OFTEN did you have a HEADACHE?

O Never	O Rarely	O Occasionally	O Frequently	O Almost constantly

23b. In the last 7 days, what was the SEVERITY of your HEADACHE at its WORST?

O None	O Mild	O Moderate	O Severe	O Very severe

23c. In the last 7 days, how much did your HEADACHE INTERFERE with your usual or daily activities?

O Not at all	O A little bit	O Somewhat	O Quite a bit	O Very much

24a. In the last 7 days, what was the SEVERITY of your INSOMNIA (INCLUDING DIFFICULTY FALLING ASLEEP, STAYING ASLEEP, OR WAKING UP EARLY) at its WORST?

O None	O Mild	O Moderate	O Severe	O Very severe

24b. In the last 7 days, how much did INSOMNIA (INCLUDING DIFFICULTY FALLING ASLEEP, STAYING ASLEEP, OR WAKING UP EARLY) INTERFERE with your usual or daily activities?

O Not at all	O A little bit	O Somewhat	O Quite a bit	O Very much

25a. In the last 7 days, what was the SEVERITY of your FATIGUE, TIREDNESS, OR LACK OF ENERGY at its WORST?

O None	O Mild	O Moderate	O Severe	O Very severe

25b. In the last 7 days, how much did FATIGUE, TIREDNESS, OR LACK OF ENERGY INTERFERE with your usual or daily activities?

O Not at all	O A little bit	O Somewhat	O Quite a bit	O Very much

26a. In the last 7 days, how OFTEN did you feel ANXIETY?

O Never	O Rarely	O Occasionally	O Frequently	O Almost constantly

26b. In the last 7 days, what was the SEVERITY of your ANXIETY at its WORST?

O None	O Mild	O Moderate	O Severe	O Very severe

26c. In the last 7 days, how much did ANXIETY INTERFERE with your usual or daily activities?

O Not at all	O A little bit	O Somewhat	O Quite a bit	O Very much

27a. In the last 7 days, how OFTEN did you FEEL THAT NOTHING COULD CHEER YOU UP?

O Never	O Rarely	O Occasionally	O Frequently	O Almost constantly

27b. In the last 7 days, what was the SEVERITY of your FEELINGS THAT NOTHING COULD CHEER YOU UP at their WORST?

O None	O Mild	O Moderate	O Severe	O Very severe

27c. In the last 7 days, how much did FEELING THAT NOTHING COULD CHEER YOU UP INTERFERE with your usual or daily activities?

O Not at all	O A little bit	O Somewhat	O Quite a bit	O Very much

28a. In the last 7 days, how OFTEN did you have LOSS OF CONTROL OF URINE (LEAKAGE)?

O Never	O Rarely	O Occasionally	O Frequently	O Almost constantly

28b. In the last 7 days, how much did LOSS OF CONTROL OF URINE (LEAKAGE) INTERFERE with your usual or daily activities?

O Not at all	O A little bit	O Somewhat	O Quite a bit	O Very much

29a. In the last 7 days, what was the SEVERITY of your DECREASED SEXUAL INTEREST at its WORST?

O None	O Mild	O Moderate	O Severe	O Very severe	O Not sexually active	O Prefer not to answer

**30a.** In the last 7 days, did you have an UNEXPECTED DECREASE IN SWEATING?	
O Yes	O No

**31a.** In the last 7 days, how OFTEN did you have HOT FLASHES/FLUSHES?				
O Never	O Rarely	O Occasionally	O Frequently	O Almost constantly
**31b.** In the last 7 days, what was the SEVERITY of your HOT FLASHES/FLUSHES at their WORST?				
O None	O Mild	O Moderate	O Severe	O Very severe

**OT HER SYMPTOMS**	
Do you have any other symptoms that you wish to report?	
O Yes	O No
**Please list any other symptoms:**	
1.	In the last 7 days, what was the SEVERITY of this symptom at its WORST?<br>O None O Mild O Moderate O Severe O Very Severe
2.	In the last 7 days, what was the SEVERITY of this symptom at its WORST?<br>O None O Mild O Moderate O Severe O Very Severe
3.	In the last 7 days, what was the SEVERITY of this symptom at its WORST?<br>O None O Mild O Moderate O Severe O Very Severe
4.	In the last 7 days, what was the SEVERITY of this symptom at its WORST?<br>O None O Mild O Moderate O Severe O Very Severe
5.	In the last 7 days, what was the SEVERITY of this symptom at its WORST?<br>O None O Mild O Moderate O Severe O Very Severe

Patient-Reported Outcomes version Of The Common Terminology Criteria For Adverse Events (PRO-CTCAE™)

QUICK GUIDE TO THE ITEM LIBRARY*

Oral	
Dry mouth	S
Difficulty swallowing	S
Mouth/throat sores	SI
Cracking at the corners of the mouth (cheilosis/cheilitis)	S
Voice quality changes	P
Hoarseness	S

Gastrointestinal	
Taste changes	S
Decreased appetite	SI
Nausea	FS
Vomiting	FS
Heartburn	FS
Gas	P
Bloating	FS
Hiccups	FS
Constipation	S
Diarrhea	F
Abdominal pain	FSI
Fecal incontinence	FI

Respiratory	
Shortness of breath	SI
Cough	SI
Wheezing	S

Cardio/Circulatory	
Swelling	FSI
Heart palpitations	FS

Cutaneous	
Rash	P
Skin dryness	S
Acne	S
Hair loss	A
Itching	S
Hives	P
Hand-foot syndrome	S
Nail loss	P
Nail ridging	P
Nail discoloration	P
Sensitivity to sunlight	P
Bed/pressure sores	P
Radiation skin reaction	S
Skin darkening	P
Stretch marks	P

Neurological	
Numbness & tingling	SI
Dizziness	SI

Visual/Perceptual	
Blurred vision	SI
Flashing lights	P
Visual floaters	P
Watery eyes	SI
Ringing in ears	S

Attention/Memory	
Concentration	SI
Memory	SI

Pain	
General pain	FSI
Headache	FSI
Muscle pain	FSI
Joint pain	FSI

Sleep/Wake	
Insomnia	SI
Fatigue	SI

Mood	
Anxious	FSI
Discouraged	FSI
Sad	FSI

Genitourinary	
Irregular periods/vaginal bleeding	P
Missed expected menstrual period	P
Vaginal discharge	A
Vaginal dryness	S
Painful urination	S
Urinary urgency	FI
Urinary frequency	FI
Change in usual urine color	P
Urinary incontinence	FI

Sexual	
Achieve and maintain erection	S
Ejaculation	F
Decreased libido	S
Delayed orgasm	P
Unable to have orgasm	P
Pain w/sexual intercourse	S

Miscellaneous	
Breast swelling and tenderness	S
Bruising	P
Chills	FS
Increased sweating	FS
Decreased sweating	P
Hot flashes	FS
Nosebleed	FS
Pain and swelling at injection site	P
Body odor	S

Attributes	
F: Frequency	I: Interference
S: Severity	P: Presence/Absence
A: Amount	

NIH National Cancer Institute

*Complete library of items available at: https://healthcaredelivery.cancer.gov/pro-ctcae

Version date: 3/11/2020

The NCI developed PRO-CTCAE (Common Terminology for Adverse Events Patient Self-Report Version) from CTCAE, which became available in 2016, and has developed into a standardized measurement system that provides flexible and applicable methods for treatment, regulatory, and health policy decisions to assess associated symptomatic adverse events in the treatment of various cancers.

In April 2019, NCI published the official Chinese version of PRO-CTCAE. PRO-CTCAE has 124 items and 78 symptoms to record adverse events within 7 days. It is highly modular and requires the selection of the corresponding modules based on clinical practice and clinical trials.

倾听患者之声

Appendix 3 Customized PRO-CTCAE and C TCAE 5.0 Entry Correspondence Table

P RO-CTCAE	C TCAE
**Dysphagia**	Dysphagia
**Taste problems when tasting food or drink**	Dysgeusia
**Decreased appetite**	Anorexia
**Queasy (nausea/regurgitation)**	Nausea
**Vomiting**	Vomiting
**Constipation**	Constipation
**Tachypnea**	Dyspnoea
**Beating or racing heart (palpitations)**	Palpitations
**Paralysis or tingling in hands or feet**	Peripheral sensory nerve disorder
**Dizziness**	Vertigo
**Blurred vision**	Blurred vision
**Flare in front of eyes**	Flash
**Tinnitus (noise in ears)**	Tinnitus
**Inability to concentrate**	Attention disturbance
**Memory (memory) problems**	Memory impairment
**Skin erythema**	Maculopapular rash
**Alopecia**	Alopecia
**Skin pruritus**	Pruritus
**Measles (itchy red pimples on the skin)**	Urticaria
**Bedsores/Pressure sores**	Skin ulcer
**Burn of skin by radiation**	Radiation dermatitis
**Pain (may appear anywhere on the body)**	Pain * (may occur anywhere on the body)
**Headache**	Headache
**Insomnia (including difficulty falling asleep, staying asleep or waking up early)**	Insomnia
**Fatigue (tiredness), tiredness, or lack of energy**	Fatigue
**Anxiety**	Anxiety
**Nothing can cheer you up/cheer you up**	Depression
**Decreased sexual interest**	Libido decreased
**Loss of control of urination (urine leakage)**	Urinary incontinence
**Abnormal decreased sweating**	Hypohidrosis
**Hot flashes (sensation of heat and sweating or rapid heartbeat)**	Hyperhidrosis

Attachment 4 Customized PRO-CTCAE Criteria for C TCAE 5.0

Entry Grading

CTCAE ADVERSE EVENTS	GRADE 1	GRADE 2	GRADE 3	GRADE 4
**DYSPHAGIA**	SYMPTOMATIC, ABLE TO EAT NORMALLY	SYMPTOMS, CHANGE IN EATING AND SWALLOWING HABITS	SEVERE CHANGE IN EATING AND SWALLOWING HABITS; REQUIRING NASOGASTRIC FEEDING, TOTAL PARENTERAL NUTRITION, OR HOSPITALIZATION	LIFE-THREATENING; URGENT TREATMENT REQUIRED
**DYSGEUSIA**	CHANGE IN TASTE WITHOUT AFFECTING NORMAL DIET	CHANGE IN TASTE AND AFFECT NORMAL DIET (EG, ORAL SUPPLEMENTS); TOXIC OR UNCOMFORTABLE TASTE; LOSS OF TASTE	-	-
**ANOREXIA**	DECREASED APPETITE WITHOUT CHANGE IN EATING HABITS	CHANGE IN FOOD INTAKE WITHOUT WEIGHT LOSS OR MALNUTRITION; ORAL NUTRITIONAL SUPPLEMENTATION REQUIRED	SIGNIFICANT WEIGHT LOSS OR MALNUTRITION (EG, INADEQUATE ORAL CALORIC INTAKE AND/OR FLUID INTAKE); REQUIRING NASOGASTRIC OR TOTAL PARENTERAL NUTRITION	LIFE-THREATENING; URGENT TREATMENT REQUIRED
**NAUSEA**	DECREASED APPETITE WITHOUT CHANGE IN EATING HABITS	DECREASED ORAL INTAKE WITHOUT SIGNIFICANT BODY WEIGHT LOSS, DEHYDRATION, OR MALNUTRITION	INADEQUATE ORAL INTAKE OF ENERGY AND WATER; NASOGASTRIC FEEDING, TOTAL PARENTERAL NUTRITION, OR HOSPITALIZATION REQUIRED	-
**VOMITING**	INTERVENTION NOT REQUIRED	OUTPATIENT INTRAVENOUS FLUIDS; MEDICAL INTERVENTION INDICATED	NEED FOR NASOGASTRIC FEEDING, TOTAL PARENTERAL NUTRITION OR HOSPITALIZATION	LIFE THREATENING

CTCAE ADVERSE EVENTS	GRADE 1	GRADE 2	GRADE 3	GRADE 4
**CONSTIPATION**	OCCASIONAL OR INTERMITTENT; OCCASIONALLY REQUIRING USE OF STOOL SOFTENERS, LAXATIVES, DIETARY MODIFICATION, OR ENEMAS	PERSISTENT SYMPTOMS REQUIRING REGULAR LAXATIVE OR ENEMAS; LIMITING ACTIVITIES OF DAILY LIVING WITH TOOLS	OBSTINATE CONSTIPATION REQUIRING MANUAL DREDGING; LIMITING SELF-CARE ACTIVITIES OF DAILY LIVING	LIFE-THREATENING; URGENT TREATMENT REQUIRED
**DYSPNOEA**	SHORTNESS OF BREATH WITH MODERATE ACTIVITY	SHORTNESS OF BREATH WITH MINIMAL ACTIVITY; IMPACTS ACTIVITIES OF DAILY LIVING WITH TOOLS	SHORTNESS OF BREATH AT REST; AFFECTS SELF-CARE ACTIVITIES OF DAILY LIVING	LIFE-THREATENING; URGENT TREATMENT REQUIRED
**PALPITATIONS**	MILD SYMPTOMS; NO TREATMENT REQUIRED	NEED FOR TREATMENT	-	-
**PERIPHERAL SENSORY NERVE DISORDER**	ASYMPTOMATIC	MODERATE; IMPACTS INSTRUMENTAL ACTIVITIES OF DAILY LIVING	SEVERE SYMPTOMS; LIMITING SELF-CARE ABILITY	LIFE-THREATENING; URGENT INTERVENTION INDICATED
**VERTIGO**	MILD SYMPTOMS	MODERATE; IMPACTS INSTRUMENTAL ACTIVITIES OF DAILY LIVING	SEVERE SYMPTOMS; AFFECT SELF-CARE ACTIVITIES OF DAILY LIVING	-
**BLURRED VISION**	NO TREATMENT REQUIRED	SYMPTOMATIC; MODERATE DECREASE IN VISUAL ACUITY (BEST-CORRECTED VISUAL ACUITY ≥ 20/40 AND	SYMPTOMATIC WITH SEVERE DECREASE IN VISUAL ACUITY (BEST-CORRECTED VISUAL ACUITY < 20/40, OR VISUAL FIELD ≥ 3 LINES FROM KNOWN BASELINE TO 20/200); LIMITING	BEST-CORRECTED VISUAL ACUITY ON AFFECTED SIDE < 20/200

CTCAE ADVERSE EVENTS	GRADE 1	GRADE 2	GRADE 3	GRADE 4
		VISUAL FIELD REDUCTION OF < 3 LINES FROM KNOWN BASELINE); LIMITATION OF ACTIVITIES OF DAILY LIVING WITH TOOLS	SELF-CARE ACTIVITIES OF DAILY LIVING	
**FLASH**	SYMPTOMATIC BUT NOT LIMITING ACTIVITIES OF DAILY LIVING	LIMITED ACTIVITIES OF DAILY LIVING WITH TOOLS	LIMITATION OF SELF-CARE ACTIVITIES OF DAILY LIVING	-
**TINNITUS**	MILD SYMPTOMS; NO TREATMENT REQUIRED	MODERATE; IMPACTS INSTRUMENTAL ACTIVITIES OF DAILY LIVING	SEVERE SYMPTOMS; AFFECT SELF-CARE ACTIVITIES OF DAILY LIVING	-
**ATTENTION DISTURBANCE**	MILD IMPAIRMENT OF CONCENTRATION OR REDUCED LEVEL OF CONCENTRATION	MODERATE IMPAIRMENT OF CONCENTRATION OR MODERATE REDUCTION IN THE LEVEL OF CONCENTRATION; AFFECTING INSTRUMENTAL ACTIVITIES OF DAILY LIVING	SEVERE IMPAIRMENT OF CONCENTRATION OR SEVERELY REDUCED LEVEL OF CONCENTRATION; AFFECTING SELF-CARE ACTIVITIES OF DAILY LIVING	-
**MEMORY IMPAIRMENT**	MILD MEMORY IMPAIRMENT	MODERATE MEMORY IMPAIRMENT; INSTRUMENTAL ACTIVITIES OF DAILY LIVING	SEVERE MEMORY IMPAIRMENT, IMPAIRED SELF-CARE ABILITY	-
**MACULOPAPULAR RASH**	MACULOPAPULAR RASH COVERING LESS THAN 10% OF	MACULOPAPULAR RASH COVERING 10-30% OF BODY SURFACE	PAPULES AND/OR PUSTULES COVERING GREATER THAN 30% OF BODY SURFACE WITH MODERATE TO	-

CTCAE ADVERSE EVENTS	GRADE 1	GRADE 2	GRADE 3	GRADE 4
	BODY SURFACE WITH/WITHOUT SYMPTOMS (E.G. ITCHING, BURNING, TIGHTNESS)	WITH/WITHOUT SYMPTOMS (E.G., ITCHING, BURNING, TIGHTNESS); AFFECTING INSTRUMENTAL ACTIVITIES OF DAILY LIVING; RASH COVERING GREATER THAN 30% OF BODY SURFACE WITH OR WITHOUT MINOR SYMPTOMS	SEVERE SYMPTOMS; AFFECTING SELF-CARE ACTIVITIES OF DAILY LIVING	
**ALOPECIA**	INDIVIDUAL HAIR LOSS WAS LESS THAN 50%, WHICH WAS NOT SIGNIFICANTLY DIFFERENT FROM DISTANT OBSERVATION, BUT WAS VISIBLE AT CLOSE RANGE. HAIR STYLE CHANGES ARE NEEDED TO COVER UP HAIR LOSS, BUT NO FAKE HAIR OR WIG CLUSTERS TO HIDE	INDIVIDUAL HAIR LOSS GREATER THAN OR EQUAL TO 50% WITH SIGNIFICANT SYMPTOMS; REQUIRING WIGS OR CLUSTERS OF WIGS IF PATIENT WANTS TO COMPLETELY COVER UP HAIR LOSS; WITH PSYCHOLOGICAL IMPACT	-	-
**PRURITUS**	MILD OR LOCALIZED; REQUIRES LOCAL TREATMENT	WIDELY DISTRIBUTED AND INTERMITTENT; SKIN	WIDELY DISTRIBUTED AND PERSISTENT EPISODES; AFFECTING SELF-	

CTCAE ADVERSE EVENTS	GRADE 1	GRADE 2	GRADE 3	GRADE 4
	T	CHANGES CAUSED BY SCRATCHING (E.G. EDEMA, PAPULES, SCRATCHES, LICHENIFICATION, EXUDATION/CRUSTS); REQUIRES ORAL MEDICATION; EFFECTS INSTRUMENTAL ACTIVITIES OF DAILY LIVING	CARE ACTIVITIES OF DAILY LIVING OR SLEEP; REQUIRING SYSTEMIC CORTICOSTEROIDS OR IMMUNOSUPPRESSANTS.	
**URTICARIA**	URTICARIA INVOLVING LESS THAN 10% OF BODY SURFACE AREA; REQUIRES TOPICAL TREATMENT	URTICARIA LESIONS COVERING 10-30% OF BODY SURFACE AREA; ORAL THERAPY REQUIRED	URTICARIA INVOLVING GREATER THAN 30% OF BODY SURFACE AREA; REQUIRES INTRAVENOUS THERAPY	-
**SKIN ULCER**	AREA OF ULCERATION LESS THAN 1 CM; ERYTHEMA NOT BLANCHED, SKIN INTACT, WITH FEVER AND EDEMA	AREA OF ULCERATION BETWEEN 1 AND 2 CM; PARTIAL LOSS OF SKIN LAYER INVOLVING SUBCUTANEOUS TISSUE OR SUBCUTANEOUS ADIPOSE TISSUE	AREA OF ULCERATION GREATER THAN 2 CM; LOSS OF FULL THICKNESS OF SKIN INVOLVING DISRUPTION OR NECROSIS OF SUBCUTANEOUS TISSUE, WHICH MAY EXTEND INTO THE FASCIAL LAYER	ULCER OF ANY SIZE, WITH EXTENSIVE TISSUE DESTRUCTION, TISSUE NECROSIS OR DAMAGE TO MUSCLE, BONE, OR SUPPORTING TISSUE, WITH/WITHOUT LOSS OF FULL THICKNESS SKIN
**RADIATION DERMATITIS**	MILD ERYTHEMA OR DESQUAMA	MODERATE TO SEVERE ERYTHEMA; PATCHY	WET DESQUAMATION NOT CONFINED TO WRINKLES AND	LIFE-THREATENING; SKIN NECROSIS

CTCAE ADVERSE EVENTS	GRADE 1	GRADE 2	GRADE 3	GRADE 4
	TION SICCA	MOIST DESQUAMATION, MOSTLY LOCALIZED IN WRINKLES AND FOLDS; MODERATE EDEMA	FOLDS; BLEEDING CAUSED BY MINOR TRAUMA OR FRICTION	OR ULCERATION OF THE TRUE LAYER; BLEEDING FROM A COMPROMISED SITE; SKIN GRAFT REQUIRED
**PAIN * (MAY OCCUR ANYWHERE ON THE BODY)**	MILD PAIN	MODERATE PAIN; AFFECTING INSTRUMENTAL ACTIVITIES OF DAILY LIVING	SEVERE PAIN; AFFECTING SELF-CARE ACTIVITIES OF DAILY LIVING	-
**HEADACHE**	MILD PAIN	MODERATE PAIN; AFFECTING INSTRUMENTAL ACTIVITIES OF DAILY LIVING	SEVERE PAIN; AFFECTING SELF-CARE ACTIVITIES OF DAILY LIVING	-
**INSOMNIA**	MILD DIFFICULTY SLEEPING, STAYING ASLEEP OR WAKING UP EARLY	MODERATE DIFFICULTY SLEEPING, STAYING ASLEEP OR WAKING UP EARLY	SEVERE DIFFICULTY SLEEPING, STAYING ASLEEP OR WAKING UP EARLY	-
**FATIGUE**	FATIGUE, RELIEVED BY REST	FATIGUE, NOT RELIEVED BY REST; IMPACTS DAILY HOUSEWORK ACTIVITIES	FATIGUE, NOT RELIEVED BY REST; AFFECTS SELF-CARE ACTIVITIES OF DAILY LIVING	**-**
**ANXIETY**	MILD SYMPTOMS; NO TREATMENT REQUIRED	MODERATE; IMPACTS INSTRUMENTAL ACTIVITIES OF DAILY LIVING	SEVERE SYMPTOMS; INFLUENCE SELF-CARE ACTIVITIES OF DAILY LIVING; HOSPITALIZATION REQUIRED	LIFE-THREATENING; URGENT INTERVENTION INDICATED
**DEPRESSION**	MILD SYMPTOMS	MODERATE SYMPTOMS; IMPACTS INSTRUMENTAL ACTIVITIES OF DAILY LIVING	SEVERE SYMPTOMS; LIMITING SELF-CARE; HOSPITALIZATION NOT INDICATED	LIFE-THREATENING, JEOPARDIZING SELF OR OTHERS;

CTCAE ADVERSE EVENTS	GRADE 1	GRADE 2	GRADE 3	GRADE 4
				HOSPITALIZATION INDICATED
**LIBIDO DECREASED**	DECREASED SEXUAL INTEREST, NO IMPACT ON RELATIONSHIP	DECREASED SEXUAL INTEREST, IMPACTS RELATIONSHIP	-	-
**URINARY INCONTINENCE**	OCCASIONALLY (E.G. COUGHING, SNEEZING, ETC.), NO PADS REQUIRED	SPONTANEOUS; REQUIRES PADS; LIMITING INSTRUMENTAL ACTIVITIES OF DAILY LIVING	INTERVENTION REQUIRED (CLIP, COLLAGEN INJECTION); SURGICAL INTERVENTION REQUIRED; LIMITING SELF-CARE ACTIVITIES OF DAILY LIVING	-
**HYPOHIDROSIS**	-	SYMPTOMATIC; IMPACTS INSTRUMENTAL ACTIVITIES OF DAILY LIVING	BODY TEMPERATURE INCREASED; AFFECTS SELF-CARE ACTIVITIES OF DAILY LIVING	HEATSTROKE
**HYPERHIDROSIS**	LIMITED TO ONE AREA (PALM, SOLE, AXILLA); REQUIRES PERSONAL HYGIENE	MORE THAN ONE SITE; PATIENT REQUIRES MEDICATION; WITH PSYCHOLOGICAL IMPACT	WITH ELECTROLYTE/HAEMODYNAMIC IMBALANCE	-

VERONICA 倾听患者之声
